# A Contrastive-Learning-Based Pre-Training Framework for Optical Property Prediction of Low-Data Rhodamines with Interpretable Multitask Graph Neural Networks

**DOI:** 10.3390/molecules31071149

**Published:** 2026-03-31

**Authors:** Jiangguo Qiu, Yanling Wu, Hong Zhang, Menglong Li, Xuemei Pu, Yanzhi Guo

**Affiliations:** 1 College of Chemistry, Sichuan University, Chengdu 610064, China; qiu_jiangguo@stu.scu.edu.cn (J.Q.); 2024322030088@stu.scu.edu.cn (Y.W.); liml@scu.edu.cn (M.L.); xmpuscu@scu.edu.cn (X.P.); 2 Functional and Molecular Imaging Key Laboratory of Sichuan Province, Department of Radiology and Huaxi MR Research Center (HMRRC), West China Hospital, Sichuan University, Chengdu 610041, China; zhanghong94@scu.edu.cn

**Keywords:** rhodamine derivatives, fluorescence wavelength prediction, contrastive learning, multitask, interpretability

## Abstract

Accurate prediction of maximum absorption (*λ*_abs_) and emission (*λ*_emi_) wavelengths are essential for the design of high-performance rhodamine probes. However, available rhodamine optical data is extremely limited and heterogeneous, posing challenges for deep learning models. Here, we developed a contrastive-learning-based multitask graph neural networks framework to predict *λ*_abs_ and *λ*_emi_ of rhodamine derivatives, using a multi-modal feature by integrating atom–bond level graph representations with solvent descriptors. The model is first pre-trained on 48,148 xanthene-derived molecules with a self-supervised contrastive strategy, as well as fine-tuning on a curated rhodamine dataset containing 390 molecule–solvent pair samples. It yields excellent performance with R^2^ of 0.923 for *λ*_abs_ and 0.913 for *λ*_emi_, respectively, outperforming machine learning, single-task, and no-pre-training GNN baselines. External dataset tests and comparisons with theoretical calculations reveal the superiority of our proposed model. Then, attention-based interpretability identifies chemically meaningful regions, including the conjugated backbone and amino substituents, which is consistent with the known photophysical mechanisms. Finally, we designed three new rhodamine derivatives exhibiting high Stokes shifts, with minimum 9 nm deviation between predicted and experimental values. These findings demonstrate that this framework enables accurate fluorescence property prediction and mechanism-informed molecular design, offering a promising theoretical guide for designing next-generation probes.

## 1. Introduction

Fluorescent probes, because of their high brightness, specificity, and sensitivity, are widely applied in bioimaging, disease diagnosis, and environmental monitoring [1,2,3]. As the key optical parameters defining dye suitability, *λ*_abs_ and *λ*_emi_ determine the imaging quality and sensing range of fluorescent molecules [4]. Accordingly, precise control and accurate prediction of *λ*_abs_ and *λ*_emi_ not only influence the performance optimization of individual probes but also govern the overall designability and scalability of multicolor systems [5].

The traditional design paradigm for fluorescent molecules relies primarily on chemical intuition, such as introducing substituents with different electronic/conjugation characteristics onto a given scaffold to tune energy levels and resonance, and on screening derivatives through a synthesize-measure cycle [6,7]. However, this strategy is costly, time-consuming, and explores a vast yet incomplete searching space [7]. Quantum mechanical (QM) calculations can, in principle, provide theoretical support for energy levels and electronic transitions, but dual limitations in cost and applicability remain when dealing with complex solvents/conformational sampling, system size, and generalization to novel scaffolds [4,8]. In recent years, machine learning (ML) and deep learning (DL) methods have offered new avenues for rapid screening and goal-directed design by learning structure–property mappings directly from data [8,9]. Compared with classical ML and other DL approaches based on molecular fingerprints or hand-crafted descriptors, graph neural networks (GNNs) operate on molecular graphs to joint model local and global topology with fewer prior assumptions, making them well suited to capture the multiscale coupling of “local substitution–global resonance–solvent response” in conjugated systems [10,11,12,13].

Most existing studies build predictors on broad fluorescent-molecule repositories and emphasize average performance across diverse scaffolds [14,15,16]. Yet probe families such as rhodamines, cyanines, and coumarins differ substantially in their parent conjugated frameworks, electron-donating/withdrawing patterns, switchable tautomerism and responses to solvent polarity, and hydrogen-bonding environments [6,7,17,18,19]. A single universal model often fails to capture family-specific, fine-grained structure–property rules, thereby limiting predictive accuracy, extrapolation stability, and interpretability for a given target family. Rhodamines, as widely used and highly engineerable probes, particularly benefit from precise modeling of *λ*_abs_/*λ*_emi_, which not only improves optimization efficiency for existing molecules but also helps elucidate, at a mechanistic level, the cooperative effects of substitution, conformation, and solvent on spectral responses [2,7,20]. Constructing a rhodamine-specific model and data resource is therefore a critical step toward customized prediction and targeted molecular design.

Given experimental limitations, available optical data for rhodamine derivatives are relatively scarce and scattered, posing challenges for data-driven DL. Nevertheless, GNNs exhibit strong adaptability across tasks and natural advantages in molecular representation learning [12]. Recent application of GNNs in quantum chemistry and molecular dynamics simulations have further demonstrated that graph-based learning can achieve high data efficiency and strong transferability even for complex electronic structure targets [21,22]. Leveraging recent advances in transfer learning and multitask learning [12,23,24,25], GNNs can be expected to achieve high performance on low-data rhodamines.

**Figure 1:**
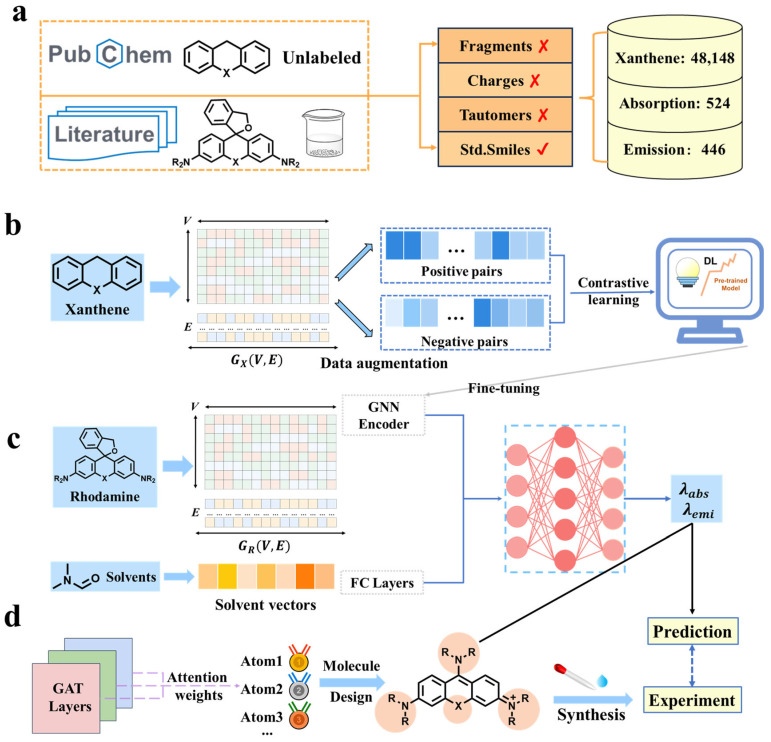
Overall workflow for predicting *λ*_abs_ and *λ*_emi_ of rhodamine derivatives using GNNs. (**a**) Curation of the xanthene dataset for the pre-training model and an integrated rhodamine-solvent dataset. ✘/✓ indicates removal or retention. (**b**) Construction of a self-supervised contrastive-learning-based pre-training model using xanthene data. (**c**) Feature representation and *λ*_abs_/*λ*_emi_ fine-tuning predictions. (**d**) Molecular design and synthesis of new rhodamines guided by the interpretability analysis based on graph attention weights.

In this study, we developed a multitask GNN framework based on contrastive learning, integrating molecular graph representations with solvent descriptors to accurately and interpretably predict *λ*_abs_ and *λ*_emi_ of rhodamines. Figure 1 illustrates overall workflow of this research. Figure 1a shows that a big dataset of 48,148 rhodamine parent-core molecules (xanthenes) was compiled for contrastive pre-training and an integrated rhodamine dataset to systematically characterize the optical properties was curated, including 524 *λ*_abs_ records for 421 molecules and 446 *λ*_emi_ records for 347 molecules across 17 common solvents, while preserving the sample mapping relationships among molecules, solvent and measurement conditions. Considering the limited scale of rhodamine data, the big xanthenes dataset was firstly used to perform a self-supervised contrastive pre-training to encourage the encoder to capture scaffold and substitution site information (Figure 1b), which is beneficial for downstream *λ*_abs_/*λ*_emi_ prediction, followed by supervised fine-tuning on the rhodamine-specific dataset (Figure 1c). The multi-modal features integrating graph feature and solvent information were presented to rationally represent rhodamine samples. For molecular graph construction, the 8-dimensional atom-level features were employed as graph node inputs that captured the key properties associated with conjugation/substitution effects. For solvent information, a compact vector representation encompassing 7 descriptors were proposed (Figure 1c). In view of the intrinsic linkage between absorption and emission via the electronic energy-level structure, a multitask framework was developed with a shared GNN encoder and task-specific regression heads. To address concerns about the interpretability of DL models, post hoc structural contribution analyses were conducted using graph attention weight attributions, localizing functional groups and substitution sites with high model sensitivity and relating them to the directions of *λ*_abs_/*λ*_emi_ shifts (Figure 1d). Comprehensive evaluation proves that the proposed pre-training multitask framework achieves stable and superior performance on both *λ*_abs_ and *λ*_emi_ predictions with R^2^ over 0.90, which reveals significant gains over the single-task and no-pre-training GNN models as well as classical ML baselines. Guided by the developed model, three candidate structures were designed and synthesized, indicating practical utility of the model in the design and screening of novel rhodamines.

## 2. Results and Discussion

### 2.1. Evaluation of the Contrastive Pre-Trained Model Representation

**Figure 2:**
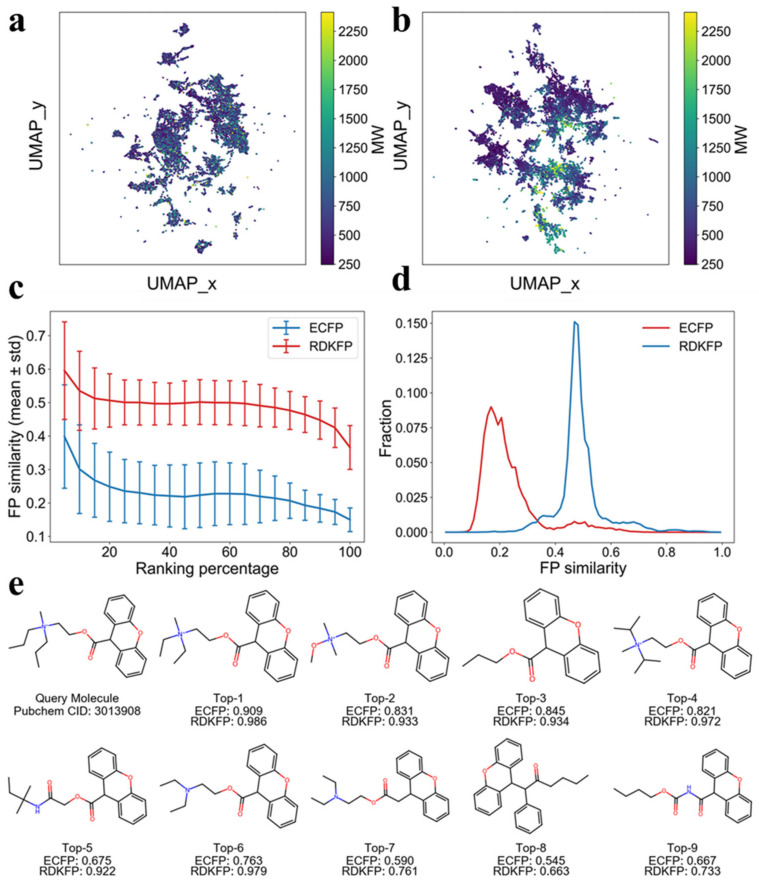
Analysis on the pre-trained model representation. (**a**,**b**) UMAP visualization of pre-trained molecular representations from a randomly initialized model and the contrastive learning pre-trained model, respectively. Representations were extracted from the GAT-based contrastive pre-training model, and each point is colored by molecular weight (g·mol^−1^). (**c**) Relationships between ECFP/RDKFP similarities and embedding distances in the pre-trained representation space. (**d**) Distributions of fingerprint similarities between the query and all other molecules. (**e**) Fingerprint similarities of the 9 nearest neighbors to the query in the pre-trained representation space.

Supplementary Figure S1 gives the loss curves of the pre-trained models based on graph convolutional networks (GCNs) and graph attention networks (GATs), respectively, which proves the stable optimization and good convergence of the models. However, the major objective of contrastive learning is to maximize the agreement between different augmented views of the same molecule while reducing the similarity to representations of other molecules. Here, Uniform Manifold Approximation and Projection (UMAP) 31 was employed to evaluate the molecular representations learned by the contrastive pre-trained model. UMAP projects high-dimensional embeddings into two dimensions by constructing a k-nearest neighbor graph using cosine distances and optimizing low-dimensional coordinates to preserve local neighborhood relations.

(1) $$d_{cos}\left(z_{i},z_{j}\right)=1-\frac{z_{i}^{⊤}z_{j}}{∥z_{i}∥∥z_{j}∥},$$

where *z_i_* and *z_j_* are sample embeddings, and *d_cos_* quantifies their normalized directional dissimilarity, with smaller values indicating more similar embeddings. The UMAP map is colored by molecular weight (MW) to facilitate interpretation. In the randomly initialized model (Figure 2a), molecules with different MW values are fully intermixed, and the Spearman correlation coefficient (r) between UMAP_y and MW is weak with r of −0.154. However, embeddings from the pre-trained encoder exhibits a markedly stronger MW gradient (Figure 2b), with r increasing to −0.450. The mean MW difference among the 15 nearest neighbors in the UMAP space decreased from 398.9 in random initialization to 174.2 after pre-training. Although MW is not provided as an explicit input feature, the pre-trained encoder implicitly captures MW-related structural information, indicating that contrastive pre-training organizes molecules into a more structured and chemically coherent representation space.

To further assess chemical interpretability, we compared the learned embeddings with conventional molecular fingerprints, including extended-connectivity fingerprints (ECFPs, radius = 2, 2048-bits) and RDKit topological fingerprints (RDKFP, 2048-bits). Given a query molecule *q*, we extracted the embedding *z_q_* using the graph encoder and computed cosine distances *d_cos_* (*z_q_, z_k_*) to other molecules *k* in the pre-training database. All molecules are ranked by this distance and uniformly divided into 20 bins according to ranking percentiles, where earlier bins correspond to molecules that are closer to the query in the embedding space. Within each bin, fingerprint similarity is quantified between *q* and candidate molecules *i* using the Dice coefficient,

(2) $$S_{Dice}(q,i)=\frac{2|q\cap k|}{|q|+|k|},$$

where *q* and *k* respectively denote the corresponding fingerprint bit sets. Taking the query molecule (PubChem CID: 3013908) as an example, Figure 2c presents the mean and standard deviation of ECFP and RDKFP Dice similarities as a function of the ranking percentile threshold for it. Because RDKFP is relatively coarse-grained and emphasizes molecular scaffolds, while ECFP encodes richer local substructures and functional-group patterns, RDKFP typically yields higher Dice similarities than ECFP. Importantly, as the embedding-space distance increases, both ECFP and RDKFP similarities show an overall decreasing trend, supporting a monotonic relationship between embedding distance and conventional structural similarity. In the final bin, which is the farthest 5% by embedding distance, the mean ECFP similarity drops to 0.15.

Figure 2d shows the similarity distribution fraction of the query molecule with all other molecules in the full pre-training database. Molecules with high ECFP and RDKFP Dice similarity (Dice > 0.8) to the query molecule are observed, but they represent a small fraction in the database. We additionally visualize the top-9 nearest neighbors of the query molecule in the embedding space and annotate their ECFP and RDKFP Dice similarities in Figure 2e. It illustrates that molecules closer to the query in the embedding space tend to have higher fingerprint similarity, with ECFP Dice values ranging from 0.545 to 0.909 and RDKFP Dice values ranging from 0.663 to 0.986 among the displayed neighbors. The most similar neighbor of fingerprints differs from the query mainly by replacing two ethyl substituents in a quaternary ammonium group with two n-butyl substituents, and this molecule is also the closest one to the query in the embedding space. Moreover, all top-9 neighbors share amino and ester functionalities. These results demonstrate that the contrastive pre-training on large-scale unlabeled data enables the encoder to learn chemically meaningful representations and to discriminate compounds in a structurally consistent manner.

### 2.2. Performance of Downstream Prediction Model

#### 2.2.1. Single-Task Model

Firstly, we constructed two single-task DL models of GCN and GAT by fine-tuning the pre-trained model on the small Rhodamine dataset. Meanwhile, in order to compare them with the classical ML models, four ML algorithms of Support Vector Machine (SVM), *k*-Nearest Neighbors (KNNs), Random Forest (RF), and eXtreme Gradient Boosting (XGBoost) were selected for the individual *λ*_abs_ or *λ*_emi_ predictions of rhodamines. For the ML baseline models, molecules were additionally encoded as ECFP (radius = 2, 2048-bits) vectors and were then concatenated with the seven-dimensional solvent descriptor vectors to form the final-input feature vectors. Training details of ML models can be seen in Supplementary Methods (Section SA).

**Figure 3:**
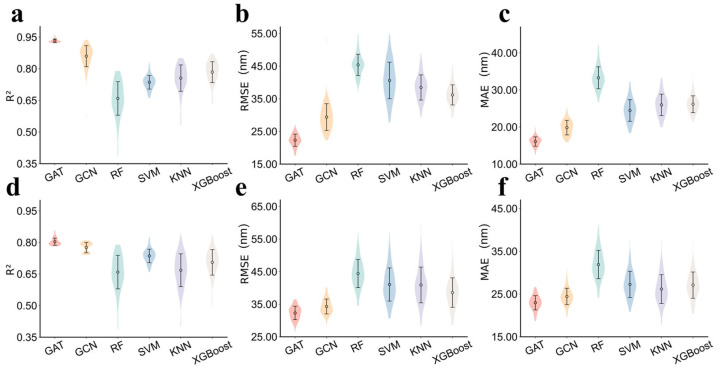
Performance of the single-task prediction models. (**a**–**c**): The average R^2^, root mean square error (RMSE), and mean absolute error (MAE) values of 100 test sets for *λ*_abs_ prediction. (**d**–**f**): The average R^2^, RMSE, and MAE values of 100 test sets for *λ*_emi_ prediction.

Comparisons among these single-task models are displayed in Figure 3 and the detailed prediction results are shown in Supplementary Table S1. It reveals that either for *λ*_abs_ or *λ*_emi_ prediction, both GAT and GCN obviously outperforms the ML models. Moreover, GAT yields more superior performance than GCN. For *λ*_abs_ prediction, the GCN model achieves a square determination coefficient (R^2^) of 0.860, while the attention-augmented GAT further increased R^2^ to 0.932. But for *λ*_emi_, the GNN models exhibit significantly reduced accuracy with R^2^ of 0.743 for GCN and 0.804 for GAT.

From a molecular representation standpoint, GNNs possess a clear structural advantage over conventional ML models. ML methods rely on fixed-length fingerprints concatenated with a limited set of solvent descriptors [26]. Although such representations capture substructural motifs and global physicochemical properties, they often fail to encode fine-grained topological connectivity, atom-level heterogeneity, and bond-type information [12,27]. In essence, they treat molecules as flat strings or vectors, where relationships between distant atoms and delocalized π-systems are only implicitly encoded through fragment enumeration [27,28]. This information bottleneck limits their ability to generalize to unseen chemical scaffolds, particularly for tasks such as *λ*_emi_ prediction that depend on subtle intramolecular charge transfer and solvent–solute interactions.

In contrast, GNNs represent molecules as node–edge graphs, where each atom is treated as a node enriched with its physicochemical features (e.g., atom type, hybridization, aromaticity, and charge) [14,24,29], which also lead to bond information such as conjugation and stereochemistry. Through iterative message passing, GNNs aggregate local neighborhood information to construct hierarchical molecular embeddings that naturally encode both local electronic environments and global topology [30,31,32]. This allows the network to learn chemically meaningful relationships, such as resonance delocalization pathways and substituent effects, which are essential for optical transition prediction [33]. The attention-based GAT further extends this capacity by adaptively weighting atom–bond interactions according to their learned relevance, enabling the model to capture conjugated chromophore regions or donor–acceptor substructures most critical to electronic excitation. This structural adaptivity explains why GAT outperformed both GCN and traditional ML algorithms.

In addition, the markedly distinct predictive behaviors observed between *λ*_abs_ and *λ*_emi_ can be rationalized from both physicochemical and modeling perspectives. Absorption is primarily determined by ground-state electronic structure [34,35], which remains relatively rigid and well defined across different environments. Therefore, its mapping from structure to property is smoother and easier to learn. Emission, conversely, is governed by excited-state relaxation dynamics and solvent–solute interactions [36,37,38,39], introducing non-trivial coupling between molecular geometry, solvation shell, and electronic transition energies. It can also be observed that quantum chemistry methods such as time-dependent density functional theory (TD-DFT) tend to exhibit larger errors for emission energies than those for absorption [26,40]. Consequently, *λ*_emi_ exhibits more complex and non-linear dependency on both molecular and environmental factors than *λ*_abs_, providing a physicochemical basis for the higher prediction uncertainty observed in our proposed models.

#### 2.2.2. Multitask Model

Since *λ*_abs_ and *λ*_emi_ originate from the same electronic transition cycle and describe two complementary processes, excitation and relaxation, it is physically meaningful to construct a consensual model to make *λ*_abs_ and *λ*_emi_ predictions jointly. While *λ*_abs_ primarily reflects a vertical electronic excitation from the ground state (S_0_) to the first excited singlet state (S_1_), *λ*_emi_ encodes the radiative energy gap between S_1_ at its relaxed minimum and S_0_, forming a correlated yet non-identical mapping from structure-to-spectral response [34,35]. Exploiting the intrinsic relationships through multitask learning enables knowledge transfer between absorption and emission prediction tasks, potentially stabilizing learning dynamics and improving prediction robustness under data-limited conditions.

Based on the mechanistic connection between absorption and emission, we a designed multitask GNN framework to jointly predict *λ*_abs_ and *λ*_emi_. In the proposed architecture, a sharing pre-trained GNN encoder (GCN or GAT) was used to learn a common molecular–solvent representation that encapsulates both topological and environmental features. The shared encoder propagated messages among atoms, capturing delocalized conjugation and substituent effects, while simultaneously integrating solvent polarity and hydrogen-bonding descriptors to model solute–solvent coupling. The encoder output was subsequently routed into two task-specific output heads, corresponding to *λ*_abs_ and *λ*_emi_ prediction, respectively. Each head consisted of a feed-forward network designed to map the latent embedding into its corresponding optical property domain, thereby allowing the shared representation to generalize across related tasks while maintaining task-specific flexibility.

The training objective was formulated as a weighted joint loss function:

(3) $${Loss}_{total}=\frac{1}{2}({Loss}_{abs}+{Loss}_{emi}),$$

where equal weighting was adopted because both *λ*_abs_ and *λ*_emi_ represent equally important and physically coupled transitions within the same photophysical cycle. From a mechanistic perspective, absorption and emission are complementary processes: excitation from the S_0_ to S_1_ state and relaxation from S_1_ back to S_0_. Assigning equal contribution in the objective ensures that the shared encoder learns a symmetrical latent representation capable of capturing both ground and excited-state determinants without introducing bias toward either excitation or relaxation dynamics.

**Figure 4:**
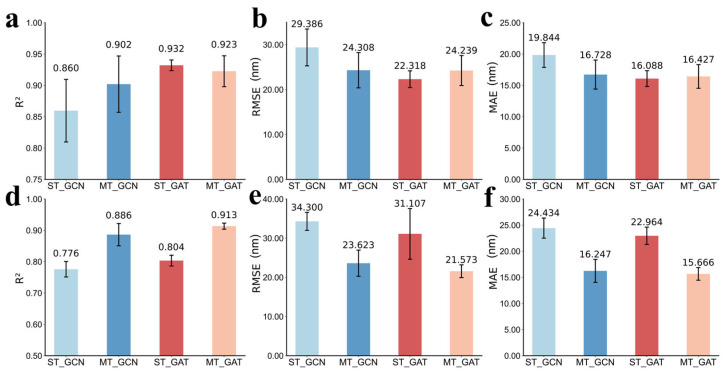
Performance comparison between single-task (ST) and multitask (MT) models. (**a**–**c**) The average R^2^, RMSE and MAE of GCN and GAT models for *λ*_abs_ prediction on the 100 different test sets. (**d**–**f**) The average R^2^, RMSE and MAE for *λ*_emi_ prediction.

As shown in Figure 4, compared to the corresponding single-task GNNs (R^2^ of 0.860 and 0.743 for GCN, 0.932 and 0.804 for GAT in *λ*_abs_ and *λ*_emi_ prediction, respectively), both GCN and GAT benefit from the multitask framework across the two prediction tasks, especially exhibiting significant improvements observed for *λ*_emi_ prediction. The multitask GCN models yield a satisfactory performance with R^2^ of 0.902 and 0.886 for *λ*_abs_ and *λ*_emi_, respectively, while the multitask GAT model reveals 0.923 for *λ*_abs_ and 0.913 for *λ*_emi_. Notably, the multitask GAT model exhibits a slight reduction (0.009) in R^2^ for *λ*_abs_ compared to its single-task counterpart (Figure 4a), but it still maintains a high R^2^ of 0.923 and achieves the highest R^2^ of 0.913 for *λ*_emi_. The performance of the multitask models is shown in Supplementary Table S1.

These findings highlight that training *λ*_abs_ and *λ*_emi_ tasks jointly by a shared encoder allows the model to transfer excitation-related structural knowledge to refine its understanding of emission processes, followed by improving the accuracy of *λ*_emi_ prediction. Meanwhile, the attention mechanism in GAT further amplifies this effect by allowing the network to dynamically re-weight atom–bond interactions according to their importance across both tasks. As a result, the multitask GAT model balances two tasks effectively, delivering substantial gains in *λ*_emi_ with only a minor decrease in *λ*_abs_.

#### 2.2.3. Ablation Experiments

To further quantify the contribution of contrastive learning-based pre-training to the prediction performance, we conducted an ablation study by training all models without pre-training stage. Four configurations were evaluated under identical training and validation protocols, include single-task GCN and GAT models (ST_GCN and ST_GAT) for *λ*_abs_ and *λ*_emi_ prediction, and their corresponding multitask variants (MT_GCN and MT_GAT). This comparison isolates the effect of pre-training from the architectural contribution of multitask learning.

**Figure 5:**
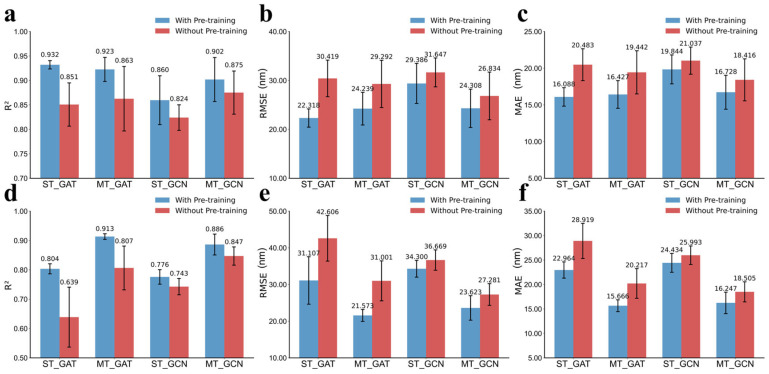
Results of ablation experiments for the model architecture, shown for single-task (ST) and multitask (MT) GCN and GAT models trained either from scratch or with contrastive pre-training under the same training and validation protocols. (**a**–**c**) R^2^, RMSE, and MAE of *λ*_abs_. (**d**–**f**) R^2^, RMSE, and MAE of *λ*_emi_.

As shown in Figure 5, in the absence of pre-training, all single-task as well as multitask models exhibit a noticeable decline in predictive accuracy. Moreover, except for the single-task model for *λ*_abs_ prediction, all other multitask models with pre-training or without pre-training reveal more superior performance than single-task models. It suggests that multitask optimization alone can partially compensate for the representational limitations of purely supervised training by coupling the excitation and relaxation learning pathways, thereby enriching the shared embedding space even without prior feature alignment. But all models achieve substantial accuracy gains by incorporating contrastive pre-training, especially the MT_GAT. So, contrastive pre-training provides a strong inductive prior, structuring the embedding space around true structure–property relationships and greatly facilitating downstream regression. Detailed results of the models without pre-training can be found in Supplementary Table S1.

The performance gains obtained by pre-training can be attributed to the representation alignment and feature discrimination effects introduced by the contrastive learning stage. During pre-training, the model learns to maximize the agreement between structurally or photophysically similar molecules while minimizing the similarity between dissimilar ones. This process shapes the latent embedding space to reflect true structure–property proximity, aligning molecular representations along photophysically meaningful dimensions such as conjugation length, donor–acceptor polarity, and solvent polarity. As a result, the pre-trained encoder provides a well-organized feature manifold that accelerates convergence and enhances generalization when fine-tuned for *λ*_abs_ and *λ*_emi_ regression.

Notably, the benefit of pre-training is complementary to multitask learning. Whereas multitask training captures cross-task consistency, contrastive pre-training enhances intra-task coherence by embedding molecules in a semantically structured latent space before supervision begins. Overall, the global representation alignment and cross-task regularization synergistically improve the model’s capacity of learning from the scare data.

#### 2.2.4. Selection of the Most Representative Prediction Model

**Figure 6:**
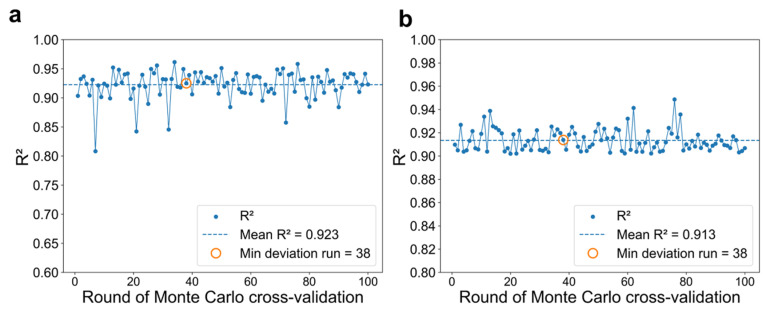
Selection of the representative MT_GAT model for external validation based on 100 Monte Carlo cross-validation rounds. (**a**,**b**) R^2^ distributions of λ_abs_ and λ_emi_, respectively.

To use a single yet representative for practical application and external validation, we then evaluated the 100 multitask GAT models (MT_GAT) constructed from 100 rounds of Monte Carlo cross-validation. The R^2^ contributions of MT_GAT for from 100 rounds are depicted in Figure 6. Detailed information has been summarized in Supplementary Table S2. We defined *d_i_* as the deviation score of the models trained based on the *ith* round:

(4) $$d_{i}=\sqrt{{(R}_{abs,i}^{2}-\bar{R_{abs}^{2}}{)}^{2}+{(R}_{emi,i}^{2}-\bar{R_{emi}^{2}}{)}^{2}},$$

where $R_{abs,i}^{2}$ and $R_{emi,i}^{2}$ denote the predictive performance of the *i*th round for the two tasks, and $\bar{R_{abs}^{2}}$ and $\bar{R_{emi}^{2}}$ are the corresponding means across 100 rounds.

The one of 100 models with the minimum deviation score was selected as most representative model for the final application test. Our results show that the model from round 38 yields the minimum deviation score of 0.00248, nearly to 0. Moreover, it achieves R^2^ values of 0.923 and 0.913 for *λ*_abs_ and *λ*_emi_, respectively. Therefore, the multitask GAT model obtained from round 38 was chosen as the representative model for following external validation.

#### 2.2.5. Performance of the Representative Model on the External Data

In order to confirm the practical feasibility of the final representative model, external validation was conducted on three held-out cohorts. In our curated *λ*_abs_ and *λ*_emi_ datasets, excluding the overlapped 390 ones from 524 and 446 entries, the remaining 134 *λ*_abs_-only values and 56 *λ*_emi_-only values that were not included in our multitask models were labeled as D_abs_ and D_emi_, respectively. In addition, in our group, we have designed and synthesized 32 rhodamines whose *λ*_abs_ and *λ*_emi_ values have also been experimentally confirmed (Supplementary Methods Section SB). Since they were not also contained for the model construction, they were used for the external validation, referred to as D_32_. Supplementary Table S3 lists the name and corresponding SMILES of each molecule. All solvents of molecules are H_2_O.

The prediction results of the final model on the three external datasets are shown in Table 1 and the full per-compound prediction tables are provided in the Supplementary Tables S4–S6. It can be seen that the final model shows strong agreement with experiments for the λ_abs_ prediction, yielding the high R^2^ of 0.862 and 0.915 on D_abs_ and D_32_, respectively. Although the prediction accuracy for λ_emi_ remains competitive but is lower than that for λ_abs_. Moreover, the predicted errors mainly concentrate in the large red-shifted emitters, which means that further gains are most likely to come from enriching emission annotations in the long-wavelength regime. Overall, these results confirm that the pre-trained multitask formulation generalizes beyond the training distribution and delivers reliable prospective estimates. Importantly, joint prediction on the paired set enables direct computation of Stokes shifts for candidate prioritization.

In addition, to further verify the superiority of our model, we also performed theoretical calculations for the 32 rhodamines in D_32_ using the quantum mechanical (QM) method. The details of QM calculations are provided in Supplementary Methods Section SC. The comparisons between QM and our MT_GAT are listed in Table 2.

In general, the MT_GAT approach exhibits more stable predictions and lower errors for both *λ*_abs_ and *λ*_emi_. The average absolute error (AE) by QM is 129 nm for *λ*_abs_ and 99 nm for *λ*_emi_, much higher than that of 19 nm for *λ*_abs_ and 23 nm for *λ*_emi_ by MT_GAT. For most samples, the AEs of λ_abs_ and λ_emi_ predicted by MT_GAT remain below 20 nm, whereas the QM errors are markedly larger for multiple molecules, with deviations reaching from 100 to 300 nm in several cases. These results indicate that the QM baseline exhibits substantially larger errors and less stable predictions than MT_GAT on our D_32_ set. Therefore, the MT_GAT method is of greater practical value for molecular screening and rapid property evaluation.

### 2.3. Interpretability Analysis

To systematically evaluate the structure–property relationships learned by the model for *λ*_abs_ and *λ*_emi_ prediction, we constructed a multilayer interpretability framework based on attention weights of the trained multitask GAT model [41]. Attention coefficients were extracted from all layers and analyzed at the atom, bond, and cross-molecule levels. In GAT, each attention coefficient *α_ij_* depends on the feature vectors of the two nodes connected by an edge and reflects the importance of neighbor *j* when updating the representation of atom *i*. Thus, edge-level attention directly indicates the relative contribution of individual chemical bonds to the final prediction, and node importance can be obtained by aggregating the attention weights associated with its incident edges.

Layer-wise attention statistics show that the proportion of high-contribution atoms (attention weight > 0.8) decreases progressively across layers with 26.49%, 13.36%, and 8.37%, respectively (Supplementary Figure S2). The first layer displays a right-skewed distribution with a mean value of approximately 0.71, indicating that the model broadly attends to local structural features such as aromatic ring frameworks and the environments around heteroatoms. As layer depth increases, the mean attention decreases to approximately 0.64 and 0.49 for the second and third layers, respectively, while the standard deviation slightly increases. This suggests a progressive refinement process: many atoms are down-weighted, while a small subset retains high importance, highlighting critical structural regions. So, it indicates that the GAT does not simply amplify attention with depth, but instead performs broad local exploration in shallow layers and selective filtering in deeper layers to emphasize structural units most relevant to spectral properties. This behavior aligns well with established photophysical understanding that optical responses are dominated by specific conjugated frameworks and donor–acceptor regions.

**Figure 7:**
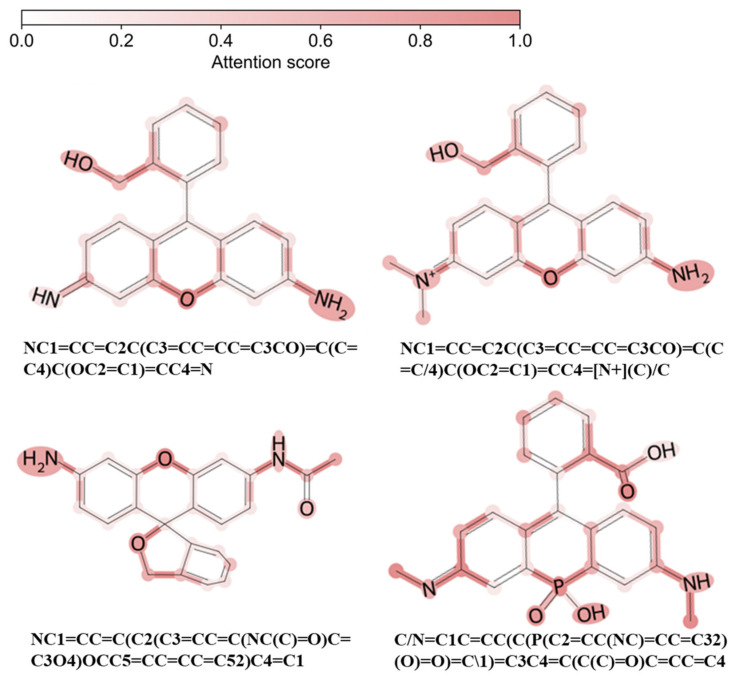
Attention-based interpretable analysis. The computed normalized attention scores determine the color gradient, with darker colors indicating higher importance assigned to atoms or edges.

We further aggregated normalized third-layer attention scores and quantified most important atoms across molecules. As shown in Figure 7, the atoms with high-attention weights are consistently enriched around heteroatoms in the xanthene core and carbon atoms near the bridging positions, with high attention also observed on electron-donating dimethylamino substituents. These patterns are consistent with classical photophysical principles: the maximum absorption wavelength of rhodamine dyes is primarily determined by the π–π* electronic excitation localized on the xanthene π-conjugated scaffold and xanthene conjugated backbone [42]; structural modifications that extend effective π-conjugation generally reduce the excitation energy gap and lead to bathochromic shifts in the absorption maxima [43]. In addition, the amino substituents on xanthene-type rhodamines act as electron-donating groups, which can modulate spectral positions [43].

Overall, the attention-based interpretability analysis demonstrates that the multitask GAT model relies on structural determinants that agree closely with known spectroscopic and photophysical mechanisms. The automatically identified high-attention regions correspond to established chromophore centers, electron-donating/withdrawing substituents, and intramolecular charge-transfer pathways. Task-specific differences in attention distribution reflect the distinct sensitivities of absorption and emission processes to electronic structure and solvent effects.

### 2.4. Designing New Molecules

The Stokes shift is closely related to *λ*_abs_ and *λ*_emi_. Near-infrared fluorophores with large Stokes shifts are desirable for high signal-to-noise biological imaging. Current strategies of increasing Stokes shift in rhodamine dyes typically involve modifications at the 9-position, but these changes often cause undesirable blue-shifts in emission [44]. Alternatively, several studies have demonstrated that replacing the oxygen atom in the rhodamine xanthene core with a carbon atom can effectively red-shift the emission wavelength, providing an additional route to tuning Stokes shifts in rhodamine-based fluorophores [45]. Guided by the attention analysis on our MT_GAT model, which highlights strong importance around xanthene heteroatoms and the 3′ and 6′ amino positions, and motivated by the objective of enlarging the Stokes shift, we designed a series of new rhodamine derivatives by introducing carbon substitution at the heteroatom site and modifying the bridging amino groups. Three compounds, including P-C-ARh, I-C-Arh, and Q-C-Arh, show the most promising Stokes shifts by our MT_GAT, as listed in Table 3. Three molecules give the predicted Stokes shifts of 153 nm, 174 nm, and 157 nm, respectively, substantially larger than the typical < 30 nm of conventional rhodamines [46], indicating that all three compounds represent feasible candidates for synthesis and experimental validation.

Then, experimental measurements confirmed the Stokes shifts of 135 nm, 164 nm, and 148 nm, respectively, with minimum AEs between predicted and experimental Stokes shifts of only 9 nm, demonstrating the strong reliability of the proposed model. We also give the results of these three compounds with QM calculations, also indicating that our model is superior to QM. These results validate that the proposed model can not only accurately predict spectral properties but also guide the rational design of new molecules, with potential applications in near-infrared imaging probes, high-performance fluorescent materials, and controllable electronic structure engineering.

## 3. Materials and Methods

### 3.1. Dataset Curation, Preprocessing and Analysis

To support small-sample regression for low-data rhodamines, we first assembled the downstream labeled dataset from public literature and patents, as provided in Supplementary Methods (Section SB). Given that the ring-opening/closing of rhodamines is affected by solvents and pH, we selected the predominantly “major” structure under the originally reported measurement conditions as the molecular input. If multiple peaks are reported for the same compound in the same solvent, the peak with the highest intensity was retained as the *λ*_abs_/*λ*_emi_ value. Buffers such as PBS were recorded as H_2_O to ensure consistency in solvent annotation.

Because rhodamine derivatives are 3′,6′-diamino xanthene fluorophores, we aligned the pre-training chemical space and mitigated distribution shift by querying PubChem (https://pubchem.ncbi.nlm.nih.gov/) [47] with xanthene via keywords, SMILES, and structure searches, yielding 122,532 unlabeled candidates for the pre-training pool.

A unified processing pipeline was applied to both the unlabeled xanthene dataset and the labeled rhodamine dataset:

1. Structure files were converted to canonical SMILES representations using RDKit (https://rdkit.org/docs/index.html, accessed on 17 October 2023, version 2023.03.3);
2. Metal-containing compounds were removed;
3. Salt forms were divided into neutral parent molecules and corresponding counterions, followed by charge neutralization;
4. Tautomeric forms were standardized;
5. Duplicate molecules were removed based on canonical SMILES.

Thus, the labeled rhodamine dataset comprises 524 λ_abs_ entries corresponding to 421 unique molecules (Supplementary Table S7) and 446 λ_emi_ entries corresponding to 347 unique ones (Supplementary Table S8) across 17 different solvents. For example, a compound reported as RDM1 [48] exhibits different λ_abs_ wavelengths in six distinct solvents, so it was recorded as six separate λ_abs_ entries.

To prevent information leakage of downstream tasks, the unlabeled xanthene set was de-duplicated against the rhodamine labeled set, so it remained 48,148 xanthenes for contrastive pre-training.

We analyzed the distributions of *λ*_abs_ and *λ*_emi_ wavelengths based on the collected experimental records (Supplementary Figure S3). From Figure S3a,d, we can see that both *λ*_abs_ and *λ*_emi_ distributions show substantial overlap in the visible and near-infrared regions, supporting the physical relevance of joint modeling. To characterize the structural heterogeneity of labeled rhodamines, the pairwise Tanimoto coefficients (Tc) were computed based on ECFPs (radius =2, 2048-bit) and RDKFPs (2048-bit). As shown in Figure S3b,c,e,f, the ECFP-based Tc values exhibit a left skewed distribution, with the median Tc in both datasets under 0.15 and approximately 95% of molecular pairs remaining below 0.45. The RDKFP-based similarities show that approximately 85% of molecular pairs have Tc values below 0.7. These findings indicate the abundant chemical structure diversity of the compounds in our datasets.

Among all labeled entries, an overlapped subset was extracted with 390 records (Supplementary Table S9), for which both *λ*_abs_ and *λ*_emi_ measurements are available under the matched solvent conditions. It forms the basis of the multitask learning framework and ensures that absorption and emission predictions are learned from physically consistent excitation–relaxation pairs. In addition, the ultra-low labeled data motivate the use of contrastive pre-training to stabilize representation learning under data scarcity.

### 3.2. Feature Engineering

#### 3.2.1. Molecular Graph Representation

Each chemical molecule is encoded from its SMILES representation as a graph:

(5) G=(V,E),

where *V* is the set of atoms and *E* is the set of covalent bonds. For each atom v∈V,

(6) $$x_{v}\in R^{d_{\mathrm{atom}}},$$

An atom feature vector comprises eight atom-level descriptors that are closely coupled to physicochemical changes underlying fluorescence absorption and emission, including atomic ordinal number, degree, valence, total number of H atoms, hybridization, aromaticity, solvent-accessible surface area (SASA), and partial charge. Detailed RDKit functions to compute these descriptors are summarized in Supplementary Table S10.

All node features are stacked into a matrix **X**:

(7) $$X=[x_{1}^{⊤}\ldots x_{n}^{⊤}{]}^{T}\in R^{n\times d_{\mathrm{atom}}},$$

The molecular graph structure is encoded by an adjacency matrix $A\in {\left\{0,1\right\}}^{n\times n}$, where $A_{ij}=1$ if atoms *i* and *j* are connected by a bond. Therefore, a molecule with *n* atoms is represented by a node feature matrix $X\in R^{8\times n_{\mathrm{atom}}}$ together with an adjacency matrix $A\in {\left\{0,1\right\}}^{n\times n}$.

#### 3.2.2. Solvent Representation

Each solvent is represented by a feature vector **z***_s_*:

(8) $$z_{s}\in R^{d_{\mathrm{sol}}},$$

It comprises 7 physicochemical descriptors to explicitly encode how solvent polarity, polarizability, and hydrogen-bonding capacity influence the photophysical processes of conjugated fluorophores, including dielectric constant, refractive index, octanol–water partition coefficient (log P), dipole moment, density, and hydrogen-bond donor and acceptor descriptors.

### 3.3. Pre-Training Framework Design and Prediction Model Construction

**Figure 8:**
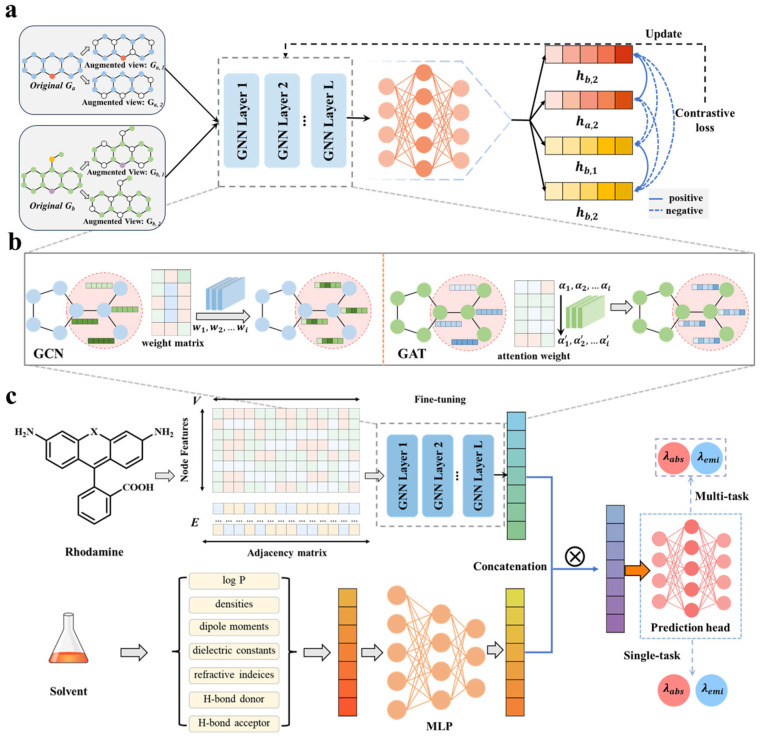
Illustration of the two-stage learning framework for predicting *λ*_abs_ and *λ*_emi_ of rhodamine derivatives. (**a**) Self-supervised contrastive pre-training on a large corpus of xanthene-derived molecules. (**b**) Message passing and node updates in the graph encoder (GCN/GAT). (**c**) Fine-tuning for downstream task. Molecular and solvent representations are combined and passed to task-specific regression heads for *λ*_abs_ and *λ*_emi_ prediction. The two tasks share the same encoder and solvent-mapping module, differing only in the output layers.

Traditional GNNs typically require high-throughput and high-quality datasets to achieve stable optimization [33]. Considering that experimentally reported *λ*_abs_ and *λ*_emi_ values for rhodamine derivatives are extremely limited in size and exhibit substantial heterogeneity, directly training a deep graph model from scratch on such a small-size dataset can lead to unstable convergence and poor generalization. To address this challenge, we adopted a pre-training strategy based on contrastive learning using a large corpus of xanthene-derived molecules. Here, the two-stage learning framework were proposed for predicting *λ*_abs_ and *λ*_emi_ of rhodamine derivatives, as shown in Figure 8.

Firstly, contrastive learning provides an unlabeled representation learning paradigm in which molecules sharing similar structural patterns are encouraged to occupy nearby regions of the latent space, whereas structurally dissimilar molecules are pushed apart [49]. In practice, two chemically conservative node-level graph augmentations are generated for each molecule within a batch, and the encoder is optimized using the NT-Xent loss to maximize agreement between augmentations of the same molecule while minimizing similarity across different molecules (Figure 8a) [24,49]. Through this procedure, the pre-trained model is encouraged to encode scaffold-level chemical knowledge and to learn representations that are sensitive to variations in conjugation, substitution patterns and π-electron delocalization, which are well-recognized determinants of the photophysical behavior of rhodamine fluorophores [7,20,24,50].

Then, the pre-trained model is then fine-tuned on the curated rhodamine dataset using supervised regression objectives for predicting *λ*_abs_ and *λ*_emi_. Graph encoders (GCN and GAT) are shared at the pre-training stage (Figure 8b), mapping atom and bond-level information into molecular embeddings that capture both local electronic environments and global conjugation topology. Solvent descriptors are encoded into a solvent-level feature vector and incorporated through a lightweight fusion network composed of multilayer perceptrons (MLPs). The resulting solvent embedding is concatenated with the molecular graph representation, enabling the model to modulate its predictions based on solvent polarity, hydrogen-bonding capacity, and refractive properties. The combined solute–solvent representation is subsequently passed to separate output heads specialized for *λ*_abs_ or *λ*_emi_ prediction. Figure 8c shows the overall downstream architecture, consisting of a graph encoder, a solvent-fusion module, and task-specific regression heads.

In the multitask GNNs, the two prediction tasks share the same graph encoder and solvent-mapping module, differing only in their task-specific output layers. This design facilitates the learning of shared and task-specific representations, reflecting the correlated yet distinct factors underlying absorption and emission processes.

The two-stage learning framework confers several advantages for downstream prediction. First, contrastive pre-training provides a strong structural prior that stabilizes supervised learning under data scarcity and reduces the risk of overfitting. Second, graph-based message passing enables the encoder to learn chemically meaningful interactions, such as resonance pathways, donor–acceptor coupling, and solvent-induced perturbations, which are difficult to model with fingerprint-based approaches. Third, the shared encoder in the multitask configuration facilitates information transfer between absorption and emission predictions, enhancing ability of the model to represent the underlying electronic transition cycle. Collectively, this design establishes a coherent and data-efficient modeling pipeline that aligns closely with the physical chemistry of rhodamine fluorophores and directly supports the performance improvements observed in Section 2.

#### 3.3.1. Graph Convolutional Network

As a simple and general graph encoder, GCN can efficiently aggregates local neighborhood information. Considering a K-layer graph convolutional network, $H^{\left(0\right)}=X$ denotes the initial node feature matrix. We add self-loops to the graph, $\tilde{A}=A+I$, and define the corresponding degree matrix $\tilde{D}$ with ${\tilde{D}}_{ii}=\sum_{j}{\tilde{A}}_{ij}$.

At layer *l*, the GCN update is given by

(9) $$H^{\left(l+1\right)}=\sigma \;({\tilde{D}}^{-\frac{1}{2}}\tilde{A}{\tilde{D}}^{-\frac{1}{2}}H^{\left(l\right)}W^{\left(l\right)}),$$

where $W^{\left(l\right)}\in R^{d_{l}\times d_{l+1}}$ is a learnable weight matrix, *σ*(·) is a pointwise nonlinearity (e.g., ReLU), and $H^{\left(l\right)}\in R^{n\times d_{l}}$ collects the node embeddings at layer *l*.

This operation can be interpreted as a normalized message passing scheme: each node aggregates a weighted average of transformed neighbor features, which captures local electronic environments, conjugation continuity, and aromatic patterns relevant for the ground-state electronic structure.

After K layers of propagation, we obtain node embeddings $H^{\left(K\right)}=[h_{1},\ldots ,h_{n}{]}^{⊤}$. A permutation-invariant READOUT operator produces a graph-level representation:

(10) $$h_{G}^{\mathrm{mol}}=\mathrm{READOUT}(\{h_{v}|v\in V\})\in R^{d_{\mathrm{mol}}},$$

Solvent information is then fused with the molecular embedding via concatenation followed by a linear projection:

(11) $$h_{G}=\phi \;([\begin{array}{c} h_{G}^{\mathrm{mol}}\\ z_{s} \end{array}])\in R^{d},$$

where *ϕ* is a small feed-forward network.

#### 3.3.2. Graph Attention Network

To allow the model to assign different importance to different neighbors, we further adopt a Graph Attention Network. Starting from node features H(l), GAT first applies a shared linear projection:

(12) $$H^{\prime (l)}=H^{\left(l\right)}W^{\left(l\right)},W^{\left(l\right)}\in R^{d_{l}\times d_{l}^{\prime }},$$

For each edge (i,j)∈E, the attention score is computed as

(13) $$e_{ij}^{\left(l\right)}=a\;(h_{i}^{\prime (l)},h_{j}^{\prime (l)}),$$

where a (·,·) is typically implemented as

(14) $$e_{ij}^{\left(l\right)}=\mathrm{LeakyReLU}(a^{⊤}[h_{i}^{\prime (l)}\;∥\;h_{j}^{\prime (l)}]),$$

with $a\in R^{2d_{l}^{\prime }}$ a learnable vector and ∥ denoting concatenation.

Attention coefficients are obtained through a softmax over the neighborhood N(i):

(15) $$\alpha_{ij}^{\left(l\right)}=\frac{exp(e_{ij}^{\left(l\right)})}{\sum_{k\in N(i)}exp(e_{ik}^{\left(l\right)})},$$

The node update is then

(16) $$h_{i}^{\left(l+1\right)}=\sigma (\sum_{j\in N(i)}\alpha_{ij}^{\left(l\right)}h_{j}^{\prime (l)}),$$

In the multi-head setting, *M* independent attention heads are used and their outputs are concatenated or averaged:

(17) $$h_{i}^{\left(l+1\right)}={∥}_{m=1}^{M}\sigma (\sum_{j\in N(i)}\alpha_{ij}^{\left(l,m\right)}h_{j}^{\prime (l,m)}),$$

This mechanism allows the model to learn which neighbors are most relevant for the optical transition, rather than uniformly aggregating all neighbors as in GCN. Graph-level pooling and solvent fusion are performed as above to obtain **h***_G_*.

#### 3.3.3. Contrastive Learning Framework

To exploit a large set of unlabeled xanthene molecules, we first pre-trained the graph encoder using contrastive learning. For each molecule *G_i_* in the unlabeled set from the same batch, two stochastic graph augmentations *t*_1_(*G_i_*) and *t*_2_(*G_i_*) were generated by node mask, preserving its chemical identity. Passing them through the encoder yields following representations:

(18) $$h_{i}^{\left(1\right)}=f_{\theta }\left(t_{1}\left(G_{i}\right)\right),h_{i}^{\left(2\right)}=f_{\theta }\left(t_{2}\left(G_{i}\right)\right),$$

which are then normalized to unit length for cosine similarity.

A batch containing B molecules gives 2B views. For a given positive pair $\left(h_{i}^{\left(1\right)},h_{i}^{\left(2\right)}\right)$ and a given negative pair ($h_{i},h_{j}$), it is defined as NT-Xent loss [24]:

(19) $$L_{ij}=-log\frac{exp(\mathrm{sim}(h_{i}^{\left(1\right)},h_{i}^{\left(2\right)})/\tau )}{\sum_{k=1}^{2B}\left\{i\neq j\right\}exp(\mathrm{sim}(h_{i},h_{j})/\tau },$$

where *τ* > 0 is temperature parameter.

Minimizing *L_ij_* forces different augmented views of the same molecule to be mapped close together in the embedding space, while pushing apart embeddings of different molecules. This encourages the encoder *f_θ_* to organize the latent space along chemically meaningful dimensions (e.g., scaffold type, conjugation length, and substitution pattern) without using optical labels. Encoder *f_θ_* was optimized on the unlabeled pre-training set with Lij. The details of pre-training process are provided in Supplementary Methods (Section SD).

### 3.4. Model Training and Evaluation Metrics

The GNN model was implemented using the PyTorch 2.6.0 deep learning framework (Python 3.9.13) and trained on an NVIDIA GeForce RTX 2080 Ti GPU [51]. Hyperparameters of DL models were optimized using Optuna [52]. Optimized hyperparameters for pre-trained models, ML models, and fine-tuning models are provided in Supplementary Tables S10–S12. Here, the labeled data used for single-task and multitask learning were randomly split into training and test sets with an 8:2 ratio.

Generally, 100 different Monte Carlo splits are repeated to avoid the prediction bias of a single split. The R^2^, MAE, and RMSE were used as statistic metrics, as calculated by Equations (20)–(22):

(20) $$R^{2}=1-\frac{\sum_{i=1}^{n}{\left(y_{i}-\hat{y_{i}}\right)}^{2}}{\sum_{i=1}^{n}{\left(y_{i}-\bar{y_{i}}\right)}^{2}},$$

(21) $$MAE=\frac{1}{n}\sum_{i=1}^{n}\left|\hat{y_{i}}-y_{i}\right|,$$

(22) $$MSE=\frac{1}{n}\sum_{i=1}^{n}{\left(y_{i}-\hat{y_{i}}\right)}^{2},$$

where *y_i_* and $\hat{y_{i}}$ represent the reported value and the predicted value of sample *i*, respectively; $\bar{y_{i}}$ is the average of *y_i_* and *n* is the number of samples.

### 3.5. Molecular Synthesis and Experimental Characterization

In order to confirm the practical feasibility of the proposed model, three novel rhodamine derivatives (P-C-ARh, I-C-ARh and Q-C-ARh) were designed and then synthesized following the reaction schemes provided in Supplementary Methods (Section SE), with the detailed synthetic routes and key intermediates summarized in Supplementary Figure S4. The chemical structures of the final compounds were verified by NMR spectroscopy, and the corresponding NMR spectra are presented in Supplementary Figure S5.

Optical properties were experimentally characterized using solution-phase spectroscopy. The UV–Visible absorption spectra of P-C-ARh, I-C-Arh, and Q-C-ARh are shown in Supplementary Figure S6, and the corresponding fluorescence emission spectra are shown in Supplementary Figure S7. From these spectra, the experimental peak positions were extracted to obtain λ_abs_ or λ_emi_, which were used for downstream validation and analysis.

## 4. Conclusions

In this work, we present a contrastive pre-trained multitask GNN framework for joint predictions of *λ*_abs_ and *λ*_emi_ on the limited rhodamine fluorescent molecules. By leveraging self-supervised contrastive learning on a large corpus of xanthene-derived structures and fine-tuning on an experimentally curated rhodamine dataset, the method achieves superior predictive performance, with R^2^ exceeding 0.90 for both *λ*_abs_ and *λ*_emi_. Our model not only outperforms classical ML baselines, but also give superior performance than theoretical calculations. Moreover, compared to the single-task models, the multitask design exploits the physical coupling between excitation and relaxation processes, enabling more robust modeling of emission behavior under data-scarce and heterogeneous experimental conditions.

Attention-based interpretability analysis demonstrates that the learned importance patterns are consistent with well-established photophysical principles, highlighting the roles of the xanthene conjugated backbone, donor–acceptor substituents, and intramolecular charge-transfer pathways. Guided by these insights, we rationally designed and experimentally validated new rhodamine derivatives with significantly enlarged predicted Stokes shifts as much as 174 nm and demonstrating a minimum prediction-experiment deviation of only 9 nm. So, the proposed framework also enables mechanism-driven molecular design.

Overall, we establish a data-efficient and mechanistically grounded paradigm for fluorescence property modeling, interpretability, and practical molecular design. The developed strategy holds broad potential for the development of near-infrared imaging probes, high-performance fluorescent materials, and tunable optoelectronic platforms.

## Figures and Tables

**Table 1:** External validation results by the MT_GAT model on three external datasets.

Dataset	Entries	R^2^ (nm)	MAE (nm)	RMSE (nm)
D_abs_	134	0.862	22.872	26.012
D_emi_	56	0.721	24.659	28.880
D_32_-*λ*_abs_	32	0.915	19.250	24.122
D_32_-*λ*_emi_	32	0.724	22.875	26.073

**Table 2:** Comparisons of experimental values (Exp.), QM calculations (Calc.), and the MT_GAT model predictions (Pred.) for optical property prediction on D_32_.

Name		Exp. (nm)	Calc. (nm)	Pred. (nm)	AE_Calc. (nm)	AE_Pred (nm)
P-Si-ARh	*λ*_abs_	465	477	460	12	5
	*λ*_emi_	622	582	596	40	26
I-Si-ARh	*λ*_abs_	464	472	472	8	8
	*λ*_emi_	655	596	638	59	17
Q-Si-ARh	*λ*_abs_	508	509	496	1	12
	*λ*_emi_	660	659	648	1	12
P-P-ARh	*λ*_abs_	499	482	505	17	6
	*λ*_emi_	630	604	622	26	8
I-P-ARh	*λ*_abs_	517	498	507	19	10
	*λ*_emi_	660	658	642	2	18
Q-P-ARh	*λ*_abs_	563	544	544	19	19
	*λ*_emi_	675	718	662	43	13
P-S-ARh	*λ*_abs_	514	508	502	6	12
	*λ*_emi_	650	618	626	32	24
N-S-ARh	*λ*_abs_	522	519	529	3	7
	*λ*_emi_	675	627	678	48	3
J-S-ARh	*λ*_abs_	542	510	539	32	3
	*λ*_emi_	705	635	682	70	23
I-S-ARh	*λ*_abs_	544	307	532	237	12
	*λ*_emi_	725	667	684	58	41
M-S-ARh	*λ*_abs_	505	495	498	10	7
	*λ*_emi_	638	587	631	51	7
FEN	*λ*_abs_	566	327	555	239	11
	*λ*_emi_	710	435	686	275	24
FMN	*λ*_abs_	556	322	544	234	12
	*λ*_emi_	698	434	681	264	17
FAN	*λ*_abs_	552	320	543	232	9
	*λ*_emi_	709	434	684	275	25
FPN	*λ*_abs_	552	320	543	232	9
	*λ*_emi_	709	434	684	275	25
Si-Ph-NH_2_	*λ*_abs_	662	561	637	101	25
	*λ*_emi_	700	693	663	7	37
Si-Ph-Ac	*λ*_abs_	548	562	522	14	26
	*λ*_emi_	690	731	668	41	22
Si-2OMe-NH_2_	*λ*_abs_	665	565	637	100	28
	*λ*_emi_	703	700	669	3	34
Si-2OMe-Ac	*λ*_abs_	550	563	520	13	30
	*λ*_emi_	700	728	670	28	30
Si-2,6OMe-NH_2_	*λ*_abs_	666	564	627	102	39
	*λ*_emi_	707	688	666	19	41
Si-2,6OMe-Ac	*λ*_abs_	552	566	530	14	22
	*λ*_emi_	703	731	676	28	27
SiR-pH	*λ*_abs_	662	353	643	309	19
	*λ*_emi_	700	438	674	262	26
SiR-IapH	*λ*_abs_	624	310	617	314	7
	*λ*_emi_	644	316	633	328	11
DMBA	*λ*_abs_	728	609	676	119	52
	*λ*_emi_	765	720	721	45	44
CN	*λ*_abs_	550	362	531	188	19
	*λ*_emi_	650	653	641	3	9
IDMN	*λ*_abs_	800	635	744	165	56
	*λ*_emi_	860	844	799	16	61
ID	*λ*_abs_	685	604	680	81	5
	*λ*_emi_	742	700	709	42	33
O-4Py	*λ*_abs_	560	352	552	208	8
	*λ*_emi_	594	456	583	138	11
C-4Py	*λ*_abs_	630	377	598	253	32
	*λ*_emi_	650	488	639	162	11
Si-4Py	*λ*_abs_	664	378	647	286	17
	*λ*_emi_	691	518	671	173	20
P-4Py	*λ*_abs_	712	399	671	313	41
	*λ*_emi_	745	561	719	184	26
SO-4Py	*λ*_abs_	718	471	669	247	49
	*λ*_emi_	755	582	742	173	13
MAE (nm)	Calc.	*λ*_abs_	129	Pred.	*λ*_abs_	19
		*λ*_emi_	99		*λ*_emi_	23

**Table 3:** The detailed optical information of the three designed molecules by our model and experimental detection.

Compound	Method	*λ*_abs_ (nm)	*λ*_emi_ (nm)	Stokes Shifts (nm)	AE (nm)
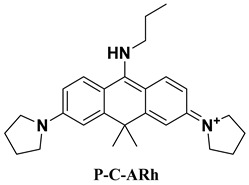	Prediction	454	607	153	18
	Experiment	465	600	135	\
	Calculation	449	566	117	18
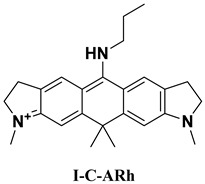	Prediction	470	644	174	10
	Experiment	469	633	164	\
	Calculation	454	574	120	54
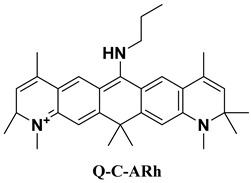	Prediction	500	657	157	9
	Experiment	504	652	148	\
	Calculation	481	644	163	15

## Data Availability

The labeled dataset and external validation set were curated from the published literature and are provided in Supporting Information, while the unlabeled dataset was retrieved from the PubChem database (keywords: “xanthene”). All datasets and Python scripts are publicly available in the GitHub repository: https://github.com/jgqiu/Rhodamine-model, accessed on updated on 23 January 2026.

## Supplementary Materials

The following supporting information can be downloaded at https://www.mdpi.com/article/10.3390/molecules31071149/s1. Figure S1. Loss curves of pre-trained models. (a) GCN and (b) GAT. Figure S2. Attention importance distribution. Figure S3. Distribution and fingerprint similarity analysis of absorption and emission data. (a–c) are for *λ*_abs_ dataset and (d–f) for *λ*_emi_ dataset. Figure S4. Detailed synthetic routes of **P-C-ARh**, **I-C-ARh**, and **Q-C-ARh**. Figure S5. NMR spectra of **P-C-ARh**, **I-C-ARh**, and **Q-C-ARh**. Figure S6. (a), (c), and (e) show the UV–visible absorption spectra of probe molecules **P-C-ARh**, **I-C-ARh**, and **Q-C-ARh**, respectively, at concentrations of 1, 2, 4, 6, 8, and 10 μM in PBS buffer (pH 7.4); (b), (d), and (f) show the corresponding linear fitting plots of absorbance versus concentration at their respective maximum absorption wavelengths. Figure S7. (a), (b), and (c) show the normalized fluorescence emission spectra of probe molecules **P-C-ARh**, **I-C-ARh**, and **Q-C-ARh** in dichloromethane, methanol, dimethyl sulfoxide, and PBS buffer, respectively. The concentration of the probe molecules in all tested systems was 10 μM. Table S1. Detailed results of ML models, single-task (ST) DL models, multitask (MT) DL models, and DL models without pre-training (WoP) on training and test set. The results are displayed as mean ± standard deviation over 100 Monte Carlo cross-validation rounds. Table S2. The R^2^ contributions of MT_GAT for from 100 rounds. Table S3. Molecular names in the D_32_ dataset and their corresponding SMILES. Table S4. Results of per-compound prediction in external validation of D_abs_ set. Table S5. Results of per-compound prediction in external validation of D_emi_ set. Table S6. Results of per-compound prediction in external validation of D_32_ set, with all solvents of molecules are H_2_O. Table S7. The labeled λ_abs_ of rhodamine derivatives. Table S8. The labeled λ_emi_ of rhodamine derivatives. Table S9. The overlapped subset of rhodamine derivatives. Table S10. Atom-level descriptors used for molecular graph representation. Table S11. Optimized hyperparameters for pre-trained model. Table S12. Optimized hyperparameters for machine learning baseline models. Table S13. Details of fine-tuning models. Supplementary Methods: Section SA: Construction of machine learning models; Section SB: Collection of rhodamine optical data; Section SC: Quantum mechanics (QM) calculation; Section SD: Pre-training Process; Section SE: Synthesis of designed molecules. Refenerces [53,54,55,56,57,58,59,60,61,62,63,64,65,66,67,68,69,70,71,72,73,74,75,76,77,78,79,80,81,82,83,84,85,86,87] are cited in the Supplementary Materials.

## Abbreviations

The following abbreviations are used in this manuscript:

*λ*_abs_	Maximum absorption wavelengths
*λ*_emi_	Maximum emission wavelengths
QM	Quantum mechanical
ML	Machine Learning
DL	Deep Learning
GNN	Graph Neural Network
UMAP	Uniform Manifold Approximation and Projection
ECFP	extended-connectivity fingerprint
RDKFP	RDKit topological fingerprint
GCN	Graph Convolutional Network
GAT	Graph Attention Network
MW	Molecular Weight
SVM	Support Vector Machine
KNN	*k*-Nearest Neighbor
RF	Random Forest
XGBoost	eXtreme Gradient Boosting
R^2^	square determination coefficient
RMSE	Root Mean Squared Error
MAE	Mean Absolute Error
S_0_	The ground state
S_1_	The first excited singlet state
ST_GCN	Single-Task GCN models
ST_GAT	Single-Task GAT models
MT_GCN	Multi Task GCN models
MT_GAT	Multi Task GAT models
Exp.	Experimental value
Calc.	Calculation
Pred.	Prediction
SMILES	Simplified Molecular Input Line Entry System
Tc	Tanimoto coefficients
MLP	Multilayer Perceptron
