# Supplementary material for: A Contrastive-Learning-Based Pre-Training Framework for Optical Property Prediction of Low-Data Rhodamines with Interpretable Multitask Graph Neural Networks
Source: Molecules. 2026 Mar 31;31(7):1149. doi: 10.3390/molecules31071149 (PMC13075052; doi:10.3390/molecules31071149)
Supplement: Supplementary file 1 [file molecules-31-01149-s001.zip › molecules-4208730-supplementary.pdf]

Supplementary Materials

A Contrastive-Learning-Based Pre-Training Framework for Optical Property Prediction of Low-Data Rhodamines with Interpretable Multitask Graph Neural Networks

Jiangguo Qiu [1], Yanling Wu [1], Hong Zhang [2], Menglong Li [1], Xuemei Pu [1] and Yanzhi Guo [1,*]

[1] College of Chemistry, Sichuan University, Chengdu 610064, China; qiu_jiangguo@stu.scu.edu.cn (J.Q.); 2024322030088@stu.scu.edu.cn (Y.W.); liml@scu.edu.cn (M.L.); xmpuscu@scu.edu.cn (X.P.)

[2] Functional and Molecular Imaging Key Laboratory of Sichuan Province, Department of Radiology and Huaxi MR Research Center (HMRRC), West China Hospital, Sichuan University, Chengdu 610041, China; zhanghong94@scu.edu.cn

* Correspondence: yzguo@scu.edu.cn

Table of Contents

Supplementary Figures 3
Figure S1 3
Figure S2 3
Figure S3. 4
Figure S4. 5
Figure S5 8
Figure S6 9
Figure S7 10
Supplementary Tables 11
Table S1 11
Table S2 12
Table S3. 13
Table S4 14
Table S5. 21
Table S6. 28
Table S7 30
Table S8 57
Table S9 82
Table S10 103
Table S11 104
Table S12 104
Table S13 104
Supplementary Methods 106
Section SA Construction of machine learning models 106
Section SB Collection of rhodamine optical data 107
Section SC Quantum mechanics (QM) calculation 115
Section SD Pre-training Process 115
Section SE Synthesis of designed molecules 116
References 135

Supplementary Figures

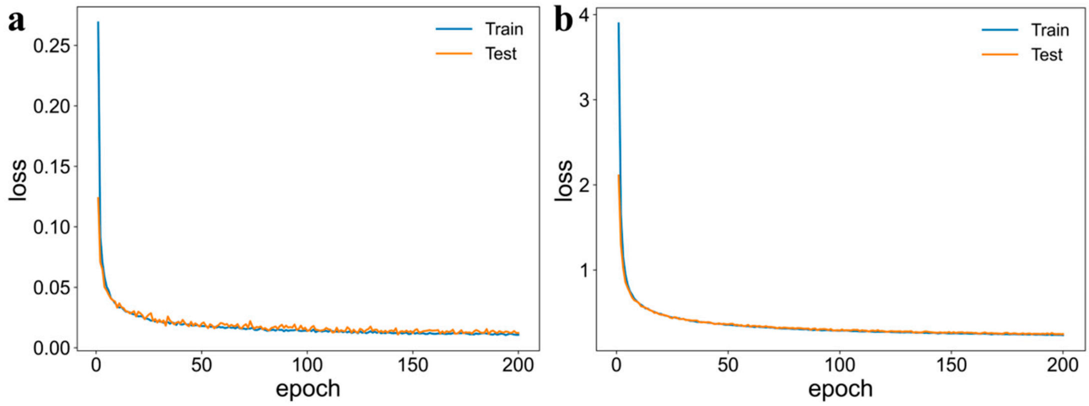

Figure S1. Loss curves of pre-trained models. (a) GCN and (b) GAT.

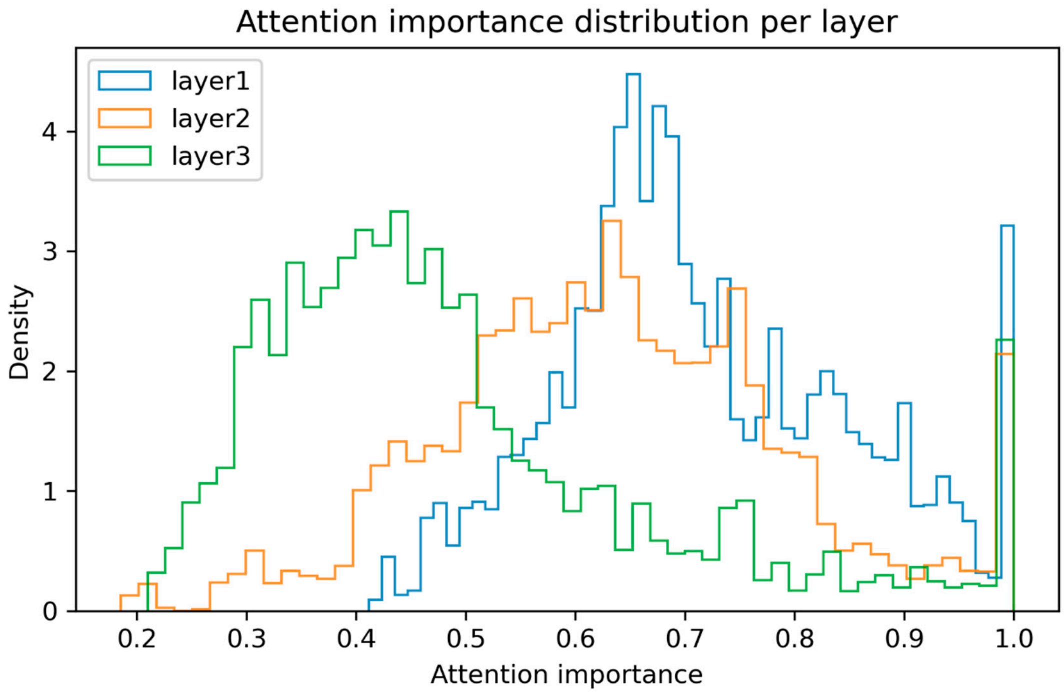

Figure S2. Attention importance distribution.

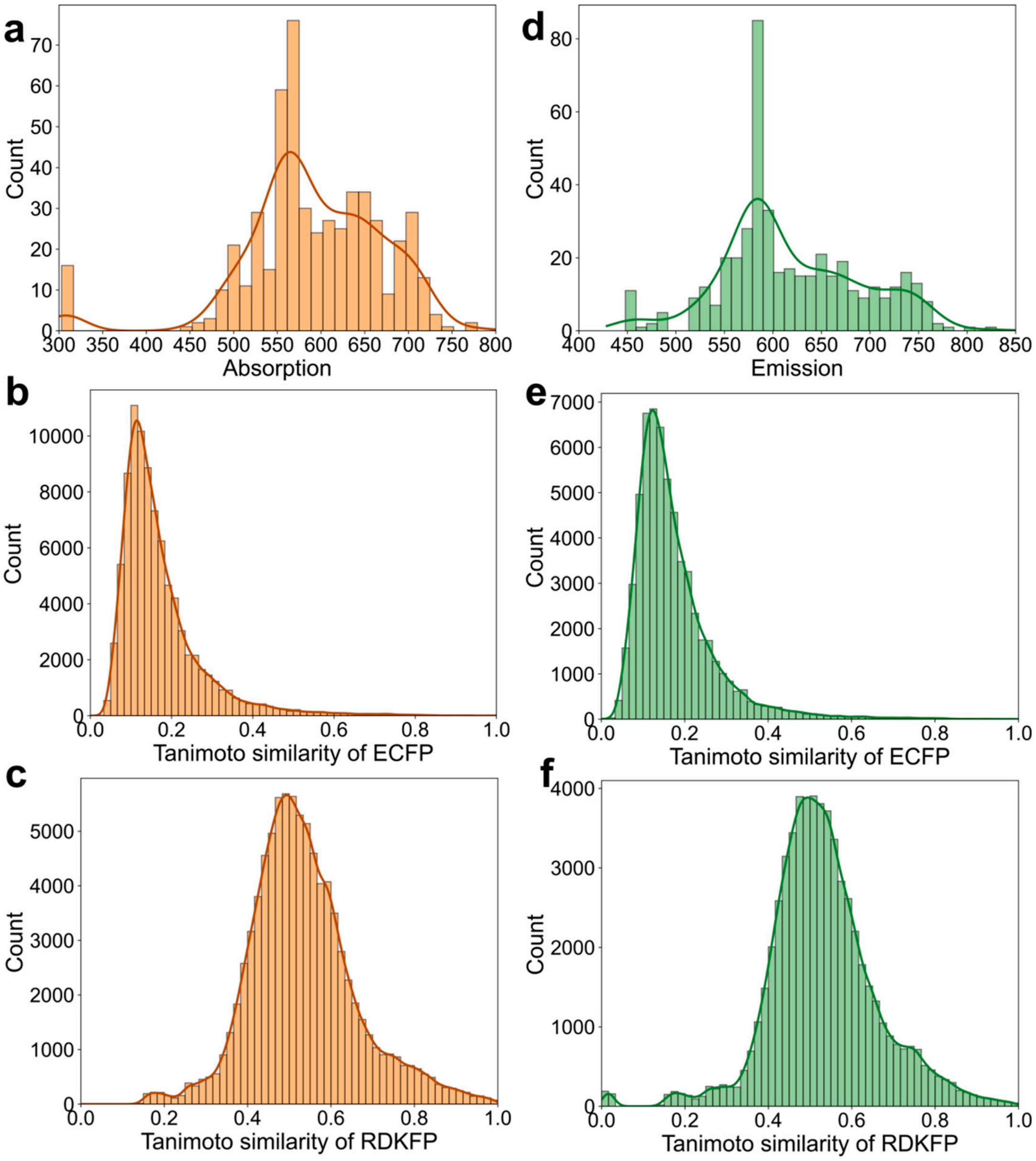

Figure S3. Distribution and fingerprint similarity analysis of absorption and emission data. (a-c) are for λ_{abs} dataset and (d-f) for λ_{emi} dataset.

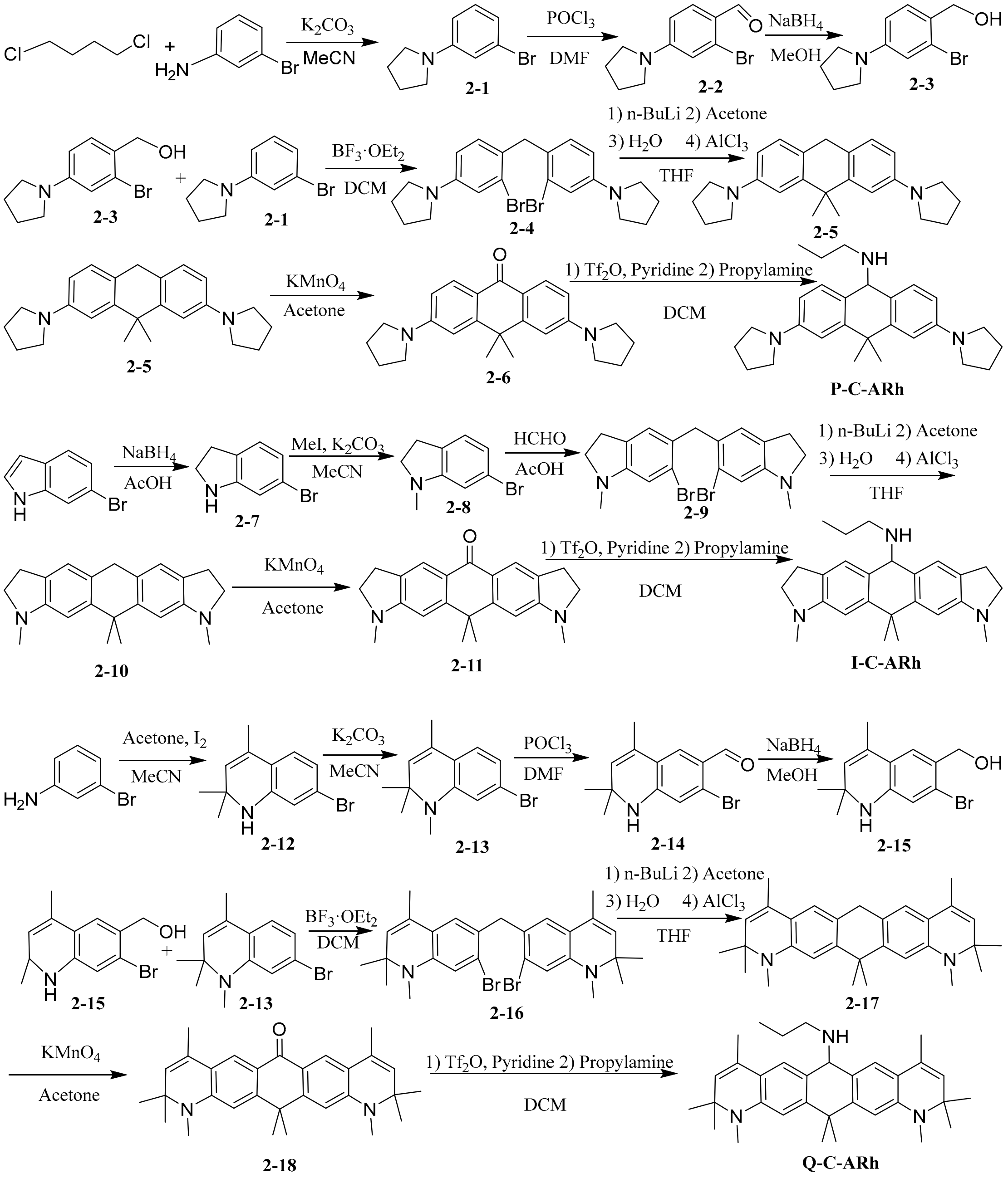

Figure S4. Detailed synthetic routes of **P-C-ARh**, **I-C-ARh**, and **Q-C-ARh**.

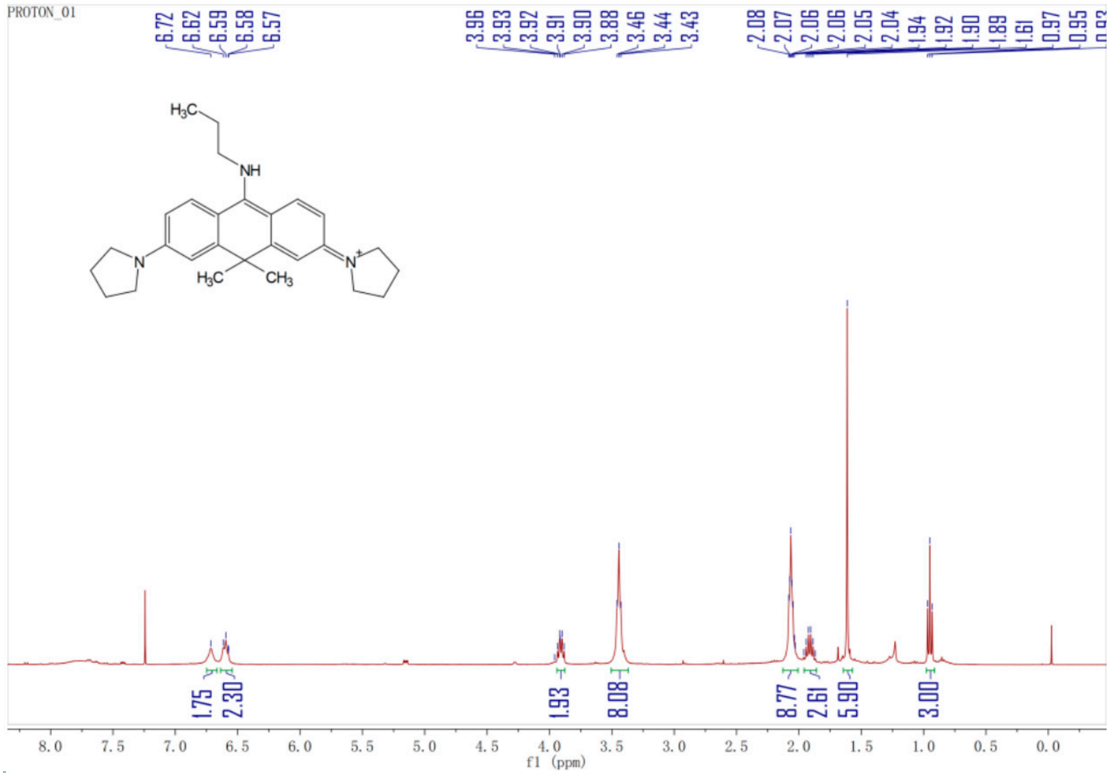
PROTON_01
6.72
6.62
6.59
6.58
6.57
3.96
3.93
3.92
3.91
3.90
3.88
3.46
3.44
3.43
2.08
2.07
2.06
2.06
2.05
2.04
1.94
1.92
1.90
1.89
1.61
0.97
0.95
1.75
2.30
1.93
8.08
8.77
2.61
5.90
3.00
f1 (ppm)

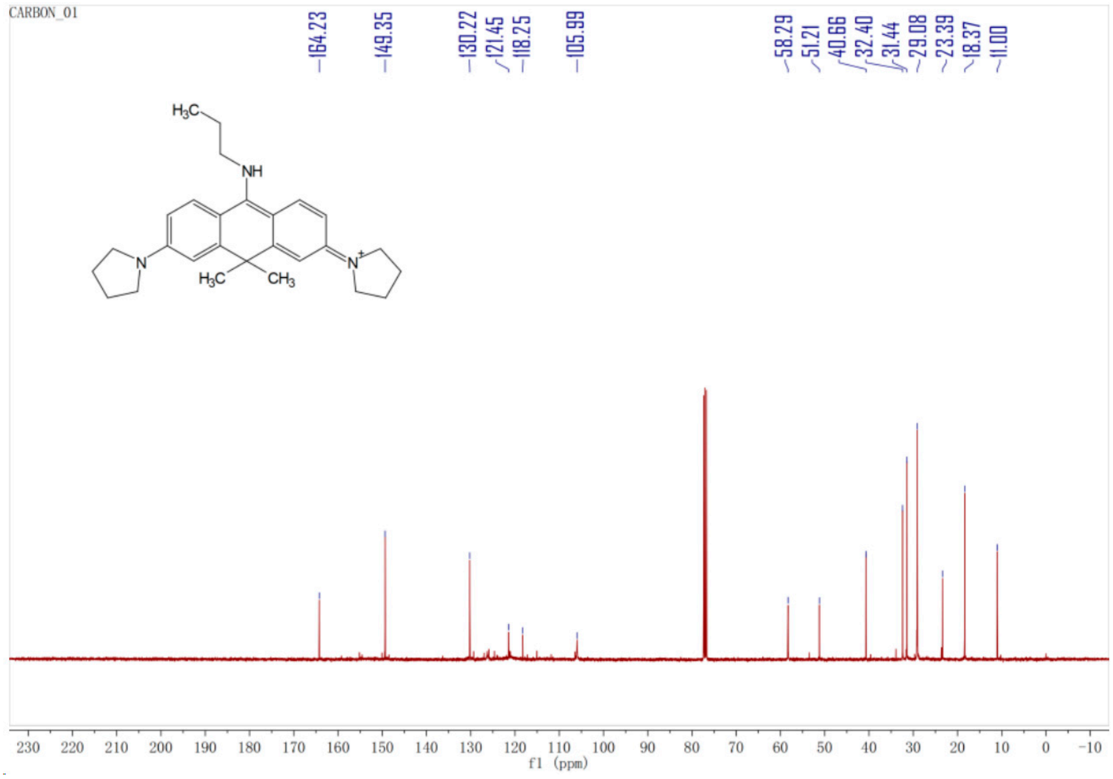
CARBON_01
164.23
149.35
130.22
121.45
118.25
105.99
58.29
51.21
40.66
32.40
31.44
29.08
23.39
18.37
11.00
f1 (ppm)

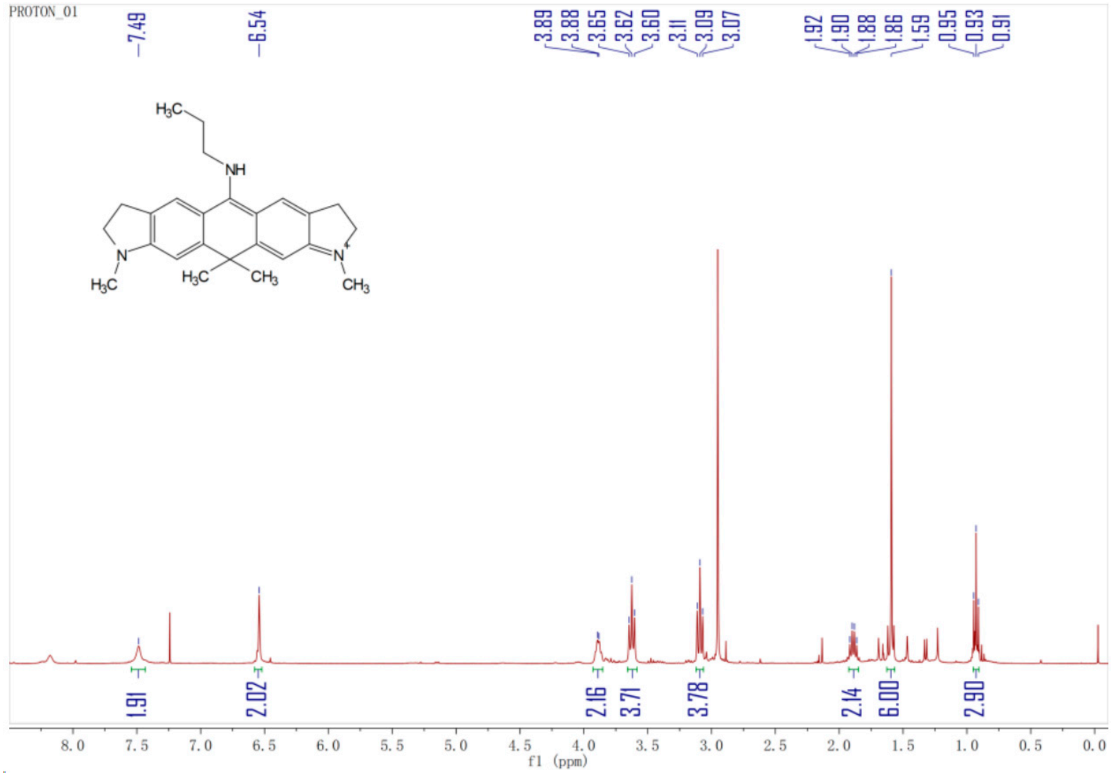
PROTON_01
7.49
6.54
3.89
3.88
3.65
3.62
3.60
3.11
3.09
3.07
1.92
1.90
1.88
1.86
1.59
0.95
0.93
0.91
1.91
2.02
2.16
3.71
3.78
2.14
6.00
2.90
f1 (ppm)

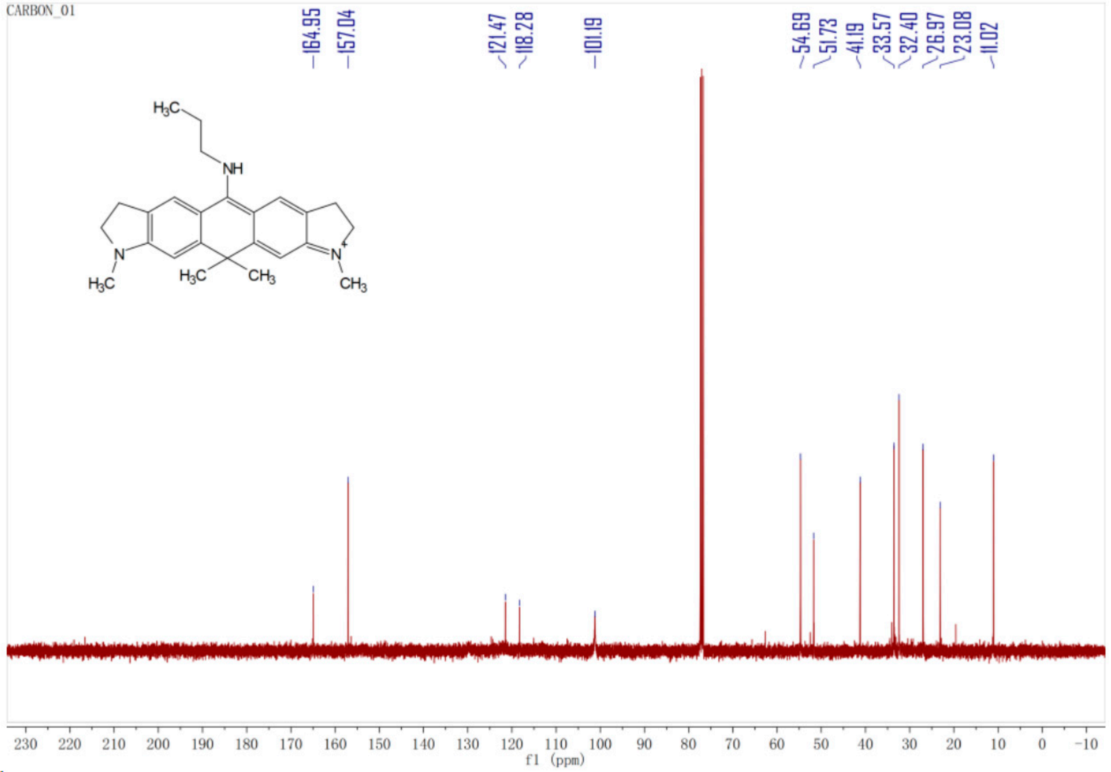
CARBON_01
164.95
157.04
121.47
118.28
101.19
54.69
51.73
41.19
33.57
32.40
26.97
23.08
11.02
f1 (ppm)

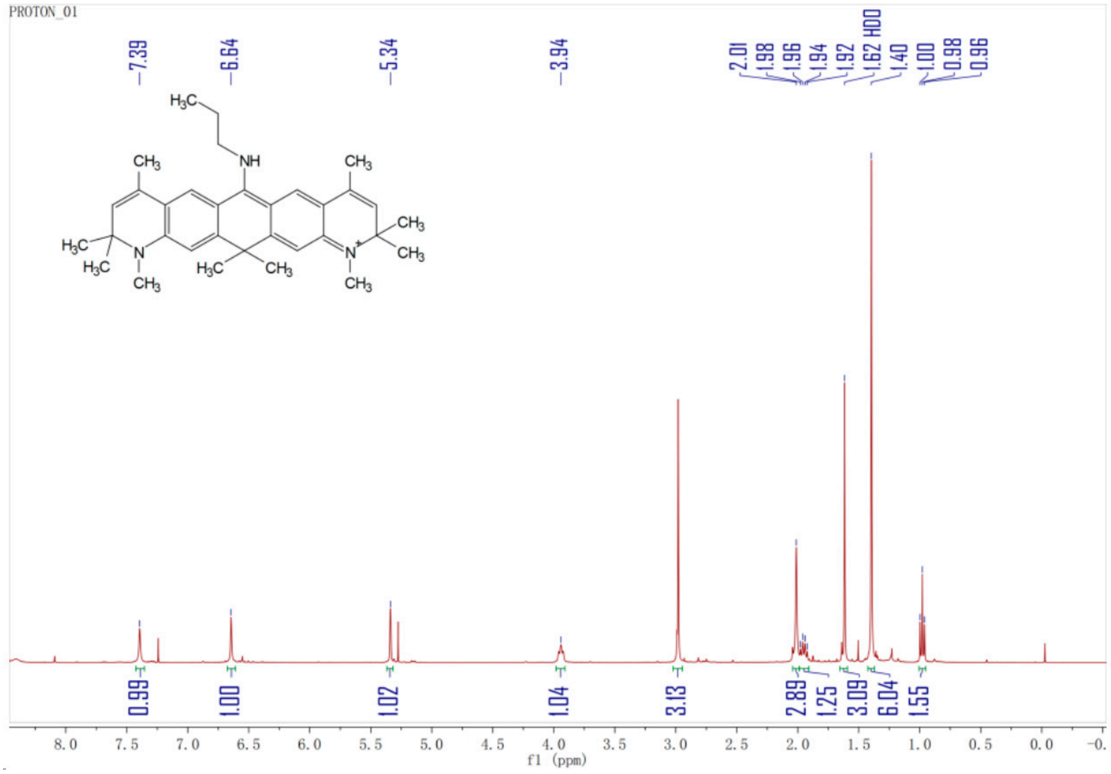

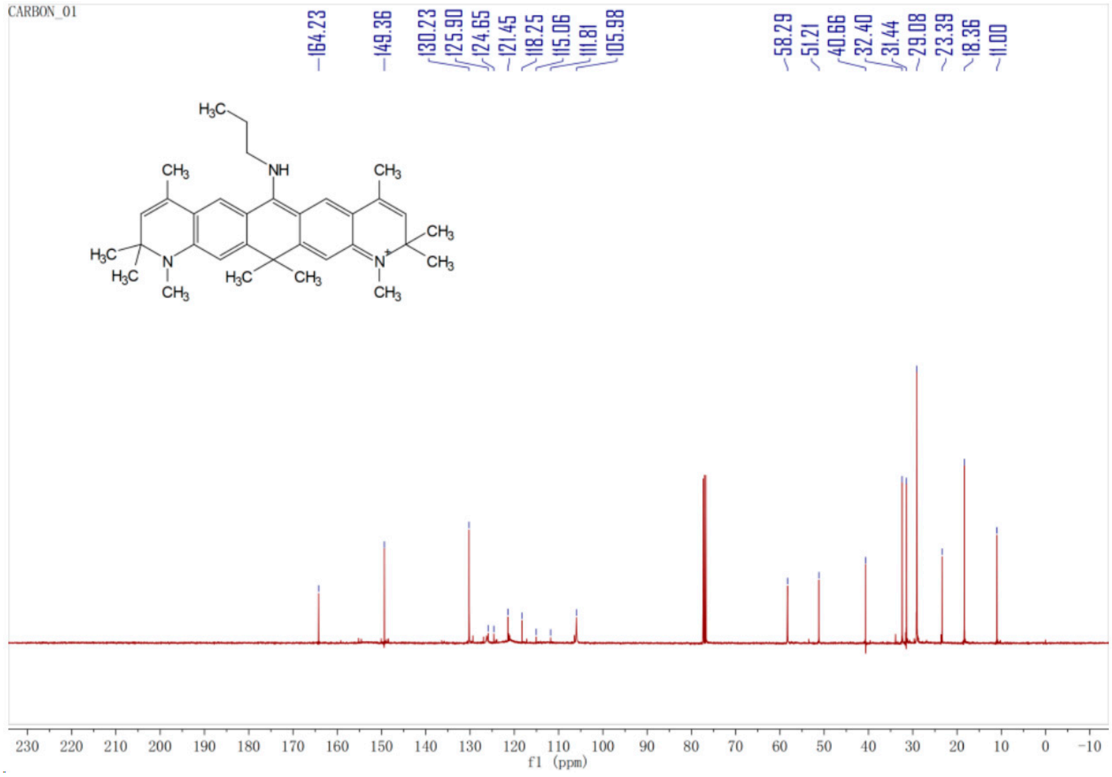

Figure S5. NMR spectra of **P-C-ARh**, **I-C-ARh**, and **Q-C-ARh**.

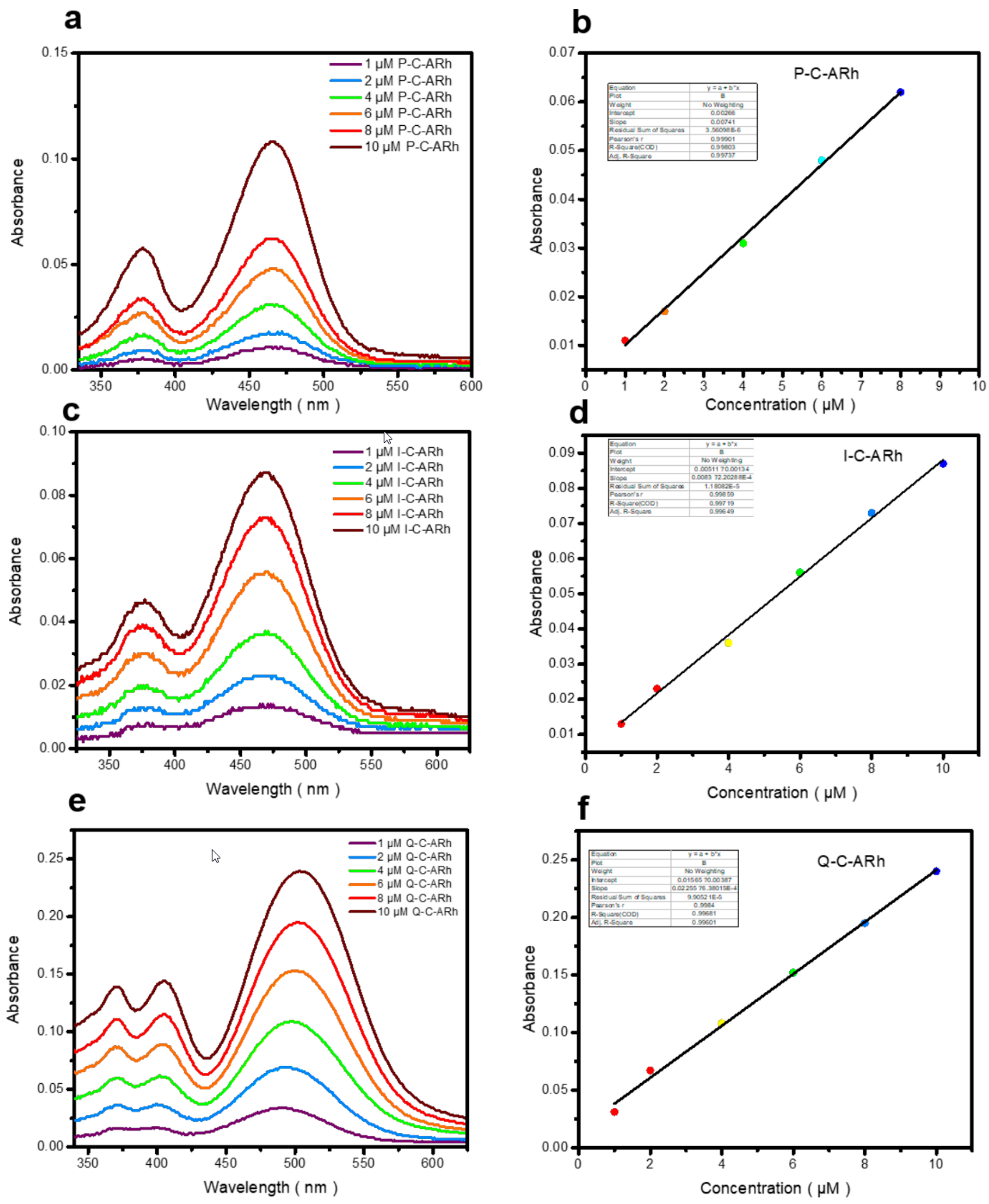

Figure S6. (a), (c), and (e) show the UV–visible absorption spectra of probe molecules **P-C-ARh**, **I-C-ARh**, and **Q-C-ARh**, respectively, at concentrations of 1, 2, 4, 6, 8, and 10 μM in PBS buffer (pH 7.4); (b), (d), and (f) show the corresponding linear fitting plots of absorbance versus concentration at their respective maximum absorption wavelengths.

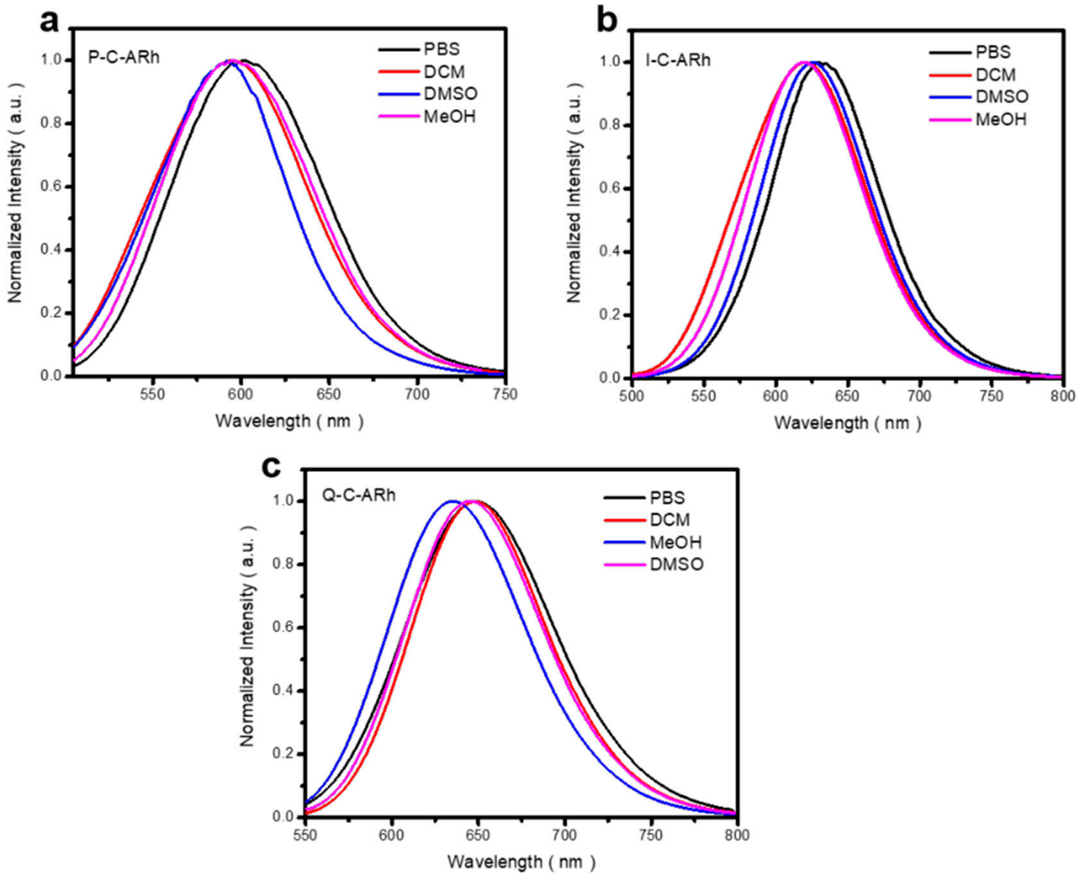

Figure S7. (a), (b), and (c) show the normalized fluorescence emission spectra of probe molecules **P-C-ARh**, **I-C-ARh**, and **Q-C-ARh** in dichloromethane, methanol, dimethyl sulfoxide, and PBS buffer, respectively. The concentration of the probe molecules in all tested systems was 10 μM.

Supplementary Tables

Table S1. Detailed results of ML models, single-task (ST) DL models, multitask (MT) DL models, and DL models without pre-training (WoP) on training and test set. The results are displayed as mean ± standard deviation over 100 Monte Carlo cross-validation rounds.

Model	Task	R^2 Train	RMSE Train	MAE Train
RF	λ_{abs}	0.729 ± 0.012	41.985 ± 0.809	31.061 ± 0.787
	λ_{emi}	0.687 ± 0.014	39.899 ± 1.000	28.684 ± 0.845
SVM	λ_{abs}	0.785 ± 0.006	37.443 ± 0.980	19.030 ± 0.623
	λ_{emi}	0.777 ± 0.014	33.681 ± 1.213	19.869 ± 0.674
KNN	λ_{abs}	0.821 ± 0.015	34.108 ± 0.981	22.751 ± 0.827
	λ_{emi}	1.000 ± 0.000	0.004 ± 0.022	0.000 ± 0.002
XGBoost	λ_{abs}	0.860 ± 0.005	30.160 ± 0.531	22.359 ± 0.478
	λ_{emi}	0.804 ± 0.010	31.585 ± 0.930	22.865 ± 0.691
ST_GCN	λ_{abs}	0.956 ± 0.013	16.749 ± 2.264	13.082 ± 1.840
	λ_{emi}	0.804 ± 0.017	31.107 ± 6.469	22.964 ± 1.660
ST_GAT	λ_{abs}	0.938 ± 0.022	19.392 ± 3.401	15.189 ± 2.822
	λ_{emi}	0.875 ± 0.059	24.588 ± 5.397	18.931 ± 4.247
MT_GCN	λ_{abs}	0.959 ± 0.015	16.459 ± 2.804	12.776 ± 2.353
	λ_{emi}	0.948 ± 0.020	16.149 ± 2.905	12.708 ± 2.378
MT_GAT	λ_{abs}	0.948 ± 0.019	17.976 ± 3.030	14.003 ± 2.558
	λ_{emi}	0.929 ± 0.026	18.714 ± 3.217	14.547 ± 2.628
ST_GCN_	λ_{abs}	0.955 ± 0.016	17.027 ± 2.812	13.327 ± 2.280
WoP	λ_{emi}	0.724 ± 0.073	37.262 ± 4.546	29.282 ± 3.615
ST_GAT_W	λ_{abs}	0.968 ± 0.004	14.293 ± 0.997	11.009 ± 0.793
oP	λ_{emi}	0.847 ± 0.087	27.097 ± 7.133	20.877 ± 5.643
MT_GCN_	λ_{abs}	0.958 ± 0.012	16.709 ± 2.313	12.959 ± 1.959
WoP	λ_{emi}	0.947 ± 0.017	16.403 ± 2.592	12.919 ± 2.083
MT_GAT_	λ_{abs}	0.946 ± 0.030	18.383 ± 4.464	14.308 ± 3.643
WoP	λ_{emi}	0.925 ± 0.041	19.102 ± 4.506	14.858 ± 3.641
**Model**	**Task**	**R^2 Test**	**RMSE Test**	**MAE Test**
RF	λ_{abs}	0.659 ± 0.080	45.374 ± 3.274	33.287 ± 2.966
	λ_{emi}	0.610 ± 0.072	44.418 ± 4.329	31.898 ± 3.316
SVM	λ_{abs}	0.736 ± 0.032	40.608 ± 5.607	24.455 ± 2.913
	λ_{emi}	0.668 ± 0.065	41.046 ± 5.097	27.251 ± 3.067
KNN	λ_{abs}	0.756 ± 0.063	38.468 ± 3.858	25.983 ± 2.891
	λ_{emi}	0.668 ± 0.078	40.911 ± 5.490	26.153 ± 3.376
XGBoost	λ_{abs}	0.784 ± 0.049	36.198 ± 3.085	26.156 ± 2.266
	λ_{emi}	0.706 ± 0.061	38.578 ± 4.528	27.076 ± 3.085
ST_GCN	λ_{abs}	0.860 ± 0.050	29.386 ± 4.098	19.844 ± 1.971
	λ_{emi}	0.723 ± 0.054	37.364 ± 3.533	29.341 ± 2.733
ST_GAT	λ_{abs}	0.932 ± 0.008	22.318 ± 1.866	16.088 ± 1.258
	λ_{emi}	0.776 ± 0.025	34.300 ± 2.289	24.434 ± 1.927
MT_GCN	λ_{abs}	0.902 ± 0.045	24.308 ± 3.923	16.728 ± 2.314

	λ_{emi}	0.886 ± 0.036	23.623 ± 3.342	16.247 ± 2.198
MT_GAT	λ_{abs}	0.923 ± 0.025	24.239 ± 3.340	16.427 ± 1.889
	λ_{emi}	0.913 ± 0.010	21.573 ± 1.630	15.666 ± 1.211
ST_GCN_	λ_{abs}	0.824 ± 0.026	31.647 ± 2.956	21.037 ± 1.853
WoP	λ_{emi}	0.743 ± 0.028	36.669 ± 2.774	25.993 ± 1.902
ST_GAT_W	λ_{abs}	0.851 ± 0.044	30.419 ± 3.746	20.483 ± 2.170
oP	λ_{emi}	0.639 ± 0.102	42.606 ± 6.227	28.919 ± 3.588
MT_GCN_	λ_{abs}	0.875 ± 0.044	26.834 ± 4.878	18.416 ± 2.860
WoP	λ_{emi}	0.847 ± 0.031	27.281 ± 2.962	18.505 ± 2.052
MT_GAT_	λ_{abs}	0.863 ± 0.066	29.292 ± 4.831	19.442 ± 2.938
WoP	λ_{emi}	0.807 ± 0.075	31.001 ± 5.447	20.217 ± 3.033

Table S2. The R^2 contributions of MT_GAT for from 100 rounds.

Round	λ_{abs} R^2	λ_{emi} R^2	*d*i	Round	λ_{abs} R^2	λ_{emi} R^2	*d*i
1	0.904	0.910	0.01944	51	0.919	0.914	0.00313
2	0.932	0.905	0.01306	52	0.926	0.924	0.01060
3	0.937	0.927	0.01955	53	0.884	0.915	0.03854
4	0.924	0.904	0.00985	54	0.931	0.903	0.01339
5	0.904	0.905	0.02052	55	0.943	0.916	0.02018
6	0.931	0.913	0.00852	56	0.915	0.924	0.01257
7	0.808	0.921	0.11464	57	0.910	0.922	0.01555
8	0.921	0.907	0.00690	58	0.909	0.904	0.01668
9	0.901	0.906	0.02261	59	0.940	0.902	0.02084
10	0.924	0.919	0.00585	60	0.907	0.932	0.02421
11	0.921	0.934	0.02043	61	0.936	0.905	0.01568
12	0.899	0.904	0.02537	62	0.937	0.941	0.03154
13	0.952	0.939	0.03887	63	0.935	0.904	0.01588
14	0.923	0.926	0.01212	64	0.895	0.911	0.02768
15	0.948	0.924	0.02788	65	0.923	0.904	0.00977
16	0.926	0.922	0.00948	66	0.911	0.911	0.01225
17	0.940	0.920	0.01877	67	0.916	0.921	0.01044
18	0.942	0.904	0.02148	68	0.908	0.902	0.01882
19	0.898	0.907	0.02522	69	0.949	0.908	0.02694
20	0.916	0.902	0.01323	70	0.941	0.912	0.01836
21	0.842	0.919	0.08048	71	0.951	0.904	0.02964
22	0.921	0.902	0.01143	72	0.857	0.904	0.06573
23	0.940	0.922	0.01908	73	0.940	0.912	0.01704
24	0.919	0.906	0.00865	74	0.942	0.924	0.02189
25	0.890	0.909	0.03329	75	0.911	0.919	0.01334
26	0.950	0.913	0.02708	76	0.958	0.949	0.05011
27	0.942	0.905	0.02141	77	0.931	0.916	0.00844
28	0.956	0.914	0.03314	78	0.932	0.936	0.02408
29	0.906	0.922	0.01909	79	0.899	0.905	0.02471
30	0.932	0.905	0.01264	80	0.885	0.910	0.03787
31	0.932	0.905	0.01268	81	0.935	0.906	0.01448
32	0.846	0.906	0.07734	82	0.897	0.913	0.02577
33	0.932	0.903	0.01421	83	0.936	0.908	0.01484
34	0.961	0.925	0.04058	84	0.927	0.918	0.00687

35	0.919	0.918	0.00550	85	0.909	0.907	0.01517
36	0.918	0.923	0.01061	86	0.948	0.912	0.02525
37	0.950	0.920	0.02765	87	0.928	0.910	0.00635
38	0.925	0.914	0.00249	88	0.930	0.905	0.01150
39	0.939	0.905	0.01843	89	0.913	0.909	0.01048
40	0.906	0.918	0.01728	90	0.884	0.911	0.03875
41	0.944	0.925	0.02414	91	0.918	0.918	0.00649
42	0.928	0.919	0.00799	92	0.941	0.913	0.01823
43	0.944	0.908	0.02223	93	0.935	0.909	0.01298
44	0.926	0.904	0.01003	94	0.942	0.909	0.01989
45	0.936	0.916	0.01333	95	0.941	0.907	0.01927
46	0.934	0.904	0.01448	96	0.927	0.917	0.00575
47	0.928	0.908	0.00766	97	0.910	0.914	0.01248
48	0.938	0.910	0.01534	98	0.923	0.903	0.01032
49	0.907	0.921	0.01700	99	0.942	0.904	0.02103
50	0.951	0.928	0.03190	100	0.923	0.907	0.00669

Table S3. Molecular names in the D_{32} dataset and their corresponding SMILES.

name	SMILES
P-Si-ARh	CCCNC(C1=CC=C(N2CCCC2)C=C1[Si](C)(C)C3=C/4)=C3C=CC4=[N+]5CCCC/5
I-Si-ARh	CCCNC1=C2C=C3CC[N+](C)=C3C=C2[Si](C)(C)C4=CC5=C(CCN5C)C=C14
Q-Si-ARh	CCCNC(C1=CC(C(C)=CC(C)(N2C)C)=C2C=C1[Si](C)(C)C3=CC4=[N+]5C)=C3C=C4C(C)=CC5(C)C
P-P-ARh	CCCNC(C1=CC=C(N2CCCC2)C=C1P3(C4=CC=CC=C4)=O)=C(C3=C/5)C=CC5=[N+]6CCCC/6
I-P-ARh	CCCNC(C1=CC(CCN2C)=C2C=C13)=C4C=C5CC[N+](C)=C5C=C4P3(C6=CC=CC=C6)=O
Q-P-ARh	CCCNC(C1=CC(C(C)=CC(C)(N2C)C)=C2C=C1P3(C4=CC=CC=C4)=O)=C(C3=CC5=[N+]6C)C=C5C(C)=CC6(C)C
P-S-ARh	CCCNC(C1=CC=C(N2CCCC2)C=C1S3(=O)=O)=C(C3=C/4)C=CC4=[N+]5CCCC/5
N-S-ARh	CCCNC(C1=CC(CCCN2C)=C2C=C13)=C4C=C5CCC[N+](C)=C5C=C4S3(=O)=O
J-S-ARh	O=S1(C2=C3C4=C(C=C2C(NCCC)=C5C=C6CCC[N+]7=C6C(CCC7)=C51)CCCN4CCC3)=O
I-S-ARh	CN1CCC2=C1C=C3C(C(NCCC)=C4C=C5CC[N+](C)=C5C=C4S3(=O)=O)=C2
M-S-ARh	O=S1(C2=CC(N(C)C)=CC=C2C(NCCC)=C3C1=C/C(C=C3)=[N+](C)/C)=O
FEN	CCCNC1=C2C(C3=CC(N(CC)CC)=CC=C31)=C/C(C=C2)=[N+](CC)/CC
FMN	CN(C)C1=CC2=C(C=C1)C(NCCC)=C3C2=C/C(C=C3)=[N+](C)\C
FAN	CCCNC1=C2C(C3=C1C=CC(N4CCC4)=C3)=C/C(C=C2)=[N+]5CCC\5

FPN	CCCNC1=C2C(C3=C1C=CC(N4CCCC4)=C3)=C/C(C=C2)=[N+]5CCCC\5
Si-Ph-NH2	CC1(C)C=C(C)C2=CC3=C(C4=CC=C(N)C=C4[Si](C)(C)C3=CC2=[N+]1C)C5=CC=CC=C5
Si-Ph-Ac	CC(C1=CC2=C(C3=CC=C(NC(C)=O)C=C3[Si](C)(C)C2=CC1=[N+]4C)C5=CC=CC=C5)=CC4(C)C
Si-2OMe-NH2	COC1=C(C(C2=CC=C(N)C=C2[Si]3(C)C)=C4C=C(C(C=C43)=[N+]5C)C(C)=CC5(C)C)C=CC=C1
Si-2OMe-Ac	CC(C1=CC2=C(C3=CC=C(NC(C)=O)C=C3[Si](C)(C)C2=CC1=[N+]4C)C5=C(OC)C=CC=C5)=CC4(C)C
Si-2,6OMe-NH2	COC1=C(C(C2=CC=C(N)C=C2[Si]3(C)C)=C4C=C(C(C=C43)=[N+]5C)C(C)=CC5(C)C)C(OC)=CC=C1
Si-2,6OMe-Ac	CC(C1=CC2=C(C3=CC=C(NC(C)=O)C=C3[Si](C)(C)C2=CC1=[N+]4C)C5=C(OC)C=CC=C5OC)=CC4(C)C
SiR-pH	NC1=CC=C(C2(C3=C(CO2)C=CC=C3)C4=CC5=C(N(C)C(C)(C)C=C5C)C=C4[Si]6(C)C)C6=C1
SiR-IapH	NC1=CC=C(C2(C3=C(CO2)C=CC=C3)C4=CC=C(N5CCCC5)C=C4[Si]6(C)C)C6=C1
DMBA	CN(C(/C(C1=O)=C2C3=CC(C=CC4(C)C)=C(N4C)C=C3[Si](C)(C)C5=CC6=C(C=C\25)C=CC(C)(N6C)C)=O)C(N1C)=O
CN	CC(C1=C2C=C3C(/C(C4=CC(C(C)=CC5(C)C)=C(N5C)C=C4[Si]3(C)C)=C(C#N)\C#N)=C1)=CC(C)(N2C)C
IDMN	CC(C1=C2C=C3C(/C(C4=CC(C(C)=CC5(C)C)=C(N5C)C=C4[Si]3(C)C)=C6/C(C7=C(C\6=C(C#N)/C#N)CCC=C7)=C(C#N)/C#N)=C1)=CC(C)(N2C)C
ID	CC(C(C=C1/C(C2=CC(C(C)=CC3(C)C)=C(N3C)C=C2[Si](C)(C)C1=C4)=C5C(C6=C(C\5=O)C=CC=C6)=O)=C4N7C)=CC7(C)C
O-4Py	CN(C)C1=CC=C2C(OC(C=C(N(C)C)C=C3)=C3/C2=N/C4=CC=CC=C4)=C1
C-4Py	CN(C)C1=CC=C2C(C(C)(C)C(C=C(N(C)C)C=C3)=C3/C2=N/C4=CC=CC=C4)=C1
Si-4Py	CN(C)C1=CC=C2C([Si](C)(C)C(C=C(N(C)C)C=C3)=C3/C2=N/C4=CC=CC=C4)=C1
P-4Py	CN(C)C1=CC=C2C(P(C(C=C(N(C)C)C=C3)=C3/C2=N/C4=CC=CC=C4)(C5=CC=CC=C5)=O)=C1
SO-4Py	CN(C)C1=CC=C2C(S(C(C=C(N(C)C)C=C3)=C3/C2=N/C4=CC=CC=C4)(=O)=O)=C1

Table S4. Results of per-compound prediction in external validation of D_{abs} set.

SMILES	Solvent (SMILES)	λ_{abs}-Exp.	λ_{abs}-Pred.
CCN(CC)c1ccc2c(-c3ccccc3CO)c3ccc(=[N+](CC)CC)cc-3oc2c1	O	559	526
CCN(CC)c1ccc2c(c1)C=CC(C(=O)NCCC(=O)OCc1ccccc1-c1c3ccc(=[N+](CC)CC)cc-3oc3cc(N(CC)CC)ccc13)O2	O	585	569
Cc1cccc2c1COC21c2ccc(N)cc2Oc2cc(N)ccc21	O	505	494
Nc1ccc2c(c1)Oc1cc(N)ccc1C21OCc2c(F)cccc21	O	508	485

Nc1ccc2c(c1)Oc1cc(N)ccc1C21OCc2c(C(F)(F)F)cccc21	O	505	496
Cc1ccc2c(c1)C1(OC2)c2ccc(N)cc2Oc2cc(N)ccc21	O	505	489
C[Si]1(C)c2cc(N)ccc2C2(OCc3sccc32)c2ccc(N)cc21	O	600	590
C[Si]1(C)c2cc(N)ccc2C2(OCc3sccc32)c2ccc(NC(=O)CCC(N)C(=O)O)cc21	O	500	480
CC1OC2(c3ccc(N)cc3C(C)(C)c3cc(N)ccc32)c2ccsc21	O	560	551
CC1OC2(c3ccc(N)cc3C(C)(C)c3cc(NC(=O)CCC(N)C(=O)O)ccc32)c2ccsc21	O	490	485
N=c1ccc2c(-c3ccccc3CO)c3ccc(NC(=O)CCC(N)C(=O)O)cc3[se]c-2c1	O	450	450
CC(=O)N=c1ccc2c(-c3ccccc3CO)c3ccc(N)cc3oc-2c1	O	493	481
CC(=O)N=c1ccc2c(-c3ccccc3CN)c3ccc(N)cc3oc-2c1	O	500	496
CN(C)c1ccc2c(c1)[Si](C)(C)C1=CC(=[N+](C)C)C=CC1=C2c1ccc(C(=O)O)cc1CS	O	653	626
CN(C)c1ccc2c(c1)[Si](C)(C)C1=CC(=[N+](C)C)C=CC1=C2c1ccc(C(=O)O)cc1CN	O	656	618
CCN=c1cc2oc3cc(NCC)c(C)cc3c(-c3ccccc3CO)c-2cc1C	O	525	512
OCc1ccccc1C1=c2cc3c4c(c2Oc2c1cc1c5c2CCCN5CCC1)CCC[N+]=4CCC3	O	579	551
CN(C)c1ccc2c(-c3ccccc3CS)c3ccc(=[N+](C)C)cc-3oc2c1	O	554	541
CN(C)c1ccc2c(-c3ccccc3CN)c3ccc(=[N+](C)C)cc-3oc2c1	O	557	531
C=CC(=O)Nc1ccc(-c2c3ccc(=[N+](CC)CC)cc-3oc3cc(N(CC)CC)ccc23)c(C(=O)O)c1	O	558	551
CCCCNc1ccc(-c2c3ccc(=[N+](CC)CC)cc-3oc3cc(N(CC)CC)ccc23)c(C(=O)O)c1	O	558	548
CCCCCCCCNc1ccc(-c2c3ccc(=[N+](CC)CC)cc-3oc3cc(N(CC)CC)ccc23)c(C(=O)O)c1	O	563	541
CCN(CC)c1ccc2c(-c3ccc(N4CCCCCC4)cc3C(=O)O)c3ccc(=[N+](CC)CC)cc-3oc2c1	O	558	546
CCN(CC)c1ccc2c(-c3ccccc3C(=O)NCCN3CCOCC3)c3ccc(=[N+](CC)CC)cc-3oc2c1	O	563	530
CCN(CC)c1ccc2c(-c3ccccc3C(=O)NCCNc3ncc(C(=O)OC)c(NCc4ccccc4)n3)c3ccc(=[N+](CC)CC)cc-3oc2c1	C1CCCO1	306	289
CC1=CC(C)=[N+]2C1=C(c1ccccc1)c1c(C)cc(/C=C/c3ccc(N(C)C)cc3)n1[B-]2(F)F	CC#N	597	590

Cc1ccccc1-c1c2ccc(=[N+](C)C)cc-2[te]c2cc(N(C)C)ccc12	O	600	567
COc1ccc(N(C)c2ccc3c(c2)[Si](C)(C)C2=CC(=[N+](C)c4ccc(OC)cc4)C=CC2=C3c2ccccc2C)cc1	CCO	660	621
COc1ccc(N(C)c2ccc3c(c2)[Si](C)(C)C2=CC(=[N+](C)c4ccc(OC)cc4)C=CC2=C3c2ccccc2C)cc1	CO	660	621
Cc1ccccc1C1=C2C=CC(=[N+]3CCc4ccccc43)C=C2[Si](C)(C)c2cc(N3CCc4ccccc43)ccc21	CCO	779	724
CCN(CC)c1cc2c(c(-c3ccccc3C(=O)O)c1)C=c1cc3c(cc1O2)=[N+](CC)c1ccccc1S3	CCO	606	578
CCN(CC)c1ccc2c(C=CC=C3N(C)c4ccccc4C3(C)C)c3ccc(N(CC)CC)cc3[o+]c2c1	CC#N	651	631
CCN(CC)c1ccc2c(C=CC=C3Sc4ccccc4N3C)c3ccc(N(CC)CC)cc3[o+]c2c1	CC#N	663	639
CCN(CC)c1ccc2c(C=CC=C3C=Cc4ccccc4N3C)c3ccc(N(CC)CC)cc3[o+]c2c1	CC#N	684	656
CN(C)c1ccc2c(c1)P(=O)(c1ccccc1)C1=CC(=[N+](C)C)C=CC1=C2c1ccccc1	O	698	675
COc1ccccc1C1=C2C=CC(=[N+](C)C)C=C2P(=O)(c2ccccc2)c2cc(N(C)C)ccc21	O	701	679
COc1cccc(OC)c1C1=C2C=CC(=[N+](C)C)C=C2P(=O)(c2ccccc2)c2cc(N(C)C)ccc21	O	703	687
CCc1ccccc1C1=C2C=CC(=[N+](C)C)C=C2P(=O)(c2ccccc2)c2cc(N(C)C)ccc21	O	699	672
Cc1cccc(C)c1C1=C2C=CC(=[N+](C)C)C=C2P(=O)(c2ccccc2)c2cc(N(C)C)ccc21	O	730	691
Cc1cc(C)c(C2=C3C=CC(=[N+](C)C)C=C3P(=O)(c3ccccc3)c3cc(N(C)C)ccc32)c(C)c1	O	700	682
Cc1ccccc1C1=C2C=CC(=[N+](C)C)C=C2P(=O)(C(C)(C)C)c2cc(N(C)C)ccc21	O	693	658
COc1cccc(OC)c1C1=C2C=CC(=[N+](C)C)C=C2P(=O)(C(C)(C)C)c2cc(N(C)C)ccc21	O	697	659
Cc1ccccc1C1=C2C=CC(=[N+](C)C)C=C2P(=O)(c2ccccc2)c2cc(N(C)C)ccc21	O	699	679
Cc1ccccc1C1=C2C=CC(=[N+]3CCC3)C=C2P(=O)(c2ccccc2)c2cc(N3CCC3)ccc21	O	699	687
Cc1ccccc1C1=C2C=CC(=[N+]3CCCC3)C=C2P(=O)(c2ccccc2)c2cc(N3CCCC3)ccc21	O	708	690
CN(C)c1ccc2c(c1)P(=O)(c1ccccc1)C1=CC(=[N+](C)C)C=CC1=C2c1ccccc1C(F)(F)F	O	706	677
O=P1(c2ccccc2)C2=CC(=[N+]3CCC3)C=CC2=C(c2ccccc2C(F)(F)F)c2ccc(N3CCC3)cc21	O	710	685
O=P1(c2ccccc2)C2=CC(=[N+]3CCCC3)C=CC2=C(c2ccccc2C(F)(F)F)c2ccc(N3CCCC3)cc21	O	718	690
Cc1ccccc1C1=C2C=CC(=[N+]3CCCC3)C=C2P(=O)(c2ccccc2)c2cc(N(C)C)ccc21	O	703	683

CCc1ccccc1C1=C2C=CC(=[N+]3CCCC3)C=C2P(=O)(c2ccccc2)c2cc(N(C)C)ccc21	O	703	676
CN(C)c1ccc2c(c1)P(=O)(c1ccccc1)C1=CC(=[N+]3CCCC3)C=CC1=C2c1ccccc1C(F)(F)F	O	713	680
COc1ccccc1C1=C2C=CC(=[N+]3CCCC3)C=C2P(=O)(c2ccccc2)c2cc(N(C)C)ccc21	O	706	682
COc1cccc(OC)c1C1=C2C=CC(=[N+]3CCCC3)C=C2P(=O)(c2ccccc2)c2cc(N(C)C)ccc21	O	706	689
O=S(=O)([O-])c1ccccc1-[c+]1c2ccc(N3C4CCC3CC4)cc2oc2cc(N3C4CCC3CC4)ccc21	CO	548	524
O=C(O)c1ccccc1-c1c2cc3c4c(c2[o+]c2c5c6c(cc12)CCCN6CCC5)CCCN4CCC3	CO	568	556
O=C(O)c1ccccc1-c1c2cc3c4c(c2[o+]c2c5c6c(cc12)CCCN6CCC5)CCCN4CCC3	CS(C)=O	567	554
CN1c2cc3c(cc2-c2sc4ccccc4c2C1(C)C)C(c1ccc(S(=O)(=O)[O-])cc1S(=O)(=O)O)=c1cc2c(cc1O3)=[N+](C)C(C)(C)c1c-2sc2ccccc12	CO	616	584
CN1c2cc3c(cc2-c2sc4ccccc4c2C1(C)c1ccccc1)C(c1ccc(S(=O)(=O)O)cc1S(=O)(=O)[O-])=c1cc2c(cc1O3)=[N+](C)C(C)(c1ccccc1)c1c-2sc2ccccc12	CS(C)=O	617	588
CN1c2cc3c(cc2-c2c(n(C)c4ccccc24)C1(C)c1ccccc1)C(c1ccc(S(=O)(=O)O)cc1S(=O)(=O)[O-])=c1cc2c(cc1O3)=[N+](C)C(C)(c1ccccc1)c1c-2c2ccccc2n1C	CO	634	604
CCN=c1cc2oc3cc(NCC)c(C)cc3c(-c3ccccc3C(=O)OC)c-2cc1C	CCO	530	493
CCN(CC)c1ccc2c(-c3ccccc3C(=O)[O-])c3ccc(=[N+](CC)CC)cc-3oc2c1	CCO	553	515
CCN(CC)c1ccc2c(-c3ccccc3C(=O)[O-])c3ccc(=[N+](CC)CC)cc-3oc2c1	O	554	532
O=C([O-])c1ccccc1C1=c2cc3c4c(c2Oc2c1cc1c5c2CCCN5CCC1)CCC[N+]=4CCC3	CCO	574	555
CC[N+](CC)=c1ccc2c(-c3ccccc3C(=O)O)c3cc4c(cc3oc-2c1)N1CCCC1CN4C	O	575	568
CCNc1cc2c(cc1C)C(c1ccccc1C(=O)O)c1cc(C)c(NCC)cc1O2	O	521	517
CCNc1cc2oc3cc(=[N+](CC)CC)ccc-3c(-c3ccccc3C(=O)[O-])c2cc1C	O	539	524
O=C(O)c1ccccc1C1c2cc3c(cc2Oc2cc4c(cc21)CCCN4)NCCC3	O	538	522

O=C(O)c1ccccc1C1c2cc3c4c(c2Oc2c1cc1c5c2CCCN5CCC1)CCCN4CCC3	O	580	554
CCN(CC)c1ccc2c(c1)Oc1c(cc3c4c1CCCN4CCC3)C2c1ccccc1C(=O)O	O	566	546
CCN=c1cc2oc3cc4c(cc3c(-c3ccccc3C(=O)O)c-2cc1C)N(CC)C1CCCCC1N4CC	CCO	570	561
CN(C)c1cc2oc3cc(=[N+](C)C)ccc-3c(-c3ccccc3C(=O)[O-])c2cc1-c1ccccc1	CO	548	524
Cc1ccccc1-c1c2ccc(=[N+](C)C)cc-2oc2cc(N(C)C)ccc12	O	550	533
CN(C)c1ccc2c(c1)[B-](O)(O)C1=CC(=[N+](C)C)C=CC1=C2	O	611	592
Cc1ccccc1C1=C2C=CC(=[N+](C)C)C=C2[B-](O)(O)c2cc(N(C)C)ccc21	O	620	607
CN(C)c1ccc2c(c1)C(C)(C)C1=CC(=[N+](C)C)C=CC1=C2c1ccccc1C(=O)[O-]	O	606	591
CC1(C)C2=CC(=[N+]3CCC3)C=CC2=C(c2ccccc2C(=O)[O-])c2ccc(N3CCC3)cc21	O	608	592
CC1(C)C2=CC(=[N+]3CCCC3)C=CC2=C(c2ccccc2C(=O)[O-])c2ccc(N3CCCC3)cc21	O	613	600
CN(C)c1ccc2c(c1)C(C)(C)C1=CC(=[N+](C)C)C=CC1=C2C#Cc1ccccc1	O	680	649
CN=C1C=CC2=C(c3cc(C(=O)O)ccc3C(=O)O)c3ccc(NC)cc3C(C)(C)C2=C1	O	582	574
CN(C)c1ccc2c(c1)C(C)(C)C1=CC(=[N+](C)C)C=CC1=C2c1cc(C(=O)O)ccc1C(=O)[O-]	O	609	596
CN(C)c1ccc2c(c1)C(C)(C)C1=CC(=[N+](C)C)C(F)=CC1=C2c1cc(C(=O)O)ccc1C(=O)[O-]	O	617	614
CN(C)c1cc2c(cc1F)C(c1cc(C(=O)O)ccc1C(=O)[O-])=C1C=C(F)C(=[N+](C)C)C=C1C2(C)C	O	628	611
CC1(C)C2=CC(=NCC(F)(F)F)C=CC2=C(c2cc(C(=O)O)ccc2C(=O)O)c2ccc(NCC(F)(F)F)cc21	O	561	563
CC1(C)C2=CC(=NCC(F)(F)CO)C=CC2=C(c2cc(C(=O)O)ccc2C(=O)O)c2ccc(NCC(F)(F)CO)cc21	O	571	553
CC1(C)C2=CC(=[N+]3CCCCCC3)C=CC2=C(c2ccccc2C(=O)[O-])c2ccc(N3CCCCCC3)cc21	O	618	594
CC1(C)C2=CC(=[N+]3CC(F)(F)C3)C=CC2=C(c2ccccc2C(=O)[O-])c2ccc(N3CC(F)(F)C3)cc21	O	585	576
Cc1ccccc1C1=c2cc3c(cc2[Si](C)(C)c2cc4c(cc21)CCN4c1ccccc1)=[N+](c1ccccc1)CC3	CO	731	678
CCN(CC)c1ccc2c(c1)[Si](C)(C)C1=CC(=[N+](CC)CC)C=CC1=C2c1ccccc1C(=O)[O-]	O	650	623
CN(C)c1ccc2c(c1)[Si](C)(C)C1=CC(=[N+](C)C)C=CC1=C2c1ccccc1C(=O)[O-]	O	643	618

C[Si]1(C)C2=CC(=[N+]3CC(O)C3)C=CC2=C(c2ccccc2C(=O)[O-])c2ccc(N3CC(O)C3)cc21	O	641	609
C[Si]1(C)C2=CC(=[N+]3CCCCCC3)C=CC2=C(c2ccccc2C(=O)[O-])c2ccc(N3CCCCCC3)cc21	O	657	628
Cc1ccccc1C1=C2C=CC(=[N+]3Cc4ccccc4C3)C=C2[Si](C)(C)c2cc(N3Cc4ccccc4C3)ccc21	CO	779	717
CN(C)c1cc2c(cc1F)C(c1cc(C(=O)O)ccc1C(=O)[O-])=C1C=C(F)C(=[N+](C)C)C=C1[Si]2(C)C	O	670	639
C[Si]1(C)C2=CC(=[N+]3CC(F)C3)C=CC2=C(c2ccccc2C(=O)[O-])c2ccc(N3CC(F)C3)cc21	O	635	624
C[Si]1(C)C2=CC(=[N+]3CCC3)C(F)=CC2=C(c2c(F)c(F)c(F)c(F)c2C(=O)[O-])c2cc(F)c(N3CCC3)cc21	O	695	663
CCOC(=O)CSc1c(F)c(F)c(C(=O)[O-])c(C2=C3C=CC(=[N+]4CCC4)C=C3[Si](C)(C)c3cc(N4CCC4)ccc32)c1F	O	668	660
C[Si]1(C)C2=CC(=[N+]3CCC3)C=CC2=C(c2c(F)c(SCC(=O)O)c(F)c(F)c2C(=O)[O-])c2ccc(N3CCC3)cc21	O	667	662
C[Si]1(C)C2=CC(=[N+]3CCC3)C=CC2=C(c2c(Cl)c(Cl)c(Cl)c(Cl)c2C(=O)[O-])c2ccc(N3CCC3)cc21	O	674	661
CN(C)c1ccc2c(c1)[Si](C)(C)C1=CC(=[N+](C)C)C=CC1=C2c1c(F)c(F)c(F)c(F)c1C(=O)[O-]	O	667	648
C[Si]1(C)C2=CC(=[N+]3CCCCCC3)C=CC2=C(c2c(F)c(F)c(F)c(F)c2C(=O)[O-])c2ccc(N3CCCCCC3)cc21	O	683	656
CCSc1c(F)c(F)c(C(=O)OC(C)CCO)c(C2=C3C=CC(=[N+](C)C)C=C3[Si](C)(C)c3cc(N(C)C)ccc32)c1F	O	662	660
CN(C)c1ccc2c(c1)[Si](C)(C)C1=CC(=[N+](C)C)C=CC1=C2C#Cc1ccccc1	O	712	674
CN(C)c1ccc2c(c1)[Si](C)(C)C1=CC(=[N+](C)C)C=CC1=C2c1cc(C(=O)O)sc1C(=O)[O-]	O	650	633
Cc1ccc(C)c(C2=C3C=CC(=[N+]4CCC4)C=C3[Si](C)(C)c3cc(N4CCC4)ccc32)c1C(=O)[O-]	O	649	635
C[Si]1(C)C2=CC(=[N+]3CCC3)C=CC2=C(c2ccsc2C(=O)[O-])c2ccc(N3CCC3)cc21	O	650	637
C[Si]1(C)C2=CC(=[N+]3CCC3)C=CC2=C(CCC(=O)[O-])c2ccc(N3CCC3)cc21	O	652	633
C[Si]1(C)C2=CC(=[N+]3CCC3)C=CC2=C(c2ccccc2)c2ccc(N3CCC3)cc21	O	648	628
Cc1ccccc1C1=C2C=CC(=[N+]3CCC3)C=C2[Si](C)(C)c2cc(N3CCC3)ccc21	O	649	636

Cc1cccc(C)c1C1=C2C=CC(=[N+]3CCC3)C=C2[Si](C)(C)c2cc(N3CCC3)ccc21	O	651	640
COc1ccc(C2=C3C=CC(=[N+]4CCC4)C=C3[Si](C)(C)c3cc(N4CCC4)ccc32)c(C)c1	O	649	631
C[Si]1(C)C2=CC(=[N+]3CCC3)C=CC2=C(c2ccccc2O)c2ccc(N3CCC3)cc21	O	651	627
Cc1cnccc1C1=C2C=CC(=[N+]3CCC3)C=C2[Si](C)(C)c2cc(N3CCC3)ccc21	O	656	623
C[Si]1(C)C2=CC(=[N+]3CCC3)C=CC2=Cc2ccc(N3CCC3)cc21	O	636	623
C[Si]1(C)C2=CC(=[N+]3CCC3)C=CC2=C(C(=O)O)c2ccc(N3CCC3)cc21	O	641	615
CC(C)[Si]1(C(C)C)C2=CC(=[N+](C)C)C=CC2=Cc2ccc(N(C)C)cc21	O	632	607
CN(C)c1ccc2c(c1)[Si](c1ccccc1)(c1ccccc1)C1=CC(=[N+](C)C)C=CC1=C2	O	649	632
CC[Si]1(CCC(C)(C)C)C2=CC(=[N+](C)C)C=CC2=Cc2ccc(N(C)C)cc21	O	637	616
Cc1ccccc1C1=C2C=CC(=[N+](C)C)C=C2[Si](O)(O)c2cc(N(C)C)ccc21	O	663	631
CN(C)c1ccc2c(c1)[Ge](C)(C)C1=CC(=[N+](C)C)C=CC1=C2c1ccccc1C(=O)[O-]	O	634	605
c1ccc(C2=c3cc4c5c(c3Sc3c2cc2c6c3CCCN6CCC2)CCC[N+]=5CCC4)cc1	CO	594	571
CN1CCC(C)(C)c2cc3c(cc21)Sc1cc2c(cc1=C3c1ccccc1)C(C)(C)CC[N+]=2C	CO	579	551
C[N+]1=c2cc3c(cc2C(C)(C)CC1)=C(c1ccccc1)c1cc2c4c(c1S3)CCCN4CCC2	CO	587	555
CN(C)c1ccc2c(-c3ccccc3)c3ccc4cc(=[N+](C)C)ccc4c3sc2c1	CO	641	582
c1ccc(C2=c3cc4c5c(c3[Se]c3c2cc2c6c3CCCN6CCC2)CCC[N+]=5CCC4)cc1	CO	604	564
CN1CCC(C)(C)c2cc3c(cc21)[Se]c1cc2c(cc1=C3c1ccccc1)C(C)(C)CC[N+]=2C	CO	590	548
C[N+]1=c2cc3c(cc2C(C)(C)CC1)=C(c1ccccc1)c1cc2c4c(c1[Se]3)CCCN4CCC2	CO	597	564
CN(C)c1ccc2c(-c3ccccc3)c3ccc(=[N+](C)C)cc-3[te]c2c1	CO	597	552
c1ccc(C2=c3cc4c5c(c3[Te]c3c2cc2c6c3CCCN6CCC2)CCC[N+]=5CCC4)cc1	CO	617	562
CN1CCC(C)(C)c2cc3c(cc21)[Te]c1cc2c(cc1=C3c1ccccc1)C(C)(C)CC[N+]=2C	CO	607	558
C[N+]1=c2cc3c(cc2C(C)(C)CC1)=C(c1ccccc1)c1cc2c4c(c1[Te]3)CCCN4CCC2	CO	610	574
Cc1cc(C)c(C2=c3cc4c5c(c3[Te]c3c2cc2c6c3CCCN6CCC2)CCC[N+]=5CCC4)c(C)c1	CO	617	576
Cc1cc(C)c(C2=c3cc4c(cc3[Te]c3cc5c(cc32)C(C)(C)CCN5C)=[N+](C)CCC4(C)C)c(C)c1	CO	606	564
CN(C)c1ccc2c(c1)S(=O)(=O)C1=CC(=[N+](C)C)C=CC1=C2CCCCCCCNC(=O)OC(C)(C)C	O	509	479

COC(=O)c1ccccc1-c1c2ccc(N)cc2oc2c1ccc1cccc(=O)c12	O	542	533
COC(=O)c1ccccc1-c1c2ccc(N)cc2oc2c1ccc1cccc(=N)c12	O	576	563

Table S5. Results of per-compound prediction in external validation of D_{emi} set.

SMILES	Solvent (SMILES)	λ_{emi}-Exp.	λ_{emi}-Pred.
C=CCN1C(=O)c2ccccc2C2(c3cc(C)c(NCC)cc3Oc3cc(NCC)c(C)cc32)N1CC=C	O	550	542
CCN(CC)c1ccc2c(c1)Oc1cc(N(CC)CC)ccc1C21c2ccccc2C(=O)N1N	O	543	543
CCN(CC)c1ccc2c(-c3ccccc3C(=O)OC3CC(C)(C)N([O-])CC3(C)C)c3ccc(=[N+](CC)CC)cc-3oc2c1	CC#N	588	561
CCc1c(C)[nH]c(C2=C3C=CC(=[N+](C)C)C=C3[Si](C)(C)c3cc(N(C)C)ccc32)c1C	O	680	642
CC[N+](CC)=C1C=CC2=C(c3ccccc3C(=O)O)C3C=CC(N=Nc4cc(C(C)C)c(O)cc4C)=CC3OC2=C1	O	550	515
CCN(CC)c1ccc2c(-c3ccccc3C(=O)N3CCN(C(=O)c4ccc(O)cc4)CC3)c3ccc(=[N+](CC)CC)cc-3oc2c1	O	590	567
CC[N+](CC)=c1ccc2c(-c3ccccc3C(=O)O)c3ccc(-n4[nH]c(C)cc4=O)cc3oc-2c1	O	579	577
CN(C(=O)OCc1ccc(C=O)c(O)c1)c1ccc2c(c1)Oc1cc(N(C)C(=O)OCc3ccc(C=O)c(O)c3)ccc1C21OC(=O)c2ccccc21	O	543	539
CCN(CC)c1ccc2c(-c3ccccc3C(=O)O)c3c([o+]c2c1)C(=Cc1ccc(NC(=O)C(=O)c2ccc([N+](=O)[O-])cc2)cc1)CCC3	O	730	722
CN(C)c1ccc2c(c1)B(O)C1=CC(=[N+](C)C)C=CC1=C2c1ccccc1CS(=O)(=O)O	O	575	548
CCN(CC)c1ccc2c(c1)Oc1cc(OC(C)=O)ccc1C21OC(=O)c2ccccc21	O	560	557
CCN(CC)c1ccc2c(c1)Oc1cc(N(CC)CC)ccc1C21c2ccccc2C(=O)N1CCNCCN1C(=O)c2cccc3c(/C=C/c4cc[n+](Cc5ccc(B6OC(C)(C)C(C)(C)O6)cc5)cc4)ccc(c23)C1=O	O	470	460
CCN(CC)c1ccc2c(-c3ccccc3C(=O)OCc3c4ccccc4cc4ccccc34)c3ccc(=[N+](CC)CC)cc-3oc2c1	O	590	568
CCN(CC)c1ccc2c(c1)OC1=CC(N(CC)CC)C=CC1=C2c1ccccc1C(=O)N1CCN(S(=O)(=O)c2ccc3c4c5c(ccc6c5c(c3c2)-c2ccc(C)cc2C6=O)C(=O)c2cc(C)ccc2-4)CC1	O	576	540

CC/N=c1/cc2oc3cc(N(CC)Cc4c5ccccc5cc5ccccc45)c(C)cc3c(-c3ccccc3C(=O)CC)c-2cc1C	O	560	582
CC/N=c1/cc2oc3cc(N(CC)Cc4c5ccccc5cc5ccccc45)c(C)cc3c(-c3ccccc3C(=O)CC)c-2cc1C	CN(C=O)C	700	631
COc1c2ccccc2c(OC)c2cc(C3=C4C=CC(=[N+](C)C)C=C4C(C)(OC)c4cc(N(C)C)ccc43)cc12	O	667	629
CCN(CC)c1ccc2c(c1)Oc1cc(N3CCN(C(=O)CN4C(=O)c5cccc6c(OC)ccc(c56)C4=O)CC3)ccc1C21c2ccccc2C(=O)N1NC(=S)Nc1ccccc1	CS(C)=O	477	455
CCN(CC)c1ccc2c(c1)Oc1cc(N3CCN(C(=O)CN4C(=O)c5cccc6c(OC)ccc(c56)C4=O)CC3)ccc1C21c2ccccc2C(=O)N1NC(=S)Nc1ccccc1	O	618	583
CCN(CC)c1ccc2c(c1)Oc1cc(N(CC)CC)ccc1C21c2ccccc2C(=S)N1NC(=O)c1ccc(CN2CCOCC2)cc1	O	592	576
CN(C)c1ccc2c(c1)B(O)c1cc(N(C)C)ccc1C21SCc2ccccc21	O	646	609
CCN(CC)c1ccc2c(c1)Oc1cc(N(CC)CC)ccc1C21c2ccccc2C(=O)N1NC=O	O	580	562
CCN(CC)C1=CC2Oc3cc(N(CC)CC)ccc3C3(c4ccccc4C(=O)N3NC(=O)C3CCC(=O)N3C(=O)C3CCC(=O)N3)C2C=C1	O	580	549
CCOCCOCCOCC[n+]1ccc(C(=O)NN2C(=O)c3ccccc3C23c2ccc(N(CC)CC)cc2Oc2cc4c(cc23)Sc2ccccc2N4CC)cc1	O	730	708
CCN(CC)c1ccc2c(c1)Oc1cc(N(CC)CC)ccc1C21c2ccccc2C(=O)C1(Cl)C#N	O	578	563
C=CC(=O)Oc1ccc2c(CC(=O)N3CCN(c4ccc5c(c4)Oc4cc(N(CC)CC)ccc4C54c5ccccc5C(=O)N4NC(=S)Nc4ccccc4)CC3)cc(=O)oc2c1	CC#N	616	548
CCN(CC)c1ccc2c(c1)Oc1cc(N(CC)CC)ccc1C21c2ccccc2C(=O)N1Nc1ccccc1	O	580	557
CCN(CC)c1ccc2c(-c3ccccc3C(=O)OC)c3cc(/C=N/N(C)C)c(=O)cc-3oc2c1	O	571	568
CCN(CC)c1ccc2c(c1)Oc1c(cc3c4c1CCCN4CCC3)C21c2ccccc2C(=O)N1N	O	585	559
CCN(CC)c1ccc2c(c1)Oc1cc(N3CCN(c4ccc5c6c(cccc46)C(=O)N(CCN(C)C)C5=O)CC3)ccc1C21c2ccccc2C(=O)N1N	O	578	551
CCN(CC)c1ccc2c(c1)Oc1c(ccc3[nH]c4ccccc4c13)C21c2ccccc2C(=O)N1N	O	585	566
CCN(CC)c1ccc2c(c1)Oc1cc(N(CC)CC)ccc1C21c2ccccc2C(=O)N2CCCCN21	O	585	559
CNc1ccc(OC)c(C2=C3C=CC(=[N+](C)C)C=C3[Si](C)(C)c3cc(N(C)C)ccc32)c1	O	672	640

CCN(CC)c1ccc2c(c1)Oc1cc(N(CC)CC)ccc1C21c2ccccc2C(=O)N1NC(=O)CN1CCN(C)CC1	O	600	576
CCN(CC)c1ccc2c(c1)Oc1cc(N(CC)CC)ccc1C21c2ccccc2CN1c1ccccc1N	O	590	579
CCOC(=O)c1ccccc1-c1c2ccc(Nc3ccccc3N)cc2[o+]c2cc(N(CC)CC)ccc12	O	581	575
CCN(CC)c1ccc2c(c1)Oc1cc(N(CC)CC)ccc1C21c2ccccc2C(=O)N1CCN=Cc1ccccc1OCc1ccccn1	O	587	557
C#Cc1ccc(N2C(=O)c3ccccc3C23c2ccc(N(CC)CC)cc2Oc2cc(N(CC)CC)ccc23)c(N)c1	O	580	526
C/C=C/C(=O)Oc1ccc(COC(=O)Nc2ccc3c(c2)Oc2cc(N)ccc2C32OC(=O)c3ccccc32)cc1	O	525	510
CCN(CC)c1ccc2c(c1)Oc1cc(N(CC)CC)ccc1C21c2ccccc2C(=O)N1CC=O	O	584	559
C=CC(=O)NN1C(=O)c2ccccc2C12c1ccc(N(CC)CC)cc1Oc1cc(N(CC)CC)ccc12	O	582	571
CCN(CC)c1ccc2c(c1)Oc1cc(N(CC)CC)ccc1C21c2cc(C(=O)O)ccc2C(=O)N1/N=C/C=O	O	576	559
CCN(CC)c1ccc2c(c1)Oc1cc(N(CC)CC)ccc1C21c2ccccc2C(=O)N1NC(=O)CBr	O	579	568
CC[N+](CC)=c1ccc2c(-c3ccccc3C(=O)N3CCN(c4ccc([N+](=O)[O-])c5nonc45)CC3)c3ccc(N=[N+]=[N-])cc3oc-2c1	O	565	527
CCN(CC)c1ccc2c(c1)Oc1cc(N3CCN(C(=O)CN4C(=O)c5cccc6c(N=[N+]=[N-])ccc(c56)C4=O)CC3)ccc1C21c2ccccc2C(=O)N1N	CN(C=O)C	560	499
C=CC(=O)Oc1ccc2c(CC(=O)N3CCN(c4ccc5c(c4)Oc4cc(N(CC)CC)ccc4C54C5C=CC=CC5C(=O)N4NC(=S)NC4=CCCC=C4)CC3)cc(=O)oc2c1	CC#N	488	469
COc1ccc(Oc2c([N+](=O)[O-])cc(C#Cc3ccc(-c4c5ccc(=[N+](C)C)cc-5oc5cc(N(C)C)ccc45)c(C(=O)[O-])c3)c3nsnc23)cc1	O	590	543
CCN(CC)c1ccc2c(-c3ccccc3C(=O)OCOC(C)=O)c3c([o+]c2c1)-c1ccc(N=[N+]=[N-])cc1CC3	O	630	599
CCN(CC)c1ccc2c(-c3ccccc3C=O)c3ccc(=[N+](CC)CC)cc-3oc2c1	O	592	565
CCN(CC)c1ccc2c(c1)Oc1cc(NC(=O)OCc3ccc(N=[N+]=[N-])cc3)ccc1C21c2ccccc2C(=O)N1CCN1CCOCC1	O	560	518

C#CC(C)OC(=O)c1ccccc1C1=C2C=CC(N(CC)CC)=CC2OC2=CC(=[N+](CC)CC)C=CC21	CN(C=O)C	590	545
CCN(CC)C1=C(OC2=CCC([N+](=O)[O-])C=C2[N+](=O)[O-])C(/C=N/N2C(=O)C3=CC=CCC3C23C2=C(CC(N(CC)CC)C=C2)OC2=CC(=[N+](CC)CC)C=CC23)=CCC1	O	770	754
CN(C)c1ccc2c(c1)S(=O)(=O)C1=CC(=[N+](C)C)C=CC1=C2CCCCCCCNC(=O)OC(C)(C)C	O	647	618
COC(=O)c1ccccc1-c1c2ccc(=N)cc-2oc2c1ccc1cccc(O)c12	O	604	596
COC(=O)c1ccccc1-c1c2ccc(=N)cc-2oc2c1ccc1cccc(N)c12	O	760	682
O=C(O)c1ccccc1-c1c2cc3c4c(c2[o+]c2c5c6c(cc12)CCCN6CCC5)CCCN4CCC3	CS(C)=O	567	554
CN1c2cc3c(cc2-c2sc4ccccc4c2C1(C)C)C(c1ccc(S(=O)(=O)[O-])cc1S(=O)(=O)O)=c1cc2c(cc1O3)=[N+](C)C(C)(C)c1c-2sc2ccccc12	CO	616	584
CN1c2cc3c(cc2-c2sc4ccccc4c2C1(C)c1ccccc1)C(c1ccc(S(=O)(=O)O)cc1S(=O)(=O)[O-])=c1cc2c(cc1O3)=[N+](C)C(C)(c1ccccc1)c1c-2sc2ccccc12	CS(C)=O	617	588
CN1c2cc3c(cc2-c2c(n(C)c4ccccc24)C1(C)c1ccccc1)C(c1ccc(S(=O)(=O)O)cc1S(=O)(=O)[O-])=c1cc2c(cc1O3)=[N+](C)C(C)(c1ccccc1)c1c-2c2ccccc2n1C	CO	634	604
CCN=c1cc2oc3cc(NCC)c(C)cc3c(-c3ccccc3C(=O)OC)c-2cc1C	CCO	530	493
CCN(CC)c1ccc2c(-c3ccccc3C(=O)[O-])c3ccc(=[N+](CC)CC)cc-3oc2c1	CCO	553	515
CCN(CC)c1ccc2c(-c3ccccc3C(=O)[O-])c3ccc(=[N+](CC)CC)cc-3oc2c1	O	554	532
O=C([O-])c1ccccc1C1=c2cc3c4c(c2Oc2c1cc1c5c2CCCN5CCC1)CCC[N+]=4CCC3	CCO	574	555
CC[N+](CC)=c1ccc2c(-c3ccccc3C(=O)O)c3cc4c(cc3oc-2c1)N1CCCC1CN4C	O	575	568
CCNc1cc2c(cc1C)C(c1ccccc1C(=O)O)c1cc(C)c(NCC)cc1O2	O	521	517
CCNc1cc2oc3cc(=[N+](CC)CC)ccc-3c(-c3ccccc3C(=O)[O-])c2cc1C	O	539	524
O=C(O)c1ccccc1C1c2cc3c(cc2Oc2cc4c(cc21)CCCN4)NCCC3	O	538	522

O=C(O)c1ccccc1C1c2cc3c4c(c2Oc2c1cc1c5c2CCCN5CCC1)CCCN4CCC3	O	580	554
CCN(CC)c1ccc2c(c1)Oc1c(cc3c4c1CCCN4CCC3)C2c1ccccc1C(=O)O	O	566	546
CCN=c1cc2oc3cc4c(cc3c(-c3ccccc3C(=O)O)c-2cc1C)N(CC)C1CCCCC1N4CC	CCO	570	561
CN(C)c1cc2oc3cc(=[N+](C)C)ccc-3c(-c3ccccc3C(=O)[O-])c2cc1-c1ccccc1	CO	548	524
Cc1ccccc1-c1c2ccc(=[N+](C)C)cc-2oc2cc(N(C)C)ccc12	O	550	533
CN(C)c1ccc2c(c1)[B-](O)(O)C1=CC(=[N+](C)C)C=CC1=C2	O	611	592
Cc1ccccc1C1=C2C=CC(=[N+](C)C)C=C2[B-](O)(O)c2cc(N(C)C)ccc21	O	620	607
CN(C)c1ccc2c(c1)C(C)(C)C1=CC(=[N+](C)C)C=CC1=C2c1ccccc1C(=O)[O-]	O	606	591
CC1(C)C2=CC(=[N+]3CCC3)C=CC2=C(c2ccccc2C(=O)[O-])c2ccc(N3CCC3)cc21	O	608	592
CC1(C)C2=CC(=[N+]3CCCC3)C=CC2=C(c2ccccc2C(=O)[O-])c2ccc(N3CCCC3)cc21	O	613	600
CN(C)c1ccc2c(c1)C(C)(C)C1=CC(=[N+](C)C)C=CC1=C2C#Cc1ccccc1	O	680	649
CN=C1C=CC2=C(c3cc(C(=O)O)ccc3C(=O)O)c3ccc(NC)cc3C(C)(C)C2=C1	O	582	574
CN(C)c1ccc2c(c1)C(C)(C)C1=CC(=[N+](C)C)C=CC1=C2c1cc(C(=O)O)ccc1C(=O)[O-]	O	609	596
CN(C)c1ccc2c(c1)C(C)(C)C1=CC(=[N+](C)C)C(F)=CC1=C2c1cc(C(=O)O)ccc1C(=O)[O-]	O	617	614
CN(C)c1cc2c(cc1F)C(c1cc(C(=O)O)ccc1C(=O)[O-])=C1C=C(F)C(=[N+](C)C)C=C1C2(C)C	O	628	611
CC1(C)C2=CC(=NCC(F)(F)F)C=CC2=C(c2cc(C(=O)O)ccc2C(=O)O)c2ccc(NCC(F)(F)F)cc21	O	561	563
CC1(C)C2=CC(=NCC(F)(F)CO)C=CC2=C(c2cc(C(=O)O)ccc2C(=O)O)c2ccc(NCC(F)(F)CO)cc21	O	571	553
CC1(C)C2=CC(=[N+]3CCCCCC3)C=CC2=C(c2ccccc2C(=O)[O-])c2ccc(N3CCCCCC3)cc21	O	618	594
CC1(C)C2=CC(=[N+]3CC(F)(F)C3)C=CC2=C(c2ccccc2C(=O)[O-])c2ccc(N3CC(F)(F)C3)cc21	O	585	576
Cc1ccccc1C1=c2cc3c(cc2[Si](C)(C)c2cc4c(cc21)CCN4c1ccccc1)=[N+](c1ccccc1)CC3	CO	731	678
CCN(CC)c1ccc2c(c1)[Si](C)(C)C1=CC(=[N+](CC)CC)C=CC1=C2c1ccccc1C(=O)[O-]	O	650	623
CN(C)c1ccc2c(c1)[Si](C)(C)C1=CC(=[N+](C)C)C=CC1=C2c1ccccc1C(=O)[O-]	O	643	618

C[Si]1(C)C2=CC(=[N+]3CC(O)C3)C=CC2=C(c2ccccc2C(=O)[O-])c2ccc(N3CC(O)C3)cc21	O	641	609
C[Si]1(C)C2=CC(=[N+]3CCCCCC3)C=CC2=C(c2ccccc2C(=O)[O-])c2ccc(N3CCCCCC3)cc21	O	657	628
Cc1ccccc1C1=C2C=CC(=[N+]3Cc4ccccc4C3)C=C2[Si](C)(C)c2cc(N3Cc4ccccc4C3)ccc21	CO	779	717
CN(C)c1cc2c(cc1F)C(c1cc(C(=O)O)ccc1C(=O)[O-])=C1C=C(F)C(=[N+](C)C)C=C1[Si]2(C)C	O	670	639
C[Si]1(C)C2=CC(=[N+]3CC(F)C3)C=CC2=C(c2ccccc2C(=O)[O-])c2ccc(N3CC(F)C3)cc21	O	635	624
C[Si]1(C)C2=CC(=[N+]3CCC3)C(F)=CC2=C(c2c(F)c(F)c(F)c(F)c2C(=O)[O-])c2cc(F)c(N3CCC3)cc21	O	695	663
CCOC(=O)CSc1c(F)c(F)c(C(=O)[O-])c(C2=C3C=CC(=[N+]4CCC4)C=C3[Si](C)(C)c3cc(N4CCC4)ccc32)c1F	O	668	660
C[Si]1(C)C2=CC(=[N+]3CCC3)C=CC2=C(c2c(F)c(SCC(=O)O)c(F)c(F)c2C(=O)[O-])c2ccc(N3CCC3)cc21	O	667	662
C[Si]1(C)C2=CC(=[N+]3CCC3)C=CC2=C(c2c(Cl)c(Cl)c(Cl)c(Cl)c2C(=O)[O-])c2ccc(N3CCC3)cc21	O	674	661
CN(C)c1ccc2c(c1)[Si](C)(C)C1=CC(=[N+](C)C)C=CC1=C2c1c(F)c(F)c(F)c(F)c1C(=O)[O-]	O	667	648
C[Si]1(C)C2=CC(=[N+]3CCCCCC3)C=CC2=C(c2c(F)c(F)c(F)c(F)c2C(=O)[O-])c2ccc(N3CCCCCC3)cc21	O	683	656
CCSc1c(F)c(F)c(C(=O)OC(C)CCO)c(C2=C3C=CC(=[N+](C)C)C=C3[Si](C)(C)c3cc(N(C)C)ccc32)c1F	O	662	660
CN(C)c1ccc2c(c1)[Si](C)(C)C1=CC(=[N+](C)C)C=CC1=C2C#Cc1ccccc1	O	712	674
CN(C)c1ccc2c(c1)[Si](C)(C)C1=CC(=[N+](C)C)C=CC1=C2c1cc(C(=O)O)sc1C(=O)[O-]	O	650	633
Cc1ccc(C)c(C2=C3C=CC(=[N+]4CCC4)C=C3[Si](C)(C)c3cc(N4CCC4)ccc32)c1C(=O)[O-]	O	649	635
C[Si]1(C)C2=CC(=[N+]3CCC3)C=CC2=C(c2ccsc2C(=O)[O-])c2ccc(N3CCC3)cc21	O	650	637
C[Si]1(C)C2=CC(=[N+]3CCC3)C=CC2=C(CCC(=O)[O-])c2ccc(N3CCC3)cc21	O	652	633
C[Si]1(C)C2=CC(=[N+]3CCC3)C=CC2=C(c2ccccc2)c2ccc(N3CCC3)cc21	O	648	628
Cc1ccccc1C1=C2C=CC(=[N+]3CCC3)C=C2[Si](C)(C)c2cc(N3CCC3)ccc21	O	649	636

Cc1cccc(C)c1C1=C2C=CC(=[N+]3CCC3)C=C2[Si](C)(C)c2cc(N3CCC3)ccc21	O	651	640
COc1ccc(C2=C3C=CC(=[N+]4CCC4)C=C3[Si](C)(C)c3cc(N4CCC4)ccc32)c(C)c1	O	649	631
C[Si]1(C)C2=CC(=[N+]3CCC3)C=CC2=C(c2ccccc2O)c2ccc(N3CCC3)cc21	O	651	627
Cc1cnccc1C1=C2C=CC(=[N+]3CCC3)C=C2[Si](C)(C)c2cc(N3CCC3)ccc21	O	656	623
C[Si]1(C)C2=CC(=[N+]3CCC3)C=CC2=Cc2ccc(N3CCC3)cc21	O	636	623
C[Si]1(C)C2=CC(=[N+]3CCC3)C=CC2=C(C(=O)O)c2ccc(N3CCC3)cc21	O	641	615
CC(C)[Si]1(C(C)C)C2=CC(=[N+](C)C)C=CC2=Cc2ccc(N(C)C)cc21	O	632	607
CN(C)c1ccc2c(c1)[Si](c1ccccc1)(c1ccccc1)C1=CC(=[N+](C)C)C=CC1=C2	O	649	632
CC[Si]1(CCC(C)(C)C)C2=CC(=[N+](C)C)C=CC2=Cc2ccc(N(C)C)cc21	O	637	616
Cc1ccccc1C1=C2C=CC(=[N+](C)C)C=C2[Si](O)(O)c2cc(N(C)C)ccc21	O	663	631
CN(C)c1ccc2c(c1)[Ge](C)(C)C1=CC(=[N+](C)C)C=CC1=C2c1ccccc1C(=O)[O-]	O	634	605
c1ccc(C2=c3cc4c5c(c3Sc3c2cc2c6c3CCCN6CCC2)CCC[N+]=5CCC4)cc1	CO	594	571
CN1CCC(C)(C)c2cc3c(cc21)Sc1cc2c(cc1=C3c1ccccc1)C(C)(C)CC[N+]=2C	CO	579	551
C[N+]1=c2cc3c(cc2C(C)(C)CC1)=C(c1ccccc1)c1cc2c4c(c1S3)CCCN4CCC2	CO	587	555
CN(C)c1ccc2c(-c3ccccc3)c3ccc4cc(=[N+](C)C)ccc4c3sc2c1	CO	641	582
c1ccc(C2=c3cc4c5c(c3[Se]c3c2cc2c6c3CCCN6CCC2)CCC[N+]=5CCC4)cc1	CO	604	564
CN1CCC(C)(C)c2cc3c(cc21)[Se]c1cc2c(cc1=C3c1ccccc1)C(C)(C)CC[N+]=2C	CO	590	548
C[N+]1=c2cc3c(cc2C(C)(C)CC1)=C(c1ccccc1)c1cc2c4c(c1[Se]3)CCCN4CCC2	CO	597	564
CN(C)c1ccc2c(-c3ccccc3)c3ccc(=[N+](C)C)cc-3[te]c2c1	CO	597	552
c1ccc(C2=c3cc4c5c(c3[Te]c3c2cc2c6c3CCCN6CCC2)CCC[N+]=5CCC4)cc1	CO	617	562
CN1CCC(C)(C)c2cc3c(cc21)[Te]c1cc2c(cc1=C3c1ccccc1)C(C)(C)CC[N+]=2C	CO	607	558
C[N+]1=c2cc3c(cc2C(C)(C)CC1)=C(c1ccccc1)c1cc2c4c(c1[Te]3)CCCN4CCC2	CO	610	574
Cc1cc(C)c(C2=c3cc4c5c(c3[Te]c3c2cc2c6c3CCCN6CCC2)CCC[N+]=5CCC4)c(C)c1	CO	617	576
Cc1cc(C)c(C2=c3cc4c(cc3[Te]c3cc5c(cc32)C(C)(C)CCN5C)=[N+](C)CCC4(C)C)c(C)c1	CO	606	564
CN(C)c1ccc2c(c1)S(=O)(=O)C1=CC(=[N+](C)C)C=CC1=C2CCCCCCCNC(=O)OC(C)(C)C	O	509	479

COC(=O)c1ccccc1-c1c2ccc(N)cc2oc2c1ccc1cccc(=O)c12	O	542	533
COC(=O)c1ccccc1-c1c2ccc(N)cc2oc2c1ccc1cccc(=N)c12	O	576	563

Table S6. Results of per-compound prediction in external validation of D_{32} set, with all solvents of molecules are H_2O.

SMILES	λabs-Exp.	λabs-Pred.	λemi-Exp.	λemi-Pred.
CCCNC(C1=CC=C(N2CCCC2)C=C1[Si](C)(C)C3=C/4)=C3C=CC4=[N+]5CCCC/5	465	460	622	596
CCCNC1=C2C=C3CC[N+](C)=C3C=C2[Si](C)(C)C4=CC5=C(CCN5C)C=C14	464	472	655	638
CCCNC(C1=CC(C(C)=CC(C)(N2C)C)=C2C=C1[Si](C)(C)C3=CC4=[N+]5C)=C3C=C4C(C)=CC5(C)C	508	496	660	648
CCCNC(C1=CC=C(N2CCCC2)C=C1P3(C4=CC=CC=C4)=O)=C(C3=C/5)C=CC5=[N+]6CCCC/6	499	505	630	622
CCCNC(C1=CC(CCN2C)=C2C=C13)=C4C=C5CC[N+](C)=C5C=C4P3(C6=CC=CC=C6)=O	517	507	660	642
CCCNC(C1=CC(C(C)=CC(C)(N2C)C)=C2C=C1P3(C4=CC=CC=C4)=O)=C(C3=CC5=[N+]6C)C=C5C(C)=CC6(C)C	563	544	675	662
CCCNC(C1=CC=C(N2CCCC2)C=C1S3(=O)=O)=C(C3=C/4)C=CC4=[N+]5CCCC/5	514	502	650	626
CCCNC(C1=CC(CCCN2C)=C2C=C13)=C4C=C5CCC[N+](C)=C5C=C4S3(=O)=O	522	529	675	678
O=S1(C2=C3C4=C(C=C2C(NCCC)=C5C=C6CCC[N+]7=C6C(CCC7)=C51)CCCN4CCC3)=O	542	539	705	682
CN1CCC2=C1C=C3C(C(NCCC)=C4C=C5CC[N+](C)=C5C=C4S3(=O)=O)=C2	544	532	725	684
O=S1(C2=CC(N(C)C)=CC=C2C(NCCC)=C3C1=C/C(C=C3)=[N+](C)/C)=O	505	498	638	631
CCCNC1=C2C(C3=CC(N(CC)CC)=CC=C31)=C/C(C=C2)=[N+](CC)/CC	566	555	710	686
CN(C)C1=CC2=C(C=C1)C(NCCC)=C3C2=C/C(C=C3)=[N+](C)\C	556	544	698	681
CCCNC1=C2C(C3=C1C=CC(N4CCC4)=C3)=C/C(C=C2)=[N+]5CCC\5	552	543	709	684
CCCNC1=C2C(C3=C1C=CC(N4CCCC4)=C3)=C/C(C=C2)=[N+]5CCCC\5	568	560	714	696

CC1(C)C=C(C)C2=CC3=C(C4=CC=C(N)C=C4[Si](C)(C)C3=CC2=[N+]1C)C5=CC=CC=C5	662	637	700	663
CC(C1=CC2=C(C3=CC=C(NC(C)=O)C=C3[Si](C)(C)C2=CC1=[N+]4C)C5=CC=CC=C5)=CC4(C)C	548	522	690	668
COC1=C(C(C2=CC=C(N)C=C2[Si]3(C)C)=C4C=C(C(C=C43)=[N+]5C)C(C)=CC5(C)C)C=CC=C1	665	637	703	669
CC(C1=CC2=C(C3=CC=C(NC(C)=O)C=C3[Si](C)(C)C2=CC1=[N+]4C)C5=C(OC)C=CC=C5)=CC4(C)C	550	520	700	670
COC1=C(C(C2=CC=C(N)C=C2[Si]3(C)C)=C4C=C(C(C=C43)=[N+]5C)C(C)=CC5(C)C)C(OC)=CC=C1	666	627	707	666
CC(C1=CC2=C(C3=CC=C(NC(C)=O)C=C3[Si](C)(C)C2=CC1=[N+]4C)C5=C(OC)C=CC=C5OC)=CC4(C)C	552	530	703	676
NC1=CC=C(C2(C3=C(CO2)C=CC=C3)C4=CC5=C(N(C)C(C)(C)C=C5C)C=C4[Si]6(C)C)C6=C1	696	643	700	674
NC1=CC=C(C2(C3=C(CO2)C=CC=C3)C4=CC=C(N5CCCC5)C=C4[Si]6(C)C)C6=C1	624	617	644	633
CN(C(/C(C1=O)=C2C3=CC(C=CC4(C)C)=C(N4C)C=C3[Si](C)(C)C5=CC6=C(C=C\25)C=CC(C)(N6C)C)=O)C(N1C)=O	728	676	765	721
CC(C1=C2C=C3C(/C(C4=CC(C(C)=CC5(C)C)=C(N5C)C=C4[Si]3(C)C)=C(C#N)\C#N)=C1)=CC(C)(N2C)C	550	531	650	641
CC(C1=C2C=C3C(/C(C4=CC(C(C)=CC5(C)C)=C(N5C)C=C4[Si]3(C)C)=C6/C(C7=C(C\6=C(C#N)/C#N)CCC=C7)=C(C#N)/C#N)=C1)=CC(C)(N2C)C	811	744	860	799
CC(C(C=C1/C(C2=CC(C(C)=CC3(C)C)=C(N3C)C=C2[Si](C)(C)C1=C4)=C5C(C6=C(C\5=O)C=CC=C6)=O)=C4N7C)=CC7(C)C	723	680	742	709
CN(C)C1=CC=C2C(OC(C=C(N(C)C)C=C3)=C3/C2=N/C4=CC=CC=C4)=C1	600	552	594	583
CN(C)C1=CC=C2C(C(C)(C)C(C=C(N(C)C)C=C3)=C3/C2=N/C4=CC=CC=C4)=C1	630	598	650	639
CN(C)C1=CC=C2C([Si](C)(C)C(C=C(N(C)C)C=C3)=C3/C2=N/C4=CC=CC=C4)=C1	664	647	691	671
CN(C)C1=CC=C2C(P(C(C=C(N(C)C)C=C3)=C3/C2=N/C4=CC=CC=C4)(C5=CC=CC=C5)=O)=C1	712	671	745	719

CN(C)C1=CC=C2C(S(C(C=C(N(C)C)C=C3)=C3/C2=N/C4=CC=CC=C4)(=O)=O)=C1	733	669	755	742

Table S7. The labeled λ_{abs} of rhodamine derivatives.

SMILES /Rhodamine	solvent /SMILES	λ_{abs}	SMILES /Rhodamine	solvent /SMILES	λ_{abs}	SMILES /Rhodamine	solvent /SMILES	λ_{abs}
N=c1ccc2c(-c3ccccc3CO)c3ccc(N)cc3oc-2c1	O	501	CCN(CC)c1ccc2c(-c3ccccc3C(=O)NCCNc3ncc(C(=O)OC)c(Nc4ccccc4)n3)c3ccc(=[N+](CC)CC)cc-3oc2c1	ClCCl	303	Cc1ccccc1C1=c2cc3c(cc2[Si](C)(C)c2cc4c(cc21)CCN4c1ccccc1)=[N+](c1ccccc1)CC3	CO	731
CC(C)CC(N)C(=O)Nc1ccc2c(c1)Oc1cc(N)ccc1C21OCc2ccccc21	O	496	CCN(CC)c1ccc2c(-c3ccccc3C(=O)NCCNc3ncc(C(=O)OC)c(Nc4ccccc4)n3)c3ccc(=[N+](CC)CC)cc-3oc2c1	CC(OCC)=O	310	CCN(CC)c1ccc2c(c1)[Si](C)(C)C1=CC(=[N+](CC)CC)C=CC1=C2c1ccccc1C(=O)[O-]	O	650
CC(=O)Nc1ccc2c(c1)Oc1cc(N)ccc1C21OCc2ccccc21	O	495	CCN(CC)c1ccc2c(-c3ccccc3C(=O)NCCNc3ncc(C(=O)OC)c(Nc4ccccc4)n3)c3ccc(=[N+](CC)CC)cc-3oc2c1	C1CCCO1	309	CN(C)c1ccc2c(c1)[Si](C)(C)C1=CC(=[N+](C)C)C=CC1=C2c1ccccc1C(=O)[O-]	O	643
C[N+](C)=c1ccc2c(-c3ccccc3CO)c3ccc(N)cc3oc-2c1	O	528	CCN(CC)c1ccc2c(-c3ccccc3C(=O)NCCNc3ncc(C(=O)OC)c(Nc4ccccc4)n3)c3ccc(=[N+](CC)CC)cc-3oc2c1	CS(C)=O	311	C[Si]1(C)C2=CC(=[N+]3CC(O)C3)C=CC2=C(c2ccccc2C(=O)[O-])c2ccc(N3CC(O)C3)cc21	O	641
CC(=O)Nc1ccc2c(c1)Oc1cc(N(C)C)ccc1C21OCc2ccccc21	O	499	CCN(CC)c1ccc2c(-c3ccccc3C(=O)NCCNc3ncc(C(=O)OC)	ClCCl	305	C[Si]1(C)C2=CC(=[N+]3CCCCC3)C=CC2=C(c2ccccc2C(=O)[O-	O	657

			c(NCc4ccccc4)n3)c3ccc(=[N+](CC)CC)cc-3oc2c1			])c2ccc(N3CCCCCC3)cc21		
CC[N+](CC)=c1ccc2c(-c3ccccc3CO)c3ccc(N)cc3oc-2c1	O	532	CCN(CC)c1ccc2c(-c3ccccc3C(=O)NCCNc3ncc(C(=O)OC)c(NCc4ccccc4)n3)c3ccc(=[N+](CC)CC)cc-3oc2c1	CC(OCC)=O	305	Cc1ccccc1C1=C2C=CC(=[N+]3Cc4ccccc4C3)C=C2[Si](C)(C)c2cc(N3Cc4ccccc4C3)ccc21	CO	779
CCN(C)c1ccc2c(c1)Oc1cc(NC(C)=O)ccc1C21OCc2ccccc21	O	501	CCN(CC)c1ccc2c(-c3ccccc3C(=O)NCCNc3ncc(C(=O)OC)c(NCc4ccccc4)n3)c3ccc(=[N+](CC)CC)cc-3oc2c1	C1CCCO1	306	CN(C)c1cc2c(cc1F)C(c1cc(C(=O)O)ccc1C(=O)[O-])=C1C=C(F)C(=[N+](C)C)C=C1[Si]2(C)C	O	670
CC(=O)NCC(=O)N1CCCC1C(=O)Nc1ccc2c(c1)Oc1cc(N)ccc1C21OCc2ccccc21	O	496	CCN(CC)c1ccc2c(-c3ccccc3C(=O)NCCNc3ncc(C(=O)OC)c(NCc4ccccc4)n3)c3ccc(=[N+](CC)CC)cc-3oc2c1	CS(C)=O	310	C[Si]1(C)C2=CC(=[N+]3CC(F)C3)C=CC2=C(c2ccccc2C(=O)[O-])c2ccc(N3CC(F)C3)cc21	O	635
Nc1ccc2c(c1)Oc1cc(NC(=O)O[C@@H]3C[C@H](CO)[C@H](O)[C@H](O)[C@H]3O)ccc1C21OCc2ccccc21	O	494	CCN(CC)c1ccc2c(-c3ccccc3C(=O)NCCNc3ncc(C(=O)OC)c(NCCc4ccccc4)n3)c3ccc(=[N+](CC)CC)cc-3oc2c1	ClCCl	306	C[Si]1(C)C2=CC(=[N+]3CCC3)C(F)=CC2=C(c2c(F)c(F)c(F)c(F)c2C(=O)[O-])c2cc(F)c(N3CCC3)cc21	O	695
CC[N+](CC)=c1ccc2c(-c3ccccc3CO)c3ccc([O-])cc3oc-2c1	O	525	CCN(CC)c1ccc2c(-c3ccccc3C(=O)NCCNc3ncc(C(=O)OC)c(NCCc4ccccc4)n3)c3ccc(=[N+](CC)CC)cc-3oc2c1	CC(OCC)=O	309	CCOC(=O)CSc1c(F)c(F)c(C(=O)[O-])c(C2=C3C=CC(=[N+]4CCC4)C=C3[Si](C)(C)c3cc(N4CCC4)ccc32)c1F	O	668
CN(C)c1ccc2c(-	O	552	CCN(CC)c1ccc2c(-	C1CCCO1	307	C[Si]1(C)C2=CC(=[N+]3C	O	667

c3ccccc3CO)c3ccc(=[N+](C)C)cc-3oc2c1			c3ccccc3C(=O)NCCNc3ncc(C(=O)OC)c(NCCc4ccccc4)n3)c3ccc(=[N+](CC)CC)cc-3oc2c1			CC3)C=CC2=C(c2c(F)c(SCC(=O)O)c(F)c(F)c2C(=O)[O-])c2ccc(N3CCC3)cc21		
CN(C)c1ccc2c(-c3c(CO)cccc3COc3ccc(N)cc3)c3ccc(=[N+](C)C)cc-3oc2c1	O	553	CCN(CC)c1ccc2c(-c3ccccc3C(=O)NCCNc3ncc(C(=O)OC)c(NCCc4ccccc4)n3)c3ccc(=[N+](CC)CC)cc-3oc2c1	CS(C)=O	310	C[Si]1(C)C2=CC(=[N+]3CCC3)C=CC2=C(c2c(Cl)c(Cl)c(Cl)c(Cl)c2C(=O)[O-])c2ccc(N3CCC3)cc21	O	674
CN(C)c1ccc2c(-c3c(CO)cccc3COc3ccc(O)cc3)c3ccc(=[N+](C)C)cc-3oc2c1	O	553	CCN(CC)c1ccc2c(-c3ccccc3C(=O)NCCNc3ncc(C(=O)OC)c(NCCc4ccc(OC)c(OC)c4)n3)c3ccc(=[N+](CC)CC)cc-3oc2c1	ClCCl	307	CN(C)c1ccc2c(c1)[Si](C)(C)C1=CC(=[N+](C)C)C=CC1=C2c1c(F)c(F)c(F)c(F)c1C(=O)[O-]	O	667
CCN(CC)c1ccc2c(-c3ccccc3CO)c3ccc(=[N+](CC)CC)cc-3oc2c1	O	559	CCN(CC)c1ccc2c(-c3ccccc3C(=O)NCCNc3ncc(C(=O)OC)c(NCCc4ccc(OC)c(OC)c4)n3)c3ccc(=[N+](CC)CC)cc-3oc2c1	CC(OCC)=O	308	C[Si]1(C)C2=CC(=[N+]3CCCCC3)C=CC2=C(c2c(F)c(F)c(F)c(F)c2C(=O)[O-])c2ccc(N3CCCCC3)cc21	O	683
CCN(CC)c1ccc2c(c1)C=CC(C(=O)NCCC(=O)OCc1ccccc1-c1c3ccc(=[N+](CC)CC)cc-3oc3cc(N(CC)CC)ccc13)O2	O	585	CCN(CC)c1ccc2c(-c3ccccc3C(=O)NCCNc3ncc(C(=O)OC)c(NCCc4ccc(OC)c(OC)c4)n3)c3ccc(=[N+](CC)CC)cc-3oc2c1	C1CCCO1	308	CCSc1c(F)c(F)c(C(=O)OC(C)CCO)c(C2=C3C=CC(=[N+](C)C)C=C3[Si](C)(C)c3cc(N(C)C)ccc32)c1F	O	662
C[N+](C)=c1ccc2c(-c3ccccc3CO)c3cc(F)c(N)cc3oc-2c1	O	532	CCN(CC)c1ccc2c(-c3ccccc3C(=O)NCCNc3ncc(C(=O)OC)	CS(C)=O	309	CN(C)c1ccc2c(c1)[Si](C)(C)C1=CC(=[N+](C)C)C=CC1	O	712

			c(NCCc4ccc(OC)c(OC)c4)n3)c3ccc(=[N+](CC)CC)cc-3oc2c1			=C2C#Cc1ccccc1		
C[N+](C)=c1ccc2c(-c3ccccc3CO)c3cc(Cl)c(N)cc3oc-2c1	O	536	CCN(CC)c1ccc2c(-c3ccccc3C(=O)N3CCN(C)CC3)c3ccc(=[N+](CC)CC)cc-3oc2c1	O	565	CN(C)c1ccc2c(c1)[Si](C)(C)C1=CC(=[N+](C)C)C=CC1=C2c1cc(C(=O)O)sc1C(=O)[O-]	O	650
CC(=O)Nc1cc2c(cc1F)C1(OCc3ccccc31)c1ccc(N(C)C)cc1O2	O	507	CCN(CC)c1ccc2c(-c3ccccc3C(=O)N3CCN(C)CC3)c3ccc(=[N+](CC)CC)cc-3oc2c1	CO	560	Cc1ccc(C)c(C2=C3C=CC(=[N+]4CCC4)C=C3[Si](C)(C)c3cc(N4CCC4)ccc32)c1C(=O)[O-]	O	649
CC(=O)Nc1cc2c(cc1Cl)C1(OCc3ccccc31)c1ccc(N(C)C)cc1O2	O	505	CCN(CC)c1ccc2c(-c3ccccc3C(=O)N3CCN(C)CC3)c3ccc(=[N+](CC)CC)cc-3oc2c1	CCO	563	C[Si]1(C)C2=CC(=[N+]3CCC3)C=CC2=C(c2ccsc2C(=O)[O-])c2ccc(N3CCC3)cc21	O	650
CC[N+](CC)=c1ccc2c(-c3ccccc3CO)c3cc(F)c(N)cc3oc-2c1	O	538	CCN(CC)c1ccc2c(-c3ccccc3C(=O)N3CCN(C)CC3)c3ccc(=[N+](CC)CC)cc-3oc2c1	CCCO	561	C[Si]1(C)C2=CC(=[N+]3CCC3)C=CC2=C(CCC(=O)[O-])c2ccc(N3CCC3)cc21	O	652
CC[N+](CC)=c1ccc2c(-c3ccccc3CO)c3cc(Cl)c(N)cc3oc-2c1	O	541	CCN(CC)c1ccc2c(-c3ccccc3C(=O)N3CCN(C)CC3)c3ccc(=[N+](CC)CC)cc-3oc2c1	CC(O)C	561	C[Si]1(C)C2=CC(=[N+]3CCC3)C=CC2=C(c2ccccc2)c2ccc(N3CCC3)cc21	O	648
CCN(CC)c1ccc2c(c1)Oc1cc(NC(C)=O)c(Cl)cc1C21OCc2ccccc21	O	507	CCN(CC)c1ccc2c(-c3ccccc3C(=O)N3CCN(C)CC3)c3ccc(=[N+](CC)CC)cc-3oc2c1	CC#N	560	Cc1ccccc1C1=C2C=CC(=[N+]3CCC3)C=C2[Si](C)(C)c2cc(N3CCC3)ccc21	O	649
CCN(CC)c1ccc2c(c1)Oc1cc(NC(C)=O)c(F)cc	O	508	CCN(CC)c1ccc2c(-c3ccccc3C(=O)N3CCN(C	CS(C)=O	568	Cc1cccc(C)c1C1=C2C=CC(=[N+]3CCC3)C=C2[Si](C)(	O	651

1C21OCc2ccccc21			)CC3)c3ccc(=[N+](CC)CC)cc-3oc2c1			C)c2cc(N3CCC3)ccc21		
Nc1ccc2c(c1)Oc1c3c4c(cc1=C2c1ccccc1CO)CCC[N+]=4CCC3	O	547	CCN(CC)c1ccc2c(-c3ccccc3C(=O)N3CCN(C)CC3)c3ccc(=[N+](CC)CC)cc-3oc2c1	CN(C=O)C	564	COc1ccc(C2=C3C=CC(=[N+]4CCC4)C=C3[Si](C)(C)c3cc(N4CCC4)ccc32)c(C)c1	O	649
Nc1cc2c(cc1F)C(c1ccccc1CO)=c1cc3c4c(c1O2)CCC[N+]=4CCC3	O	553	CCN(CC)c1ccc2c(-c3ccccc3C(=O)N3CCN(C)CC3)c3ccc(=[N+](CC)CC)cc-3oc2c1	ClCCl	565	C[Si]1(C)C2=CC(=[N+]3CCC3)C=CC2=C(c2ccccc2O)c2ccc(N3CCC3)cc21	O	651
Nc1cc2c(cc1Cl)C(c1ccccc1CO)=c1cc3c4c(c1O2)CCC[N+]=4CCC3	O	555	CCN(CC)c1ccc2c(-c3ccccc3C(=O)N3CCN(C)CC3)c3ccc(=[N+](CC)CC)cc-3oc2c1	ClC(Cl)Cl	561	Cc1cnccc1C1=C2C=CC(=[N+]3CCC3)C=C2[Si](C)(C)c2cc(N3CCC3)ccc21	O	656
OCc1ccccc1-c1c2ccc(=NCC(F)(F)F)cc-2oc2cc(NCC(F)(F)F)ccc12	O	502	CCN(CC)c1ccc2c(-c3ccccc3C(=O)N3CCN(C)CC3)c3ccc(=[N+](CC)CC)cc-3oc2c1	C1COCCO1	562	C[Si]1(C)C2=CC(=[N+]3CCC3)C=CC2=Cc2ccc(N3CCC3)cc21	O	636
O=C(O)c1ccc(-c2c3ccc(=[N+](CC(F)(F)F)CC(F)(F)F)cc-3oc3cc(N(CC(F)(F)F)CC(F)(F)F)ccc23)c(CO)c1	O	507	CCN(CC)CCN(C)C(=O)c1ccccc1-c1c2ccc(=[N+](CC)CC)cc-2oc2cc(N(CC)CC)ccc12	O	566	C[Si]1(C)C2=CC(=[N+]3CCC3)C=CC2=C(C(=O)O)c2ccc(N3CCC3)cc21	O	641
O=C(O)c1ccc(-c2c3ccc(=[N+](CC(F)(F)F)CC(F)(F)F)cc-3oc3cc(N(CC(F)(F)F)CC(F)(F)F)ccc23)c(CCO)c1	O	507	CCN(CC)CCN(C)C(=O)c1ccccc1-c1c2ccc(=[N+](CC)CC)cc-2oc2cc(N(CC)CC)ccc12	CO	561	CC(C)[Si]1(C(C)C)C2=CC(=[N+](C)C)C=CC2=Cc2ccc(N(C)C)cc21	O	632
CC(=O)Nc1ccc2c(c1)Oc1c3c4c(cc1=C2c1ccccc	O	513	CCN(CC)CCN(C)C(=O)c1ccccc1-c1c2ccc(=[N+	CCO	564	CN(C)c1ccc2c(c1)[Si](c1ccccc1)(c1ccccc1)C1=CC(=[N	O	649

1CO)CCC[N+]=4CCC3			](CC)CC)cc-2oc2cc(N(CC)CC)ccc12			+](C)C)C=CC1=C2		
CC(=O)Nc1cc2c(cc1F)C(c1ccccc1CO)=c1cc3c4c(c1O2)CCC[N+]=4CCC3	O	519	CCN(CC)CCN(C)C(=O)c1ccccc1-c1c2ccc(=[N+](CC)CC)cc-2oc2cc(N(CC)CC)ccc12	CCCO	562	CC[Si]1(CCC(C)(C)C)C2=CC(=[N+](C)C)C=CC2=Cc2ccc(N(C)C)cc21	O	637
CC(=O)Nc1cc2c(cc1Cl)C(c1ccccc1CO)=c1cc3c4c(c1O2)CCC[N+]=4CCC3	O	517	CCN(CC)CCN(C)C(=O)c1ccccc1-c1c2ccc(=[N+](CC)CC)cc-2oc2cc(N(CC)CC)ccc12	CC(O)C	563	Cc1ccccc1C1=C2C=CC(=[N+](C)C)C=C2[Si](O)(O)c2cc(N(C)C)ccc21	O	663
CN(C)c1ccc2c(c1)[Si](C)(C)C1=CC(=[N+](C)C)C=CC1=C2c1ccc(C(=O)O)cc1CO	O	650	CCN(CC)CCN(C)C(=O)c1ccccc1-c1c2ccc(=[N+](CC)CC)cc-2oc2cc(N(CC)CC)ccc12	CC#N	560	CN(C)c1ccc2c(c1)[Ge](C)(C)C1=CC(=[N+](C)C)C=CC1=C2c1ccccc1C(=O)[O-]	O	634
O=C(O)c1ccccc1C1=c2cc3c(cc2Oc2cc4c(cc21)CCCN4)=NCCC3	O	538	CCN(CC)CCN(C)C(=O)c1ccccc1-c1c2ccc(=[N+](CC)CC)cc-2oc2cc(N(CC)CC)ccc12	CS(C)=O	569	c1cc2cc3ccc(N4CCC4)cc3nc2cc1N1CCC1	O	492
C[Si]1(C)c2cc3c(cc2C(c2ccccc2C(=O)O)=c2cc4c(cc21)=NCCC4)CCCN3	O	637	CCN(CC)CCN(C)C(=O)c1ccccc1-c1c2ccc(=[N+](CC)CC)cc-2oc2cc(N(CC)CC)ccc12	CN(C=O)C	566	Cc1ccccc1C1=C2C=CC(=[N+](C)C)C=C2P(=O)([O-])c2cc(N(C)C)ccc21	O	666
N=C1C=CC2=C(c3ccccc3C(=O)O)c3ccc(N)cc3P(=O)(O)C2=C1	CO	635	CCN(CC)CCN(C)C(=O)c1ccccc1-c1c2ccc(=[N+](CC)CC)cc-2oc2cc(N(CC)CC)ccc12	ClCCl	561	CN(C)c1ccc2c(-c3ccccc3)c3ccc(=[N+](C)C)cc-3sc2c1	CO	571
CCN(CC)c1ccc2c(c1)P(=O)([O-])C1=CC(=[N+](CC)CC)C=CC	CO	670	CCN(CC)CCN(C)C(=O)c1ccccc1-c1c2ccc(=[N+](CC)CC)cc-	ClC(Cl)Cl	562	c1ccc(C2=c3cc4c5c(c3Sc3c2cc2c6c3CCCN6CCC2)CCC[N+]=5CCC4)cc1	CO	594

1=C2c1ccccc1C(=O)O			2oc2cc(N(CC)CC)ccc12					
CCN1c2cc3c(cc2C(C)=CC1(C)C)C(c1ccccc1C(=O)O)=c1cc2c(cc1P3(=O)[O-])=[N+](CC)C(C)(C)C=C2C	CO	715	CCN(CC)CCN(C)C(=O)c1ccccc1-c1c2ccc(=[N+](CC)CC)cc-2oc2cc(N(CC)CC)ccc12	C1COCCO1	563	CN1CCC(C)(C)c2cc3c(cc21)Sc1cc2c(cc1=C3c1ccccc1)C(C)(C)CC[N+]=2C	CO	579
CN=C1C=CC2=C(c3ccccc3C(=O)O)c3ccc(NC)cc3P(=O)(O)C2=C1	CO	641	CCN(CC)c1ccc2c(-c3ccccc3C(=O)N3C[C@@H](C)O[C@@H](C)C3)c3ccc(=[N+](CC)CC)cc-3oc2c1	O	566	C[N+]1=c2cc3c(cc2C(C)(C)CC1)=C(c1ccccc1)c1cc2c4c(c1S3)CCCN4CCC2	CO	587
CN=C1C=CC2=C(c3ccccc3S(=O)(=O)O)c3ccc(NC)cc3P(=O)(O)C2=C1	CO	649	CCN(CC)c1ccc2c(-c3ccccc3C(=O)N3C[C@@H](C)O[C@@H](C)C3)c3ccc(=[N+](CC)CC)cc-3oc2c1	CO	560	CN(C)c1ccc2c(-c3ccccc3)c3ccc4cc(=[N+](C)C)ccc4c3sc2c1	CO	641
CN(C)c1ccc2c(c1)Oc1cc(N(C)C)ccc1C21c2ccccc2S(=O)(=O)N1CCCC(=O)NCCOCCOCCCCCCCl	O	561	CCN(CC)c1ccc2c(-c3ccccc3C(=O)N3C[C@@H](C)O[C@@H](C)C3)c3ccc(=[N+](CC)CC)cc-3oc2c1	CCO	563	CN(C)c1ccc2c(-c3ccccc3)c3ccc(=[N+](C)C)cc-3[se]c2c1	CO	581
CN(C)c1ccc2c(c1)Oc1cc(N(C)C)ccc1C21c2ccccc2S(=O)(=O)N1CCCC(=O)NCCOCCOCCCCCCCl	CCO	559	CCN(CC)c1ccc2c(-c3ccccc3C(=O)N3C[C@@H](C)O[C@@H](C)C3)c3ccc(=[N+](CC)CC)cc-3oc2c1	CCCO	562	c1ccc(C2=c3cc4c5c(c3[Se]c3c2cc2c6c3CCCN6CCC2)CCC[N+]=5CCC4)cc1	CO	604
CN(C)c1ccc2c(c1)Oc1cc(N(C)C)ccc1C21c2ccccc2S(=O)(=O)N1CCCC(=O)	OCC(O)CO	565	CCN(CC)c1ccc2c(-c3ccccc3C(=O)N3C[C@@H](C)O[C@@H](C)C3)c3c	CC(O)C	562	CN1CCC(C)(C)c2cc3c(cc21)[Se]c1cc2c(cc1=C3c1ccccc1)C(C)(C)CC[N+]=2C	CO	590

NCCOCCOCCCCCCCl			cc(=[N+](CC)CC)cc-3oc2c1					
CCN(CC)c1ccc2c(c1)Oc1cc(N(CC)CC)ccc1C21c2ccccc2S(=O)(=O)N1CCCC(=O)NCCOCCOCCCCCCCl	O	566	CCN(CC)c1ccc2c(-c3ccccc3C(=O)N3C[C@@H](C)O[C@@H](C)C3)c3ccc(=[N+](CC)CC)cc-3oc2c1	CC#N	560	C[N+]1=c2cc3c(cc2C(C)(C)CC1)=C(c1ccccc1)c1cc2c4c(c1[Se]3)CCCN4CCC2	CO	597
CCN(CC)c1ccc2c(c1)Oc1cc(N(CC)CC)ccc1C21c2ccccc2S(=O)(=O)N1CCCC(=O)NCCOCCOCCCCCCCl	CCO	560	CCN(CC)c1ccc2c(-c3ccccc3C(=O)N3C[C@@H](C)O[C@@H](C)C3)c3ccc(=[N+](CC)CC)cc-3oc2c1	CS(C)=O	568	CN(C)c1ccc2c(-c3ccccc3)c3ccc(=[N+](C)C)cc-3[te]c2c1	CO	597
CCN(CC)c1ccc2c(c1)Oc1cc(N(CC)CC)ccc1C21c2ccccc2S(=O)(=O)N1CCCC(=O)NCCOCCOCCCCCCCl	OCC(O)CO	572	CCN(CC)c1ccc2c(-c3ccccc3C(=O)N3C[C@@H](C)O[C@@H](C)C3)c3ccc(=[N+](CC)CC)cc-3oc2c1	CN(C=O)C	565	c1ccc(C2=c3cc4c5c(c3[Te]c3c2cc2c6c3CCCN6CCC2)CCC[N+]=5CCC4)cc1	CO	617
O=C(CCCN1C2(c3ccc(N4CCCC4)cc3Oc3cc(N4CCCC4)ccc32)c2ccccc2S1(=O)=O)NCCOCCOCCCCCCCl	O	567	CCN(CC)c1ccc2c(-c3ccccc3C(=O)N3C[C@@H](C)O[C@@H](C)C3)c3ccc(=[N+](CC)CC)cc-3oc2c1	ClCCl	562	CN1CCC(C)(C)c2cc3c(cc21)[Te]c1cc2c(cc1=C3c1ccccc1)C(C)(C)CC[N+]=2C	CO	607
O=C(CCCN1C2(c3ccc(N4CCCC4)cc3Oc3cc(N4CCCC4)ccc32)c2ccccc2S1(=O)=O)NCCOCCOCCCCCCCl	CCO	563	CCN(CC)c1ccc2c(-c3ccccc3C(=O)N3C[C@@H](C)O[C@@H](C)C3)c3ccc(=[N+](CC)CC)cc-3oc2c1	ClC(Cl)Cl	563	C[N+]1=c2cc3c(cc2C(C)(C)CC1)=C(c1ccccc1)c1cc2c4c(c1[Te]3)CCCN4CCC2	CO	610
O=C(CCCN1C2(c3ccc(N4CCCCC4)cc3Oc3c	O	573	CCN(CC)c1ccc2c(-c3ccccc3C(=	C1COCCO1	563	Cc1cc(C)c(C2=c3cc4c5c(c3[Te]c3c2cc2c6	CO	617

c(N4CCCCC4)ccc32)c2ccccc2S1(=O)=O)NCCOCCOCCCCCCCl			O)N3C[C@@H](C)O[C@@H](C)C3)c3ccc(=[N+](CC)CC)cc-3oc2c1			c3CCCN6CCC2)CCC[N+]=5CCC4)c(C)c1		
O=C(CCCN1C2(c3ccc(N4CCCCC4)cc3Oc3cc(N4CCCCC4)ccc32)c2ccccc2S1(=O)=O)NCCOCCOCCCCCCCl	CCO	569	CCN(CC)c1ccc2c(-c3ccccc3C(=O)N3CCN(CCO)CC3)c3ccc(=[N+](CC)CC)cc-3oc2c1	O	566	Cc1cc(C)c(C2=c3cc4c(cc3[Te]c3cc5c(cc32)C(C)(C)CCN5C)=[N+](C)CCC4(C)C)c(C)c1	CO	606
Cc1cccc2c1COC21c2ccc(N)cc2Oc2cc(N)ccc21	O	505	CCN(CC)c1ccc2c(-c3ccccc3C(=O)N3CCN(CCO)CC3)c3ccc(=[N+](CC)CC)cc-3oc2c1	CO	561	CCN=c1cc2oc3cc(NCC)c(C)cc3c(-c3ccccc3C(=O)O)c-2cc1C	O	521
Nc1ccc2c(c1)Oc1cc(N)ccc1C21OCc2c(F)cccc21	O	508	CCN(CC)c1ccc2c(-c3ccccc3C(=O)N3CCN(CCO)CC3)c3ccc(=[N+](CC)CC)cc-3oc2c1	CCO	563	CCN=c1ccc2c(-c3ccccc3C(=O)O)c3ccc(NCC)cc3oc-2c1	O	554
Nc1ccc2c(c1)Oc1cc(N)ccc1C21OCc2c(C(F)(F)F)cccc21	O	505	CCN(CC)c1ccc2c(-c3ccccc3C(=O)N3CCN(CCO)CC3)c3ccc(=[N+](CC)CC)cc-3oc2c1	CCCO	562	CCN=c1cc2oc3cc(NCC)ccc3c(-c3ccccc3C(=O)O)c-2cc1C	O	539
Cc1ccc2c(c1)C1(OC2)c2ccc(N)cc2Oc2cc(N)ccc21	O	505	CCN(CC)c1ccc2c(-c3ccccc3C(=O)N3CCN(CCO)CC3)c3ccc(=[N+](CC)CC)cc-3oc2c1	CC(O)C	561	O=C([O-])c1ccccc1C1=c2cc3c4c(c2Oc2c1cc1c5c2CCCN5CCC1)CCC[N+]=4CCC3	O	580
C[Si]1(C)c2cc(N)ccc2C2(OCc3sccc32)c2ccc(N)cc21	O	600	CCN(CC)c1ccc2c(-c3ccccc3C(=O)N3CCN(CCO)CC3)c3c	CC#N	560	CCN(CC)c1ccc2c(c1)Oc1c3c4c(cc1=C2c1ccccc1C(=O)[O-	O	566

			cc(=[N+](CC)CC)cc-3oc2c1			])CCC[N+]=4CCC3		
C[Si]1(C)c2cc(N)ccc2C2(OCc3sccc32)c2ccc(NC(=O)CCC(N)C(=O)O)cc21	O	500	CCN(CC)c1ccc2c(-c3ccccc3C(=O)N3CCN(CCO)CC3)c3ccc(=[N+](CC)CC)cc-3oc2c1	CS(C)=O	569	CCNc1cc2c(cc1C)C(c1ccccc1C(=O)[O-])=c1cc3c4c(c1O2)CCC[N+]=4CCC3	O	554
CC1OC2(c3ccc(N)cc3C(C)(C)c3cc(N)ccc32)c2ccsc21	O	560	CCN(CC)c1ccc2c(-c3ccccc3C(=O)N3CCN(CCO)CC3)c3ccc(=[N+](CC)CC)cc-3oc2c1	CN(C=O)C	566	CC/N=c1/cc2oc3cc4c(cc3c(-c3ccccc3C(=O)O)c-2cc1C)N(CC)C1CCCCC1N4CC	CCO	570
CC1OC2(c3ccc(N)cc3C(C)(C)c3cc(NC(=O)CCC(N)C(=O)O)ccc32)c2ccsc21	O	490	CCN(CC)c1ccc2c(-c3ccccc3C(=O)N3CCN(CCO)CC3)c3ccc(=[N+](CC)CC)cc-3oc2c1	ClCCl	562	N=c1ccc2c(-c3ccc(C(=O)O)cc3C(=O)O)c3ccc(N)c(S(=O)(=O)O)c3oc-2c1S(=O)(=O)O	O	495
C[Si]1(C)c2cc(N3CCC3)ccc2C2(OCc3cc(C(=O)O)ccc32)C2C=CC(N3CCC3)=CC21	N#CC	655	CCN(CC)c1ccc2c(-c3ccccc3C(=O)N3CCN(CCO)CC3)c3ccc(=[N+](CC)CC)cc-3oc2c1	ClC(Cl)Cl	563	CC1CC(C)(C)N=c2c1cc1c(c2S(=O)(=O)O)Oc2c(ccc(N)c2S(=O)(=O)O)C=1c1ccc(C(=O)O)cc1C(=O)O	O	518
C[Si]1(C)c2cc(N3CCC3)ccc2C2(OCc3cc(C(=O)O)ccc32)C2C=CC(N3CCC3)=CC21	ClC(Cl)Cl	567	CCN(CC)c1ccc2c(-c3ccccc3C(=O)N3CCN(CCO)CC3)c3ccc(=[N+](CC)CC)cc-3oc2c1	C1COCCO1	564	CC1N=c2c(cc3c(c2S(=O)(=O)O)Oc2c(cc4c(c2S(=O)(=O)O)NC(C)C4(C)C)C=3c2ccc(C(=O)O)cc2C(=O)O)C1(C)C	O	531
C[Si]1(C)c2cc(N3CCC3)ccc2C2(OCc3cc(C(=O)O)ccc32)C2C=CC(N3CCC3)=CC21	CS(C)=O	667	CCN(CC)c1ccc2c(-c3ccccc3C(=O)N3CCN(c4ccccc4O)CC3)c3ccc(=[N+	O	566	CC1(C)C=C(CS(=O)(=O)O)c2cc3c(c(S(=O)(=O)O)c2=N1)Oc1c(cc2c(c1S(=O)(=O)O)NC(C)(C)	O	578

			](CC)CC)cc-3oc2c1			C=C2CS(=O)(=O)O)C=3c1ccc(C(=O)O)cc1C(=O)O		
C[Si]1(C)c2cc(N3CCC3)ccc2C2(OCc3cc(C(=O)O)ccc32)C2C=CC(N3CCC3)=CC21	CC(OCC)=O	658	CCN(CC)c1ccc2c(-c3ccccc3C(=O)N3CCN(c4ccccc4O)CC3)c3ccc(=[N+](CC)CC)cc-3oc2c1	CO	561	CN1c2c(cc3c(c2S(=O)(=O)O)Oc2c(S(=O)(=O)[O-])c4c(cc2=C3c2ccc(C(=O)O)cc2C(=O)O)C(CS(=O)(=O)O)=CC(C)(C)[N+]=4C)C(CS(=O)(=O)O)=CC1(C)C	O	590
C[Si]1(C)c2cc(N3CCC3)ccc2C2(OCc3cc(C(=O)O)ccc32)C2C=CC(N3CCC3)=CC21	CCO	657	CCN(CC)c1ccc2c(-c3ccccc3C(=O)N3CCN(c4ccccc4O)CC3)c3ccc(=[N+](CC)CC)cc-3oc2c1	CCO	563	Nc1ccc2c(c1)Cc1c-2[o+]c2ccccc2c1-c1ccccc1C(=O)O	O	478
C[Si]1(C)c2cc(N3CCC3)ccc2C2(OCc3cc(C(=O)O)ccc32)C2C=CC(N3CCC3)=CC21	O	655	CCN(CC)c1ccc2c(-c3ccccc3C(=O)N3CCN(c4ccccc4O)CC3)c3ccc(=[N+](CC)CC)cc-3oc2c1	CCCO	562	COc1ccc2c(-c3ccccc3C(=O)O)c3c([o+]c2c1)-c1ccc(N)cc1C3	O	490
C[N+]1(C)CCN(C2=CC3Oc4cc(N5CC[N+](C)(C)CC5)ccc4C4(OCc5ccccc54)C3C=C2)CC1	N#CC	533	CCN(CC)c1ccc2c(-c3ccccc3C(=O)N3CCN(c4ccccc4O)CC3)c3ccc(=[N+](CC)CC)cc-3oc2c1	CC(O)C	562	Nc1ccc2c(c1)Cc1c-2[o+]c2cc(O)ccc2c1-c1ccccc1C(=O)O	O	522
C[N+]1(C)CCN(C2=CC3Oc4cc(N5CC[N+](C)(C)CC5)ccc4C4(OCc5ccccc54)C3C=C2)CC1	CC(OCC)=O	541	CCN(CC)c1ccc2c(-c3ccccc3C(=O)N3CCN(c4ccccc4O)CC3)c3ccc(=[N+](CC)CC)cc-3oc2c1	CC#N	560	CN(C)c1ccc2c(-c3ccccc3C(=O)O)c3c([o+]c2c1)-c1ccc(N)cc1C3	O	557
C[N+]1(C)CCN(C2=CC3Oc4cc(N5CC[N+](	CCO	537	CCN(CC)c1ccc2c(-c3ccccc3C(=	CS(C)=O	568	Nc1ccc2c(c1)Cc1c-2[o+]c2cc(Cl)	O	484

C)(C)CC5)ccc4C4(OCc5ccccc54)C3C=C2)CC1			O)N3CCN(c4ccccc4O)CC3)c3ccc(=[N+](CC)CC)cc-3oc2c1			ccc2c1-c1ccccc1C(=O)O		
C[N+]1(C)CCN(C2=CC3Oc4cc(N5CC[N+](C)(C)CC5)ccc4C4(OCc5ccccc54)C3C=C2)CC1	O	531	CCN(CC)c1ccc2c(-c3ccccc3C(=O)N3CCN(c4ccccc4O)CC3)c3ccc(=[N+](CC)CC)cc-3oc2c1	CN(C=O)C	566	Nc1ccc2c(c1)Cc1c-2[o+]c2cc3c4c(c2c1-c1ccccc1C(=O)O)CCCN4CCC3	O	582
CCN(CC)c1ccc2c(c1)[Si](C)(C)c1cc(N(CC)CC)ccc1C21OC(=O)c2ccccc21	O	650	CCN(CC)c1ccc2c(-c3ccccc3C(=O)N3CCN(c4ccccc4O)CC3)c3ccc(=[N+](CC)CC)cc-3oc2c1	ClCCl	563	CCN(CC)c1ccc2c(-c3ccccc3C(=O)O)c3c([o+]c2c1)-c1ccc(N)cc1C3	O	564
O=c1ccc2c(-c3ccccc3CO)c3ccc(NCC(F)(F)F)cc3oc-2c1	O	479	CCN(CC)c1ccc2c(-c3ccccc3C(=O)N3CCN(c4ccccc4O)CC3)c3ccc(=[N+](CC)CC)cc-3oc2c1	ClC(Cl)Cl	562	CCN(CC)c1ccc2c(-c3ccccc3C(=O)O)c3c([o+]c2c1)-c1ccc(Cl)cc1C3	CCO	540
Oc1ccc2c(c1)Oc1cc(NCC(F)(F)F)ccc1C21OCc2ccccc21	O	498	CCN(CC)c1ccc2c(-c3ccccc3C(=O)N3CCN(c4ccccc4O)CC3)c3ccc(=[N+](CC)CC)cc-3oc2c1	C1COCCO1	564	CCN(CC)c1ccc2c(-c3ccccc3C(=O)O)c3c([o+]c2c1)-c1ccccc1C3	CCO	524
CC(=O)N[C@H]1[C@@H](Oc2ccc3c(c2)Oc2cc(NCC(F)(F)F)ccc2C3c2ccccc2CO)O[C@H](CO)[C@@H](O)[C@@H]1O	O	482	CC1=CC(C)=[N+]2C1=C(c1ccccc1)c1c(C)cc(/C=C/c3ccc(N(C)C)cc3)n1[B-]2(F)F	CC#N	597	CCN(CC)c1ccc2c(-c3ccccc3C(=O)O)c3c([o+]c2c1)-c1ccc(OC)cc1C3	CCO	550
CCN(CC)c1ccc2c(c1)Oc1c(ccc(NC(=O)CCC(N)C(=O)O)c1CF)C21OCc2ccccc21	O	499	Cc1ccccc1C1=c2cc3c(cc2[Si](C)(C)c2cc4c(cc21)CCN4C)=[N+](C)CC3	O	691	CCN(CC)c1ccc2c(-c3ccccc3C(=O)O)c3c([o+]c2c1)-	CCO	567

						c1ccc(O)cc1C3		
CCN(CC)c1ccc2c(-c3ccccc3CO)c3ccc(=N)c(CO)c-3oc2c1	O	534	CC1=CC(C)(C)N(C)c2cc3c(cc21)C(c1ccccc1C)=c1cc2c(cc1[Si]3(C)C)=[N+](C)C(C)(C)C=C2C	O	712	CCN(CC)c1ccc2c(-c3ccccc3C(=O)O)c3c([o+]c2c1)-c1ccc(NC)cc1C3	CCO	573
CN(C)c1ccc2c(c1)[Si](C)(C)c1cc(O)ccc1C21OC(=O)c2ccccc21	O	609	Cc1ccccc1-c1c2ccc(=[N+](C)C)cc-2[te]c2cc(N(C)C)ccc12	O	600	CCN(CC)c1ccc2c(c1)Cc1c-2[o+]c2cc(N(CC)CC)ccc2c1-c1ccccc1C(=O)O	CCO	583
CN(C)c1ccc2c(c1)[Si](C)(C)c1c(ccc(O)c1CO)C21OC(=O)c2ccccc21	O	610	COc1ccc(N(C)c2ccc3c(c2)[Si](C)(C)C2=CC(=[N+](C)c4ccc(OC)cc4)C=CC2=C3c2ccccc2C)cc1	CCO	660	CN(C)c1ccc2c(c1)Cc1c-2[o+]c2cc3c4c(c2c1-c1ccccc1C(=O)O)CCCN4CCC3	O	600
CCN(CC)c1ccc2c(c1)Oc1c(ccc(O[C@@H]3O[C@H](CO)[C@H](O)[C@H](O)[C@H]3O)c1CF)C2c1ccccc1CO	O	493	COc1ccc(N(C)c2ccc3c(c2)[Si](C)(C)C2=CC(=[N+](C)c4ccc(OC)cc4)C=CC2=C3c2ccccc2C)cc1	CO	660	CCN(CC)c1ccc2c(c1)Cc1c-2[o+]c2cc3c4c(c2c1-c1ccccc1C(=O)O)CCCN4CCC3	O	604
CCN(CC)c1ccc2c(c1)Oc1c(ccc(O[C@@H]3O[C@H](CO)[C@H](O)[C@H](O)[C@H]3O)c1C(F)F)C2c1ccccc1CO	O	493	Cc1ccccc1C1=C2C=CC(=[N+]3CCc4ccccc43)C=C2[Si](C)(C)c2cc(N3CCc4ccccc43)ccc21	CCO	779	CCN(CC)c1ccc2c(-c3ccccc3C(=O)O)c3c([o+]c2c1)-c1cccc(O)c1C3	O	532
CCN(CC)c1ccc2c(c1)Oc1cc(O[C@@H]3O[C@H](CO)[C@H](O)[C@H](O)[C@H]3O)ccc1C2c1ccccc1CO	O	493	CCN(CC)c1ccc2c(c1)P(C)(=O)C1=CC(=[N+](CC)CC)C=CC1=C2c1c(C)cc(C)cc1C	ClCCl	687	CCN(CC)c1ccc2c(-c3ccccc3C(=O)O)c3c([o+]c2c1)-c1ccc(O)cc1C3	O	567
CC[N+](CC)=c1ccc2c(-c3ccccc3CO)c3ccc([O-	O	526	CCN(CC)c1ccc2c(c1)P(C)(=O)C1=CC(=[N+](CC)CC)	CC#N	684	CCN(CC)c1ccc2c(-c3ccccc3C(=O)O)c3c([o+]c2	O	545

])c(CO)c3oc-2c1			C=CC1=C2c1c(C)cc(C)cc1C			c1)-c1cc(O)ccc1C3		
CCN(CC)c1ccc2c(-c3ccccc3CO)c3ccc(=O)c(C=O)c-3oc2c1	O	524	CCN(CC)c1ccc2c(c1)P(C)(=O)C1=CC(=[N+](CC)CC)C=CC1=C2c1c(C)cc(C)cc1C	CCO	691	CCN(CC)c1ccc2c(-c3ccccc3C(=O)O)c3c([o+]c2c1)-c1c(O)cccc1C3	O	575
CCN(CC)c1ccc2c(-c3ccccc3CO)c3ccc(=O)cc-3oc2c1	O	525	CCN(CC)c1ccc2c(c1)P(C)(=O)C1=CC(=[N+](CC)CC)C=CC1=C2c1c(C)cc(C)cc1C	O	696	CCN(CC)c1ccc2c(-c3ccccc3C(=O)O)c3c([o+]c2c1)-c1ccc(N)cc1CC3	O	574
N=c1ccc2c(-c3ccccc3CO)c3ccc(N)cc3[se]c-2c1	O	534	CCN(CC)c1cc2c(c(-c3ccccc3C(=O)O)c1)C=c1cc3c(cc1O2)=[N+](CC)c1ccccc1S3	CCO	606	O=S(=O)([O-])c1ccccc1-[c+]1c2ccc(N3C4CCC3CC4)cc2oc2cc(N3[C@H]4CC[C@@H]3CC4)ccc21	CO	548
N=c1ccc2c(-c3ccccc3CO)c3ccc(NC(=O)CCC(N)C(=O)O)cc3[se]c-2c1	O	450	CCOP1(=O)C2=CC(=[N+](C)C)C=CC2=C(c2ccccc2C)c2ccc(N(C)C)cc21	O	698	CCN(CC)c1ccc2c(c1)Oc1cc3c(cc1=C2c1ccccc1C(=O)O)Sc1ccccc1[N+]=3CC	CCO	606
CC[N+](CC)=c1ccc2c(-c3ccccc3CO)c3ccc(NC(=O)CCC(N)C(=O)O)cc3[se]c-2c1	O	588	Cc1ccccc1C1=c2cc3c4c(c2P(=O)([O-])c2c1cc1c5c2CCCN5CCC1)CCC[N+]=4CCC3	O	700	CN(C)c1cc2oc3cc(=[N+](C)C)c(-c4ccccc4)cc-3c(-c3ccccc3C(=O)[O-])c2cc1-c1ccccc1	CO	548
CC[N+](CC)=c1ccc2c(-c3ccccc3CO)c3ccc(N)cc3[se]c-2c1	O	567	CCOP1(=O)c2c(cc3c4c2CCCN4CCC3)C(c2ccccc2C)=c2cc3c4c(c21)CCC[N+]=4CCC3	O	744	CN1c2cc3c(cc2-c2ccccc2C1(C)C)C(c1ccccc1C(=O)[O-])=c1cc2c(cc1O3)=[N+](C)C(C)(C)c1ccccc1-2	CO	582
CCN(CC)c1ccc2c(c1)[Si](C)(C)c1cc(N(CC)C	O	651	CCN(CC)c1ccc2c(c1)P(C)(=O)C1=CC(=	ClCCl	688	CN1c2cc3c(cc2-c2ccsc2C1(C)	CO	581

C)ccc1C21OB(O)c2ccccc21			[N+](CC)CC)C=CC1=C2c1ccccc1			C)C(c1ccccc1C(=O)[O-])=c1cc2c(cc1O3)=[N+](C)C(C)(C)c1sccc1-2		
CCN(CC)c1ccc2c(-c3ccccc3B(O)O)c3ccc(=[N+](CC)CC)cc-3oc2c1	O	555	CCN(CC)c1ccc2c(c1)P(C)(=O)C1=CC(=[N+](CC)CC)C=CC1=C2c1ccccc1	CC#N	682	CN1c2cc3c(cc2-c2sccc2C1(C)C)C(c1ccc(C(=O)O)cc1C(=O)[O-])=c1cc2c(cc1O3)=[N+](C)C(C)(C)c1ccsc1-2	CO	596
CN(C)c1ccc2c(c1)P(=O)(O)c1cc(N(C)C)ccc1C21OC(=O)c2ccccc21	O	667	CCN(CC)c1ccc2c(c1)P(C)(=O)C1=CC(=[N+](CC)CC)C=CC1=C2c1ccccc1	CCO	690	CN1c2cc3c(cc2-c2sc4ccccc4c2C1(C)C)C(c1ccc(C(=O)O)cc1C(=O)[O-])=c1cc2c(cc1O3)=[N+](C)C(C)(C)c1c-2sc2ccccc12	CO	600
CCN(CC)c1ccc2c(c1)P(=O)(O)c1cc(N(CC)CC)ccc1C21OC(=O)c2ccccc21	O	672	CCN(CC)c1ccc2c(c1)P(C)(=O)C1=CC(=[N+](CC)CC)C=CC1=C2c1ccccc1	O	694	CN1c2cc3c(cc2-c2sccc2C1(C)C)C(c1c(Cl)c(Cl)c(Cl)c(Cl)c1C(=O)[O-])=c1cc2c(cc1O3)=[N+](C)C(C)(C)c1ccsc1-2	CO	631
CCN1CCCc2cc3c(cc21)P(=O)(O)c1cc2c(cc1C31OC(=O)c3ccccc31)CCCN2CC	O	700	CCN(CC)c1ccc2c(c1)P(C)(=O)C1=CC(=[N+](CC)CC)C=CC1=C2c1ccccc1C	ClCCl	688	CN1c2cc3c(cc2-c2sccc2C1(C)C)C(c1c(F)c(F)c(F)c(F)c1C(=O)[O-])=c1cc2c(cc1O3)=[N+](C)C(C)(C)c1ccsc1-2	CO	628
CCN1CCc2cc3c(cc21)P(=O)(O)c1cc2c(cc1C31OC(=O)c3ccccc31)CCN2CC	O	725	CCN(CC)c1ccc2c(c1)P(C)(=O)C1=CC(=[N+](CC)CC)	CC#N	684	CN1c2cc3c(cc2-c2ccc4ccccc4c2C1(C)C)C(c1c(Cl)c(Cl)c(	CO	627

			C=CC1=C2c1ccccc1C			Cl)c(Cl)c1C(=O)[O-])=c1cc2c(cc1O3)=[N+](C)C(C)(C)c1c-2ccc2ccccc12		
Cc1cccc(-c2c3ccc(=N)cc-3oc3cc(N)ccc23)c1C(C)O	O	498	CCN(CC)c1ccc2c(c1)P(C)(=O)C1=CC(=[N+](CC)CC)C=CC1=C2c1ccccc1C	CCO	691	CN1c2cc3c(cc2-c2sccc2C1(C)C)C(c1ccc(S(=O)(=O)O)cc1S(=O)(=O)[O-])=c1cc2c(cc1O3)=[N+](C)C(C)(C)c1ccsc1-2	CO	610
CC1(C)C2=CC(=N)C=CC2=C(c2ccccc2CO)c2ccc(N)cc21	O	559	CCN(CC)c1ccc2c(c1)P(C)(=O)C1=CC(=[N+](CC)CC)C=CC1=C2c1ccccc1C	O	694	CN1c2cc3c(cc2-c2sc4ccccc4c2C1(C)C)C(c1ccc(S(=O)(=O)O)cc1S(=O)(=O)[O-])=c1cc2c(cc1O3)=[N+](C)C(C)(C)c1c-2sc2ccccc12	CO	616
C[Si]1(C)C2=CC(=N)C=CC2=C(c2cc(C(=O)O)ccc2CO)c2ccc(N)cc21	O	595	CN(C)c1ccc2c(c1)S(=O)(=O)C1=CC(=[N+](C)C)C=CC1=C2c1ccccc1	O	703	CN1c2cc3c(cc2-c2c(sc4ccccc24)C1(C)c1ccccc1)C(c1ccc(S(=O)(=O)O)cc1S(=O)(=O)[O-])=c1cc2c(cc1O3)=[N+](C)C(C)(c1ccccc1)c1sc3ccccc3c1-2	CO	634
C[Si]1(C)C2=CC(=N)C=CC2=C(c2ccccc2CO)c2ccc(N)cc21	O	595	Cc1ccccc1C1=C2C=CC(=[N+](C)C)C=C2S(=O)(=O)c2cc(N(C)C)ccc21	O	703	CCN1CCCc2cc3c(cc21)C(C)(C)c1cc2c(cc1=C3c1ccccc1C(=O)O)CCC[N+]=2CC	CCO	632
C[N+](C)=C1C=CC2=C(c3ccccc3CO)c3ccc(N)	O	623	COc1ccccc1C1=C2C=CC(=[N+](C)C)C=C2S(=O)(=O)	O	707	CCN1CCCc2cc3c(cc21)C(C)(C)c1cc2c(cc1=C3c1ccccc	CCO	653

cc3[Si](C)(C)C2=C1			c2cc(N(C)C)ccc21			1C(=O)O)C(C)=CC(C)(C)[N+]=2CC		
C[Si]1(C)c2cc(N)ccc2C(c2ccccc2CO)=c2cc3c4c(c21)CCC[N+]=4CCC3	O	637	Cc1cccc(C)c1C1=C2C=CC(=[N+](C)C)C=C2S(=O)(=O)c2cc(N(C)C)ccc21	O	704	CCN1c2cc3c(cc2C(C)=CC1(C)C)C(c1ccccc1C(=O)O)=c1cc2c(cc1C3(C)C)=[N+](CC)C(C)(C)C=C2C	CCO	670
C[Si]1(C)c2cc(NC(=O)CCC(N)C(=O)O)ccc2C(c2ccccc2CO)=c2cc3c4c(c21)CCC[N+]=4CCC3	O	500	COc1cccc(OC)c1C1=C2C=CC(=[N+](C)C)C=C2S(=O)(=O)c2cc(N(C)C)ccc21	O	710	CCN1CCCc2cc3c(cc21)C(C)(C)c1cc2c(cc1=C3c1c(Cl)c(Cl)c(Cl)c(Cl)c1C(=O)O)CCC[N+]=2CC	CCO	662
CC(=O)N=c1ccc2c(-c3ccccc3CO)c3ccc(N)cc3oc-2c1	O	493	CCN(CC)c1ccc2c(C=CC=C3N(C)c4ccccc4C3(C)C)c3ccc(N(CC)CC)cc3[o+]c2c1	CC#N	651	CCN1CCCc2cc3c(cc21)C(C)(C)c1cc2c(cc1=C3c1c(Cl)c(Cl)c(Cl)c(Cl)c1C(=O)O)C(C)=CC(C)(C)[N+]=2CC	CCO	683
CC(=O)N=c1ccc2c(-c3ccccc3CN)c3ccc(N)cc3oc-2c1	O	500	CCN(CC)c1ccc2c(C=CC=C3Sc4ccccc4N3C)c3ccc(N(CC)CC)cc3[o+]c2c1	CC#N	663	CCN1c2cc3c(cc2C(C)=CC1(C)C)C(c1c(Cl)c(Cl)c(Cl)c(Cl)c1C(=O)O)=c1cc2c(cc1C3(C)C)=[N+](CC)C(C)(C)C=C2C	CCO	700
CN(C)c1ccc2c(c1)[Si](C)(C)C1=CC(=[N+](C)C)C=CC1=C2c1ccc(C(=O)O)cc1CS	O	653	CCN(CC)c1ccc2c(C=CC=C3C=Cc4ccccc4N3C)c3ccc(N(CC)CC)cc3[o+]c2c1	CC#N	684	CN(C)c1ccc2c(c1)C(C)(C)C1=CC(=[N+](C)C)C=CC1=C2c1c(Cl)c(Cl)c(Cl)c(Cl)c1C(=O)O	CCO	642
CN(C)c1ccc2c(c1)[Si](C)(C)C1=CC(=[N+](C)C)C=CC1=C2c1ccc(C(=O)O)cc1CN	O	656	CN(C)c1ccc2c(c1)P(=O)(c1ccccc1)C1=CC(=[N+](C)C)C=CC1=C2c1ccccc1	O	698	Cc1ccccc1C1=C2C=CC(=N)C=C2[Si](C)(C)c2cc(N)ccc21	O	593
CCN=c1cc2oc3cc(NCC)c(C)cc3c(-	O	525	COc1ccccc1C1=C2C=CC(=[N+](C)C)C=	O	701	C[Si]1(C)c2cc3c(cc2C(c2ccccc2C(=O)O)=	O	637

c3ccccc3CO)c-2cc1C			C2P(=O)(c2ccccc2)c2cc(N(C)C)ccc21			c2cc4c(cc21)=NCC=C4)CCCN3		
OCc1ccccc1C1=c2cc3c4c(c2Oc2c1cc1c5c2CCCN5CCC1)CCC[N+]=4CCC3	O	579	COc1cccc(OC)c1C1=C2C=CC(=[N+](C)C)C=C2P(=O)(c2ccccc2)c2cc(N(C)C)ccc21	O	703	CCN(CC)c1ccc2c(c1)[Si](C)(C)C1=CC(=[N+](CC)CC)C=CC1=C2c1ccccc1C(=O)O	O	650
CN(C)c1ccc2c(-c3ccccc3CS)c3ccc(=[N+](C)C)cc-3oc2c1	O	554	CCc1ccccc1C1=C2C=CC(=[N+](C)C)C=C2P(=O)(c2ccccc2)c2cc(N(C)C)ccc21	O	699	CN(C)c1ccc2cc3ccc(=[N+](C)C)cc-3oc2c1	O	547
CN(C)c1ccc2c(-c3ccccc3CN)c3ccc(=[N+](C)C)cc-3oc2c1	O	557	Cc1cccc(C)c1C1=C2C=CC(=[N+](C)C)C=C2P(=O)(c2ccccc2)c2cc(N(C)C)ccc21	O	730	CN(C)c1ccc2c(c1)C(C)(C)C1=CC(=[N+](C)C)C=CC1=C2CCCCCCCNC(=O)OC(C)(C)C	O	459
O=C(O)c1ccccc1-c1c2ccc(N3CCC3)cc2nc2cc(N3CCC3)ccc12	O	502	Cc1cc(C)c(C2=C3C=CC(=[N+](C)C)C=C3P(=O)(c3ccccc3)c3cc(N(C)C)ccc32)c(C)c1	O	700	CN(C)c1ccc2c(c1)[Si](C)(C)C1=CC(=[N+](C)C)C=CC1=C2CCCCCCCNC(=O)OC(C)(C)C	O	458
O=C([O-])c1ccccc1-c1c2ccc(=[N+]3CCC3)cc-2oc2cc(N3CCC3)ccc12	O	549	Cc1ccccc1C1=C2C=CC(=[N+](C)C)C=C2P(=O)(C(C)(C)C)c2cc(N(C)C)ccc21	O	693	CN(C)c1ccc2c(c1)S(=O)(=O)C1=CC(=[N+](C)C)C=CC1=C2CCCCCCCNC(=O)OC(C)(C)C	O	509
O=C([O-])c1ccccc1-c1c2ccc(=[N+]3CCC3)cc-2sc2cc(N3CCC3)ccc12	O	570	COc1cccc(OC)c1C1=C2C=CC(=[N+](C)C)C=C2P(=O)(C(C)(C)C)c2cc(N(C)C)ccc21	O	697	CN(C)c1ccc2c(c1)C(C)(C)C1=CC(=[N+](C)C)C=CC1=C2	CCO	606
O=C([O-])c1ccccc1C1=C2C=CC(=[N+]3CCC3)C=C2Cc2cc(N3CCC3)ccc21	O	608	Cc1ccccc1C1=C2C=CC(=[N+](C)C)C=C2P(=O)(c2ccccc2)c2cc(N(C)C)ccc21	O	699	CCN(CC)c1ccc2c(c1)C(C)(C)C1=CC(=[N+](C)C)C=CC1=C2	CO	608

C[Si]1(C)C2=CC(=[N+]3CCC3)C=CC2=C(c2ccccc2C(=O)[O-])c2ccc(N3CCC3)cc21	O	646	Cc1ccccc1C1=C2C=CC(=[N+]3CCC3)C=C2P(=O)(c2ccccc2)c2cc(N3CCC3)ccc21	O	699	CCN1CCc2cc3c(cc21)C(C)(C)c1cc2c(cc1=C3)CC[N+]=2CC	CCO	633
O=C([O-])c1ccccc1C1=C2C=CC(=[N+]3CCC3)C=C2P(=O)(O)c2cc(N3CCC3)ccc21	O	668	Cc1ccccc1C1=C2C=CC(=[N+]3CCCC3)C=C2P(=O)(c2ccccc2)c2cc(N3CCCC3)ccc21	O	708	CCN1CCCc2cc3c(cc21)C(C)(C)c1cc2c(cc1=C3)CCC[N+]=2CC	CCO	626
O=C([O-])c1ccccc1-c1c2ccc(=[N+]3CC(F)(F)C3)cc-2oc2cc(N3CC(F)(F)C3)ccc12	O	525	CN(C)c1ccc2c(c1)P(=O)(c1ccccc1)C1=CC(=[N+](C)C)C=CC1=C2c1ccccc1C(F)(F)F	O	706	CCN1c2cc3c(cc2C(C)=CC1(C)C)C=c1cc2c(cc1C3(C)C)=[N+](CC)C(C)(C)C=C2C	CCO	664
O=C([O-])c1ccccc1C1=C2C=CC(=[N+]3CC(F)(F)C3)C=C2Cc2cc(N3CC(F)(F)C3)ccc21	O	585	O=P1(c2ccccc2)C2=CC(=[N+]3CCC3)C=CC2=C(c2ccccc2C(F)(F)F)c2ccc(N3CCC3)cc21	O	710	CCN1CCc2cc3c(cc21)C(C)(C)c1cc2c(cc1=C3)CCC[N+]=2CC	CCO	629
O=C(O)c1ccccc1-c1c2ccc(N3CC(F)(F)C3)cc2nc2cc(N3CC(F)(F)C3)ccc12	O	579	O=P1(c2ccccc2)C2=CC(=[N+]3CCCC3)C=CC2=C(c2ccccc2C(F)(F)F)c2ccc(N3CCCC3)cc21	O	718	CCN1CCc2cc3c(cc21)C(C)(C)c1cc2c(cc1=C3)C(C)=CC(C)(C)[N+]=2CC	CCO	647
O=C([O-])c1ccccc1-c1c2cc(F)c(=[N+]3CCC3)cc-2oc2cc(N3CCC3)c(F)cc12	O	552	Cc1ccccc1C1=C2C=CC(=[N+]3CCCC3)C=C2P(=O)(c2ccccc2)c2cc(N(C)C)ccc21	O	703	CCN1CCCc2cc3c(cc21)C(C)(C)c1cc2c(cc1=C3)C(C)=CC(C)(C)[N+]=2CC	CCO	647
O=C([O-])c1ccccc1-c1c2cc(F)c(=[N+]3CC(F)(F)C3)cc-2oc2cc(N3CC(F)(F)C3)c(F)cc12	O	526	CCc1ccccc1C1=C2C=CC(=[N+]3CCCC3)C=C2P(=O)(c2ccccc2)c2cc(N(C)C)ccc21	O	703	CCN1CCc2cc3c(cc21)C(C)(C)C1=CC(=[N+](C)C)C=CC1=C3	CCO	617
C[Si]1(C)C2=CC(=[N+]3CCC3	O	669	CN(C)c1ccc2c(c1)P(=O)(c1	O	713	CCN1CCCc2cc3c(cc21)C(	CCO	616

)C=CC2=C(c2c(F)c(F)c(F)c(F)c2C(=O)[O-])c2ccc(N3CCC3)cc21			ccccc1)C1=CC(=[N+]3CCCC3)C=CC1=C2c1ccccc1C(F)(F)F			C)(C)C1=CC(=[N+](C)C)C=CC1=C3		
O=C([O-])c1c(F)c(F)c(F)c(F)c1C1=C2C=CC(=[N+]3CCC3)C=C2P(=O)(O)c2cc(N3CCC3)ccc21	O	690	COc1ccccc1C1=C2C=CC(=[N+]3CCCC3)C=C2P(=O)(c2ccccc2)c2cc(N(C)C)ccc21	O	706	CCN1c2cc3c(cc2C(C)=CC1(C)C)C=C1C=CC(=[N+](C)C)C=C1C3(C)C	CCO	634
O=C([O-])c1c(F)c(F)c(F)c(F)c1C1=C2C=CC(=[N+]3CCC3)C=C2P(=O)(c2ccccc2)c2cc(N3CCC3)ccc21	O	722	COc1cccc(OC)c1C1=C2C=CC(=[N+]3CCCC3)C=C2P(=O)(c2ccccc2)c2cc(N(C)C)ccc21	O	706	CCN(CC)c1ccc2c(SCC(NC(=O)CCC(N)C(=O)O)C(=O)NCC(=O)O)c3ccc(=[N+](CC)CC)cc-3oc2c1	O	593
O=C([O-])c1c(F)c(F)c(F)c(F)c1C1=C2C=CC(=[N+]3CCC3)C=C2S(=O)(=O)c2cc(N3CCC3)ccc21	O	724	O=S(=O)([O-])c1ccccc1-[c+]1c2ccc(N3C4CCC3CC4)cc2oc2cc(N3C4CCC3CC4)ccc21	CO	548	N#CC1=c2cc3c4c(c2Oc2c1cc1c5c2CCCN5CCC1)CCC[N+]=4CCC3	O	690
O=C([O-])c1c(F)c(F)c(F)c(F)c1-c1c2ccc(=[N+]3CCC3)cc-2oc2cc(N3CCC3)ccc12	O	571	CN(C)c1ccc2c(c1)oc1cc(N(C)C)ccc1[c+]2-c1ccccc1S(=O)(=O)[O-]	CO	550	C=c1ccc2c(c1)Oc1cc(C)ccc1N=2	CO	583
O=C([O-])c1c(F)c(F)c(F)c(F)c1-c1c2ccc(=[N+]3CCC3)cc-2sc2cc(N3CCC3)ccc12	O	593	O=C(O)c1ccccc1-c1c2cc3c4c(c2[o+]c2c5c6c(cc12)CCCN6CCC5)CCCN4CCC3	CO	568	CC=Cc1ccc2c(c1)Oc1cc(CCC)ccc1N2	CO	589
O=C([O-])c1c(F)c(F)c(F)c(F)c1-c1c2ccc(=[N+]3CC(F)C3)cc-2oc2cc(N3CC(F)C3)ccc12	O	559	O=C(O)c1ccccc1-c1c2cc3c4c(c2[o+]c2c5c6c(cc12)CCCN6CCC5)CCCN4CCC3	CS(C)=O	567	CC=C(CC)c1ccc2c(c1)Oc1cc(C(CC)CC)ccc1N2	CO	643
O=C([O-])c1c(F)c(F)c(F)c(F)c1C1=C2C	O	711	CN1c2cc3c(cc2-c2sc4ccccc4c	CO	616	CC[N+]1=c2cc3c(cc2CC1)=	CCO	612

=CC(=[N+]3CC(F)C3)C=C2P(=O)(c2ccccc2)c2cc(N3CC(F)C3)ccc21			2C1(C)C)C(c1ccc(S(=O)(=O)[O-])cc1S(=O)(=O)O)=c1cc2c(cc1O3)=[N+](C)C(C)(C)c1c-2sc2ccccc12			Nc1ccc(C)cc1O3		
N=c1ccc2c(-c3cccc(C(=O)O)c3C(=O)O)c3ccc(N)cc3oc-2c1	O	500	CN1c2cc3c(cc2-c2sc4ccccc4c2C1(C)c1ccccc1)C(c1ccc(S(=O)(=O)O)cc1S(=O)(=O)[O-])=c1cc2c(cc1O3)=[N+](C)C(C)(c1ccccc1)c1c-2sc2ccccc12	CO	613	CC[N+]1=c2cc3c(cc2CCC1)=Nc1cc2c(cc1O3)N(CCCC(=O)O)CCC2	O	662
CN=c1ccc2c(-c3cccc(C(=O)O)c3C(=O)O)c3ccc(NC)cc3oc-2c1	O	522	CN1c2cc3c(cc2-c2sc4ccccc4c2C1(C)c1ccccc1)C(c1ccc(S(=O)(=O)O)cc1S(=O)(=O)[O-])=c1cc2c(cc1O3)=[N+](C)C(C)(c1ccccc1)c1c-2sc2ccccc12	CS(C)=O	617	CC[N+]1=c2cc3c(cc2C(C)CC1(C)C)=Nc1cc2c(cc1O3)N(CCCC(=O)O)CCC2	O	664
C[N+](C)=c1ccc2c(-c3cccc(C(=O)O)c3C(=O)[O-])c3ccc(O)cc3oc-2c1	O	521	CN1c2cc3c(cc2-c2c(n(C)c4ccccc24)C1(C)c1ccccc1)C(c1ccc(S(=O)(=O)O)cc1S(=O)(=O)[O-])=c1cc2c(cc1O3)=[N+](C)C(C)(c1ccccc1)c1c-2c2ccccc2n1C	CO	634	CN1CCOc2cc3c(cc21)Oc1cc2c(cc1=N3)OCC[N+]=2C	CO	644
C=CCN(CC=C)c1ccc2c(-c3cccc(C(=O)O	O	548	CN(C)c1ccc2c(c1)[Si](C)(C)C1=CC(=[	O	634	CN1CCSc2cc3c(cc21)Oc1c	CO	659

)c3C(=O)[O-])c3ccc(=[N+](CC=C)CC=C)cc-3oc2c1			N+](C)C)C=CC1=C2			c2c(cc1=N3)OCC[N+]=2C		
C=CCN(C)c1ccc2c(-c3cccc(C(=O)O)c3C(=O)[O-])c3cc/c(=[N+](\C)CC=C)cc-3oc2c1	O	549	Cc1ccccc1C1=C2C=CC(=[N+](C)C)C=C2[Si](C)(C)c2cc(N(C)C)ccc21	O	646	CN1CCCc2cc3c(cc21)Oc1cc2c(cc1=N3)OCC[N+]=2C	CO	649
CC1(C)C2=CC(=O)C=CC2=C(c2cccc(C(=O)O)c2C(=O)O)c2ccc(O)cc21	O	549	Cc1ccccc1C1=C2C=CC(=[N+](C)C)C=C2[Ge](C)(C)c2cc(N(C)C)ccc21	O	635	N=c1cc2oc3cc(N)ccc3nc-2c2ccccc12	CCO	600
CN(C)c1ccc2c(-c3cccc(C(=O)O)c3C(=O)[O-])c3ccc(=[N+](C)C)cc-3oc2c1	O	551	Cc1ccccc1C1=c2cc3c(cc2[Si](C)(C)c2cc4c(cc21)CCCN4C)=[N+](C)CCC3	O	674	CCN(CC)c1ccc2nc3c4ccccc4c(=N)cc-3oc2c1	O	635
O=C(O)c1cccc(C2=c3cc4c5c(c3Oc3c2cc2c6c3CCCN6CCC2)CCC[N+]=5CCC4)c1C(=O)[O-]	O	579	CCN=c1cc2oc3cc(NCC)c(C)cc3c(-c3ccccc3C(=O)OC)c-2cc1C	CCO	530	N=c1cc2oc3cc(N4CCCC4)ccc3nc-2c2ccccc12	O	643
CN=C1C=CC2=C(c3cccc(C(=O)O)c3C(=O)O)c3ccc(NC)cc3CC2=C1	O	585	CCN(CC)c1ccc2c(-c3ccccc3C(=O)[O-])c3ccc(=[N+](CC)CC)cc-3oc2c1	CCO	553	N=c1cc2oc3cc(N(CCCS(=O)(=O)O)CCCS(=O)(=O)O)ccc3nc-2c2ccccc12	O	633
CN(C)c1ccc2c(c1)C(C)(C)C1=CC(=[N+](C)C)C=CC1=C2c1cccc(C(=O)O)c1C(=O)[O-]	O	611	CCN(CC)c1ccc2c(-c3ccccc3C(=O)[O-])c3ccc(=[N+](CC)CC)cc-3oc2c1	O	554	CCN(CC)c1ccc2c(c1)OC1=C(/C=C/C3=[N+](C)c4ccccc4C3(C)C)CCCC1=C2	CO	700
C=CCN(C)c1ccc2c(c1)C(C)(C)C1=C/C(=[N+](/C)CC=C)C=CC1=C2c1cccc(C(=O)O)c1C(=O)[O-]	O	611	O=C([O-])c1ccccc1C1=c2cc3c4c(c2Oc2c1cc1c5c2CCCN5CCC1)CCC[N+]=4CCC3	CCO	574	CCNc1cc2c(cc1C)C(c1ccccc1C(=O)O)=C1CCCC(/C=C/C3=[N+](C)c4ccccc4C3(C)C)=C1O2	CCO	688

CN(C)c1ccc2c(c1)C(C)(C)c1cc3c(cc1=C2c1cccc(C(=O)O)c1C(=O)[O-])CC[N+]=3C	O	621	CC[N+](CC)=c1ccc2c(-c3ccccc3C(=O)O)c3cc4c(cc3oc-2c1)N1CCCC1CN4C	O	575	CCNc1cc2c(cc1C)C(c1ccccc1C(=O)O)=C1CCCC(/C=C/C3=[N+](C)c4ccc5ccccc5c4C3(C)C)=C1O2	CCO	708
CN(C)c1ccc2c(c1)C(C)(C)c1c3c4c(cc1=C2c1cccc(C(=O)O)c1C(=O)[O-])CCC[N+]=4CCC3	O	624	CN1CC2CCCN2c2cc3c(cc21)C(c1ccccc1C(=O)O)=c1cc2c4c(c1O3)CCC[N+]=4CCC2	O	586	CCN(CC)c1cc2c(cc1C)C(c1ccccc1C(=O)O)=C1CCCC(/C=C/C3=[N+](C)c4ccccc4C3(C)C)=C1O2	CCO	700
CN1CCc2cc3c(cc21)C(C)(C)c1cc2c(cc1=C3c1ccccc(C(=O)O)c1C(=O)[O-])CC[N+]=2C	O	636	N=c1ccc2c(-c3ccccc3C(=O)O)c3ccc(N)cc3oc-2c1	O	479	CCN(CC)c1cc2c(cc1C)C(c1ccccc1C(=O)O)=C1CCCC(/C=C/C3=[N+](C)c4ccc5ccccc5c4C3(C)C)=C1O2	CCO	720
CN(C)c1ccc2c(c1)C(C)(C)c1cc3c(cc1=C2c1cccc(C(=O)O)c1C(=O)[O-])C=CC(C)(C)[N+]=3C	O	641	CN=c1ccc2c(-c3ccccc3C(=O)O)c3ccc(NC)cc3oc-2c1	O	530	C[N+]1=C(/C=C/C2=C3Oc4c(cc5c6c4CCCN6CCC5)C(c4ccccc4C(=O)O)=C3CCC2)C(C)(C)c2ccccc21	CCO	710
CC1(C)c2c(cc3c4c2CCCN4CCC3)C(c2cccc(C(=O)O)c2C(=O)[O-])=c2cc3c4c(c21)CCC[N+]=4CCC3	O	642	CN(C)c1ccc2c(-c3ccccc3C(=O)[O-])c3ccc(=[N+](C)C)cc-3oc2c1	O	548	C[N+]1=C(/C=C/C2=C3Oc4c(cc5c6c4CCCN6CCC5)C(c4ccccc4C(=O)O)=C3CCC2)C(C)(C)c2c1ccc1ccccc21	CCO	728
CN(C)c1ccc2c(c1)[Si](C)(C)C1=CC(=[N+](C)C)C=CC1=C2c1cccc(C(=O)O)c1C(=O)[O-]	O	649	CCNc1cc2c(cc1C)C(c1ccccc1C(=O)O)c1cc(C)c(NCC)cc1O2	O	521	CCN(CC)c1ccc2c(c1)OC1=C(/C=C/C3=[N+](CC)c4ccccc4C3(C)C)CCCC1=C2/C=C1/N(CC)c2ccccc2C1(C)C	ClCCl	720
CN(C)c1ccc2c(c1)[Si](C)(C)c1	O	663	CCNc1cc2oc3cc(=[N+](C	O	539	CCNc1cc2c(cc1C)C(/C=C1	ClCCl	710

c3c4c(cc1=C2c1cccc(C(=O)O)c1C(=O)[O-])CCC[N+]=4CCC3			C)CC)ccc-3c(-c3ccccc3C(=O)[O-])c2cc1C			/N(CC)c3ccccc3C1(C)C)=C1CCCC(/C=C/C3=[N+](CC)c4ccccc4C3(C)C)=C1O2		
CN(C)c1ccc2c(c1)[Si](C)(C)c1cc3c(cc1=C2c1cccc(C(=O)O)c1C(=O)[O-])CC[N+]=3C	O	668	O=C([O-])c1ccccc1-c1c2ccc(=[N+]3CCCC3)cc-2oc2cc(N3CCCC3)ccc12	O	553	CCNc1ccc2c(c1)OC1=C(/C=C/C3=[N+](CC)c4ccccc4C3(C)C)CCCC1=C2/C=C1/N(CC)c2ccccc2C1(C)C	ClCCl	712
CN1CCc2cc3c(cc21)[Si](C)(C)c1c2c4c(cc1=C3c1cccc(C(=O)O)c1C(=O)[O-])CCC[N+]=4CCC2	O	687	O=C([O-])c1ccccc1-c1c2ccc(=[N+]3CCCCC3)cc-2oc2cc(N3CCCCC3)ccc12	O	560	CCN1/C(=C/C2=C3CCCC(/C=C/C4=[N+](CC)c5ccccc5C4(C)C)=C3Oc3c2cc2c4c3CCCN4CCC2)C(C)(C)c2ccccc21	ClCCl	724
CC1=CC(C)(C)[N+](C)=c2cc3c(cc21)=C(c1cccc(C(=O)O)c1C(=O)[O-])c1ccc(N(C)C)cc1[Si]3(C)C	O	687	O=C([O-])c1ccccc1-c1c2ccc(=[N+]3CCCCCC3)cc-2oc2cc(N3CCCCCC3)ccc12	O	560	CCN(CC)c1ccc2c(-c3ccccc3C(=O)O)c3c([o+]c2c1)C(=Cc1ccc(O)cc1)CCC3	CO	570
CN1CCc2cc3c(cc21)[Si](C)(C)c1cc2c(cc1=C3c1cccc(C(=O)O)c1C(=O)[O-])CC[N+]=2C	O	694	O=C(O)c1ccccc1C1c2cc3c(cc2Oc2cc4c(cc21)CCCN4)NCCC3	O	538	CCN(CC)c1ccc2c(-c3ccccc3C(=O)O)c3c([o+]c2c1)C(=Cc1ccc(O)c(Cl)c1)CCC3	CO	577
CC1=CC(C)(C)N(C)c2cc3c(cc21)C(c1cccc(C(=O)O)c1C(=O)[O-])=c1cc2c(cc1[Si]3(C)C)=[N+](C)C(C)(C)C=C2C	O	721	O=C(O)c1ccccc1C1c2cc3c4c(c2Oc2c1cc1c5c2CCCN5CCC1)CCCN4CCC3	O	580	CCN(CC)c1cc(/C=C2\CCCc3c2[o+]c2cc(N(CC)CC)ccc2c3-c2ccccc2C(=O)O)cc1	CO	663
CC[N+]1=c2cc3c(cc2Sc2ccccc21)=C(c1c(F)c(F)c(F)c(F)c1C(=	CC(C)=O	633	CCN(CC)c1ccc2c(c1)Oc1c(cc3c4c1CCCN4CCC3)C2	O	566	CCN(CC)c1cc(/C=C2\CCCc3c2[o+]c2cc(N(CC)CC)c	CO	699

O)[O-])c1cc2c4c(c1O3)C(C)(C)CCN4CCC2(C)C			c1ccccc1C(=O)O			cc2c3-c2ccccc2C(=O)O)c(O)c1		
CC[N+]1=c2cc3c(cc2Sc2ccccc21)=C(c1c(F)c(F)c(F)c(F)c1C(=O)[O-])c1cc2c4c(c1O3)C(C)(C)CCN4CCC2(C)C	C1=CC=CC=C1	638	CCN1c2cc3oc4cc(=N)ccc-4c(-c4ccccc4C(=O)O)c3cc2N(CC)C2CCCCC21	CCO	565	CCN(CC)c1ccc2c(c1)OC(=C1C=CC(=[N+](CC)CC)C=C1)C=C2c1ccccc1C(=O)O	O	598
CC[N+]1=c2cc3c(cc2Sc2ccccc21)=C(c1c(F)c(F)c(F)c(F)c1C(=O)[O-])c1cc2c4c(c1O3)C(C)(C)CCN4CCC2(C)C	ClC(Cl)Cl	627	CCN=c1cc2oc3cc4c(cc3c(-c3ccccc3C(=O)O)c-2cc1C)N(CC)C1CCCCC1N4CC	CCO	570	CC[N+](CC)=C1C=CC(=C2C=C(c3ccccc3C(=O)O)c3cc4c5c(c3O2)CCCN5CCC4)C=C1	O	612
CC[N+]1=c2cc3c(cc2Sc2ccccc21)=C(c1c(F)c(F)c(F)c(F)c1C(=O)[O-])c1cc2c4c(c1O3)C(C)(C)CCN4CCC2(C)C	ClCCl	621	CCN1c2cc3oc4cc(=[N+](CC)CC)ccc-4c(-c4ccccc4C(=O)[O-])c3cc2N(CC)C2CCCCC21	CCO	584	CCN(CC)c1ccc2c(c1)O/C(=C/C=C1C=CC(=[N+](CC)CC)C=C1)C=C2c1ccccc1C(=O)O	O	676
CC[N+]1=c2cc3c(cc2Sc2ccccc21)=C(c1c(F)c(F)c(F)c(F)c1C(=O)[O-])c1cc2c4c(c1O3)C(C)(C)CCN4CCC2(C)C	CN(C=O)C	626	CCN1c2cc3c(cc2N(CC)C2CCCCC21)C(c1ccccc1C(=O)[O-])=c1cc2c4c(c1O3)CCC[N+]=4CCC2	CCO	593	CC[N+](CC)=C1C=CC(=C/C=C2\C=C(c3ccccc3C(=O)O)c3cc4c5c(c3O2)CCCN5CCC4)C=C1	O	678
CC[N+]1=c2cc3c(cc2Sc2ccccc21)=C(c1c(F)c(F)c(F)c(F)c1C(=O)[O-])c1cc2c4c(c1O3)C(C)(C)CCN4CCC2(C)C	CS(C)=O	634	CN(C)c1cc2oc3cc(=[N+](C)C)ccc-3c(-c3ccccc3C(=O)[O-])c2cc1-c1ccccc1	CO	548	CCN(CC)c1ccc2cc(/C=C/C=C3\CCCc4c3[o+]c3cc(N(CC)CC)ccc3c4-c3ccccc3C(=O)O)c(=O)oc2c1	CO	630
CC[N+]1=c2cc3c(cc2Sc2ccccc21)=C(c1c(F)c(F)c(F)c(F)c1C(=O)[O-	CC(OCC)=O	639	CC1(C)C2=CC(=N)C=CC2=C(c2ccccc2C(=O)O)c2ccc(N)cc21	O	552	CCN(CC)c1ccc2cc(/C=C/C=C/C=C3\CCCc4c3[o+]c3cc(N(CC)CC)c	CO	652

])c1cc2c4c(c1O3)C(C)(C)CCN4CCC2(C)C						cc3c4-c3ccccc3C(=O)O)c(=O)oc2c1		
CC[N+]1=c2cc3c(cc2Sc2ccccc21)=C(c1c(F)c(F)c(F)c(F)c1C(=O)[O-])c1cc2c4c(c1O3)C(C)(C)CCN4CCC2(C)C	CCO	622	CN(C)c1ccc2c(c1)C(C)(C)C1=CC(=[N+](C)C)C=CC1=C2c1ccccc1C(=O)O	CCO	612	CCN(CC)c1ccc2c(c1)oc(=O)c1c(-c3ccccc3C(=O)O)c3ccc(=[N+](CC)CC)cc-3oc12	CCO	591
CC[N+]1=c2cc3c(cc2Sc2ccccc21)=C(c1c(F)c(F)c(F)c(F)c1C(=O)[O-])c1cc2c4c(c1O3)C(C)(C)CCN4CCC2(C)C	O	613	CC1(C)C2=CC(=[N+]3CCCC3)C=CC2=C(c2ccccc2C(=O)O)c2ccc(N3CCCC3)cc21	O	613	CC[N+](CC)=c1ccc2c(-c3ccccc3C(=O)O)c3c(=O)oc4ccccc4c3oc-2c1	CCO	598
CC[N+]1=c2cc3c(cc2Sc2ccccc21)=C(c1c(F)c(F)c(F)c(F)c1C(=O)[O-])c1cc2c4c(c1O3)C(C)(C)CCN4CCC2(C)C	CC#N	616	CN(C)c1ccc2c(c1)[Ge](C)(C)C1=CC(=[N+](C)C)C=CC1=C2	O	621	CC[N+](CC)=c1ccc2c(-c3ccccc3C(=O)O)c3c(=O)oc4c5c6c(cc4c3oc-2c1)CCCN6CCC5	CCO	612
CC[N+]1=c2cc3c(cc2Sc2ccccc21)=C(c1c(F)c(F)c(F)c(F)c1C(=O)[O-])c1cc2c4c(c1O3)C(C)(C)CCN4CCC2(C)C	CO	623	CN(C)c1ccc2cc3ccc(N(C)C)cc3nc2c1	O	493	COC(=O)c1ccccc1-c1c2ccc(N)cc2oc2c1ccc1ccc(=O)c12	O	542
CC[N+]1=c2cc3c(cc2Sc2ccccc21)=C(c1c(F)c(F)c(F)c(F)c1C(=O)[O-])c1cc2c4c(c1O3)C(C)(C)CCN4CCC2(C)C	C1CCCO1	631	Cc1ccccc1-c1c2ccc(=[N+](C)C)cc-2oc2cc(N(C)C)ccc12	O	550	COC(=O)c1ccccc1-c1c2ccc(=N)cc-2oc2c1ccc1ccc(OC)c12	O	538
CC[N+]1=c2cc3c(cc2Sc2ccccc21)=C(c1c(F)c(F)c(F)c(F)c1C(=O)[O-])c1cc2c4c(c1O	C1COCCO1	637	CN(C)c1ccc2c(c1)[B-](O)(O)C1=CC(=[N+](C)C)C=CC1=C2	O	611	COC(=O)c1ccccc1-c1c2ccc(=[N+](C)C)cc-2oc2c1ccc1ccc(O)c12	O	567

3)C(C)(C)CCN4CCC2(C)C								
C=CC(=O)Nc1ccc(-c2c3ccc(=[N+](CC)CC)cc-3oc3cc(N(CC)CC)ccc23)c(C(=O)O)c1	O	558	Cc1ccccc1C1=C2C=CC(=[N+](C)C)C=C2[B-](O)(O)c2cc(N(C)C)ccc21	O	620	COC(=O)c1ccccc1-c1c2ccc(=[N+](C)C)cc-2oc2c1ccc1cccc(OC)c12	O	568
CCCCNc1ccc(-c2c3ccc(=[N+](CC)CC)cc-3oc3cc(N(CC)CC)ccc23)c(C(=O)O)c1	O	558	CN(C)c1ccc2c(c1)C(C)(C)C1=CC(=[N+](C)C)C=CC1=C2c1ccccc1C(=O)[O-]	O	606	O=C([O-])c1ccccc1C1=c2cc3c4c(c2Oc2c1ccc1cccc(O)c21)CCC[N+]=4CCC3	O	585
CCCCCCCCNc1ccc(-c2c3ccc(=[N+](CC)CC)cc-3oc3cc(N(CC)CC)ccc23)c(C(=O)O)c1	O	563	CC1(C)C2=CC(=[N+]3CCC3)C=CC2=C(c2ccccc2C(=O)[O-])c2ccc(N3CCC3)cc21	O	608	COc1cccc2ccc3c(c12)Oc1c2c4c(cc1=C3c1ccccc1C(=O)[O-])CCC[N+]=4CCC2	O	582
CCN(CC)c1ccc2c(-c3ccc(N4CCCCCC4)cc3C(=O)O)c3ccc(=[N+](CC)CC)cc-3oc2c1	O	558	CC1(C)C2=CC(=[N+]3CCCC3)C=CC2=C(c2ccccc2C(=O)[O-])c2ccc(N3CCCC3)cc21	O	613	CC[N+](CC)=c1ccc2c(-c3ccccc3C(=O)O)c3c(ccc4ccc(O)cc43)oc-2c1	O	560
CCN(CC)c1ccc2c(-c3ccccc3C(=O)NCCN3CCOCC3)c3ccc(=[N+](CC)CC)cc-3oc2c1	O	563	CN(C)c1ccc2c(c1)C(C)(C)C1=CC(=[N+](C)C)C=CC1=C2C#Cc1ccccc1	O	680	COC(=O)c1ccccc1-c1c2ccc(N)cc2oc2c1ccc1cccc(=N)c12	O	576
CCN(CC)c1ccc2c(-c3ccccc3CNC3CC3)c3ccc(=[N+](CC)CC)cc-3oc2c1	O	565	CN=C1C=CC2=C(c3cc(C(=O)O)ccc3C(=O)O)c3ccc(NC)cc3C(C)(C)C2=C1	O	582	COC(=O)c1ccccc1-c1c2ccc(=[N+](C)C)cc-2oc2c1ccc1cccc(N)c12	O	598
CCN(CC)c1ccc2c(-c3ccccc3CNC3CCC3)c3ccc(=[N+](CC)CC)cc-3oc2c1	O	558	CN(C)c1ccc2c(c1)C(C)(C)C1=CC(=[N+](C)C)C=CC1=C2c1cc(C(=O)O)ccc1C(=O)[O-]	O	609	COC(=O)c1ccccc1C1=c2cc3c4c(c2Oc2c1ccc1cccc(N)c21)CCC[N+]=4CCC3	O	601
CCN(CC)c1ccc2c(-	O	568	CN(C)c1ccc2c(c1)C(C)(C)	O	617	N=c1ccc2c(-c3ccccc3C(=O	O	533

c3ccccc3CNC3CCCC3)c3ccc(=[N+](CC)CC)cc-3oc2c1			C1=CC(=[N+](C)C)C(F)=CC1=C2c1cc(C(=O)O)ccc1C(=O)[O-]			)O)c3ccc4cc(N)ccc4c3oc-2c1		
CCN(CC)c1ccc2c(-c3ccccc3CNC3CCCCC3)c3ccc(=[N+](CC)CC)cc-3oc2c1	O	566	CN(C)c1cc2c(cc1F)C(c1cc(C(=O)O)ccc1C(=O)[O-])=C1C=C(F)C(=[N+](C)C)C=C1C2(C)C	O	628	C[N+](C)=c1ccc2c(-c3ccccc3C(=O)[O-])c3ccc4cc(N)ccc4c3oc-2c1	O	564
CCN=c1cc2oc3cc(NCC)c(C)cc3c(-c3ccccc3CNC3CC3)c-2cc1C	O	532	CC1(C)C2=CC(=NCC(F)(F)F)C=CC2=C(c2cc(C(=O)O)ccc2C(=O)O)c2ccc(NCC(F)(F)F)cc21	O	561	CN(C)c1ccc2c(ccc3c(-c4ccccc4C(=O)O)c4ccc(=N)cc-4oc32)c1	O	568
CCN=c1cc2oc3cc(NCC)c(C)cc3c(-c3ccccc3CNC3CCC3)c-2cc1C	O	529	CC1(C)C2=CC(=NCC(F)(F)CO)C=CC2=C(c2cc(C(=O)O)ccc2C(=O)O)c2ccc(NCC(F)(F)CO)cc21	O	571	Nc1ccc2c3c(ccc2c1)C(c1ccccc1C(=O)[O-])=c1cc2c4c(c1O3)CCC[N+]=4CCC2	O	577
CCN=c1cc2oc3cc(NCC)c(C)cc3c(-c3ccccc3CNC3CCCC3)c-2cc1C	O	532	CC1(C)C2=CC(=[N+]3CCCCCC3)C=CC2=C(c2ccccc2C(=O)[O-])c2ccc(N3CCCCCC3)cc21	O	618	CNc1ccc2c3c(ccc2c1)C(c1ccccc1C(=O)[O-])=c1cc2c4c(c1O3)CCC[N+]=4CCC2	O	585
CCN=c1cc2oc3cc(NCC)c(C)cc3c(-c3ccccc3CNC3CCCCC3)c-2cc1C	O	533	CC1(C)C2=CC(=[N+]3CC(F)(F)C3)C=CC2=C(c2ccccc2C(=O)[O-])c2ccc(N3CC(F)(F)C3)cc21	O	585			

Table S8. The labeled λ_{emi} of rhodamine derivatives.

SMILES /Rhodamine	Solvent/SMILES	λ_{emi}	SMILES /Rhodamine	Solvent/SMILES	λ_{emi}	SMILES /Rhodamine	Solvent/S	λ_{emi}

							MIL ES	
N=c1ccc2c(-c3ccccc3CO)c3ccc(N)cc3oc-2c1	O	524	CCN(CC)c1ccc2c(-c3ccccc3CNC3CCCCC3)c3ccc(=[N+](CC)CC)cc-3oc2c1	O	585	CCN=c1ccc2c(-c3ccccc3C(=O)O)c3ccc(NCC)cc3oc-2c1	O	577
CC(C)CC(N)C(=O)Nc1ccc2c(c1)Oc1cc(N)ccc1C21OCc2ccccc21	O	525	CCN=c1cc2oc3cc(NCC)c(C)cc3c(-c3ccccc3CNC3CC3)c-2cc1C	O	552	CCN=c1cc2oc3cc(NCC)cc3c(-c3ccccc3C(=O)O)c-2cc1C	O	566
CC(=O)Nc1ccc2c(c1)Oc1cc(N)ccc1C21OCc2ccccc21	O	526	CCN=c1cc2oc3cc(NCC)c(C)cc3c(-c3ccccc3CNC3CCC3)c-2cc1C	O	554	O=C([O-])c1ccccc1-c1c2ccc(=[N+]3CCCC3)cc-2oc2cc(N3CCCC3)ccc12	O	576
C[N+](C)=c1ccc2c(-c3ccccc3CO)c3ccc(N)cc3oc-2c1	O	552	CCN=c1cc2oc3cc(NCC)c(C)cc3c(-c3ccccc3CNC3CCCC3)c-2cc1C	O	554	O=C([O-])c1ccccc1-c1c2ccc(=[N+]3CCCCC3)cc-2oc2cc(N3CCCCC3)ccc12	O	586
CC(=O)Nc1ccc2c(c1)Oc1cc(N(C)C)ccc1C21OCc2ccccc21	O	571	CCN=c1cc2oc3cc(NCC)c(C)cc3c(-c3ccccc3CNC3CCCCC3)c-2cc1C	O	554	O=C([O-])c1ccccc1-c1c2ccc(=[N+]3CCCCCC3)cc-2oc2cc(N3CCCCCC3)ccc12	O	583
CC[N+](CC)=c1ccc2c(-c3ccccc3CO)c3ccc(N)cc3oc-2c1	O	555	CCN(CC)c1ccc2c(-c3ccccc3C(=O)NCCNc3ncc(C(=O)OC)c(Nc4ccccc4)n3)c3ccc(=[N+](CC)CC)cc-3oc2c1	ClCCl	460	O=C(O)c1ccccc1C1=c2cc3c(cc2Oc2cc4c(cc21)CCCN4)=NCCC3	O	588
CCN(C)c1ccc2c(c1)Oc1cc(NC(C)=O)ccc1C21OCc2ccccc21	O	573	CCN(CC)c1ccc2c(-c3ccccc3C(=O)NCCNc3ncc(C(=O)OC)c(Nc4ccccc4)n3)c3ccc(=[N	CC(OCC)=O	456	O=C([O-])c1ccccc1C1=c2cc3c4c(c2Oc2c1cc1c5c2CCCN5CCC1)CCC[N+]=4CCC3	O	600

			+](CC)CC)cc-3oc2c1					
CC(=O)NCC(=O)N1CCCC1C(=O)Nc1ccc2c(c1)Oc1cc(N)ccc1C21OCc2ccccc21	O	527	CCN(CC)c1ccc2c(-c3ccccc3C(=O)NCCNc3ncc(C(=O)OC)c(Nc4ccccc4)n3)c3ccc(=[N+](CC)CC)cc-3oc2c1	C1CCCO1	451	CCN(CC)c1ccc2c(c1)Oc1c3c4c(cc1=C2c1ccccc1C(=O)[O-])CCC[N+]=4CCC3	O	587
Nc1ccc2c(c1)Oc1cc(NC(=O)O[C@@H]3C[C@H](CO)[C@H](O)[C@H](O)[C@@H]3O)ccc1C21OCc2ccccc21	O	525	CCN(CC)c1ccc2c(-c3ccccc3C(=O)NCCNc3ncc(C(=O)OC)c(Nc4ccccc4)n3)c3ccc(=[N+](CC)CC)cc-3oc2c1	CS(C)=O	491	CCNc1cc2c(cc1C)C(c1ccccc1C(=O)[O-])=c1cc3c4c(c1O2)CCC[N+]=4CCC3	O	580
CC[N+](CC)=c1ccc2c(-c3ccccc3CO)c3ccc([O-])cc3oc-2c1	O	543	CCN(CC)c1ccc2c(-c3ccccc3C(=O)NCCNc3ncc(C(=O)OC)c(NCc4ccccc4)n3)c3ccc(=[N+](CC)CC)cc-3oc2c1	ClCCl	456	CCN1c2cc3oc4cc(=N)ccc-4c(-c4ccccc4C(=O)O)c3cc2N(CC)C2CCCCC21	CCO	648
CN(C)c1ccc2c(-c3ccccc3CO)c3ccc(=[N+](C)C)cc-3oc2c1	O	574	CCN(CC)c1ccc2c(-c3ccccc3C(=O)NCCNc3ncc(C(=O)OC)c(NCc4ccccc4)n3)c3ccc(=[N+](CC)CC)cc-3oc2c1	CC(OCC)=O	454	CC/N=c1/cc2oc3cc4c(cc3c(-c3ccccc3C(=O)O)c-2cc1C)N(CC)C1CCCCC1N4CC	CCO	646
CN(C)c1ccc2c(-c3c(CO)cccc3COc3ccc(N)cc3)c3ccc(=[N+](C)C)cc-3oc2c1	O	574	CCOC(C)=O	C1CCCO1	449	CCN1c2cc3oc4cc(=[N+](CC)CC)ccc-4c(-c4ccccc4C(=O)[O-])c3cc2N(CC)C2CCCCC21	CCO	660
CN(C)c1ccc2c(-c3c(CO)cccc3COc3ccc(O)cc3)c	O	574	CCN(CC)c1ccc2c(-c3ccccc3C(=O)NCCNc3ncc(C(=O)OC)	CS(C)=O	489	CCN1c2cc3c(cc2N(CC)C2CCCCC21)C(c1ccccc1C(=O)[O-	CCO	587

3ccc(=[N+](C)C)cc-3oc2c1			c(NCc4ccccc4)n3)c3ccc(=[N+](CC)CC)cc-3oc2c1			])=c1cc2c4c(c1O3)CCC[N+]=4CCC2		
C[N+](C)=c1ccc2c(-c3ccccc3CO)c3cc(F)c(N)cc3oc-2c1	O	559	CCN(CC)c1ccc2c(-c3ccccc3C(=O)NCCNc3ncc(C(=O)OC)c(NCCc4ccccc4)n3)c3ccc(=[N+](CC)CC)cc-3oc2c1	ClCCl	456	N=c1ccc2c(-c3ccc(C(=O)O)cc3C(=O)O)c3ccc(N)c(S(=O)(=O)O)c3oc-2c1S(=O)(=O)O	O	519
C[N+](C)=c1ccc2c(-c3ccccc3CO)c3cc(Cl)c(N)cc3oc-2c1	O	559	CCN(CC)c1ccc2c(-c3ccccc3C(=O)NCCNc3ncc(C(=O)OC)c(NCCc4ccccc4)n3)c3ccc(=[N+](CC)CC)cc-3oc2c1	CC(OCC)=O	453	CC1CC(C)(C)N=c2c1cc1c(c2S(=O)(=O)O)Oc2c(ccc(N)c2S(=O)(=O)O)C=1c1ccc(C(=O)O)cc1C(=O)O	O	540
CC(=O)Nc1cc2c(cc1F)C1(OCc3ccccc31)c1ccc(N(C)C)cc1O2	O	582	CCN(CC)c1ccc2c(-c3ccccc3C(=O)NCCNc3ncc(C(=O)OC)c(NCCc4ccccc4)n3)c3ccc(=[N+](CC)CC)cc-3oc2c1	C1CCCO1	448	CC1N=c2c(cc3c(c2S(=O)(=O)O)Oc2c(cc4c(c2S(=O)(=O)O)NC(C)C4(C)C)C=3c2ccc(C(=O)O)cc2C(=O)O)C1(C)C	O	554
CC(=O)Nc1cc2c(cc1Cl)C1(OCc3ccccc31)c1ccc(N(C)C)cc1O2	O	581	CCN(CC)c1ccc2c(-c3ccccc3C(=O)NCCNc3ncc(C(=O)OC)c(NCCc4ccccc4)n3)c3ccc(=[N+](CC)CC)cc-3oc2c1	CS(C)=O	489	CC1(C)C=C(CS(=O)(=O)O)c2cc3c(c(S(=O)(=O)O)c2=N1)Oc1c(cc2c(c1S(=O)(=O)O)NC(C)(C)C=C2CS(=O)(=O)O)C=3c1ccc(C(=O)O)cc1C(=O)O	O	603
CC[N+](CC)=c1ccc2c(-c3ccccc3CO)c3cc(F)c(N)cc3oc-2c1	O	566	CCN(CC)c1ccc2c(-c3ccccc3C(=O)NCCNc3ncc(C(=O)OC)c(NCCc4ccc(OC)c(OC)c4)n3)c3ccc(=[N	ClCCl	456	CN1c2c(cc3c(c2S(=O)(=O)O)Oc2c(S(=O)(=O)[O-])c4c(cc2=C3c2ccc(C(=O)O)cc2C(=O)O)C(CS(=O)(=O)O)=CC(C)(C)	O	617

			+](CC)CC)cc-3oc2c1			[N+]=4C)C(CS(=O)(=O)O)=CC1(C)C		
CC[N+](CC)=c1ccc2c(-c3ccccc3CO)c3cc(Cl)c(N)cc3oc-2c1	O	568	CCN(CC)c1ccc2c(-c3ccccc3C(=O)NCCNc3ncc(C(=O)OC)c(NCCc4ccc(OC)c(OC)c4)n3)c3ccc(=[N+](CC)CC)cc-3oc2c1	CC(OCC)=O	453	Nc1ccc2c(c1)Cc1c-2[o+]c2ccccc2c1-c1ccccc1C(=O)O	O	548
CCN(CC)c1ccc2c(c1)Oc1cc(NC(C)=O)c(Cl)cc1C21OCc2ccccc21	O	578	CCN(CC)c1ccc2c(-c3ccccc3C(=O)NCCNc3ncc(C(=O)OC)c(NCCc4ccc(OC)c(OC)c4)n3)c3ccc(=[N+](CC)CC)cc-3oc2c1	C1CCCO1	449	COc1ccc2c(-c3ccccc3C(=O)O)c3c([o+]c2c1)-c1ccc(N)cc1C3	O	550
CCN(CC)c1ccc2c(c1)Oc1cc(NC(C)=O)c(F)cc1C21OCc2ccccc21	O	583	CCN(CC)c1ccc2c(-c3ccccc3C(=O)NCCNc3ncc(C(=O)OC)c(NCCc4ccc(OC)c(OC)c4)n3)c3ccc(=[N+](CC)CC)cc-3oc2c1	CS(C)=O	489	Nc1ccc2c(c1)Cc1c-2[o+]c2cc(O)ccc2c1-c1ccccc1C(=O)O	O	582
Nc1ccc2c(c1)Oc1c3c4c(cc1=C2c1ccccc1CO)CCC[N+]=4CCC3	O	569	CCN(CC)c1ccc2c(-c3ccccc3C(=O)N3CCN(C)CC3)c3ccc(=[N+](CC)CC)cc-3oc2c1	O	588	CN(C)c1ccc2c(-c3ccccc3C(=O)O)c3c([o+]c2c1)-c1ccc(N)cc1C3	O	608
Nc1cc2c(cc1F)C(c1ccccc1CO)=c1cc3c4c(c1O2)CCC[N+]=4CCC3	O	582	CCN(CC)c1ccc2c(-c3ccccc3C(=O)N3CCN(C)CC3)c3ccc(=[N+](CC)CC)cc-3oc2c1	CO	585	Nc1ccc2c(c1)Cc1c-2[o+]c2cc(Cl)ccc2c1-c1ccccc1C(=O)O	O	554
Nc1cc2c(cc1Cl)C(c1ccccc1CO)=c1cc3c4c(c1O	O	582	CCN(CC)c1ccc2c(-c3ccccc3C(=O)N3CCN(C	CCO	586	Nc1ccc2c(c1)Cc1c-2[o+]c2cc3c4c(c2c1-	O	632

2)CCC[N+]=4CCC3			)CC3)c3ccc(=[N+](CC)CC)cc-3oc2c1			c1ccccc1C(=O)O)CCCN4CCC3		
OCc1ccccc1-c1c2ccc(=NCC(F)(F)F)cc-2oc2cc(NCC(F)(F)F)ccc12	O	526	CCN(CC)c1ccc2c(-c3ccccc3C(=O)N3CCN(C)CC3)c3ccc(=[N+](CC)CC)cc-3oc2c1	CCCO	585	CCN(CC)c1ccc2c(-c3ccccc3C(=O)O)c3c([o+]c2c1)-c1ccc(N)cc1C3	O	619
O=C(O)c1ccc(-c2c3ccc(=[N+](CC(F)(F)F)CC(F)(F)F)cc-3oc3cc(N(CC(F)(F)F)CC(F)(F)F)ccc23)c(CO)c1	O	532	CCN(CC)c1ccc2c(-c3ccccc3C(=O)N3CCN(C)CC3)c3ccc(=[N+](CC)CC)cc-3oc2c1	CC(O)C	585	CCN(CC)c1ccc2c(-c3ccccc3C(=O)O)c3c([o+]c2c1)-c1ccc(Cl)cc1C3	CCO	608
O=C(O)c1ccc(-c2c3ccc(=[N+](CC(F)(F)F)CC(F)(F)F)cc-3oc3cc(N(CC(F)(F)F)CC(F)(F)F)ccc23)c(CCO)c1	O	530	CCN(CC)c1ccc2c(-c3ccccc3C(=O)N3CCN(C)CC3)c3ccc(=[N+](CC)CC)cc-3oc2c1	CC#N	584	CCN(CC)c1ccc2c(-c3ccccc3C(=O)O)c3c([o+]c2c1)-c1ccccc1C3	CCO	602
CC(=O)Nc1ccc2c(c1)Oc1c3c4c(cc1=C2c1ccccc1CO)CCC[N+]=4CCC3	O	589	CCN(CC)c1ccc2c(-c3ccccc3C(=O)N3CCN(C)CC3)c3ccc(=[N+](CC)CC)cc-3oc2c1	CS(C)=O	594	CCN(CC)c1ccc2c(-c3ccccc3C(=O)O)c3c([o+]c2c1)-c1ccc(OC)cc1C3	CCO	596
CC(=O)Nc1cc2c(cc1F)C(c1ccccc1CO)=c1cc3c4c(c1O2)CCC[N+]=4CCC3	O	599	CCN(CC)c1ccc2c(-c3ccccc3C(=O)N3CCN(C)CC3)c3ccc(=[N+](CC)CC)cc-3oc2c1	CN(C=O)C	589	CCN(CC)c1ccc2c(-c3ccccc3C(=O)O)c3c([o+]c2c1)-c1ccc(O)cc1C3	CCO	604
CC(=O)Nc1cc2c(cc1Cl)C(c1ccccc1CO)=c1cc3c4c(c1O2)CCC[N+]=4CCC3	O	592	CCN(CC)c1ccc2c(-c3ccccc3C(=O)N3CCN(C)CC3)c3ccc(=[N+](CC)CC)cc-3oc2c1	ClCCl	585	CCN(CC)c1ccc2c(-c3ccccc3C(=O)O)c3c([o+]c2c1)-c1ccc(NC)cc1C3	CCO	613
CN(C)c1ccc2c(c1)[Si](C)(C)C1=CC(=[N+](C)C)C=CC1=C2c	O	671	CCN(CC)c1ccc2c(-c3ccccc3C(=O)N3CCN(C	ClC(Cl)Cl	581	CCN(CC)c1ccc2c(c1)Cc1c-2[o+]c2cc(N(CC)CC)ccc2c	CCO	639

1ccc(C(=O)O)cc1CO			)CC3)c3ccc(=[N+](CC)CC)cc-3oc2c1			1-c1ccccc1C(=O)O		
C[Si]1(C)c2cc3c(cc2C(c2ccccc2C(=O)O)=c2cc4c(cc21)=NCCC4)CCCN3	O	654	CCN(CC)c1ccc2c(-c3ccccc3C(=O)N3CCN(C)CC3)c3ccc(=[N+](CC)CC)cc-3oc2c1	C1COCCO1	587	CN(C)c1ccc2c(c1)Cc1c-2[o+]c2cc3c4c(c2c1-c1ccccc1C(=O)O)CCCN4CCC3	O	640
N=C1C=CC2=C(c3ccccc3C(=O)O)c3ccc(N)cc3P(=O)(O)C2=C1	CO	655	CCN(CC)CCN(C)C(=O)c1ccccc1-c1c2ccc(=[N+](CC)CC)cc-2oc2cc(N(CC)CC)ccc12	O	590	CCN(CC)c1ccc2c(c1)Cc1c-2[o+]c2cc3c4c(c2c1-c1ccccc1C(=O)O)CCCN4CCC3	O	642
CCN(CC)c1ccc2c(c1)P(=O)([O-])C1=CC(=[N+](CC)CC)C=CC1=C2c1ccccc1C(=O)O	CO	690	CCN(CC)CCN(C)C(=O)c1ccccc1-c1c2ccc(=[N+](CC)CC)cc-2oc2cc(N(CC)CC)ccc12	CO	586	CCN(CC)c1ccc2c(-c3ccccc3C(=O)O)c3c([o+]c2c1)-c1cccc(O)c1C3	O	576
CCN1c2cc3c(cc2C(C)=CC1(C)C)C(c1ccccc1C(=O)O)=c1cc2c(cc1P3(=O)[O-])=[N+](CC)C(C)(C)C=C2C	CO	732	CCN(CC)CCN(C)C(=O)c1ccccc1-c1c2ccc(=[N+](CC)CC)cc-2oc2cc(N(CC)CC)ccc12	CCO	589	CCN(CC)c1ccc2c(-c3ccccc3C(=O)O)c3c([o+]c2c1)-c1ccc(O)cc1C3	O	604
CN=C1C=CC2=C(c3ccccc3C(=O)O)c3ccc(NC)cc3P(=O)(O)C2=C1	CO	661	CCN(CC)CCN(C)C(=O)c1ccccc1-c1c2ccc(=[N+](CC)CC)cc-2oc2cc(N(CC)CC)ccc12	CCCO	586	CCN(CC)c1ccc2c(-c3ccccc3C(=O)O)c3c([o+]c2c1)-c1cc(O)ccc1C3	O	573
CN=C1C=CC2=C(c3ccccc3S(=O)(=O)O)c3ccc(NC)cc3P(=O)(O)C2=C1	CO	691	CCN(CC)CCN(C)C(=O)c1ccccc1-c1c2ccc(=[N+](CC)CC)cc-2oc2cc(N(CC)CC)ccc12	CC(O)C	585	CCN(CC)c1ccc2c(-c3ccccc3C(=O)O)c3c([o+]c2c1)-c1c(O)cccc1C3	O	597
CN(C)c1ccc2c(c1)Oc1cc(N(C)C)ccc1C21c2ccccc2S(=O)(=O)N1CCCC(=O)	O	580	CCN(CC)CCN(C)C(=O)c1ccccc1-c1c2ccc(=[N+](CC)CC)cc-	CC#N	584	CCN(CC)c1ccc2c(-c3ccccc3C(=O)O)c3c([o+]c2c1)-	O	627

NCCOCCOCCCCCCCl			2oc2cc(N(CC)CC)ccc12			c1ccc(N)cc1CC3		
CN(C)c1ccc2c(c1)Oc1cc(N(C)C)ccc1C21c2ccccc2S(=O)(=O)N1CCCC(=O)NCCOCCOCCCCCCCl	CCO	580	CCN(CC)CCN(C)C(=O)c1ccccc1-c1c2ccc(=[N+](CC)CC)cc-2oc2cc(N(CC)CC)ccc12	CS(C)=O	593	O=S(=O)([O-])c1ccccc1-[c+]1c2ccc(N3C4CCC3CC4)cc2oc2cc(N3[C@H]4CC[C@@H]3CC4)ccc21	CO	567
CN(C)c1ccc2c(c1)Oc1cc(N(C)C)ccc1C21c2ccccc2S(=O)(=O)N1CCCC(=O)NCCOCCOCCCCCCCl	OCC(O)CO	586	CCN(CC)CCN(C)C(=O)c1ccccc1-c1c2ccc(=[N+](CC)CC)cc-2oc2cc(N(CC)CC)ccc12	CN(C=O)C	590	CN(C)c1ccc2c(c1)oc1cc(N(C)C)ccc1[c+]2-c1ccccc1S(=O)(=O)[O-]	CO	568
CCN(CC)c1ccc2c(c1)Oc1cc(N(CC)CC)ccc1C21c2ccccc2S(=O)(=O)N1CCCC(=O)NCCOCCOCCCCCCCl	O	582	CCN(CC)CCN(C)C(=O)c1ccccc1-c1c2ccc(=[N+](CC)CC)cc-2oc2cc(N(CC)CC)ccc12	ClCCl	583	CN1CC2CCCN2c2cc3c(cc21)C(c1ccccc1C(=O)O)=c1cc2c4c(c1O3)CCC[N+]=4CCC2	O	644
CCN(CC)c1ccc2c(c1)Oc1cc(N(CC)CC)ccc1C21c2ccccc2S(=O)(=O)N1CCCC(=O)NCCOCCOCCCCCCCl	CCO	582	CCN(CC)CCN(C)C(=O)c1ccccc1-c1c2ccc(=[N+](CC)CC)cc-2oc2cc(N(CC)CC)ccc12	ClC(Cl)Cl	585	CCN(CC)c1ccc2c(c1)Oc1cc3c(cc1=C2c1ccccc1C(=O)O)Sc1ccccc1[N+]=3CC	CCO	730
CCN(CC)c1ccc2c(c1)Oc1cc(N(CC)CC)ccc1C21c2ccccc2S(=O)(=O)N1CCCC(=O)NCCOCCOCCCCCCCl	OCC(O)CO	587	CCN(CC)CCN(C)C(=O)c1ccccc1-c1c2ccc(=[N+](CC)CC)cc-2oc2cc(N(CC)CC)ccc12	C1COCCO1	582	CN(C)c1cc2oc3cc(=[N+](C)C)c(-c4ccccc4)cc-3c(-c3ccccc3C(=O)[O-])c2cc1-c1ccccc1	CO	571
O=C(CCCN1C2(c3ccc(N4CCCC4)cc3Oc3cc(N4CCCC4)ccc32)c2ccccc2S1(=O)=O)NCCOCCOCCCCCCCl	O	588	CCN(CC)c1ccc2c(-c3ccccc3C(=O)N3C[C@@H](C)O[C@@H](C)C3)c3ccc(=[N+](CC)CC)cc-3oc2c1	O	591	CN1c2cc3c(cc2-c2ccccc2C1(C)C)C(c1ccccc1C(=O)[O-])=c1cc2c(cc1O3)=[N+](C)C(C)(C)c1ccccc1-2	CO	604
O=C(CCCN1C2(c3ccc(N4CCCC4)cc3Oc3cc(	CCO	586	CCN(CC)c1ccc2c(-c3ccccc3C(=	CO	586	CN1c2cc3c(cc2-c2ccsc2C1(C)	CO	605

N4CCCC4)ccc32)c2ccccc2S1(=O)=O)NCCOCCOCCCCCCCl			O)N3C[C@@H](C)O[C@@H](C)C3)c3ccc(=[N+](CC)CC)cc-3oc2c1			C)C(c1ccccc1C(=O)[O-])=c1cc2c(cc1O3)=[N+](C)C(C)(C)c1sccc1-2		
O=C(CCCN1C2(c3ccc(N4CCCCC4)cc3Oc3cc(N4CCCCC4)ccc32)c2ccccc2S1(=O)=O)NCCOCCOCCCCCCCl	O	596	CCN(CC)c1ccc2c(-c3ccccc3C(=O)N3C[C@@H](C)O[C@@H](C)C3)c3ccc(=[N+](CC)CC)cc-3oc2c1	CCO	590	CN1c2cc3c(cc2-c2sccc2C1(C)C)C(c1ccc(C(=O)O)cc1C(=O)[O-])=c1cc2c(cc1O3)=[N+](C)C(C)(C)c1ccsc1-2	CO	620
O=C(CCCN1C2(c3ccc(N4CCCCC4)cc3Oc3cc(N4CCCCC4)ccc32)c2ccccc2S1(=O)=O)NCCOCCOCCCCCCCl	CCO	592	CCN(CC)c1ccc2c(-c3ccccc3C(=O)N3C[C@@H](C)O[C@@H](C)C3)c3ccc(=[N+](CC)CC)cc-3oc2c1	CCCO	587	CN1c2cc3c(cc2-c2sc4ccccc4c2C1(C)C)C(c1ccc(C(=O)O)cc1C(=O)[O-])=c1cc2c(cc1O3)=[N+](C)C(C)(C)c1c-2sc2ccccc12	CO	627
C[Si]1(C)c2cc(N3CCC3)ccc2C2(OCc3cc(C(=O)O)ccc32)C2C=CC(N3CCC3)=CC21	N#CC	670	CCN(CC)c1ccc2c(-c3ccccc3C(=O)N3C[C@@H](C)O[C@@H](C)C3)c3ccc(=[N+](CC)CC)cc-3oc2c1	CC(O)C	588	CN1c2cc3c(cc2-c2sccc2C1(C)C)C(c1c(Cl)c(Cl)c(Cl)c(Cl)c1C(=O)[O-])=c1cc2c(cc1O3)=[N+](C)C(C)(C)c1ccsc1-2	CO	648
C[Si]1(C)c2cc(N3CCC3)ccc2C2(OCc3cc(C(=O)O)ccc32)C2C=CC(N3CCC3)=CC21	ClC(Cl)Cl	668	CCN(CC)c1ccc2c(-c3ccccc3C(=O)N3C[C@@H](C)O[C@@H](C)C3)c3ccc(=[N+](CC)CC)cc-3oc2c1	CC#N	585	CN1c2cc3c(cc2-c2sccc2C1(C)C)C(c1c(F)c(F)c(F)c(F)c1C(=O)[O-])=c1cc2c(cc1O3)=[N+](C)C(C)(C)c1ccsc1-2	CO	649
C[Si]1(C)c2cc(N3CCC3)ccc2C2(OCc3cc(C(=O)O)ccc32)C2	CS(C)=O	683	CCN(CC)c1ccc2c(-c3ccccc3C(=O)N3C[C@@H](C)O[C@@	CS(C)=O	595	CN1c2cc3c(cc2-c2ccc4ccccc4c2C1(C)C)C(c1c(Cl)c(Cl)c(	CO	645

C=CC(N3CCC3)=CC21			H](C)C3)c3cc(=[N+](CC)CC)cc-3oc2c1			Cl)c(Cl)c1C(=O)[O-])=c1cc2c(cc1O3)=[N+](C)C(C)(C)c1c-2ccc2ccccc12		
C[Si]1(C)c2cc(N3CCC3)ccc2C2(OCc3cc(C(=O)O)ccc32)C2C=CC(N3CCC3)=CC21	CC(OCC)=O	675	CCN(CC)c1ccc2c(-c3ccccc3C(=O)N3C[C@@H](C)O[C@@H](C)C3)c3cc(=[N+](CC)CC)cc-3oc2c1	CN(C=O)C	590	CN1c2cc3c(cc2-c2sccc2C1(C)C)C(c1ccc(S(=O)(=O)O)cc1S(=O)(=O)[O-])=c1cc2c(cc1O3)=[N+](C)C(C)(C)c1ccsc1-2	CO	629
C[Si]1(C)c2cc(N3CCC3)ccc2C2(OCc3cc(C(=O)O)ccc32)C2C=CC(N3CCC3)=CC21	CCO	689	CCN(CC)c1ccc2c(-c3ccccc3C(=O)N3C[C@@H](C)O[C@@H](C)C3)c3cc(=[N+](CC)CC)cc-3oc2c1	ClCCl	583	CN1c2cc3c(cc2-c2sc4ccccc4c2C1(C)C)C(c1ccc(S(=O)(=O)O)cc1S(=O)(=O)[O-])=c1cc2c(cc1O3)=[N+](C)C(C)(C)c1c-2sc2ccccc12	CO	633
C[Si]1(C)c2cc(N3CCC3)ccc2C2(OCc3cc(C(=O)O)ccc32)C2C=CC(N3CCC3)=CC21	O	669	CCN(CC)c1ccc2c(-c3ccccc3C(=O)N3C[C@@H](C)O[C@@H](C)C3)c3cc(=[N+](CC)CC)cc-3oc2c1	ClC(Cl)Cl	585	CN1c2cc3c(cc2-c2sc4ccccc4c2C1(C)c1ccccc1)C(c1ccc(S(=O)(=O)O)cc1S(=O)(=O)[O-])=c1cc2c(cc1O3)=[N+](C)C(C)(c1ccccc1)c1c-2sc2ccccc12	CO	638
C[N+]1(C)CCN(C2=CC3Oc4cc(N5CC[N+](C)(C)CC5)ccc4C4(OCc5ccccc54)C3C=C2)CC1	N#CC	558	CCN(CC)c1ccc2c(-c3ccccc3C(=O)N3C[C@@H](C)O[C@@H](C)C3)c3cc(=[N+](CC)CC)cc-3oc2c1	C1COCCO1	580	CN1c2cc3c(cc2-c2c(sc4ccccc24)C1(C)c1ccccc1)C(c1ccc(S(=O)(=O)O)cc1S(=O)(=O)[O-])=c1cc2c(cc1O3)=[N+](C)C(C)(c1ccccc	CO	672

						1)c1sc3ccccc3c1-2		
C[N+]1(C)CCN(C2=CC3Oc4cc(N5CC[N+](C)(C)CC5)ccc4C4(OCc5ccccc54)C3C=C2)CC1	CC(OCC)=O	559	CCN(CC)c1ccc2c(-c3ccccc3C(=O)N3CCN(CCO)CC3)c3ccc(=[N+](CC)CC)cc-3oc2c1	O	589	CC1(C)C2=CC(=N)C=CC2=C(c2ccccc2C(=O)O)c2ccc(N)cc21	O	577
C[N+]1(C)CCN(C2=CC3Oc4cc(N5CC[N+](C)(C)CC5)ccc4C4(OCc5ccccc54)C3C=C2)CC1	CCO	558	CCN(CC)c1ccc2c(-c3ccccc3C(=O)N3CCN(CCO)CC3)c3ccc(=[N+](CC)CC)cc-3oc2c1	CO	589	CN(C)c1ccc2c(c1)C(C)(C)C1=CC(=[N+](C)C)C=CC1=C2c1ccccc1C(=O)O	CCO	642
C[N+]1(C)CCN(C2=CC3Oc4cc(N5CC[N+](C)(C)CC5)ccc4C4(OCc5ccccc54)C3C=C2)CC1	O	555	CCN(CC)c1ccc2c(-c3ccccc3C(=O)N3CCN(CCO)CC3)c3ccc(=[N+](CC)CC)cc-3oc2c1	CCO	588	CC1(C)C2=CC(=[N+]3CCCC3)C=CC2=C(c2ccccc2C(=O)O)c2ccc(N3CCCC3)cc21	O	633
CCN(CC)c1ccc2c(c1)[Si](C)(C)c1cc(N(CC)CC)ccc1C21OC(=O)c2ccccc21	O	666	CCN(CC)c1ccc2c(-c3ccccc3C(=O)N3CCN(CCO)CC3)c3ccc(=[N+](CC)CC)cc-3oc2c1	CCCO	586	CCN1CCCc2cc3c(cc21)C(C)(C)c1cc2c(cc1=C3c1ccccc1C(=O)O)CCC[N+]=2CC	CCO	662
O=c1ccc2c(-c3ccccc3CO)c3ccc(NCC(F)(F)F)cc3oc-2c1	O	515	CCN(CC)c1ccc2c(-c3ccccc3C(=O)N3CCN(CCO)CC3)c3ccc(=[N+](CC)CC)cc-3oc2c1	CC(O)C	585	CCN1CCCc2cc3c(cc21)C(C)(C)c1cc2c(cc1=C3c1ccccc1C(=O)O)C(C)=CC(C)(C)[N+]=2CC	CCO	683
Oc1ccc2c(c1)Oc1cc(NCC(F)(F)F)ccc1C21OCc2ccccc21	O	518	CCN(CC)c1ccc2c(-c3ccccc3C(=O)N3CCN(CCO)CC3)c3ccc(=[N+](CC)CC)cc-3oc2c1	CC#N	584	CCN1c2cc3c(cc2C(C)=CC1(C)C)C(c1ccccc1C(=O)O)=c1cc2c(cc1C3(C)C)=[N+](CC)C(C)(C)C=C2C	CCO	700

CC(=O)N[C@H]1[C@@H](Oc2ccc3c(c2)Oc2cc(NCC(F)(F)F)ccc2C3c2ccccc2CO)O[C@H](CO)[C@@H](O)[C@@H]1O	O	519	CCN(CC)c1ccc2c(-c3ccccc3C(=O)N3CCN(CCO)CC3)c3ccc(=[N+](CC)CC)cc-3oc2c1	CS(C)=O	595	CCN1CCCc2cc3c(cc21)C(C)(C)c1cc2c(cc1=C3c1c(Cl)c(Cl)c(Cl)c(Cl)c1C(=O)O)CCC[N+]=2CC	CCO	692
CCN(CC)c1ccc2c(c1)Oc1c(ccc(NC(=O)CCC(N)C(=O)O)c1CF)C21OCc2ccccc21	O	576	CCN(CC)c1ccc2c(-c3ccccc3C(=O)N3CCN(CCO)CC3)c3ccc(=[N+](CC)CC)cc-3oc2c1	CN(C=O)C	590	CCN1CCCc2cc3c(cc21)C(C)(C)c1cc2c(cc1=C3c1c(Cl)c(Cl)c(Cl)c(Cl)c1C(=O)O)C(C)=CC(C)(C)[N+]=2CC	CCO	713
CCN(CC)c1ccc2c(-c3ccccc3CO)c3ccc(=N)c(CO)c-3oc2c1	O	558	CCN(CC)c1ccc2c(-c3ccccc3C(=O)N3CCN(CCO)CC3)c3ccc(=[N+](CC)CC)cc-3oc2c1	ClCCl	582	CCN1c2cc3c(cc2C(C)=CC1(C)C)C(c1c(Cl)c(Cl)c(Cl)c(Cl)c1C(=O)O)=c1cc2c(cc1C3(C)C)=[N+](CC)C(C)(C)C=C2C	CCO	730
CN(C)c1ccc2c(c1)[Si](C)(C)c1cc(O)ccc1C21OC(=O)c2ccccc21	O	630	CCN(CC)c1ccc2c(-c3ccccc3C(=O)N3CCN(CCO)CC3)c3ccc(=[N+](CC)CC)cc-3oc2c1	ClC(Cl)Cl	585	CN(C)c1ccc2c(c1)C(C)(C)C1=CC(=[N+](C)C)C=CC1=C2c1c(Cl)c(Cl)c(Cl)c(Cl)c1C(=O)O	CCO	672
CN(C)c1ccc2c(c1)[Si](C)(C)c1c(ccc(O)c1CO)C21OC(=O)c2ccccc21	O	630	CCN(CC)c1ccc2c(-c3ccccc3C(=O)N3CCN(CCO)CC3)c3ccc(=[N+](CC)CC)cc-3oc2c1	C1COCCO1	587	Cc1ccccc1C1=C2C=CC(=N)C=C2[Si](C)(C)c2cc(N)ccc21	O	613
CCN(CC)c1ccc2c(c1)Oc1c(ccc(O[C@@H]3O[C@H](CO)[C@@H](O)[C@H](O)[C@H]3O)c1CF)C2c1ccccc1CO	O	560	CCN(CC)c1ccc2c(-c3ccccc3C(=O)N3CCN(c4ccccc4O)CC3)c3ccc(=[N+](CC)CC)cc-3oc2c1	O	589	Cc1ccccc1C1=C2C=CC(=[N+](C)C)C=C2[Si](C)(C)c2cc(N(C)C)ccc21	O	660
CCN(CC)c1ccc2c(c1)Oc1c(ccc	O	560	CCN(CC)c1ccc2c(-	CO	582	Cc1ccccc1C1=c2cc3c(cc2[S	O	712

(O[C@@H]3O[C@H](CO)[C@H](O)[C@H](O)[C@H]3O)c1C(F)F)C2c1ccccc1CO			c3ccccc3C(=O)N3CCN(c4ccccc4O)CC3)c3ccc(=[N+](CC)CC)cc-3oc2c1			i](C)(C)c2cc4c(cc21)CCN4C)=[N+](C)CC3		
CCN(CC)c1ccc2c(c1)Oc1cc(O[C@@H]3O[C@H](CO)[C@H](O)[C@H](O)[C@H]3O)ccc1C2c1ccccc1CO	O	536	CCN(CC)c1ccc2c(-c3ccccc3C(=O)N3CCN(c4ccccc4O)CC3)c3ccc(=[N+](CC)CC)cc-3oc2c1	CCO	585	Cc1ccccc1C1=c2cc3c(cc2[Si](C)(C)c2cc4c(cc21)CCCN4C)=[N+](C)CCC3	O	689
CC[N+](CC)=c1ccc2c(-c3ccccc3CO)c3ccc([O-])c(CO)c3oc-2c1	O	553	CCN(CC)c1ccc2c(-c3ccccc3C(=O)N3CCN(c4ccccc4O)CC3)c3ccc(=[N+](CC)CC)cc-3oc2c1	CCCO	581	CC1=CC(C)(C)N(C)c2cc3c(cc21)C(c1ccccc1C)=c1cc2c(cc1[Si]3(C)C)=[N+](C)C(C)(C)C=C2C	O	740
CCN(CC)c1ccc2c(-c3ccccc3CO)c3ccc(=O)c(C=O)c-3oc2c1	O	556	CCN(CC)c1ccc2c(-c3ccccc3C(=O)N3CCN(c4ccccc4O)CC3)c3ccc(=[N+](CC)CC)cc-3oc2c1	CC(O)C	581	C[Si]1(C)c2cc3c(cc2C(c2ccccc2C(=O)O)=c2cc4c(cc21)=NCC=C4)CCCN3	O	654
CCN(CC)c1ccc2c(-c3ccccc3CO)c3ccc(=O)cc-3oc2c1	O	543	CCN(CC)c1ccc2c(-c3ccccc3C(=O)N3CCN(c4ccccc4O)CC3)c3ccc(=[N+](CC)CC)cc-3oc2c1	CC#N	584	CCN(CC)c1ccc2c(c1)[Si](C)(C)C1=CC(=[N+](CC)CC)C=CC1=C2c1ccccc1C(=O)O	O	666
N=c1ccc2c(-c3ccccc3CO)c3ccc(N)cc3[se]c-2c1	O	562	CCN(CC)c1ccc2c(-c3ccccc3C(=O)N3CCN(c4ccccc4O)CC3)c3ccc(=[N+](CC)CC)cc-3oc2c1	CS(C)=O	592	CN(C)c1ccc2c(-c3ccccc3)c3ccc(=[N+](C)C)cc-3sc2c1	CO	599
CC[N+](CC)=c1ccc2c(-c3ccccc3CO)c3ccc(NC(=O)CC	O	602	CCN(CC)c1ccc2c(-c3ccccc3C(=O)N3CCN(c4ccccc4O)CC	CN(C=O)C	588	CN(C)c1ccc2c(-c3ccccc3)c3ccc(=[N+](C)C)cc-3[se]c2c1	CO	608

C(N)C(=O)O)cc3[se]c-2c1			3)c3ccc(=[N+](CC)CC)cc-3oc2c1					
CC[N+](CC)=c1ccc2c(-c3ccccc3CO)c3ccc(N)cc3[se]c-2c1	O	602	CCN(CC)c1ccc2c(-c3ccccc3C(=O)N3CCN(c4ccccc4O)CC3)c3ccc(=[N+](CC)CC)cc-3oc2c1	ClCCl	583	Cc1ccccc1C1=C2C=CC(=[N+](C)C)C=C2[Ge](C)(C)c2cc(N(C)C)ccc21	O	649
CCN(CC)c1ccc2c(c1)[Si](C)(C)c1cc(N(CC)CC)ccc1C21OB(O)c2ccccc21	O	667	CCN(CC)c1ccc2c(-c3ccccc3C(=O)N3CCN(c4ccccc4O)CC3)c3ccc(=[N+](CC)CC)cc-3oc2c1	ClC(Cl)Cl	586	Cc1ccccc1C1=C2C=CC(=[N+](C)C)C=C2P(=O)([O-])c2cc(N(C)C)ccc21	O	685
CCN(CC)c1ccc2c(-c3ccccc3B(O)O)c3ccc(=[N+](CC)CC)cc-3oc2c1	O	576	CCN(CC)c1ccc2c(-c3ccccc3C(=O)N3CCN(c4ccccc4O)CC3)c3ccc(=[N+](CC)CC)cc-3oc2c1	C1COCCO1	581	CCOP1(=O)C2=CC(=[N+](C)C)C=CC2=C(c2ccccc2C)c2ccc(N(C)C)cc21	O	712
CN(C)c1ccc2c(c1)P(=O)(O)c1cc(N(C)C)ccc1C21OC(=O)c2ccccc21	O	690	C=CCN1C(=O)c2ccccc2C2(c3cc(C)c(NCC)cc3Oc3cc(NCC)c(C)cc32)N1CC=C	O	550	Cc1ccccc1C1=c2cc3c4c(c2P(=O)([O-])c2c1cc1c5c2CCCN5CCC1)CCC[N+]=4CCC3	O	722
CCN(CC)c1ccc2c(c1)P(=O)(O)c1cc(N(CC)CC)ccc1C21OC(=O)c2ccccc21	O	695	CCN(CC)c1ccc2c(c1)Oc1cc(N(CC)CC)ccc1C21c2ccccc2C(=O)N1N	O	543	CCOP1(=O)c2c(cc3c4c2CCCN4CCC3)C(c2ccccc2C)=c2cc3c4c(c21)CCC[N+]=4CCC3	O	764
CCN1CCCc2cc3c(cc21)P(=O)(O)c1cc2c(cc1C31OC(=O)c3ccccc31)CCCN2CC	O	725	CCN(CC)c1ccc2c(-c3ccccc3C(=O)OC3CC(C)(C)N([O-])CC3(C)C)c3ccc(=[N+](CC)CC)cc-3oc2c1	CC#N	588	CN(C)c1ccc2c(c1)S(=O)(=O)C1=CC(=[N+](C)C)C=CC1=C2c1ccccc1	O	736

CCN1CCc2cc3c(cc21)P(=O)(O)c1cc2c(cc1C31OC(=O)c3ccccc31)CCN2CC	O	755	CCc1c(C)[nH]c(C2=C3C=CC(=[N+](C)C)C=C3[Si](C)(C)c3cc(N(C)C)ccc32)c1C	O	680	Cc1ccccc1C1=C2C=CC(=[N+](C)C)C=C2S(=O)(=O)c2cc(N(C)C)ccc21	O	742
Cc1cccc(-c2c3ccc(=N)cc-3oc3cc(N)ccc23)c1C(C)O	O	520	CC[N+](CC)=C1C=CC2=C(c3ccccc3C(=O)O)C3C=CC(N=Nc4cc(C(C)C)c(O)cc4C)=CC3OC2=C1	O	550	COc1ccccc1C1=C2C=CC(=[N+](C)C)C=C2S(=O)(=O)c2cc(N(C)C)ccc21	O	747
CC1(C)C2=CC(=N)C=CC2=C(c2ccccc2CO)c2ccc(N)cc21	O	582	CCN(CC)c1ccc2c(-c3ccccc3C(=O)N3CCN(C(=O)c4ccc(O)cc4)CC3)c3ccc(=[N+](CC)CC)cc-3oc2c1	O	590	Cc1cccc(C)c1C1=C2C=CC(=[N+](C)C)C=C2S(=O)(=O)c2cc(N(C)C)ccc21	O	742
C[Si]1(C)C2=CC(=N)C=CC2=C(c2cc(C(=O)O)ccc2CO)c2ccc(N)cc21	O	615	CC[N+](CC)=c1ccc2c(-c3ccccc3C(=O)O)c3ccc(-n4[nH]c(C)cc4=O)cc3oc-2c1	O	579	COc1cccc(OC)c1C1=C2C=CC(=[N+](C)C)C=C2S(=O)(=O)c2cc(N(C)C)ccc21	O	752
C[Si]1(C)C2=CC(=N)C=CC2=C(c2ccccc2CO)c2ccc(N)cc21	O	615	CN(C(=O)OCc1ccc(C=O)c(O)c1)c1ccc2c(c1)Oc1cc(N(C)C(=O)OCc3ccc(C=O)c(O)c3)ccc1C21OC(=O)c2ccccc21	O	543	CN(C)c1ccc2cc3ccc(=[N+](C)C)cc-3oc2c1	O	562
C[N+](C)=C1C=CC2=C(c3ccccc3CO)c3ccc(N)cc3[Si](C)(C)C2=C1	O	643	CCN(CC)c1ccc2c(c1)Oc1cc(OCc3ccc(B4OC(C)(C)C(C)(C)O4)cc3)c(COC(=O)c3ccc(/N=N/c4ccc(N(C)C)cc4)cc3)cc1C21c2ccccc2C(=O)N1CCC[P+](	O	528	CN(C)c1ccc2c(c1)[Si](C)(C)C1=CC(=[N+](C)C)C=CC1=C2	O	653

			c1ccccc1)(c1ccccc1)c1ccccc1					
C[Si]1(C)c2cc(N)ccc2C(c2ccccc2CO)=c2cc3c4c(c21)CCC[N+]=4CCC3	O	662	CCN(CC)c1ccc2c(-c3ccccc3C(=O)O)c3c([o+]c2c1)C(=Cc1ccc(NC(=O)C(=O)c2ccc([N+](=O)[O-])cc2)cc1)CCC3	O	730	CN(C)c1ccc2c(c1)[Ge](C)(C)C1=CC(=[N+](C)C)C=CC1=C2	O	634
C[Si]1(C)c2cc(NC(=O)CCC(N)C(=O)O)ccc2C(c2ccccc2CO)=c2cc3c4c(c21)CCC[N+]=4CCC3	O	671	CN(C)c1ccc2c(c1)B(O)C1=CC(=[N+](C)C)C=CC1=C2c1ccccc1CS(=O)(=O)O	O	575	CN(C)c1ccc2cc3ccc(N(C)C)cc3nc2c1	O	528
O=C(O)c1ccccc1-c1c2ccc(N3CCC3)cc2nc2cc(N3CCC3)ccc12	O	533	CCN(CC)c1ccc2c(c1)Oc1cc(OC(C)=O)ccc1C21OC(=O)c2ccccc21	O	560	c1cc2cc3ccc(N4CCC4)cc3nc2cc1N1CCC1	O	531
O=C([O-])c1ccccc1-c1c2ccc(=[N+]3CCC3)cc-2oc2cc(N3CCC3)ccc12	O	571	CCN(CC)c1ccc2c(c1)Oc1cc(N(CC)CC)ccc1C21c2ccccc2C(=O)N1CCNCCN1C(=O)c2cccc3c(/C=C/c4cc[n+](Cc5ccc(B6OC(C)(C)C(C)(C)O6)cc5)cc4)ccc(c23)C1=O	O	470	CN(C)c1ccc2c(c1)C(C)(C)C1=CC(=[N+](C)C)C=CC1=C2CCCCCCCNC(=O)OC(C)(C)C	O	599
O=C([O-])c1ccccc1-c1c2ccc(=[N+]3CCC3)cc-2sc2cc(N3CCC3)ccc12	O	593	CCN(CC)c1ccc2c(-c3ccccc3C(=O)OCc3c4ccccc4cc4ccccc34)c3ccc(=[N+](CC)CC)cc-3oc2c1	O	590	CN(C)c1ccc2c(c1)[Si](C)(C)C1=CC(=[N+](C)C)C=CC1=C2CCCCCCCNC(=O)OC(C)(C)C	O	623
O=C([O-])c1ccccc1C1=C2C=CC(=[N+]3CCC3)C=C2C	O	631	CCN(CC)c1ccc2c(c1)OC1=CC(N(CC)CC)C=CC1=C2c1ccccc1C(=	O	576	CN(C)c1ccc2c(c1)S(=O)(=O)C1=CC(=[N+](C)C)C=CC1=C2CCCC	O	647

c2cc(N3CCC3)ccc21			O)N1CCN(S(=O)(=O)c2ccc3c4c5c(ccc6c5c(c3c2)-c2ccc(C)cc2C6=O)C(=O)c2cc(C)ccc2-4)CC1			CCCNC(=O)OC(C)(C)C		
C[Si]1(C)C2=CC(=[N+]3CCC3)C=CC2=C(c2ccccc2C(=O)[O-])c2ccc(N3CCC3)cc21	O	664	CC/N=c1/cc2oc3cc(N(CC)Cc4c5ccccc5cc5ccccc45)c(C)cc3c(-c3ccccc3C(=O)CC)c-2cc1C	O	560	CN(C)c1ccc2c(c1)C(C)(C)C1=CC(=[N+](C)C)C=CC1=C2	CCO	627
O=C([O-])c1ccccc1C1=C2C=CC(=[N+]3CCC3)C=C2P(=O)(O)c2cc(N3CCC3)ccc21	O	687	CC/N=c1/cc2oc3cc(N(CC)Cc4c5ccccc5cc5ccccc45)c(C)cc3c(-c3ccccc3C(=O)CC)c-2cc1C	CN(C=O)C	700	CCN(CC)c1ccc2c(c1)C(C)(C)C1=CC(=[N+](C)C)C=CC1=C2	CO	630
O=C([O-])c1ccccc1-c1c2ccc(=[N+]3CC(F)(F)C3)cc-2oc2cc(N3CC(F)(F)C3)ccc12	O	549	COc1c2ccccc2c(OC)c2cc(C3=C4C=CC(=[N+](C)C)C=C4C(C)(OC)c4cc(N(C)C)ccc43)ccc12	O	667	CCN1CCc2cc3c(cc21)C(C)(C)c1cc2c(cc1=C3)CC[N+]=2CC	CCO	657
O=C([O-])c1ccccc1C1=C2C=CC(=[N+]3CC(F)(F)C3)C=C2Cc2cc(N3CC(F)(F)C3)ccc21	O	609	CCN(CC)c1ccc2c(c1)Oc1cc(N3CCN(C(=O)CN4C(=O)c5cccc6c(OC)ccc(c56)C4=O)CC3)ccc1C21c2ccccc2C(=O)N1NC(=S)Nc1ccccc1	CS(C)=O	477	CCN1CCCc2cc3c(cc21)C(C)(C)c1cc2c(cc1=C3)CCC[N+]=2CC	CCO	648
O=C(O)c1ccccc1-c1c2ccc(N3CC(F)(F)C3)cc2nc2cc(N3CC(F)(F)C3)ccc12	O	517	CCN(CC)c1ccc2c(c1)Oc1cc(N3CCN(C(=O)CN4C(=O)c5cccc6c(OC)ccc(c56)C4=O)CC3)ccc1C21c2ccccc2C(=O)N1N	O	618	CCN1c2cc3c(cc2C(C)=CC1(C)C)C=c1cc2c(cc1C3(C)C)=[N+](CC)C(C)(C)C=C2C	CCO	688

			C(=S)Nc1ccccc1					
O=C([O-])c1ccccc1-c1c2cc(F)c(=[N+]3CCC3)cc-2oc2cc(N3CCC3)c(F)cc12	O	575	CCN(CC)c1ccc2c(c1)Oc1cc(N(CC)CC)ccc1C21c2ccccc2C(=S)N1NC(=O)c1ccc(CN2CCOCC2)cc1	O	592	CCN1CCc2cc3c(cc21)C(C)(C)c1cc2c(cc1=C3)CCC[N+]=2CC	CCO	650
O=C([O-])c1ccccc1-c1c2cc(F)c(=[N+]3CC(F)(F)C3)cc-2oc2cc(N3CC(F)(F)C3)c(F)cc12	O	550	CN(C)c1ccc2c(c1)B(O)c1cc(N(C)C)ccc1C21SCc2ccccc21	O	646	CCN1CCc2cc3c(cc21)C(C)(C)c1cc2c(cc1=C3)C(C)=CC(C)(C)[N+]=2CC	CCO	675
C[Si]1(C)C2=CC(=[N+]3CCC3)C=CC2=C(c2c(F)c(F)c(F)c(F)c2C(=O)[O-])c2ccc(N3CCC3)cc21	O	682	CCN(CC)c1ccc2c(c1)Oc1cc(N(CC)CC)ccc1C21c2ccccc2C(=O)N1NC=O	O	580	CCN1CCCc2cc3c(cc21)C(C)(C)c1cc2c(cc1=C3)C(C)=CC(C)(C)[N+]=2CC	CCO	664
O=C([O-])c1c(F)c(F)c(F)c(F)c1C1=C2C=CC(=[N+]3CCC3)C=C2P(=O)(O)c2cc(N3CCC3)ccc21	O	707	CCN(CC)C1=CC2Oc3cc(N(CC)CC)ccc3C3(c4ccccc4C(=O)N3NC(=O)C3CCC(=O)N3C(=O)C3CCC(=O)N3)C2C=C1	O	580	CCN1CCc2cc3c(cc21)C(C)(C)C1=CC(=[N+](C)C)C=CC1=C3	CCO	641
O=C([O-])c1c(F)c(F)c(F)c(F)c1C1=C2C=CC(=[N+]3CCC3)C=C2P(=O)(c2ccccc2)c2cc(N3CCC3)ccc21	O	743	CCOCCOCCOCC[n+]1ccc(C(=O)NN2C(=O)c3ccccc3C23c2ccc(N(CC)CC)cc2Oc2cc4c(cc23)Sc2ccccc2N4C)cc1	O	730	CCN1CCCc2cc3c(cc21)C(C)(C)C1=CC(=[N+](C)C)C=CC1=C3	CCO	640
O=C([O-])c1c(F)c(F)c(F)c(F)c1C1=C2C=CC(=[N+]3CCC3)C=C2S(=O)(=O)c2cc(N3CCC3)ccc21	O	748	CCN(CC)c1ccc2c(c1)Oc1cc(N(CC)CC)ccc1C21c2ccccc2C(=O)C1(Cl)C#N	O	578	CCN1c2cc3c(cc2C(C)=CC1(C)C)C=C1C=CC(=[N+](C)C)C=C1C3(C)C	CCO	658

O=C([O-])c1c(F)c(F)c(F)c(F)c1-c1c2ccc(=[N+]3CCC3)cc-2oc2cc(N3CCC3)ccc12	O	590	C=CC(=O)Oc1ccc2c(CC(=O)N3CCN(c4ccc5c(c4)Oc4cc(N(CC)CC)ccc4C54c5ccccc5C(=O)N4NC(=S)Nc4ccccc4)CC3)cc(=O)oc2c1	CC#N	616	CCN(CC)c1ccc2c(SCC(NC(=O)CCC(N)C(=O)O)C(=O)NCC(=O)O)c3ccc(=[N+](CC)CC)cc-3oc2c1	O	622
O=C([O-])c1c(F)c(F)c(F)c(F)c1-c1c2ccc(=[N+]3CCC3)cc-2sc2cc(N3CCC3)ccc12	O	612	CCN(CC)c1ccc2c(c1)Oc1cc(N(CC)CC)ccc1C21c2ccccc2C(=O)N1Nc1ccccc1	O	580	N#CC1=c2cc3c4c(c2Oc2c1cc1c5c2CCCN5CCC1)CCC[N+]=4CCC3	O	720
O=C([O-])c1c(F)c(F)c(F)c(F)c1-c1c2ccc(=[N+]3CC(F)C3)cc-2oc2cc(N3CC(F)C3)ccc12	O	579	CCN(CC)c1ccc2c(-c3ccccc3C(=O)OC)c3cc(/C=N/N(C)C)c(=O)cc-3oc2c1	O	571	C=c1ccc2c(c1)Oc1cc(C)ccc1N=2	CO	599
O=C([O-])c1c(F)c(F)c(F)c(F)c1C1=C2C=CC(=[N+]3CC(F)C3)C=C2P(=O)(c2ccccc2)c2cc(N3CC(F)C3)ccc21	O	732	CCN(CC)c1ccc2c(c1)Oc1c(cc3c4c1CCCN4CCC3)C21c2ccccc2C(=O)N1N	O	585	CC=Cc1ccc2c(c1)Oc1cc(CCC)ccc1N2	CO	615
N=c1ccc2c(-c3cccc(C(=O)O)c3C(=O)O)c3ccc(N)cc3oc-2c1	O	522	CCN(CC)c1ccc2c(c1)Oc1cc(N3CCN(c4ccc5c6c(cccc46)C(=O)N(CCN(C)C)C5=O)CC3)ccc1C21c2ccccc2C(=O)N1N	O	578	CC=C(CC)c1ccc2c(c1)Oc1cc(C(CC)CC)ccc1N2	CO	664
CN=c1ccc2c(-c3cccc(C(=O)O)c3C(=O)O)c3ccc(NC)cc3oc-2c1	O	545	CCN(CC)c1ccc2c(c1)Oc1c(ccc3[nH]c4ccccc4c13)C21c2ccccc2C(=O)N1N	O	585	CC[N+]1=c2cc3c(cc2CC1)=Nc1ccc(C)cc1O3	CCO	642
C[N+](C)=c1ccc2c(-c3cccc(C(=O)O)c3C(=O)[O-	O	547	CCN(CC)c1ccc2c(c1)Oc1cc(N(CC)CC)ccc1C21c2cccc	O	585	CC[N+]1=c2cc3c(cc2CCC1)=Nc1cc2c(cc1O3)N(CCC	O	676

])c3ccc(O)cc3oc-2c1			c2C(=O)N2CCCCN21			C(=O)O)CCC2		
C=CCN(CC=C)c1ccc2c(-c3cccc(C(=O)O)c3C(=O)[O-])c3ccc(=[N+](CC=C)CC=C)cc-3oc2c1	O	570	CNc1ccc(OC)c(C2=C3C=CC(=[N+](C)C)C=C3[Si](C)(C)c3cc(N(C)C)ccc32)c1	O	672	CC[N+]1=c2cc3c(cc2C(C)CC1(C)C)=Nc1cc2c(cc1O3)N(CCCC(=O)O)CCC2	O	682
C=CCN(C)c1ccc2c(-c3cccc(C(=O)O)c3C(=O)[O-])c3cc/c(=[N+](\C)CC=C)cc-3oc2c1	O	572	CCN(CC)c1ccc2c(c1)Oc1cc(N(CC)CC)ccc1C21c2ccccc2C(=O)N1NC(=O)CN1CCN(C)CC1	O	600	CN1CCOc2cc3c(cc21)Oc1cc2c(cc1=N3)OCC[N+]=2C	CO	670
CC1(C)C2=CC(=O)C=CC2=C(c2cccc(C(=O)O)c2C(=O)O)c2ccc(O)cc21	O	574	CCN(CC)c1ccc2c(c1)Oc1cc(N(CC)CC)ccc1C21c2ccccc2CN1c1ccccc1N	O	590	CN1CCSc2cc3c(cc21)Oc1cc2c(cc1=N3)OCC[N+]=2C	CO	695
CN(C)c1ccc2c(-c3cccc(C(=O)O)c3C(=O)[O-])c3ccc(=[N+](C)C)cc-3oc2c1	O	573	CCOC(=O)c1ccccc1-c1c2ccc(Nc3ccccc3N)cc2[o+]c2cc(N(CC)CC)ccc12	O	581	CN1CCCc2cc3c(cc21)Oc1cc2c(cc1=N3)OCC[N+]=2C	CO	677
O=C(O)c1cccc(C2=c3cc4c5c(c3Oc3c2cc2c6c3CCCN6CCC2)CCC[N+]=5CCC4)c1C(=O)[O-]	O	599	CCN(CC)c1ccc2c(c1)Oc1cc(N(CC)CC)ccc1C21c2ccccc2C(=O)N1CCN=Cc1ccccc1OCc1ccccn1	O	587	N=c1cc2oc3cc(N)ccc3nc-2c2ccccc12	CCO	619
CN=C1C=CC2=C(c3cccc(C(=O)O)c3C(=O)O)c3ccc(NC)cc3CC2=C1	O	609	C#Cc1ccc(N2C(=O)c3ccccc3C23c2ccc(N(CC)CC)cc2Oc2cc(N(CC)CC)ccc23)c(N)c1	O	580	CCN(CC)c1ccc2nc3c4ccccc4c(=N)cc-3oc2c1	O	675
CN(C)c1ccc2c(c1)C(C)(C)C1=CC(=[N+](C)C)C=CC1=C2c1cccc(C(=O)O)c1C(=O)[O-]	O	636	C/C=C/C(=O)Oc1ccc(COC(=O)Nc2ccc3c(c2)Oc2cc(N)ccc2C32OC(=O)c3ccccc32)cc1	O	525	N=c1cc2oc3cc(N4CCCC4)ccc3nc-2c2ccccc12	O	678

C=CCN(C)c1ccc2c(c1)C(C)(C)C1=C/C(=[N+](/C)CC=C)C=CC1=C2c1cccc(C(=O)O)c1C(=O)[O-]	O	636	CCN(CC)c1ccc2c(c1)Oc1cc(N(CC)CC)ccc1C21c2ccccc2C(=O)N1CC=O	O	584	N=c1cc2oc3cc(N(CCCS(=O)(=O)O)CCCS(=O)(=O)O)ccc3nc-2c2ccccc12	O	675
CN(C)c1ccc2c(c1)C(C)(C)c1cc3c(cc1=C2c1cccc(C(=O)O)c1C(=O)[O-])CC[N+]=3C	O	648	C=CC(=O)NN1C(=O)c2ccccc2C12c1ccc(N(CC)CC)cc1Oc1cc(N(CC)CC)ccc12	O	582	CCN(CC)c1ccc2c(c1)OC1=C(/C=C/C3=[N+](C)c4ccccc4C3(C)C)CCCC1=C2	CO	743
CN(C)c1ccc2c(c1)C(C)(C)c1c3c4c(cc1=C2c1cccc(C(=O)O)c1C(=O)[O-])CCC[N+]=4CCC3	O	644	CCN(CC)c1ccc2c(c1)Oc1cc(N(CC)CC)ccc1C21c2cc(C(=O)O)ccc2C(=O)N1/N=C/C=O	O	576	CCNc1cc2c(cc1C)C(c1ccccc1C(=O)O)=C1CCCC(/C=C/C3=[N+](C)c4ccccc4C3(C)C)=C1O2	CCO	721
CN1CCc2cc3c(cc21)C(C)(C)c1cc2c(cc1=C3c1cccc(C(=O)O)c1C(=O)[O-])CC[N+]=2C	O	661	CCN(CC)c1ccc2c(c1)Oc1cc(N(CC)CC)ccc1C21c2ccccc2C(=O)N1NC(=O)CBr	O	579	CCNc1cc2c(cc1C)C(c1ccccc1C(=O)O)=C1CCCC(/C=C/C3=[N+](C)c4ccc5ccccc5c4C3(C)C)=C1O2	CCO	741
CN(C)c1ccc2c(c1)C(C)(C)c1cc3c(cc1=C2c1cccc(C(=O)O)c1C(=O)[O-])C=CC(C)(C)[N+]=3C	O	678	CC[N+](CC)=c1ccc2c(-c3ccccc3C(=O)N3CCN(c4ccc([N+](=O)[O-])c5nonc45)CC3)c3ccc(N=[N+]=[N-])cc3oc-2c1	O	565	CCN(CC)c1cc2c(cc1C)C(c1ccccc1C(=O)O)=C1CCCC(/C=C/C3=[N+](C)c4ccccc4C3(C)C)=C1O2	CCO	731
CC1(C)c2c(cc3c4c2CCCN4CCC3)C(c2cccc(C(=O)O)c2C(=O)[O-])=c2cc3c4c(c21)CCC[N+]=4CCC3	O	658	CCN(CC)c1ccc2c(c1)Oc1cc(N3CCN(C(=O)CN4C(=O)c5cccc6c(N=[N+]=[N-])ccc(c56)C4=O)CC3)ccc1C21c2ccccc2C(=O)N1N	CN(C=O)C	560	CCN(CC)c1cc2c(cc1C)C(c1ccccc1C(=O)O)=C1CCCC(/C=C/C3=[N+](C)c4ccc5ccccc5c4C3(C)C)=C1O2	CCO	750
CN(C)c1ccc2c(c1)[Si](C)(C)C1=CC(=[N+](C)	O	669	C=CC(=O)Oc1ccc2c(CC(=O)N3CCN(c	CC#N	488	C[N+]1=C(/C=C/C2=C3Oc4c(cc5c6c4CC	CCO	745

C)C=CC1=C2c1cccc(C(=O)O)c1C(=O)[O-]			4ccc5c(c4)Oc4cc(N(CC)CC)ccc4C54C5C=CC=CC5C(=O)N4NC(=S)NC4=CCCC=C4)CC3)cc(=O)oc2c1			CN6CCC5)C(c4ccccc4C(=O)O)=C3CCC2)C(C)(C)c2ccccc21		
CN(C)c1ccc2c(c1)[Si](C)(C)c1c3c4c(cc1=C2c1cccc(C(=O)O)c1C(=O)[O-])CCC[N+]=4CCC3	O	683	COc1ccc(Oc2c([N+](=O)[O-])cc(C#Cc3ccc(-c4c5ccc(=[N+](C)C)cc-5oc5cc(N(C)C)ccc45)c(C(=O)[O-])c3)c3nsnc23)cc1	O	590	C[N+]1=C(/C=C/C2=C3Oc4c(cc5c6c4CCCN6CCC5)C(c4ccccc4C(=O)O)=C3CCC2)C(C)(C)c2c1ccc1ccccc21	CCO	763
CN(C)c1ccc2c(c1)[Si](C)(C)c1cc3c(cc1=C2c1cccc(C(=O)O)c1C(=O)[O-])CC[N+]=3C	O	693	CCN(CC)c1ccc2c(-c3ccccc3C(=O)OCOC(C)=O)c3c([o+]c2c1)-c1ccc(N=[N+]=[N-])cc1CC3	O	630	CCN(CC)c1ccc2c(c1)OC1=C(/C=C/C3=[N+](CC)c4ccccc4C3(C)C)CCCC1=C2/C=C1/N(CC)c2ccccc2C1(C)C	ClCCl	740
CN1CCc2cc3c(cc21)[Si](C)(C)c1c2c4c(cc1=C3c1cccc(C(=O)O)c1C(=O)[O-])CCC[N+]=4CCC2	O	708	CCN(CC)c1ccc2c(-c3ccccc3C=O)c3ccc(=[N+](CC)CC)cc-3oc2c1	O	592	CCNc1cc2c(cc1C)C(/C=C1/N(CC)c3ccccc3C1(C)C)=C1CCCC(/C=C/C3=[N+](CC)c4ccccc4C3(C)C)=C1O2	ClCCl	732
CC1=CC(C)(C)[N+](C)=c2cc3c(cc21)=C(c1cccc(C(=O)O)c1C(=O)[O-])c1ccc(N(C)C)cc1[Si]3(C)C	O	719	CCN(CC)c1ccc2c(c1)Oc1cc(NC(=O)OCc3ccc(N=[N+]=[N-])cc3)ccc1C21c2ccccc2C(=O)N1CCN1CCOCC1	O	560	CCNc1ccc2c(c1)OC1=C(/C=C/C3=[N+](CC)c4ccccc4C3(C)C)CCCC1=C2/C=C1/N(CC)c2ccccc2C1(C)C	ClCCl	735
CN1CCc2cc3c(cc21)[Si](C)(C)c1cc2c(cc1=C3c1cccc(C(=O)O)	O	721	C#CC(C)OC(=O)c1ccccc1C1=C2C=CC(N(CC)CC)=CC2OC2=CC(	CN(C=O)C	590	CCN1/C(=C/C2=C3CCCC(/C=C/C4=[N+](CC)c5ccccc5C4(C)C)=C	ClCCl	744

c1C(=O)[O-])CC[N+]=2C			=[N+](CC)CC)C=CC21			3Oc3c2cc2c4c3CCCN4CCC2)C(C)(C)c2ccccc21		
CC1=CC(C)(C)N(C)c2cc3c(cc21)C(c1cccc(C(=O)O)c1C(=O)[O-])=c1cc2c(cc1[Si]3(C)C)=[N+](C)C(C)(C)C=C2C	O	757	CCN(CC)C1=C(OC2=CCC([N+](=O)[O-])C=C2[N+](=O)[O-])C(/C=N/N2C(=O)C3=CC=CCC3C23C2=C(CC(N(CC)CC)C=C2)OC2=CC(=[N+](CC)CC)C=CC23)=CCC1	O	770	CCN(CC)c1ccc2c(-c3ccccc3C(=O)O)c3c([o+]c2c1)C(=Cc1ccc(O)cc1)CCC3	CO	654
CC[N+]1=c2cc3c(cc2Sc2ccccc21)=C(c1c(F)c(F)c(F)c(F)c1C(=O)[O-])c1cc2c4c(c1O3)C(C)(C)CCN4CCC2(C)C	CC(C)=O	756	CCN(CC)c1ccc2c(c1)P(C)(=O)C1=CC(=[N+](CC)CC)C=CC1=C2c1ccccc1	ClCCl	702	CCN(CC)c1ccc2c(-c3ccccc3C(=O)O)c3c([o+]c2c1)C(=Cc1ccc(O)c(Cl)c1)CCC3	CO	657
CC[N+]1=c2cc3c(cc2Sc2ccccc21)=C(c1c(F)c(F)c(F)c(F)c1C(=O)[O-])c1cc2c4c(c1O3)C(C)(C)CCN4CCC2(C)C	C1=CC=CC=C1	731	CCN(CC)c1ccc2c(c1)P(C)(=O)C1=CC(=[N+](CC)CC)C=CC1=C2c1ccccc1	CC#N	700	CCN(CC)c1ccc(/C=C2\CCCc3c2[o+]c2cc(N(CC)CC)ccc2c3-c2ccccc2C(=O)O)cc1	CO	747
CC[N+]1=c2cc3c(cc2Sc2ccccc21)=C(c1c(F)c(F)c(F)c(F)c1C(=O)[O-])c1cc2c4c(c1O3)C(C)(C)CCN4CCC2(C)C	ClC(Cl)Cl	736	CCN(CC)c1ccc2c(c1)P(C)(=O)C1=CC(=[N+](CC)CC)C=CC1=C2c1ccccc1	CCO	709	CCN(CC)c1ccc(/C=C2\CCCc3c2[o+]c2cc(N(CC)CC)ccc2c3-c2ccccc2C(=O)O)c(O)c1	CO	753
CC[N+]1=c2cc3c(cc2Sc2ccccc21)=C(c1c(F)c(F)c(F)c(F)c1C(=O)[O-])c1cc2c4c(c1O3)C(C)(C)CCN4CCC2(C)C	ClCCl	740	CCN(CC)c1ccc2c(c1)P(C)(=O)C1=CC(=[N+](CC)CC)C=CC1=C2c1ccccc1	O	712	CCN(CC)c1ccc2c(c1)OC(=C1C=CC(=[N+](CC)CC)C=C1)C=C2c1ccccc1C(=O)O	O	648

CC[N+]1=c2cc3c(cc2Sc2ccccc21)=C(c1c(F)c(F)c(F)c(F)c1C(=O)[O-])c1cc2c4c(c1O3)C(C)(C)CCN4CCC2(C)C	CN(C=O)C	750	CCN(CC)c1ccc2c(c1)P(C)(=O)C1=CC(=[N+](CC)CC)C=CC1=C2c1ccccc1C	ClCCl	702	CC[N+](CC)=C1C=CC(=C2C=C(c3ccccc3C(=O)O)c3cc4c5c(c3O2)CCCN5CCC4)C=C1	O	648
CC[N+]1=c2cc3c(cc2Sc2ccccc21)=C(c1c(F)c(F)c(F)c(F)c1C(=O)[O-])c1cc2c4c(c1O3)C(C)(C)CCN4CCC2(C)C	CS(C)=O	776	CCN(CC)c1ccc2c(c1)P(C)(=O)C1=CC(=[N+](CC)CC)C=CC1=C2c1ccccc1C	CC#N	700	CCN(CC)c1ccc2c(c1)O/C(=C/C=C1C=CC(=[N+](CC)CC)C=C1)C=C2c1ccccc1C(=O)O	O	727
CC[N+]1=c2cc3c(cc2Sc2ccccc21)=C(c1c(F)c(F)c(F)c(F)c1C(=O)[O-])c1cc2c4c(c1O3)C(C)(C)CCN4CCC2(C)C	CC(OCC)=O	739	CCN(CC)c1ccc2c(c1)P(C)(=O)C1=CC(=[N+](CC)CC)C=CC1=C2c1ccccc1C	CCO	709	CC[N+](CC)=C1C=CC(=C/C=C2\C=C(c3ccccc3C(=O)O)c3cc4c5c(c3O2)CCCN5CCC4)C=C1	O	738
CC[N+]1=c2cc3c(cc2Sc2ccccc21)=C(c1c(F)c(F)c(F)c(F)c1C(=O)[O-])c1cc2c4c(c1O3)C(C)(C)CCN4CCC2(C)C	CCO	750	CCN(CC)c1ccc2c(c1)P(C)(=O)C1=CC(=[N+](CC)CC)C=CC1=C2c1ccccc1C	O	712	CCN(CC)c1ccc2cc(/C=C/C=C3\CCCc4c3[o+]c3cc(N(CC)CC)ccc3c4-c3ccccc3C(=O)O)c(=O)oc2c1	CO	807
CC[N+]1=c2cc3c(cc2Sc2ccccc21)=C(c1c(F)c(F)c(F)c(F)c1C(=O)[O-])c1cc2c4c(c1O3)C(C)(C)CCN4CCC2(C)C	O	758	CCN(CC)c1ccc2c(c1)P(C)(=O)C1=CC(=[N+](CC)CC)C=CC1=C2c1c(C)cc(C)cc1C	ClCCl	703	CCN(CC)c1ccc2cc(/C=C/C=C/C=C3\CCCc4c3[o+]c3cc(N(CC)CC)ccc3c4-c3ccccc3C(=O)O)c(=O)oc2c1	CO	830
CC[N+]1=c2cc3c(cc2Sc2ccccc21)=C(c1c(F)c(F)c(F)c(F)c1C(=O)[O-])c1cc2c4c(c1O3)C(C)(C)CCN4CCC2(C)C	CC#N	747	CCN(CC)c1ccc2c(c1)P(C)(=O)C1=CC(=[N+](CC)CC)C=CC1=C2c1c(C)cc(C)cc1C	CC#N	702	CCN(CC)c1ccc2c(c1)oc(=O)c1c(-c3ccccc3C(=O)O)c3ccc(=[N+](CC)CC)cc-3oc12	CCO	637

CC[N+]1=c2cc3c(cc2Sc2ccccc21)=C(c1c(F)c(F)c(F)c(F)c1C(=O)[O-])c1cc2c4c(c1O3)C(C)(C)CCN4CCC2(C)C	CO	754	CCN(CC)c1ccc2c(c1)P(C)(=O)C1=CC(=[N+](CC)CC)C=CC1=C2c1c(C)cc(C)cc1C	CCO	710	CC[N+](CC)=c1ccc2c(-c3ccccc3C(=O)O)c3c(=O)oc4ccccc4c3oc-2c1	CCO	651
CC[N+]1=c2cc3c(cc2Sc2ccccc21)=C(c1c(F)c(F)c(F)c(F)c1C(=O)[O-])c1cc2c4c(c1O3)C(C)(C)CCN4CCC2(C)C	C1CCCO1	737	CCN(CC)c1ccc2c(c1)P(C)(=O)C1=CC(=[N+](CC)CC)C=CC1=C2c1c(C)cc(C)cc1C	O	713	CC[N+](CC)=c1ccc2c(-c3ccccc3C(=O)O)c3c(=O)oc4c5c6c(cc4c3oc-2c1)CCCN6CCC5	CCO	674
CC[N+]1=c2cc3c(cc2Sc2ccccc21)=C(c1c(F)c(F)c(F)c(F)c1C(=O)[O-])c1cc2c4c(c1O3)C(C)(C)CCN4CCC2(C)C	C1COCCO1	729	N=c1ccc2c(-c3ccccc3C(=O)O)c3ccc(N)cc3oc-2c1	O	520	COC(=O)c1ccccc1-c1c2ccc(=N)cc-2oc2c1ccc1ccc(O)c12	O	604
CCN(CC)c1ccc2c(-c3ccccc3CNC3CC3)c3ccc(=[N+](CC)CC)cc-3oc2c1	O	585	CN=c1ccc2c(-c3ccccc3C(=O)O)c3ccc(NC)cc3oc-2c1	O	553	COC(=O)c1ccccc1-c1c2ccc(=N)cc-2oc2c1ccc1ccc(OC)c12	O	596
CCN(CC)c1ccc2c(-c3ccccc3CNC3CCC3)c3ccc(=[N+](CC)CC)cc-3oc2c1	O	585	CN(C)c1ccc2c(-c3ccccc3C(=O)[O-])c3ccc(=[N+](C)C)cc-3oc2c1	O	572	COC(=O)c1ccccc1-c1c2ccc(=[N+](C)C)cc-2oc2c1ccc1ccc(O)c12	O	614
CCN(CC)c1ccc2c(-c3ccccc3CNC3CCCC3)c3ccc(=[N+](CC)CC)cc-3oc2c1	O	585	CCN=c1cc2oc3cc(NCC)c(C)cc3c(-c3ccccc3C(=O)O)c-2cc1C	O	548	COC(=O)c1ccccc1-c1c2ccc(=[N+](C)C)cc-2oc2c1ccc1ccc(OC)c12	O	606
N=c1ccc2c(-c3ccccc3C(=O)O)c3ccc4cc(N)ccc4c3oc-2c1	O	645	CC[N+](CC)=c1ccc2c(-c3ccccc3C(=O)O)c3c(ccc4ccc(O)cc43)oc-2c1	O	605	O=C([O-])c1ccccc1C1=c2cc3c4c(c2Oc2c1ccc1ccc(O)c21)CCC[N+]=4CCC3	O	624
C[N+](C)=c1ccc2c(-	O	612	COC(=O)c1ccccc1-	O	760	COc1cccc2ccc3c(c12)Oc1c	O	622

c3ccccc3C(=O)[O-])c3ccc4cc(N)ccc4c3oc-2c1			c1c2ccc(=N)cc-2oc2c1ccc1cccc(N)c12			2c4c(cc1=C3c1ccccc1C(=O)[O-])CCC[N+]=4CCC2		
CN(C)c1ccc2c(ccc3c(-c4ccccc4C(=O)O)c4ccc(=N)cc-4oc32)c1	O	652	COC(=O)c1ccccc1-c1c2ccc(=[N+](C)C)cc-2oc2c1ccc1cccc(N)c12	O	770	CNc1ccc2c3c(ccc2c1)C(c1ccccc1C(=O)[O-])=c1cc2c4c(c1O3)CCC[N+]=4CCC2	O	624
Nc1ccc2c3c(ccc2c1)C(c1ccccc1C(=O)[O-])=c1cc2c4c(c1O3)CCC[N+]=4CCC2	O	612	COC(=O)c1ccccc1C1=c2cc3c4c(c2Oc2c1ccc1cccc(N)c21)CCC[N+]=4CCC3	O	740			

Table S9. The overlapped subset of rhodamine derivatives.

SMILES /Rhodamine	Solvent/SMILES	$\lambda_{abs}/\lambda_{emi}$	SMILES /Rhodamine	Solvent/SMILES	$\lambda_{abs}/\lambda_{emi}$	SMILES /Rhodamine	Solvent/SMILES	$\lambda_{abs}/\lambda_{emi}$
N=c1ccc2c(-c3ccccc3CO)c3ccc(N)cc3oc-2c1	O	501/524	CC1=CC(C)(C)N(C)c2cc3c(cc21)C(c1ccc(C(=O)O)cc1C(=O)[O-])=c1cc2c(cc1[Si]3(C)C)=[N+](C)C(C)(C)C=C2C	O	721/757	CC1(C)C2=CC(=N)C=CC2=C(c2ccccc2C(=O)O)c2ccc(N)cc21	O	552/577
CC(C)CC(N)C(=O)Nc1ccc2c(c1)Oc1cc(N)ccc1C21OCc2ccccc21	O	496/525	CC[N+]1=c2cc3c(cc2Sc2ccccc21)=C(c1c(F)c(F)c(F)c(F)c1C(=O)[O-])c1cc2c4c(c1O3)C(C)(C)CCN4CCC2(C)C	CC(C)=O	633/756	CN(C)c1ccc2c(c1)C(C)(C)C1=CC(=[N+](C)C)C=CC1=C2c1ccccc1C(=O)O	CCO	612/642
CC(=O)Nc1ccc2c(c1)Oc1cc(N)ccc1C21OCc2ccccc21	O	495/526	CC[N+]1=c2cc3c(cc2Sc2ccccc21)=C(c1c(F)c(F)c(F)c(F)c1C(=O)[O-])c1cc2c4c(c1O3)C(C)(C)C	C1=CC=CC=C1	638/731	CC1(C)C2=CC(=[N+]3CCCC3)C=CC2=C(c2ccccc2C(=O)O)c2ccc(N3CCCC3)cc21	O	613/633

			CN4CCC2(C)C					
C[N+](C)=c1ccc2c(-c3ccccc3CO)c3ccc(N)cc3oc-2c1	O	528/552	CC[N+]1=c2cc3c(cc2Sc2ccccc21)=C(c1c(F)c(F)c(F)c(F)c1C(=O)[O-])c1cc2c4c(c1O3)C(C)(C)CCN4CCC2(C)C	ClC(Cl)Cl	627/736	CN(C)c1ccc2c(c1)[Ge](C)(C)C1=CC(=[N+](C)C)C=CC1=C2	O	621/634
CC(=O)Nc1ccc2c(c1)Oc1cc(N(C)C)ccc1C21OCc2ccccc21	O	499/571	CC[N+]1=c2cc3c(cc2Sc2ccccc21)=C(c1c(F)c(F)c(F)c(F)c1C(=O)[O-])c1cc2c4c(c1O3)C(C)(C)CCN4CCC2(C)C	ClCCl	621/740	CN(C)c1ccc2cc3ccc(N(C)C)cc3nc2c1	O	493/528
CC[N+](CC)=c1ccc2c(-c3ccccc3CO)c3ccc(N)cc3oc-2c1	O	532/555	CC[N+]1=c2cc3c(cc2Sc2ccccc21)=C(c1c(F)c(F)c(F)c(F)c1C(=O)[O-])c1cc2c4c(c1O3)C(C)(C)CCN4CCC2(C)C	CN(C=O)C	626/750	c1cc2cc3ccc(N4CCC4)cc3nc2cc1N1CCC1	O	492/531
CCN(C)c1ccc2c(c1)Oc1cc(NC(C)=O)ccc1C21OCc2ccccc21	O	501/573	CC[N+]1=c2cc3c(cc2Sc2ccccc21)=C(c1c(F)c(F)c(F)c(F)c1C(=O)[O-])c1cc2c4c(c1O3)C(C)(C)CCN4CCC2(C)C	CS(C)=O	634/776	Cc1ccccc1C1=C2C=CC(=[N+](C)C)C=C2P(=O)([O-])c2cc(N(C)C)ccc21	O	666/685
CC(=O)NCC(=O)N1CCCC1C(=O)Nc1ccc2c(c1)Oc1cc(N)ccc1C21OCc2ccccc21	O	496/527	CC[N+]1=c2cc3c(cc2Sc2ccccc21)=C(c1c(F)c(F)c(F)c(F)c1C(=O)[O-])c1cc2c4c(c1O3)C(C)(C)CCN4CCC2(C)C	CC(OCC)=O	639/739	CN(C)c1ccc2c(-c3ccccc3)c3ccc(=[N+](C)C)cc-3sc2c1	CO	571/599
Nc1ccc2c(c1)Oc1cc(NC(=O)O[C@@H]3C[C@H](CO)[C@H](	O	494/525	CC[N+]1=c2cc3c(cc2Sc2ccccc21)=C(c1c(F)c(F)c(F)c(F	CCO	622/750	CN(C)c1ccc2c(-c3ccccc3)c3cc	CO	581/608

O)[C@H](O)[C@H]3O)ccc1C21OCc2ccccc21			)c1C(=O)[O-])c1cc2c4c(c1O3)C(C)(C)CCN4CCC2(C)C			c(=[N+](C)C)cc-3[se]c2c1		
CC[N+](CC)=c1ccc2c(-c3ccccc3CO)c3ccc([O-])cc3oc-2c1	O	525/543	CC[N+]1=c2cc3c(cc2Sc2ccccc21)=C(c1c(F)c(F)c(F)c(F)c1C(=O)[O-])c1cc2c4c(c1O3)C(C)(C)CCN4CCC2(C)C	O	613/758	CCN=c1cc2oc3cc(NCC)c(C)cc3c(-c3ccccc3C(=O)O)c-2cc1C	O	521/548
CN(C)c1ccc2c(-c3ccccc3CO)c3ccc(=[N+](C)C)cc-3oc2c1	O	552/574	CC[N+]1=c2cc3c(cc2Sc2ccccc21)=C(c1c(F)c(F)c(F)c(F)c1C(=O)[O-])c1cc2c4c(c1O3)C(C)(C)CCN4CCC2(C)C	CC#N	616/747	CCN=c1ccc2c(-c3ccccc3C(=O)O)c3ccc(NCC)cc3oc-2c1	O	554/577
CN(C)c1ccc2c(-c3c(CO)cccc3COc3ccc(N)cc3)c3ccc(=[N+](C)C)cc-3oc2c1	O	553/574	CC[N+]1=c2cc3c(cc2Sc2ccccc21)=C(c1c(F)c(F)c(F)c(F)c1C(=O)[O-])c1cc2c4c(c1O3)C(C)(C)CCN4CCC2(C)C	CO	623/754	CCN=c1cc2oc3cc(NCC)ccc3c(-c3ccccc3C(=O)O)c-2cc1C	O	539/566
CN(C)c1ccc2c(-c3c(CO)cccc3COc3ccc(O)cc3)c3ccc(=[N+](C)C)cc-3oc2c1	O	553/574	CC[N+]1=c2cc3c(cc2Sc2ccccc21)=C(c1c(F)c(F)c(F)c(F)c1C(=O)[O-])c1cc2c4c(c1O3)C(C)(C)CCN4CCC2(C)C	C1CCCO1	631/737	O=C([O-])c1ccccc1C1=c2cc3c4c(c2Oc2c1cc1c5c2CCCN5CCC1)CCC[N+]=4CCC3	O	580/600
C[N+](C)=c1ccc2c(-c3ccccc3CO)c3cc(F)c(N)cc3oc-2c1	O	532/559	CC[N+]1=c2cc3c(cc2Sc2ccccc21)=C(c1c(F)c(F)c(F)c(F)c1C(=O)[O-])c1cc2c4c(c1O3)C(C)(C)CCN4CCC2(C)C	C1COCCO1	637/729	CCN(CC)c1ccc2c(c1)Oc1c3c4c(cc1=C2c1ccccc1C(=O)[O-])CCC[N+]=4CCC3	O	566/587

C[N+](C)=c1ccc2c(-c3ccccc3CO)c3cc(Cl)c(N)cc3oc-2c1	O	536/559	CCN(CC)c1ccc2c(-c3ccccc3CNC3CC3)c3ccc(=[N+](CC)CC)cc-3oc2c1	O	565/585	CCNc1cc2c(cc1C)C(c1ccccc1C(=O)[O-])=c1cc3c4c(c1O2)CCC[N+]=4CCC3	O	554/580
CC(=O)Nc1cc2c(cc1F)C1(OCc3ccccc31)c1ccc(N(C)C)cc1O2	O	507/582	CCN(CC)c1ccc2c(-c3ccccc3CNC3CCC3)c3ccc(=[N+](CC)CC)cc-3oc2c1	O	558/585	CC/N=c1/cc2oc3cc4c(cc3c(-c3ccccc3C(=O)O)c-2cc1C)N(CC)C1CCCCC1N4CC	CCO	570/646
CC(=O)Nc1cc2c(cc1Cl)C1(OCc3ccccc31)c1ccc(N(C)C)cc1O2	O	505/581	CCN(CC)c1ccc2c(-c3ccccc3CNC3CCCC3)c3ccc(=[N+](CC)CC)cc-3oc2c1	O	568/585	N=c1ccc2c(-c3ccc(C(=O)O)cc3C(=O)O)c3ccc(N)c(S(=O)(=O)O)c3oc-2c1S(=O)(=O)O	O	495/519
CC[N+](CC)=c1ccc2c(-c3ccccc3CO)c3cc(F)c(N)cc3oc-2c1	O	538/566	CCN(CC)c1ccc2c(-c3ccccc3CNC3CCCCC3)c3ccc(=[N+](CC)CC)cc-3oc2c1	O	566/585	CC1CC(C)(C)N=c2c1cc1c(c2S(=O)(=O)O)Oc2c(ccc(N)c2S(=O)(=O)O)C=1c1ccc(C(=O)O)cc1C(=O)O	O	518/540
CC[N+](CC)=c1ccc2c(-c3ccccc3CO)c3cc(Cl)c(N)cc3oc-2c1	O	541/568	CCN=c1cc2oc3cc(NCC)c(C)cc3c(-c3ccccc3CNC3CC3)c-2cc1C	O	532/552	CC1N=c2c(cc3c(c2S(=O)(=O)O)Oc2c(cc4c(c2S(=O)(=O)O)NC(C)C4(C)C)C=3c2ccc(C(=O)O)cc2C(=O)O)C1(C)C	O	531/554
CCN(CC)c1ccc2c(c1)Oc1cc(NC(C)=O)c(Cl)cc1C21OCc2ccccc21	O	507/578	CCN=c1cc2oc3cc(NCC)c(C)cc3c(-c3ccccc3CNC3CCC3)c-2cc1C	O	529/554	CC1(C)C=C(CS(=O)(=O)O)c2cc3c(c(S(=O)(=O)O)c2=N1)Oc1c(cc2c(c1S(=O)(=O)O)NC(C)(C)C=C2CS(=O)(=O)O)C=3c1ccc(C(=O)O)cc1C(=O)O	O	578/603

CCN(CC)c1ccc2c(c1)Oc1cc(NC(C)=O)c(F)cc1C21OCc2ccccc21	O	508/583	CCN=c1cc2oc3cc(NCC)c(C)cc3c(-c3ccccc3CNC3CCCC3)c-2cc1C	O	532/554	CN1c2c(cc3c(c2S(=O)(=O)O)Oc2c(S(=O)(=O)[O-])c4c(cc2=C3c2ccc(C(=O)O)cc2C(=O)O)C(CS(=O)(=O)O)=CC(C)(C)[N+]=4C)C(CS(=O)(=O)O)=CC1(C)C	O	590/617
Nc1ccc2c(c1)Oc1c3c4c(cc1=C2c1ccccc1CO)CCC[N+]=4CCC3	O	547/569	CCN=c1cc2oc3cc(NCC)c(C)cc3c(-c3ccccc3CNC3CCCCC3)c-2cc1C	O	533/554	Nc1ccc2c(c1)Cc1c-2[o+]c2ccccc2c1-c1ccccc1C(=O)O	O	478/548
Nc1cc2c(cc1F)C(c1ccccc1CO)=c1cc3c4c(c1O2)CCC[N+]=4CCC3	O	553/582	CCN(CC)c1ccc2c(-c3ccccc3C(=O)NCCNc3ncc(C(=O)OC)c(Nc4ccccc4)n3)c3ccc(=[N+](CC)CC)cc-3oc2c1	ClCCl	303/460	COc1ccc2c(-c3ccccc3C(=O)O)c3c([o+]c2c1)-c1ccc(N)cc1C3	O	490/550
Nc1cc2c(cc1Cl)C(c1ccccc1CO)=c1cc3c4c(c1O2)CCC[N+]=4CCC3	O	555/582	CCN(CC)c1ccc2c(-c3ccccc3C(=O)NCCNc3ncc(C(=O)OC)c(Nc4ccccc4)n3)c3ccc(=[N+](CC)CC)cc-3oc2c1	CC(OCC)=O	310/456	Nc1ccc2c(c1)Cc1c-2[o+]c2cc(O)ccc2c1-c1ccccc1C(=O)O	O	522/582
OCc1ccccc1-c1c2ccc(=NCC(F)(F)F)cc-2oc2cc(NCC(F)(F)F)ccc12	O	502/526	CCN(CC)c1ccc2c(-c3ccccc3C(=O)NCCNc3ncc(C(=O)OC)c(Nc4ccccc4)n3)c3ccc(=[N+](CC)CC)cc-3oc2c1	C1CCCO1	309/451	CN(C)c1ccc2c(-c3ccccc3C(=O)O)c3c([o+]c2c1)-c1ccc(N)cc1C3	O	557/608
O=C(O)c1ccc(-c2c3ccc(=[N+](CC(F)(F)F)CC(F)(F)F)cc-3oc3cc(N(CC(F)(F)F)CC(F)(F)	O	507/532	CCN(CC)c1ccc2c(-c3ccccc3C(=O)NCCNc3ncc(C(=O)OC)c(Nc4ccccc4)	CS(C)=O	311/491	Nc1ccc2c(c1)Cc1c-2[o+]c2cc(Cl)ccc2c1-c1ccccc1C(=O)O	O	484/554

F)ccc23)c(CO)c1			n3)c3ccc(=[N+](CC)CC)cc-3oc2c1					
O=C(O)c1ccc(-c2c3ccc(=[N+](CC(F)(F)F)CC(F)(F)F)cc-3oc3cc(N(CC(F)(F)F)CC(F)(F)F)ccc23)c(CCO)c1	O	507/530	CCN(CC)c1ccc2c(-c3ccccc3C(=O)NCCNc3ncc(C(=O)OC)c(NCc4ccccc4)n3)c3ccc(=[N+](CC)CC)cc-3oc2c1	ClCCl	305/456	Nc1ccc2c(c1)Cc1c-2[o+]c2cc3c4c(c2c1-c1ccccc1C(=O)O)CCCN4CCC3	O	582/632
CC(=O)Nc1ccc2c(c1)Oc1c3c4c(cc1=C2c1ccccc1CO)CCC[N+]=4CCC3	O	513/589	CCN(CC)c1ccc2c(-c3ccccc3C(=O)NCCNc3ncc(C(=O)OC)c(NCc4ccccc4)n3)c3ccc(=[N+](CC)CC)cc-3oc2c1	CC(OCC)=O	305/454	CCN(CC)c1ccc2c(-c3ccccc3C(=O)O)c3c([o+]c2c1)-c1ccc(N)cc1C3	O	564/619
CC(=O)Nc1cc2c(cc1F)C(c1ccccc1CO)=c1cc3c4c(c1O2)CCC[N+]=4CCC3	O	519/599	CCN(CC)c1ccc2c(-c3ccccc3C(=O)NCCNc3ncc(C(=O)OC)c(NCc4ccccc4)n3)c3ccc(=[N+](CC)CC)cc-3oc2c1	CS(C)=O	310/489	CCN(CC)c1ccc2c(-c3ccccc3C(=O)O)c3c([o+]c2c1)-c1ccc(Cl)cc1C3	CCO	540/608
CC(=O)Nc1cc2c(cc1Cl)C(c1ccccc1CO)=c1cc3c4c(c1O2)CCC[N+]=4CCC3	O	517/592	CCN(CC)c1ccc2c(-c3ccccc3C(=O)NCCNc3ncc(C(=O)OC)c(NCCc4ccccc4)n3)c3ccc(=[N+](CC)CC)cc-3oc2c1	ClCCl	306/456	CCN(CC)c1ccc2c(-c3ccccc3C(=O)O)c3c([o+]c2c1)-c1ccccc1C3	CCO	524/602
CN(C)c1ccc2c(c1)[Si](C)(C)C1=CC(=[N+](C)C)C=CC1=C2c1ccc(C(=O)O)cc1CO	O	650/671	CCN(CC)c1ccc2c(-c3ccccc3C(=O)NCCNc3ncc(C(=O)OC)c(NCCc4ccccc4)n3)c3ccc(=[N+](CC)CC)cc-3oc2c1	CC(OCC)=O	309/453	CCN(CC)c1ccc2c(-c3ccccc3C(=O)O)c3c([o+]c2c1)-c1ccc(OC)cc1C3	CCO	550/596
O=C(O)c1ccccc1C1=c2cc3c(cc2Oc2cc4c(cc21)	O	538/588	CCN(CC)c1ccc2c(-c3ccccc3C(=	C1CCCO1	307/448	CCN(CC)c1ccc2c(-c3ccccc3C(=O	CCO	567/604

CCCN4)=NCCC3			O)NCCNc3ncc(C(=O)OC)c(NCCc4ccccc4)n3)c3ccc(=[N+](CC)CC)cc-3oc2c1			)O)c3c([o+]c2c1)-c1ccc(O)cc1C3		
C[Si]1(C)c2cc3c(cc2C(c2ccccc2C(=O)O)=c2cc4c(cc21)=NCCC4)CCCN3	O	637/654	CCN(CC)c1ccc2c(-c3ccccc3C(=O)NCCNc3ncc(C(=O)OC)c(NCCc4ccccc4)n3)c3ccc(=[N+](CC)CC)cc-3oc2c1	CS(C)=O	310/489	CCN(CC)c1ccc2c(-c3ccccc3C(=O)O)c3c([o+]c2c1)-c1ccc(NC)cc1C3	CCO	573/613
N=C1C=CC2=C(c3ccccc3C(=O)O)c3ccc(N)cc3P(=O)(O)C2=C1	CO	635/655	CCN(CC)c1ccc2c(-c3ccccc3C(=O)NCCNc3ncc(C(=O)OC)c(NCCc4ccc(OC)c(OC)c4)n3)c3ccc(=[N+](CC)CC)cc-3oc2c1	ClCCl	307/456	CCN(CC)c1ccc2c(c1)Cc1c-2[o+]c2cc(N(CC)CC)ccc2c1-c1ccccc1C(=O)O	CCO	583/639
CCN(CC)c1ccc2c(c1)P(=O)([O-])C1=CC(=[N+](CC)CC)C=CC1=C2c1ccccc1C(=O)O	CO	670/690	CCN(CC)c1ccc2c(-c3ccccc3C(=O)NCCNc3ncc(C(=O)OC)c(NCCc4ccc(OC)c(OC)c4)n3)c3ccc(=[N+](CC)CC)cc-3oc2c1	CC(OCC)=O	308/453	CN(C)c1ccc2c(c1)Cc1c-2[o+]c2cc3c4c(c2c1-c1ccccc1C(=O)O)CCCN4CCC3	O	600/640
CCN1c2cc3c(cc2C(C)=CC1(C)C)C(c1ccccc1C(=O)O)=c1cc2c(cc1P3(=O)[O-])=[N+](CC)C(C)(C)C=C2C	CO	715/732	CCN(CC)c1ccc2c(-c3ccccc3C(=O)NCCNc3ncc(C(=O)OC)c(NCCc4ccc(OC)c(OC)c4)n3)c3ccc(=[N+](CC)CC)cc-3oc2c1	C1CCCO1	308/449	CCN(CC)c1ccc2c(c1)Cc1c-2[o+]c2cc3c4c(c2c1-c1ccccc1C(=O)O)CCCN4CCC3	O	604/642
CN=C1C=CC2=C(c3ccccc3C(=O)O)c3ccc(NC)cc3P(=O)(O)C2=C1	CO	641/661	CCN(CC)c1ccc2c(-c3ccccc3C(=O)NCCNc3ncc(C(=O)OC)c(NCCc4ccc(	CS(C)=O	309/489	CCN(CC)c1ccc2c(-c3ccccc3C(=O)O)c3c([o+]c2c1)-	O	532/576

			OC)c(OC)c4)n3)c3ccc(=[N+](CC)CC)cc-3oc2c1			c1cccc(O)c1C3		
CN=C1C=CC2=C(c3ccccc3S(=O)(=O)O)c3ccc(NC)cc3P(=O)(O)C2=C1	CO	649/691	CCN(CC)c1ccc2c(-c3ccccc3C(=O)N3CCN(C)CC3)c3ccc(=[N+](CC)CC)cc-3oc2c1	O	565/588	CCN(CC)c1ccc2c(-c3ccccc3C(=O)O)c3c([o+]c2c1)-c1ccc(O)cc1C3	O	567/604
CN(C)c1ccc2c(c1)Oc1cc(N(C)C)ccc1C21c2ccccc2S(=O)(=O)N1CCCC(=O)NCCOCCOCCCCCCCl	O	561/580	CCN(CC)c1ccc2c(-c3ccccc3C(=O)N3CCN(C)CC3)c3ccc(=[N+](CC)CC)cc-3oc2c1	CO	560/585	CCN(CC)c1ccc2c(-c3ccccc3C(=O)O)c3c([o+]c2c1)-c1cc(O)ccc1C3	O	545/573
CN(C)c1ccc2c(c1)Oc1cc(N(C)C)ccc1C21c2ccccc2S(=O)(=O)N1CCCC(=O)NCCOCCOCCCCCCCl	CCO	559/580	CCN(CC)c1ccc2c(-c3ccccc3C(=O)N3CCN(C)CC3)c3ccc(=[N+](CC)CC)cc-3oc2c1	CCO	563/586	CCN(CC)c1ccc2c(-c3ccccc3C(=O)O)c3c([o+]c2c1)-c1c(O)cccc1C3	O	575/597
CN(C)c1ccc2c(c1)Oc1cc(N(C)C)ccc1C21c2ccccc2S(=O)(=O)N1CCCC(=O)NCCOCCOCCCCCCCl	OCC(O)CO	565/586	CCN(CC)c1ccc2c(-c3ccccc3C(=O)N3CCN(C)CC3)c3ccc(=[N+](CC)CC)cc-3oc2c1	CCCO	561/585	CCN(CC)c1ccc2c(-c3ccccc3C(=O)O)c3c([o+]c2c1)-c1ccc(N)cc1C3	O	574/627
CCN(CC)c1ccc2c(c1)Oc1cc(N(CC)CC)ccc1C21c2ccccc2S(=O)(=O)N1CCCC(=O)NCCOCCOCCCCCCCl	O	566/582	CCN(CC)c1ccc2c(-c3ccccc3C(=O)N3CCN(C)CC3)c3ccc(=[N+](CC)CC)cc-3oc2c1	CC(O)C	561/585	O=S(=O)([O-])c1ccccc1-[c+]1c2ccc(N3C4CCC3CC4)cc2oc2cc(N3[C@H]4CC[C@@H]3CC4)ccc21	CO	548/567
CCN(CC)c1ccc2c(c1)Oc1cc(N(CC)CC)ccc1C21c2ccccc2S(=O)(=O)N1CCCC(=O)NCCOCCOCCCCCCCl	CCO	560/582	CCN(CC)c1ccc2c(-c3ccccc3C(=O)N3CCN(C)CC3)c3ccc(=[N+](CC)CC)cc-3oc2c1	CC#N	560/584	CCN(CC)c1ccc2c(c1)Oc1cc3c(cc1=C2c1ccccc1C(=O)O)Sc1ccccc1[N+]=3CC	CCO	606/730
CCN(CC)c1ccc2c(c1)Oc1cc(N(CC)CC)ccc1C21c2ccccc2S(=O)	OCC(O)CO	572/587	CCN(CC)c1ccc2c(-c3ccccc3C(=O)N3CCN(C	CS(C)=O	568/594	CN(C)c1cc2oc3cc(=[N+](C)C)c(-c4ccccc4)cc-	CO	548/571

(=O)N1CCCC(=O)NCCOCCOCCCCCCCl			)CC3)c3ccc(=[N+](CC)CC)cc-3oc2c1			3c(-c3ccccc3C(=O)[O-])c2cc1-c1ccccc1		
O=C(CCCN1C2(c3ccc(N4CCCC4)cc3Oc3cc(N4CCCC4)ccc32)c2ccccc2S1(=O)=O)NCCOCCOCCCCCCCl	O	567/588	CCN(CC)c1ccc2c(-c3ccccc3C(=O)N3CCN(C)CC3)c3ccc(=[N+](CC)CC)cc-3oc2c1	CN(C=O)C	564/589	CN1c2cc3c(cc2-c2ccccc2C1(C)C)C(c1ccccc1C(=O)[O-])=c1cc2c(cc1O3)=[N+](C)C(C)(C)c1ccccc1-2	CO	582/604
O=C(CCCN1C2(c3ccc(N4CCCC4)cc3Oc3cc(N4CCCC4)ccc32)c2ccccc2S1(=O)=O)NCCOCCOCCCCCCCl	CCO	563/586	CCN(CC)c1ccc2c(-c3ccccc3C(=O)N3CCN(C)CC3)c3ccc(=[N+](CC)CC)cc-3oc2c1	ClCCl	565/585	CN1c2cc3c(cc2-c2ccsc2C1(C)C)C(c1ccccc1C(=O)[O-])=c1cc2c(cc1O3)=[N+](C)C(C)(C)c1sccc1-2	CO	581/605
O=C(CCCN1C2(c3ccc(N4CCCCC4)cc3Oc3cc(N4CCCCC4)ccc32)c2ccccc2S1(=O)=O)NCCOCCOCCCCCCCl	O	573/596	CCN(CC)c1ccc2c(-c3ccccc3C(=O)N3CCN(C)CC3)c3ccc(=[N+](CC)CC)cc-3oc2c1	ClC(Cl)Cl	561/581	CN1c2cc3c(cc2-c2sccc2C1(C)C)C(c1ccc(C(=O)O)cc1C(=O)[O-])=c1cc2c(cc1O3)=[N+](C)C(C)(C)c1ccsc1-2	CO	596/620
O=C(CCCN1C2(c3ccc(N4CCCCC4)cc3Oc3cc(N4CCCCC4)ccc32)c2ccccc2S1(=O)=O)NCCOCCOCCCCCCCl	CCO	569/592	CCN(CC)c1ccc2c(-c3ccccc3C(=O)N3CCN(C)CC3)c3ccc(=[N+](CC)CC)cc-3oc2c1	C1COCCO1	562/587	CN1c2cc3c(cc2-c2sc4ccccc4c2C1(C)C)C(c1ccc(C(=O)O)cc1C(=O)[O-])=c1cc2c(cc1O3)=[N+](C)C(C)(C)c1c-2sc2ccccc12	CO	600/627
C[Si]1(C)c2cc(N3CCC3)ccc2C2(OCc3cc(C(=O)O)ccc32)C2C=CC(N3CCC3)=CC21	N#CC	655/670	CCN(CC)CCN(C)C(=O)c1ccccc1-c1c2ccc(=[N+](CC)CC)cc-2oc2cc(N(CC)CC)ccc12	O	566/590	CN1c2cc3c(cc2-c2sccc2C1(C)C)C(c1c(Cl)c(Cl)c(Cl)c(Cl)c1C(=O)[O-])=c1cc2c(cc1O3)=[N+](C)	CO	631/648

						C(C)(C)c1ccsc1-2		
C[Si]1(C)c2cc(N3CCC3)ccc2C2(OCc3cc(C(=O)O)ccc32)C2C=CC(N3CCC3)=CC21	ClC(Cl)Cl	567/668	CCN(CC)CCN(C)C(=O)c1ccccc1-c1c2ccc(=[N+](CC)CC)cc-2oc2cc(N(CC)CC)ccc12	CO	561/586	CN1c2cc3c(cc2-c2sccc2C1(C)C)C(c1c(F)c(F)c(F)c(F)c1C(=O)[O-])=c1cc2c(cc1O3)=[N+](C)C(C)(C)c1ccsc1-2	CO	628/649
C[Si]1(C)c2cc(N3CCC3)ccc2C2(OCc3cc(C(=O)O)ccc32)C2C=CC(N3CCC3)=CC21	CS(C)=O	667/683	CCN(CC)CCN(C)C(=O)c1ccccc1-c1c2ccc(=[N+](CC)CC)cc-2oc2cc(N(CC)CC)ccc12	CCO	564/589	CN1c2cc3c(cc2-c2ccc4ccccc4c2C1(C)C)C(c1c(Cl)c(Cl)c(Cl)c(Cl)c1C(=O)[O-])=c1cc2c(cc1O3)=[N+](C)C(C)(C)c1c-2ccc2ccccc12	CO	627/645
C[Si]1(C)c2cc(N3CCC3)ccc2C2(OCc3cc(C(=O)O)ccc32)C2C=CC(N3CCC3)=CC21	CC(OCC)=O	658/675	CCN(CC)CCN(C)C(=O)c1ccccc1-c1c2ccc(=[N+](CC)CC)cc-2oc2cc(N(CC)CC)ccc12	CCCO	562/586	CN1c2cc3c(cc2-c2sccc2C1(C)C)C(c1ccc(S(=O)(=O)O)cc1S(=O)(=O)[O-])=c1cc2c(cc1O3)=[N+](C)C(C)(C)c1ccsc1-2	CO	610/629
C[Si]1(C)c2cc(N3CCC3)ccc2C2(OCc3cc(C(=O)O)ccc32)C2C=CC(N3CCC3)=CC21	CCO	657/689	CCN(CC)CCN(C)C(=O)c1ccccc1-c1c2ccc(=[N+](CC)CC)cc-2oc2cc(N(CC)CC)ccc12	CC(O)C	563/585	CN1c2cc3c(cc2-c2sc4ccccc4c2C1(C)C)C(c1ccc(S(=O)(=O)O)cc1S(=O)(=O)[O-])=c1cc2c(cc1O3)=[N+](C)C(C)(C)c1c-2sc2ccccc12	CO	616/633
C[Si]1(C)c2cc(N3CCC3)ccc2C2(OCc3cc(C(=O)O)ccc32)C2C=CC(N3CCC3)=CC21	O	655/669	CCN(CC)CCN(C)C(=O)c1ccccc1-c1c2ccc(=[N+](CC)CC)cc-	CC#N	560/584	CN1c2cc3c(cc2-c2c(sc4ccccc24)C1(C)c1ccccc1)C(c1ccc(S(=O)(=O)O)cc	CO	634/672

			2oc2cc(N(CC)CC)ccc12			1S(=O)(=O)[O-])=c1cc2c(cc1O3)=[N+](C)C(C)(c1ccccc1)c1sc3ccccc3c1-2		
C[N+]1(C)CCN(C2=CC3Oc4cc(N5CC[N+](C)(C)CC5)ccc4C4(OCc5ccccc54)C3C=C2)CC1	N#CC	533/558	CCN(CC)CCN(C)C(=O)c1ccccc1-c1c2ccc(=[N+](CC)CC)cc-2oc2cc(N(CC)CC)ccc12	CS(C)=O	569/593	CCN1CCCc2cc3c(cc21)C(C)(C)c1cc2c(cc1=C3c1ccccc1C(=O)O)CCC[N+]=2CC	CCO	632/662
C[N+]1(C)CCN(C2=CC3Oc4cc(N5CC[N+](C)(C)CC5)ccc4C4(OCc5ccccc54)C3C=C2)CC1	CC(OCC)=O	541/559	CCN(CC)CCN(C)C(=O)c1ccccc1-c1c2ccc(=[N+](CC)CC)cc-2oc2cc(N(CC)CC)ccc12	CN(C=O)C	566/590	CCN1CCCc2cc3c(cc21)C(C)(C)c1cc2c(cc1=C3c1ccccc1C(=O)O)C(C)=CC(C)(C)[N+]=2CC	CCO	653/683
C[N+]1(C)CCN(C2=CC3Oc4cc(N5CC[N+](C)(C)CC5)ccc4C4(OCc5ccccc54)C3C=C2)CC1	CCO	537/558	CCN(CC)CCN(C)C(=O)c1ccccc1-c1c2ccc(=[N+](CC)CC)cc-2oc2cc(N(CC)CC)ccc12	ClCCl	561/583	CCN1c2cc3c(cc2C(C)=CC1(C)C)C(c1ccccc1C(=O)O)=c1cc2c(cc1C3(C)C)=[N+](CC)C(C)(C)C=C2	CCO	670/700
C[N+]1(C)CCN(C2=CC3Oc4cc(N5CC[N+](C)(C)CC5)ccc4C4(OCc5ccccc54)C3C=C2)CC1	O	531/555	CCN(CC)CCN(C)C(=O)c1ccccc1-c1c2ccc(=[N+](CC)CC)cc-2oc2cc(N(CC)CC)ccc12	ClC(Cl)Cl	562/585	CCN1CCCc2cc3c(cc21)C(C)(C)c1cc2c(cc1=C3c1c(Cl)c(Cl)c(Cl)c(Cl)c1C(=O)O)CCC[N+]=2CC	CCO	662/692
CCN(CC)c1ccc2c(c1)[Si](C)(C)c1cc(N(CC)CC)ccc1C21OC(=O)c2ccccc21	O	650/666	CCN(CC)CCN(C)C(=O)c1ccccc1-c1c2ccc(=[N+](CC)CC)cc-2oc2cc(N(CC)CC)ccc12	C1COCCO1	563/582	CCN1CCCc2cc3c(cc21)C(C)(C)c1cc2c(cc1=C3c1c(Cl)c(Cl)c(Cl)c(Cl)c1C(=O)O)C(C)=CC(C)(C)[N+]=2CC	CCO	683/713
O=c1ccc2c(-c3ccccc3CO)c3ccc(NCC(F)(F)F)cc3oc-2c1	O	479/515	CCN(CC)c1ccc2c(-c3ccccc3C(=O)N3C[C@@H](C)O[C@@H](C)C3)c3ccc(=[N+](CC)	O	566/591	CCN1c2cc3c(cc2C(C)=CC1(C)C)C(c1c(Cl)c(Cl)c(Cl)c(Cl)c1C(=O)O)=c1cc2c(cc1C3(C)C)=[N+	CCO	700/730

			CC)cc-3oc2c1			](CC)C(C)(C)C=C2C		
Oc1ccc2c(c1)Oc1cc(NCC(F)(F)F)ccc1C21OCc2ccccc21	O	498/518	CCN(CC)c1ccc2c(-c3ccccc3C(=O)N3C[C@@H](C)O[C@@H](C)C3)c3ccc(=[N+](CC)CC)cc-3oc2c1	CO	560/586	CN(C)c1ccc2c(c1)C(C)(C)C1=CC(=[N+](C)C)C=CC1=C2c1c(Cl)c(Cl)c(Cl)c(Cl)c1C(=O)O	CCO	642/672
CC(=O)N[C@H]1[C@@H](Oc2ccc3c(c2)Oc2cc(NCC(F)(F)F)ccc2C3c2ccccc2CO)O[C@H](CO)[C@@H](O)[C@@H]1O	O	482/519	CCN(CC)c1ccc2c(-c3ccccc3C(=O)N3C[C@@H](C)O[C@@H](C)C3)c3ccc(=[N+](CC)CC)cc-3oc2c1	CCO	563/590	Cc1ccccc1C1=C2C=CC(=N)C=C2[Si](C)(C)c2cc(N)ccc21	O	593/613
CCN(CC)c1ccc2c(c1)Oc1c(ccc(NC(=O)CCC(N)C(=O)O)c1CF)C21OCc2ccccc21	O	499/576	CCN(CC)c1ccc2c(-c3ccccc3C(=O)N3C[C@@H](C)O[C@@H](C)C3)c3ccc(=[N+](CC)CC)cc-3oc2c1	CCCO	562/587	C[Si]1(C)c2cc3c(cc2C(c2ccccc2C(=O)O)=c2cc4c(cc21)=NCC=C4)CCCN3	O	637/654
CCN(CC)c1ccc2c(-c3ccccc3CO)c3ccc(=N)c(CO)c-3oc2c1	O	534/558	CCN(CC)c1ccc2c(-c3ccccc3C(=O)N3C[C@@H](C)O[C@@H](C)C3)c3ccc(=[N+](CC)CC)cc-3oc2c1	CC(O)C	562/588	CCN(CC)c1ccc2c(c1)[Si](C)(C)C1=CC(=[N+](CC)CC)C=CC1=C2c1ccccc1C(=O)O	O	650/666
CN(C)c1ccc2c(c1)[Si](C)(C)c1cc(O)ccc1C21OC(=O)c2ccccc21	O	609/630	CCN(CC)c1ccc2c(-c3ccccc3C(=O)N3C[C@@H](C)O[C@@H](C)C3)c3ccc(=[N+](CC)CC)cc-3oc2c1	CC#N	560/585	CN(C)c1ccc2cc3ccc(=[N+](C)C)cc-3oc2c1	O	547/562
CN(C)c1ccc2c(c1)[Si](C)(C)c1c(ccc(O)c1CO)	O	610/630	CCN(CC)c1ccc2c(-c3ccccc3C(=O)N3C[C@@	CS(C)=O	568/595	CN(C)c1ccc2c(c1)C(C)(C)C1=CC(=[N+](C)C)C=CC1	O	459/599

C21OC(=O)c2ccccc21			H](C)O[C@@H](C)C3)c3ccc(=[N+](CC)CC)cc-3oc2c1			=C2CCCCCCCNC(=O)OC(C)(C)C		
CCN(CC)c1ccc2c(c1)Oc1c(ccc(O[C@@H]3O[C@H](CO)[C@H](O)[C@H](O)[C@H]3O)c1CF)C2c1ccccc1CO	O	493/560	CCN(CC)c1ccc2c(-c3ccccc3C(=O)N3C[C@@H](C)O[C@@H](C)C3)c3ccc(=[N+](CC)CC)cc-3oc2c1	CN(C=O)C	565/590	CN(C)c1ccc2c(c1)[Si](C)(C)C1=CC(=[N+](C)C)C=CC1=C2CCCCCCCNC(=O)OC(C)(C)C	O	458/623
CCN(CC)c1ccc2c(c1)Oc1c(ccc(O[C@@H]3O[C@H](CO)[C@H](O)[C@H](O)[C@H]3O)c1C(F)F)C2c1ccccc1CO	O	493/560	CCN(CC)c1ccc2c(-c3ccccc3C(=O)N3C[C@@H](C)O[C@@H](C)C3)c3ccc(=[N+](CC)CC)cc-3oc2c1	ClCCl	562/583	CN(c1cc(S(=O)(C2=C/C(C=CC2=C3CCCCCCCNC(OC(C)(C)C)=O)=[N+](C)\C)=O)c3cc1)C	O	509/647
CCN(CC)c1ccc2c(c1)Oc1cc(O[C@@H]3O[C@H](CO)[C@H](O)[C@H](O)[C@H]3O)ccc1C2c1ccccc1CO	O	493/536	CCN(CC)c1ccc2c(-c3ccccc3C(=O)N3C[C@@H](C)O[C@@H](C)C3)c3ccc(=[N+](CC)CC)cc-3oc2c1	ClC(Cl)Cl	563/585	CN(C)c1ccc2c(c1)C(C)(C)C1=CC(=[N+](C)C)C=CC1=C2	CCO	606/627
CC[N+](CC)=c1ccc2c(-c3ccccc3CO)c3ccc([O-])c(CO)c3oc-2c1	O	526/553	CCN(CC)c1ccc2c(-c3ccccc3C(=O)N3C[C@@H](C)O[C@@H](C)C3)c3ccc(=[N+](CC)CC)cc-3oc2c1	C1COCCO1	563/580	CCN(CC)c1ccc2c(c1)C(C)(C)C1=CC(=[N+](C)C)C=CC1=C2	CO	608/630
CCN(CC)c1ccc2c(-c3ccccc3CO)c3ccc(=O)c(C=O)c-3oc2c1	O	524/556	CCN(CC)c1ccc2c(-c3ccccc3C(=O)N3CCN(CCO)CC3)c3ccc(=[N+](CC)CC)cc-3oc2c1	O	566/589	CCN1CCc2cc3c(cc21)C(C)(C)c1cc2c(cc1=C3)CC[N+]=2CC	CCO	633/657
CCN(CC)c1ccc2c(-	O	525/543	CCN(CC)c1ccc2c(-	CO	561/589	CCN1CCCc2cc3c(cc21)C(	CCO	626/648

c3ccccc3CO)c3ccc(=O)cc-3oc2c1			c3ccccc3C(=O)N3CCN(CO)CC3)c3ccc(=[N+](CC)CC)cc-3oc2c1			C)(C)c1cc2c(cc1=C3)CCC[N+]=2CC		
N=c1ccc2c(-c3ccccc3CO)c3ccc(N)cc3[se]c-2c1	O	534/562	CCN(CC)c1ccc2c(-c3ccccc3C(=O)N3CCN(CO)CC3)c3ccc(=[N+](CC)CC)cc-3oc2c1	CCO	563/588	CCN1c2cc3c(cc2C(C)=CC1(C)C)C=c1cc2c(cc1C3(C)C)=[N+](CC)C(C)(C)C=C2C	CCO	664/688
CC[N+](CC)=c1ccc2c(-c3ccccc3CO)c3ccc(NC(=O)CCC(N)C(=O)O)cc3[se]c-2c1	O	588/602	CCN(CC)c1ccc2c(-c3ccccc3C(=O)N3CCN(CO)CC3)c3ccc(=[N+](CC)CC)cc-3oc2c1	CCCO	562/586	CCN1CCc2cc3c(cc21)C(C)(C)c1cc2c(cc1=C3)CCC[N+]=2CC	CCO	629/650
CC[N+](CC)=c1ccc2c(-c3ccccc3CO)c3ccc(N)cc3[se]c-2c1	O	567/602	CCN(CC)c1ccc2c(-c3ccccc3C(=O)N3CCN(CO)CC3)c3ccc(=[N+](CC)CC)cc-3oc2c1	CC(O)C	561/585	CCN1CCc2cc3c(cc21)C(C)(C)c1cc2c(cc1=C3)C(C)=CC(C)(C)[N+]=2CC	CCO	647/675
CCN(CC)c1ccc2c(c1)[Si](C)(C)c1cc(N(CC)CC)ccc1C21OB(O)c2ccccc21	O	651/667	CCN(CC)c1ccc2c(-c3ccccc3C(=O)N3CCN(CO)CC3)c3ccc(=[N+](CC)CC)cc-3oc2c1	CC#N	560/584	CCN1CCCc2cc3c(cc21)C(C)(C)c1cc2c(cc1=C3)C(C)=CC(C)(C)[N+]=2CC	CCO	647/664
CCN(CC)c1ccc2c(-c3ccccc3B(O)O)c3ccc(=[N+](CC)CC)cc-3oc2c1	O	555/576	CCN(CC)c1ccc2c(-c3ccccc3C(=O)N3CCN(CO)CC3)c3ccc(=[N+](CC)CC)cc-3oc2c1	CS(C)=O	569/595	CCN1CCc2cc3c(cc21)C(C)(C)C1=CC(=[N+](C)C)C=CC1=C3	CCO	617/641
CN(C)c1ccc2c(c1)P(=O)(O)c1cc(N(C)C)ccc1C21OC(=O)c2ccccc21	O	667/690	CCN(CC)c1ccc2c(-c3ccccc3C(=O)N3CCN(CO)CC3)c3c	CN(C=O)C	566/590	CCN1CCCc2cc3c(cc21)C(C)(C)C1=CC(=[N+](C)C)C=CC1=C3	CCO	616/640

			cc(=[N+](CC)CC)cc-3oc2c1					
CCN(CC)c1ccc2c(c1)P(=O)(O)c1cc(N(CC)CC)ccc1C21OC(=O)c2ccccc21	O	672/695	CCN(CC)c1ccc2c(-c3ccccc3C(=O)N3CCN(CCO)CC3)c3ccc(=[N+](CC)CC)cc-3oc2c1	ClCCl	562/582	CCN1c2cc3c(cc2C(C)=CC1(C)C)C=C1C=CC(=[N+](C)C)C=C1C3(C)C	CCO	634/658
CCN1CCCc2cc3c(cc21)P(=O)(O)c1cc2c(cc1C31OC(=O)c3ccccc31)CCCN2CC	O	700/725	CCN(CC)c1ccc2c(-c3ccccc3C(=O)N3CCN(CCO)CC3)c3ccc(=[N+](CC)CC)cc-3oc2c1	ClC(Cl)Cl	563/585	CCN(CC)c1ccc2c(SCC(NC(=O)CCC(N)C(=O)O)C(=O)NCC(=O)O)c3ccc(=[N+](CC)CC)cc-3oc2c1	O	593/622
CCN1CCc2cc3c(cc21)P(=O)(O)c1cc2c(cc1C31OC(=O)c3ccccc31)CCN2CC	O	725/755	CCN(CC)c1ccc2c(-c3ccccc3C(=O)N3CCN(CCO)CC3)c3ccc(=[N+](CC)CC)cc-3oc2c1	C1COCCO1	564/587	N#CC1=c2cc3c4c(c2Oc2c1cc1c5c2CCCN5CCC1)CCC[N+]=4CCC3	O	690/720
Cc1cccc(-c2c3ccc(=N)cc-3oc3cc(N)ccc23)c1C(C)O	O	498/520	CCN(CC)c1ccc2c(-c3ccccc3C(=O)N3CCN(c4ccccc4O)CC3)c3ccc(=[N+](CC)CC)cc-3oc2c1	O	566/589	C=c1ccc2c(c1)Oc1cc(C)ccc1N=2	CO	583/599
CC1(C)C2=CC(=N)C=CC2=C(c2ccccc2CO)c2ccc(N)cc21	O	559/582	CCN(CC)c1ccc2c(-c3ccccc3C(=O)N3CCN(c4ccccc4O)CC3)c3ccc(=[N+](CC)CC)cc-3oc2c1	CO	561/582	CC=Cc1ccc2c(c1)Oc1cc(CC C)ccc1N2	CO	589/615
C[Si]1(C)C2=CC(=N)C=CC2=C(c2cc(C(=O)O)ccc2CO)c2ccc(N)cc21	O	595/615	CCN(CC)c1ccc2c(-c3ccccc3C(=O)N3CCN(c4ccccc4O)CC3)c3ccc(=[N+](CC)CC)cc-3oc2c1	CCO	563/585	CC=C(CC)c1ccc2c(c1)Oc1cc(C(CC)CC)ccc1N2	CO	643/664

C[Si]1(C)C2=CC(=N)C=CC2=C(c2ccccc2CO)c2ccc(N)cc21	O	595/615	CCN(CC)c1ccc2c(-c3ccccc3C(=O)N3CCN(c4ccccc4O)CC3)c3ccc(=[N+](CC)CC)cc-3oc2c1	CCCO	562/581	CC[N+]1=c2cc3c(cc2CC1)=Nc1ccc(C)cc1O3	CCO	612/642
C[N+](C)=C1C=CC2=C(c3ccccc3CO)c3ccc(N)cc3[Si](C)(C)C2=C1	O	623/643	CCN(CC)c1ccc2c(-c3ccccc3C(=O)N3CCN(c4ccccc4O)CC3)c3ccc(=[N+](CC)CC)cc-3oc2c1	CC(O)C	562/581	CC[N+]1=c2cc3c(cc2CCC1)=Nc1cc2c(cc1O3)N(CCCC(=O)O)CCC2	O	662/676
C[Si]1(C)c2cc(N)ccc2C(c2ccccc2CO)=c2cc3c4c(c21)CCC[N+]=4CCC3	O	637/662	CCN(CC)c1ccc2c(-c3ccccc3C(=O)N3CCN(c4ccccc4O)CC3)c3ccc(=[N+](CC)CC)cc-3oc2c1	CC#N	560/584	CC[N+]1=c2cc3c(cc2C(C)CC1(C)C)=Nc1cc2c(cc1O3)N(CCCC(=O)O)CCC2	O	664/682
C[Si]1(C)c2cc(NC(=O)CCC(N)C(=O)O)ccc2C(c2ccccc2CO)=c2cc3c4c(c21)CCC[N+]=4CCC3	O	500/671	CCN(CC)c1ccc2c(-c3ccccc3C(=O)N3CCN(c4ccccc4O)CC3)c3ccc(=[N+](CC)CC)cc-3oc2c1	CS(C)=O	568/592	CN1CCOc2cc3c(cc21)Oc1cc2c(cc1=N3)OCC[N+]=2C	CO	644/670
O=C(O)c1ccccc1-c1c2ccc(N3CCC3)cc2nc2cc(N3CCC3)ccc12	O	502/533	CCN(CC)c1ccc2c(-c3ccccc3C(=O)N3CCN(c4ccccc4O)CC3)c3ccc(=[N+](CC)CC)cc-3oc2c1	CN(C=O)C	566/588	CN1CCSc2cc3c(cc21)Oc1c2c(cc1=N3)OCC[N+]=2C	CO	659/695
O=C([O-])c1ccccc1-c1c2ccc(=[N+]3CCC3)cc-2oc2cc(N3CCC3)ccc12	O	549/571	CCN(CC)c1ccc2c(-c3ccccc3C(=O)N3CCN(c4ccccc4O)CC3)c3ccc(=[N+](CC)CC)cc-3oc2c1	ClCCl	563/583	CN1CCCc2cc3c(cc21)Oc1c2c(cc1=N3)OCC[N+]=2C	CO	649/677
O=C([O-])c1ccccc1-c1c2ccc(=[N+]3	O	570/593	CCN(CC)c1ccc2c(-c3ccccc3C(=	ClC(Cl)Cl	562/586	N=c1cc2oc3cc(N)ccc3nc-2c2ccccc12	CCO	600/619

CCC3)cc-2sc2cc(N3CCC3)ccc12			O)N3CCN(c4ccccc4O)CC3)c3ccc(=[N+](CC)CC)cc-3oc2c1					
O=C([O-])c1ccccc1C1=C2C=CC(=[N+]3CCC3)C=C2Cc2cc(N3CCC3)ccc21	O	608/631	CCN(CC)c1ccc2c(-c3ccccc3C(=O)N3CCN(c4ccccc4O)CC3)c3ccc(=[N+](CC)CC)cc-3oc2c1	C1COCCO1	564/581	CCN(CC)c1ccc2nc3c4ccccc4c(=N)cc-3oc2c1	O	635/675
C[Si]1(C)C2=CC(=[N+]3CCC3)C=CC2=C(c2ccccc2C(=O)[O-])c2ccc(N3CCC3)cc21	O	646/664	Cc1ccccc1C1=c2cc3c(cc2[Si](C)(C)c2cc4c(cc21)CCN4C)=[N+](C)CC3	O	691/712	N=c1cc2oc3cc(N4CCCC4)ccc3nc-2c2ccccc12	O	643/678
O=C([O-])c1ccccc1C1=C2C=CC(=[N+]3CCC3)C=C2P(=O)(O)c2cc(N3CCC3)ccc21	O	668/687	CC1=CC(C)(C)N(C)c2cc3c(cc21)C(c1ccccc1C)=c1cc2c(cc1[Si]3(C)C)=[N+](C)C(C)(C)C=C2C	O	712/740	N=c1cc2oc3cc(N(CCCS(=O)(=O)O)CCCS(=O)(=O)O)ccc3nc-2c2ccccc12	O	633/675
O=C([O-])c1ccccc1-c1c2ccc(=[N+]3CC(F)(F)C3)cc-2oc2cc(N3CC(F)(F)C3)ccc12	O	525/549	CCN(CC)c1ccc2c(c1)P(C)(=O)C1=CC(=[N+](CC)CC)C=CC1=C2c1c(C)cc(C)cc1C	ClCCl	687/703	CCN(CC)c1ccc2c(c1)OC1=C(/C=C/C3=[N+](C)c4ccccc4C3(C)C)CCCC1=C2	CO	700/743
O=C([O-])c1ccccc1C1=C2C=CC(=[N+]3CC(F)(F)C3)C=C2Cc2cc(N3CC(F)(F)C3)ccc21	O	585/609	CCN(CC)c1ccc2c(c1)P(C)(=O)C1=CC(=[N+](CC)CC)C=CC1=C2c1c(C)cc(C)cc1C	CC#N	684/702	CCNc1cc2c(cc1C)C(c1ccccc1C(=O)O)=C1CCCC(/C=C/C3=[N+](C)c4ccccc4C3(C)C)=C1O2	CCO	688/721
O=C(O)c1ccccc1-c1c2ccc(N3CC(F)(F)C3)cc2nc2cc(N3CC(F)(F)C3)ccc12	O	579/517	CCN(CC)c1ccc2c(c1)P(C)(=O)C1=CC(=[N+](CC)CC)C=CC1=C2c1c(C)cc(C)cc1C	CCO	691/710	CCNc1cc2c(cc1C)C(c1ccccc1C(=O)O)=C1CCCC(/C=C/C3=[N+](C)c4ccc5ccccc5c4C3(C)C)=C1O2	CCO	708/741
O=C([O-])c1ccccc1-c1c2cc(F)c(=[N	O	552/575	CCN(CC)c1ccc2c(c1)P(C)(=O)C1=CC(=	O	696/713	CCN(CC)c1c2c(cc1C)C(c1ccccc1C(=O)	CCO	700/731

+]3CCC3)cc-2oc2cc(N3CCC3)c(F)cc12			[N+](CC)CC)C=CC1=C2c1c(C)cc(C)cc1C			O)=C1CCCC(/C=C/C3=[N+](C)c4ccccc4C3(C)C)=C1O2		
O=C([O-])c1ccccc1-c1c2cc(F)c(=[N+]3CC(F)(F)C3)cc-2oc2cc(N3CC(F)(F)C3)c(F)cc12	O	526/550	CCOP1(=O)C2=CC(=[N+](C)C)C=CC2=C(c2ccccc2C)c2ccc(N(C)C)cc21	O	698/712	CCN(CC)c1cc2c(cc1C)C(c1ccccc1C(=O)O)=C1CCCC(/C=C/C3=[N+](C)c4ccc5ccccc5c4C3(C)C)=C1O2	CCO	720/750
C[Si]1(C)C2=CC(=[N+]3CCC3)C=CC2=C(c2c(F)c(F)c(F)c(F)c2C(=O)[O-])c2ccc(N3CCC3)cc21	O	669/682	Cc1ccccc1C1=c2cc3c4c(c2P(=O)([O-])c2c1cc1c5c2CCCN5CCC1)CCC[N+]=4CCC3	O	700/722	C[N+]1=C(/C=C/C2=C3Oc4c(cc5c6c4CCCN6CCC5)C(c4ccccc4C(=O)O)=C3CCC2)C(C)(C)c2ccccc21	CCO	710/745
O=C([O-])c1c(F)c(F)c(F)c(F)c1C1=C2C=CC(=[N+]3CCC3)C=C2P(=O)(O)c2cc(N3CCC3)ccc21	O	690/707	CCOP1(=O)c2c(cc3c4c2CCCN4CCC3)C(c2ccccc2C)=c2cc3c4c(c21)CCC[N+]=4CCC3	O	744/764	C[N+]1=C(/C=C/C2=C3Oc4c(cc5c6c4CCCN6CCC5)C(c4ccccc4C(=O)O)=C3CCC2)C(C)(C)c2c1ccc1ccccc21	CCO	728/763
O=C([O-])c1c(F)c(F)c(F)c(F)c1C1=C2C=CC(=[N+]3CCC3)C=C2P(=O)(c2ccccc2)c2cc(N3CCC3)ccc21	O	722/743	CCN(CC)c1ccc2c(c1)P(C)(=O)C1=CC(=[N+](CC)CC)C=CC1=C2c1ccccc1	ClCCl	688/702	CCN(CC)c1ccc2c(c1)OC1=C(/C=C/C3=[N+](CC)c4ccccc4C3(C)C)CCCC1=C2/C=C1/N(CC)c2ccccc2C1(C)C	ClCCl	720/740
O=C([O-])c1c(F)c(F)c(F)c(F)c1C1=C2C=CC(=[N+]3CCC3)C=C2S(=O)(=O)c2cc(N3CCC3)ccc21	O	724/748	CCN(CC)c1ccc2c(c1)P(C)(=O)C1=CC(=[N+](CC)CC)C=CC1=C2c1ccccc1	CC#N	682/700	CCNc1cc2c(cc1C)C(/C=C1/N(CC)c3ccccc3C1(C)C)=C1CCCC(/C=C/C3=[N+](CC)c4ccccc4C3(C)C)=C1O2	ClCCl	710/732
O=C([O-])c1c(F)c(F)c(F)c(F)c1-c1c2ccc(=[N+]3CCC3)cc-	O	571/590	CCN(CC)c1ccc2c(c1)P(C)(=O)C1=CC(=[N+](CC)CC)	CCO	690/709	CCNc1ccc2c(c1)OC1=C(/C=C/C3=[N+](CC)c4ccccc4C3(C)C)CCC	ClCCl	712/735

2oc2cc(N3CCC3)ccc12			C=CC1=C2c1ccccc1			C1=C2/C=C1/N(CC)c2ccccc2C1(C)C		
O=C([O-])c1c(F)c(F)c(F)c(F)c1-c1c2ccc(=[N+]3CCC3)cc-2sc2cc(N3CCC3)ccc12	O	593/612	CCN(CC)c1ccc2c(c1)P(C)(=O)C1=CC(=[N+](CC)CC)C=CC1=C2c1ccccc1	O	694/712	CCN1/C(=C/C2=C3CCCC(/C=C/C4=[N+](CC)c5ccccc5C4(C)C)=C3Oc3c2cc2c4c3CCCN4CCC2)C(C)(C)c2ccccc21	ClCCl	724/744
O=C([O-])c1c(F)c(F)c(F)c(F)c1-c1c2ccc(=[N+]3CC(F)C3)cc-2oc2cc(N3CC(F)C3)ccc12	O	559/579	CCN(CC)c1ccc2c(c1)P(C)(=O)C1=CC(=[N+](CC)CC)C=CC1=C2c1ccccc1C	ClCCl	688/702	CCN(CC)c1ccc2c(-c3ccccc3C(=O)O)c3c([o+]c2c1)C(=Cc1ccc(O)cc1)CCC3	CO	570/654
O=C([O-])c1c(F)c(F)c(F)c(F)c1C1=C2C=CC(=[N+]3CC(F)C3)C=C2P(=O)(c2ccccc2)c2cc(N3CC(F)C3)ccc21	O	711/732	CCN(CC)c1ccc2c(c1)P(C)(=O)C1=CC(=[N+](CC)CC)C=CC1=C2c1ccccc1C	CC#N	684/700	CCN(CC)c1ccc2c(-c3ccccc3C(=O)O)c3c([o+]c2c1)C(=Cc1ccc(O)c(Cl)c1)CCC3	CO	577/657
N=c1ccc2c(-c3cccc(C(=O)O)c3C(=O)O)c3ccc(N)cc3oc-2c1	O	500/522	CCN(CC)c1ccc2c(c1)P(C)(=O)C1=CC(=[N+](CC)CC)C=CC1=C2c1ccccc1C	CCO	691/709	CCN(CC)c1ccc(/C=C2\CCCc3c2[o+]c2cc(N(CC)CC)ccc2c3-c2ccccc2C(=O)O)cc1	CO	663/747
CN=c1ccc2c(-c3cccc(C(=O)O)c3C(=O)O)c3ccc(NC)cc3oc-2c1	O	522/545	CCN(CC)c1ccc2c(c1)P(C)(=O)C1=CC(=[N+](CC)CC)C=CC1=C2c1ccccc1C	O	694/712	CCN(CC)c1ccc(/C=C2\CCCc3c2[o+]c2cc(N(CC)CC)ccc2c3-c2ccccc2C(=O)O)c(O)c1	CO	699/753
C[N+](C)=c1ccc2c(-c3cccc(C(=O)O)c3C(=O)[O-])c3ccc(O)cc3oc-2c1	O	521/547	CN(C)c1ccc2c(c1)S(=O)(=O)C1=CC(=[N+](C)C)C=CC1=C2c1ccccc1	O	703/736	CCN(CC)c1ccc2c(c1)OC(=C1C=CC(=[N+](CC)CC)C=C1)C=C2c1ccccc1C(=O)O	O	598/648
C=CCN(CC=C)c1ccc2c(-c3cccc(C(=O)O)c3C(=O)[O-	O	548/570	Cc1ccccc1C1=C2C=CC(=[N+](C)C)C=C2S(=O)(=O)	O	703/742	CC[N+](CC)=C1C=CC(=C2C=C(c3ccccc3C(=O)O)c3cc	O	612/648

])c3ccc(=[N+](CC=C)CC=C)cc-3oc2c1			c2cc(N(C)C)ccc21			4c5c(c3O2)CCCN5CCC4)C=C1		
C=CCN(C)c1ccc2c(-c3cccc(C(=O)O)c3C(=O)[O-])c3cc/c(=[N+](\C)CC=C)cc-3oc2c1	O	549/572	COc1ccccc1C1=C2C=CC(=[N+](C)C)C=C2S(=O)(=O)c2cc(N(C)C)ccc21	O	707/747	CCN(CC)c1ccc2c(c1)O/C(=C/C=C1C=CC(=[N+](CC)CC)C=C1)C=C2c1ccccc1C(=O)O	O	676/727
CC1(C)C2=CC(=O)C=CC2=C(c2cccc(C(=O)O)c2C(=O)O)c2ccc(O)cc21	O	549/574	Cc1cccc(C)c1C1=C2C=CC(=[N+](C)C)C=C2S(=O)(=O)c2cc(N(C)C)ccc21	O	704/742	CC[N+](CC)=C1C=CC(=C/C=C2\C=C(c3ccccc3C(=O)O)c3cc4c5c(c3O2)CCCN5CCC4)C=C1	O	678/738
CN(C)c1ccc2c(-c3cccc(C(=O)O)c3C(=O)[O-])c3ccc(=[N+](C)C)cc-3oc2c1	O	551/573	COc1cccc(OC)c1C1=C2C=CC(=[N+](C)C)C=C2S(=O)(=O)c2cc(N(C)C)ccc21	O	710/752	CCN(CC)c1ccc2cc(/C=C/C=C3\CCCc4c3[o+]c3cc(N(CC)CC)ccc3c4-c3ccccc3C(=O)O)c(=O)oc2c1	CO	630/807
O=C(O)c1cccc(C2=c3cc4c5c(c3Oc3c2cc2c6c3CCCN6CCC2)CCC[N+]=5CCC4)c1C(=O)[O-]	O	579/599	CN(C)c1ccc2c(c1)oc1cc(N(C)C)ccc1[c+]2-c1ccccc1S(=O)(=O)[O-]	CO	550/568	CCN(CC)c1ccc2cc(/C=C/C=C/C=C3\CCCc4c3[o+]c3cc(N(CC)CC)ccc3c4-c3ccccc3C(=O)O)c(=O)oc2c1	CO	652/830
CN=C1C=CC2=C(c3cccc(C(=O)O)c3C(=O)O)c3ccc(NC)cc3CC2=C1	O	585/609	CN1c2cc3c(cc2-c2sc4ccccc4c2C1(C)c1ccccc1)C(c1ccc(S(=O)(=O)O)cc1S(=O)(=O)[O-])=c1cc2c(cc1O3)=[N+](C)C(C)(c1ccccc1)c1c-2sc2ccccc12	CO	613/638	CCN(CC)c1ccc2c(c1)oc(=O)c1c(-c3ccccc3C(=O)O)c3ccc(=[N+](CC)CC)cc-3oc12	CCO	591/637
CN(C)c1ccc2c(c1)C(C)(C)C1=CC(=[N+](C)C)	O	611/636	CN(C)c1ccc2c(c1)[Si](C)(C)C1=CC(=[	O	634/653	CC[N+](CC)=c1ccc2c(-c3ccccc3C(=O	CCO	598/651

C=CC1=C2c1cccc(C(=O)O)c1C(=O)[O-]			N+](C)C)C=CC1=C2			)O)c3c(=O)oc4ccccc4c3oc-2c1		
C=CCN(C)c1ccc2c(c1)C(C)(C)C1=C/C(=[N+](/C)CC=C)C=CC1=C2c1cccc(C(=O)O)c1C(=O)[O-]	O	611/636	Cc1ccccc1C1=C2C=CC(=[N+](C)C)C=C2[Si](C)(C)c2cc(N(C)C)ccc21	O	646/660	CC[N+](CC)=c1ccc2c(-c3ccccc3C(=O)O)c3c(=O)oc4c5c6c(cc4c3oc-2c1)CCCN6CCC5	CCO	612/674
CN(C)c1ccc2c(c1)C(C)(C)c1cc3c(cc1=C2c1cccc(C(=O)O)c1C(=O)[O-])CC[N+]=3C	O	621/648	Cc1ccccc1C1=C2C=CC(=[N+](C)C)C=C2[Ge](C)(C)c2cc(N(C)C)ccc21	O	635/649	COC(=O)c1ccccc1-c1c2ccc(=N)cc-2oc2c1ccc1cccc(OC)c12	O	538/596
CN(C)c1ccc2c(c1)C(C)(C)c1c3c4c(cc1=C2c1cccc(C(=O)O)c1C(=O)[O-])CCC[N+]=4CCC3	O	624/644	Cc1ccccc1C1=c2cc3c(cc2[Si](C)(C)c2cc4c(cc21)CCCN4C)=[N+](C)CCC3	O	674/689	COC(=O)c1ccccc1-c1c2ccc(=[N+](C)C)cc-2oc2c1ccc1cccc(O)c12	O	567/614
CN1CCc2cc3c(cc21)C(C)(C)c1cc2c(cc1=C3c1cccc(C(=O)O)c1C(=O)[O-])CC[N+]=2C	O	636/661	CN1CC2CCCN2c2cc3c(cc21)C(c1ccccc1C(=O)O)=c1cc2c4c(c1O3)CCC[N+]=4CCC2	O	586/644	COC(=O)c1ccccc1-c1c2ccc(=[N+](C)C)cc-2oc2c1ccc1cccc(OC)c12	O	568/606
CN(C)c1ccc2c(c1)C(C)(C)c1cc3c(cc1=C2c1cccc(C(=O)O)c1C(=O)[O-])C=CC(C)(C)[N+]=3C	O	641/678	N=c1ccc2c(-c3ccccc3C(=O)O)c3ccc(N)cc3oc-2c1	O	479/520	O=C([O-])c1ccccc1C1=c2cc3c4c(c2Oc2c1ccc1ccc(O)c21)CCC[N+]=4CCC3	O	585/624
CC1(C)c2c(cc3c4c2CCCN4CCC3)C(c2cccc(C(=O)O)c2C(=O)[O-])=c2cc3c4c(c21)CCC[N+]=4CCC3	O	642/658	CN=c1ccc2c(-c3ccccc3C(=O)O)c3ccc(NC)cc3oc-2c1	O	530/553	COc1cccc2ccc3c(c12)Oc1c2c4c(cc1=C3c1ccccc1C(=O)[O-])CCC[N+]=4CCC2	O	582/622
CN(C)c1ccc2c(c1)[Si](C)(C)C1=CC(=[N+](C)C)C=CC1=C2c	O	649/669	CN(C)c1ccc2c(-c3ccccc3C(=O)[O-])c3ccc(=[N+]	O	548/572	CC[N+](CC)=c1ccc2c(-c3ccccc3C(=O)O)c3c(ccc4cc	O	560/605

1cccc(C(=O)O)c1C(=O)[O-]			(C)C)cc-3oc2c1			c(O)cc43)oc-2c1		
CN(C)c1ccc2c(c1)[Si](C)(C)c1c3c4c(cc1=C2c1cccc(C(=O)O)c1C(=O)[O-])CCC[N+]=4CCC3	O	663/683	O=C([O-])c1ccccc1-c1c2ccc(=[N+]3CCCC3)cc-2oc2cc(N3CCCC3)ccc12	O	553/576	COC(=O)c1ccccc1-c1c2ccc(=[N+](C)C)cc-2oc2c1ccc1ccc(N)c12	O	598/770
CN(C)c1ccc2c(c1)[Si](C)(C)c1cc3c(cc1=C2c1cccc(C(=O)O)c1C(=O)[O-])CC[N+]=3C	O	668/693	O=C([O-])c1ccccc1-c1c2ccc(=[N+]3CCCCC3)cc-2oc2cc(N3CCCCC3)ccc12	O	560/586	COC(=O)c1ccccc1C1=c2cc3c4c(c2Oc2c1ccc1cccc(N)c21)CCC[N+]=4CCC3	O	601/740
CN1CCc2cc3c(cc21)[Si](C)(C)c1c2c4c(cc1=C3c1cccc(C(=O)O)c1C(=O)[O-])CCC[N+]=4CCC2	O	687/708	O=C([O-])c1ccccc1-c1c2ccc(=[N+]3CCCCCC3)cc-2oc2cc(N3CCCCCC3)ccc12	O	560/583	N=c1ccc2c(-c3ccccc3C(=O)O)c3ccc4cc(N)ccc4c3oc-2c1	O	533/645
CC1=CC(C)(C)[N+](C)=c2cc3c(cc21)=C(c1cccc(C(=O)O)c1C(=O)[O-])c1ccc(N(C)C)cc1[Si]3(C)C	O	687/719	CCN1c2cc3oc4cc(=N)ccc-4c(-c4ccccc4C(=O)O)c3cc2N(CC)C2CCCCC21	CCO	565/648	C[N+](C)=c1ccc2c(-c3ccccc3C(=O)[O-])c3ccc4cc(N)ccc4c3oc-2c1	O	564/612
CN1CCc2cc3c(cc21)[Si](C)(C)c1cc2c(cc1=C3c1cccc(C(=O)O)c1C(=O)[O-])CC[N+]=2C	O	694/721	CCN1c2cc3oc4cc(=[N+](CC)CC)ccc-4c(-c4ccccc4C(=O)[O-])c3cc2N(CC)C2CCCCC21	CCO	584/660	CN(C)c1ccc2c(ccc3c(-c4ccccc4C(=O)O)c4ccc(=N)cc-4oc32)c1	O	568/652
CNc1ccc2c3c(ccc2c1)C(c1ccccc1C(=O)[O-])=c1cc2c4c(c1O3)CCC[N+]=4CCC2	O	585/624	CCN1c2cc3c(cc2N(CC)C2CCCCC21)C(c1ccccc1C(=O)[O-])=c1cc2c4c(c1O3)CCC[N+]=4CCC2	CCO	593/587	Nc1ccc2c3c(ccc2c1)C(c1ccccc1C(=O)[O-])=c1cc2c4c(c1O3)CCC[N+]=4CCC2	O	577/612

Table S10. Atom-level descriptors used for molecular graph representation.

Feature	Description	Method
Atomic number	Element identity	GetAtomicNum()
Degree	Number of bonded neighbors	GetTotalDegree()
Valence	Explicit valence	GetTotalValence()
No. of Hs	total number of Hs on the atom	GetTotalNumHs()
Hybridization	sp / sp^2 / sp^3 / other	GetHybridization()
Aromaticity	Aromatic or non-aromatic	GetIsAromatic()
SASA	Solvent-accessible surface area	GetProp('SASA')
Partial charge	Atomic partial charge	GetDoubleProp('_GasteigerCharge')

Table S11. Optimized hyperparameters for pre-trained model.

Name	GAT	GCN
Number of graph layers	3	3
Hidden dimension	8×32; 32×32; 32×32	8×128; 128×128; 128×256
Attention heads	7	\
Activation function	ReLU	ReLU
Dropout rate	0	0
Projection head	FC: 64× 1024 × 512; MLP: 512×128×32	FC: 256×512; MLP: 512×256×128×64
Batch size	8	8
Learning rate	2×10^{-4}	2×10^{-4}
τ	0.1	0.1

Table S12. Optimized hyperparameters for machine learning baseline models.

Model	λ_{abs}	λ_{emi}
RandomForest	max_depth = 3; min_samples_split = 2; n_estimators = 300	max_depth = None; min_samples_split = 2; n_estimators = 300
SVM	C = 0.01; epsilon = 0.5; gamma = scale; kernel = linear	C = 1.0; epsilon = 0.5; gamma = scale; kernel = linear
KNN	n_neighbors = 10; p = 2; weights = uniform	n_neighbors = 3; p = 1; weights = distance
XGBoost	colsample_bytree = 0.8; learning_rate = 0.01; max_depth = 4; n_estimators = 200; subsample = 0.8	colsample_bytree = 0.8; learning_rate = 0.1; max_depth = 4; n_estimators = 300; subsample = 0.8

Table S13. Details of fine-tuning models.

Model	λ_{abs}	λ_{emi}
GCN_ST	FC layers of solvents: 7×1024; Ir = 3×10^{-3}; batch size = 8	FC layers of solvents: 7×32; Ir = 1.5×10^{-3}; batch size = 4; drop out = 0.3
GAT_ST	FC layers of solvents: 7×128; Ir = 3×10^{-3}; batch size = 32	FC layers of solvents: 7×256; Ir = 7×10^{-3}; batch size = 16
GCN_MT	FC layers of solvents: 7×64; Ir = 8×10^{-3}; batch size = 8	
GAT_MT	FC layers of solvents: 7×512; Ir = 6×10^{-3}; batch size = 16	

Supplementary Methods

Section A Construction of machine learning models

Molecular fingerprints

To establish classical machine learning baselines alongside graph neural networks, molecules were additionally encoded using Morgan circular fingerprint. Specifically, 2,048-bit Morgan fingerprints with a radius of 2 were generated from SMILES strings using RDKit. Each fingerprint was represented as a binary vector, where each bit indicates the presence or absence of a specific circular substructure. The same fingerprint representation was used consistently across all baseline models, including Random Forest, Support Vector Machine, k-Nearest Neighbors, and XGBoost.

For machine learning baseline models, the Morgan fingerprint vector X_{mol} and the solvent descriptor vector X_{solvent} were concatenated to form the final input feature vector:

$$X = [X_{\mathrm{mol}} \parallel X_{\mathrm{solvent}}]$$

where $X_{\mathrm{mol}} \in \mathbb{R}^{2048}$ and $X_{\mathrm{solvent}} \in \mathbb{R}^{7}$.

This unified input representation was used for all classical machine learning models to ensure fair comparison with graph-based deep learning approaches.

To ensure methodological consistency, identical solvent descriptors were used across both graph neural network models and classical machine learning baselines. Differences in predictive performance therefore primarily reflect

differences in molecular representation learning and model architecture, rather than discrepancies in input information.

Classical machine learning model

Several classical machine learning regression models were implemented and evaluated to establish baseline performance for absorption and emission wavelength prediction, including Random Forest (RF), Support Vector Machine (SVM), k-Nearest Neighbors (KNN), and Extreme Gradient Boosting (XGBoost). For all machine learning models, molecular representations were constructed by concatenating Morgan fingerprints with solvent descriptor vectors.

For each machine learning model, hyperparameters were selected using grid search over a predefined parameter space. Each machine learning model was trained using the optimized hyperparameters and evaluated using ten-fold cross-validation. Performance metrics, including coefficient of determination (R^2), mean absolute error (MAE), and root mean squared error (RMSE), were computed based on the cross-validation results.

Section B Collection of rhodamine optical data

The labeled dataset for downstream prediction of maximum absorption (λ_{abs}) and emission (λ_{em}) wavelengths of rhodamine derivatives was curated from publicly available literature and patent sources [1-28].

1. Koide, Y.; Urano, Y.; Kenmoku, S.; Kojima, H.; Nagano, T. Design and Synthesis of Fluorescent Probes for Selective Detection of Highly Reactive

Oxygen Species in Mitochondria of Living Cells. *Journal of the American Chemical Society* **2007,** *129* (34), 10324-10325.

2. Kamiya, M.; Asanuma, D.; Kuranaga, E.; Takeishi, A.; Sakabe, M.; Miura, M.; Nagano, T.; Urano, Y. Β-Galactosidase Fluorescence Probe with Improved Cellular Accumulation Based on a Spirocyclized Rhodol Scaffold. *Journal of the American Chemical Society* **2011,** *133* (33), 12960-12963.

3. Li, Z.; Pande, P. Fluorescent Dyes Containing Phosphorus or Arsenic. Google Patents: 2013.

4. Sakabe, M.; Asanuma, D.; Kamiya, M.; Iwatate, R. J.; Hanaoka, K.; Terai, T.; Nagano, T.; Urano, Y. Rational Design of Highly Sensitive Fluorescence Probes for Protease and Glycosidase Based on Precisely Controlled Spirocyclization. *Journal of the American Chemical Society* **2013,** *135* (1), 409-414.

5. Uno, S.-n.; Kamiya, M.; Yoshihara, T.; Sugawara, K.; Okabe, K.; Tarhan, M. C.; Fujita, H.; Funatsu, T.; Okada, Y.; Tobita, S., et al. A Spontaneously Blinking Fluorophore Based on Intramolecular Spirocyclization for Live-Cell Super-Resolution Imaging. *Nat. Chem.* **2014,** *6* (8), 681-689.

6. Liu, C.; Best, Q. A.; Suarez, B.; Pertile, J.; McCarroll, M. E.; Scott, C. N. Cycloalkyl-Aminomethylrhodamines: Ph Dependent Photophysical Properties Tuned by Cycloalkane Ring Size. *J. Fluoresc.* **2015,** *25* (2), 231-237.

7. Zhu, W.; Chai, X.; Wang, B.; Zou, Y.; Wang, T.; Meng, Q.; Wu, Q. Spiroboronate Si-Rhodamine as a near-Infrared Probe for Imaging Lysosomes

Based on the Reversible Ring-Opening Process. *Chem. Commun.* **2015,** *51* (47), 9608-9611.

8. Grimm, J. B.; Klein, T.; Kopek, B. G.; Shtengel, G.; Hess, H. F.; Sauer, M.; Lavis, L. D. Synthesis of a Far-Red Photoactivatable Silicon-Containing Rhodamine for Super-Resolution Microscopy. *Angew. Chem. Int. Ed.* **2016,** *55* (5), 1723-1727.

9. Iwatate, R. J.; Kamiya , M.; Urano , Y. Asymmetric Rhodamine-Based Fluorescent Probe for Multicolour in Vivo Imaging. *Chemistry – A European Journal* **2016,** *22* (5), 1696-1703.

10. Matsuzaki, H.; Kamiya, M.; Iwatate, R. J.; Asanuma, D.; Watanabe, T.; Urano, Y. Novel Hexosaminidase-Targeting Fluorescence Probe for Visualizing Human Colorectal Cancer. *Bioconjugate Chem.* **2016,** *27* (4), 973-981.

11. Chiba, M.; Ichikawa, Y.; Kamiya, M.; Komatsu, T.; Ueno, T.; Hanaoka, K.; Nagano, T.; Lange, N.; Urano, Y. An Activatable Photosensitizer Targeted to Γ-Glutamyltranspeptidase. *Angew. Chem. Int. Ed.* **2017,** *56* (35), 10418-10422.

12. Chai, X.; Xiao, J.; Li, M.; Wang, C.; An, H.; Li, C.; Li, Y.; Zhang, D.; Cui, X.; Wang, T. Bridge-Caging Strategy in Phosphorus-Substituted Rhodamine for Modular Development of near-Infrared Fluorescent Probes. *Chemistry – A European Journal* **2018,** *24* (54), 14506-14512.

13. Ito, H.; Kawamata, Y.; Kamiya, M.; Tsuda-Sakurai, K.; Tanaka, S.; Ueno, T.; Komatsu, T.; Hanaoka, K.; Okabe, S.; Miura, M., et al. Red-

Shifted Fluorogenic Substrate for Detection of Lacz-Positive Cells in Living Tissue with Single-Cell Resolution. *Angew. Chem. Int. Ed.* **2018,** *57* (48), 15702-15706.

14. Uno, S.-n.; Kamiya, M.; Morozumi, A.; Urano, Y. A Green-Light-Emitting, Spontaneously Blinking Fluorophore Based on Intramolecular Spirocyclization for Dual-Colour Super-Resolution Imaging. *Chem. Commun.* **2018,** *54* (1), 102-105.

15. Chi, W.; Qiao, Q.; Wang, C.; Zheng, J.; Zhou, W.; Xu, N.; Wu, X.; Jiang, X.; Tan, D.; Xu, Z., et al. Descriptor $\Delta G_{C\text{-}O}$ Enables the Quantitative Design of Spontaneously Blinking Rhodamines for Live-Cell Super-Resolution Imaging. *Angew. Chem. Int. Ed.* **2020,** *59* (45), 20215-20223.

16. Tachibana, R.; Kamiya, M.; Morozumi, A.; Miyazaki, Y.; Fujioka, H.; Nanjo, A.; Kojima, R.; Komatsu, T.; Ueno, T.; Hanaoka, K., et al. Design of Spontaneously Blinking Fluorophores for Live-Cell Super-Resolution Imaging Based on Quantum-Chemical Calculations. *Chem. Commun.* **2020,** *56* (86), 13173-13176.

17. Tachibana, R.; Kamiya, M.; Suzuki, S.; Morokuma, K.; Nanjo, A.; Urano, Y. Molecular Design Strategy of Fluorogenic Probes Based on Quantum Chemical Prediction of Intramolecular Spirocyclization. *Commun. Chem.* **2020,** *3* (1), 82.

18. Luo, J.; Zhang, H.; Guan, J.; An, B.; Peng, J.; Zhu, W.; Wei, N.; Zhang, Y. Detection of Lipase Activity in Human Serum Based on a Ratiometric Fluorescent Probe. *New J. Chem.* **2021,** *45* (21), 9561-9568.

19. Obara, R.; Kamiya, M.; Tanaka, Y.; Abe, A.; Kojima, R.; Kawaguchi, T.; Sugawara, M.; Takahashi, A.; Noda, T.; Urano, Y. Γ-Glutamyltranspeptidase (Ggt)-Activatable Fluorescence Probe for Durable Tumor Imaging. *Angew. Chem. Int. Ed.* **2021,** *60* (4), 2125-2129.

20. Shao, J.; Liu, Y.; Yan, J.; Yan, Z.-Y.; Wu, Y.; Ru, Z.; Liao, J.-Y.; Miao, X.; Qian, L. Prediction of Maximum Absorption Wavelength Using Deep Neural Networks. *J. Chem. Inf. Model.* **2022,** *62* (6), 1368-1375.

21. Bucevičius, J.; Gerasimaitė, R.; Kiszka, K. A.; Pradhan, S.; Kostiuk, G.; Koenen, T.; Lukinavičius, G. A General Highly Efficient Synthesis of Biocompatible Rhodamine Dyes and Probes for Live-Cell Multicolor Nanoscopy. *Nat. Commun.* **2023,** *14* (1), 1306.

22. Zheng, Y.; Ye, Z.; Xiao, Y. Subtle Structural Translation Magically Modulates the Super-Resolution Imaging of Self-Blinking Rhodamines. *Anal. Chem.* **2023,** *95* (8), 4172-4179.

23. Zheng, Y.; Ye, Z.; Zhang, X.; Xiao, Y. Recruiting Rate Determines the Blinking Propensity of Rhodamine Fluorophores for Super-Resolution Imaging. *Journal of the American Chemical Society* **2023,** *145* (9), 5125-5133.

24. Battula, H.; Reddy Bollareddy, S.; Vamsi Krishna Venuganti, V.; Jayanty, S. Rhodamine B Amide Derivatives with Anticancer Activity and

Fluorescence Properties for Potential Theranostic Applications. *ChemistrySelect* **2024,** *9* (1), e202302367.

25. Mondal, M.; Das, R.; Pal, R.; Nag, S.; Banerjee, P. Design and Application of Rhodamine Derivatives in Redox Biology: A Roadmap of the Last Decade Towards Artificial Intelligence. *J. Mater. Chem. A* **2024,** *12* (33), 21626-21676.

26. Zhou, M.; Jin, T.; Liu, Y.; Wang, S.; Feng, J.; Shao, S.; Lu, C.; Jin, G. Design, Synthesis, Cell Imaging, and Bioactivity Assessment of Novel Rhodamine-Pyrimidine Nido-Carborane Derivatives as Fluorescent Anticancer Agents. *J. Mol. Struct.* **2024,** *1299*, 137211.

27. Guan, Y.; Wu, W.; Su, J.; Zhang, L. Synthesis of Rhodamine B Amine Derivatives with Improved Light Resistance and Its Application in Thermochromic Materials. *Dyes Pigm.* **2025,** *233*, 112529.

28. Liu, C.; Feng, C.; Liu, Y.; Wu, Y.; Yao, H.; He, S.; Zeng, X. Construction of a Novel Nir-Emissive Rhodamine Derivative for Monitoring Mitochondrial Viscosity in Ferroptosis. *Spectrochim. Acta, Part A* **2025,** *328*, 125486.

Published 32 rhodamines that designed and synthesized in our group, which were used for the external validation and named as D_{32}. [29-36]

29. Zhang, H.; Shi, L.; Li, K.; Liu, X.; Won, M.; Liu, Y.-Z.; Choe, Y.; Liu, X.-Y.; Liu, Y.-H.; Chen, S.-Y., et al. Discovery of an Ultra-Rapid and Sensitive Lysosomal Fluorescence Lipophagy Process. *Angew. Chem. Int. Ed.* **2022,** *61* (11), e202116439.

30. Chen, Y.-J.; Zhang, H.; Liu, Y.-Z.; Shi, L.; Xiang, F.-F.; Lin, R.-D.; Liu, Y.-H.; Chen, S.-Y.; Yu, X.-Q.; Li, K. Rational Design of Ph-Independent and High-Fidelity near-Infrared Tunable Fluorescent Probes for Tracking Leucine Aminopeptidase in Vivo. *ACS Sens.* **2023,** *8* (6), 2359-2367.

31. Ran, X.-Y.; Chen, P.; Liu, Y.-Z.; Shi, L.; Chen, X.; Liu, Y.-H.; Zhang, H.; Zhang, L.-N.; Li, K.; Yu, X.-Q. Rational Design of Polymethine Dyes with Nir-Ii Emission and High Photothermal Conversion Efficiency for Multimodal-Imaging-Guided Photo-Immunotherapy. *Adv. Mater.* **2023,** *35* (12), 2210179.

32. Liu, Y.-H.; Zhang, H.; Yu, K.-K.; Pei, X.-F.; Xu, J.-N.; Chen, S.-Y.; Yu, X.-Q.; Li, K. Si–Rhodamine Derivative with a Large Stokes Shift for Elisa-Based Detection of Sars-Cov-2. *Sensors & Diagnostics* **2024,** *3* (5), 822-826.

33. Liu, Y.-Z.; Ran, X.-Y.; Zhou, D.-H.; Zhang, H.; Chen, Y.-J.; Xu, J.-X.; Chen, S.-Y.; Kong, Q.-Q.; Yu, X.-Q.; Li, K. Novel Dibenzofulvene-Based Nir-Ii Emission Phototheranostic Agent with an 82.6% Photothermal Conversion Efficiency for Photothermal Therapy. *Adv. Funct. Mater.* **2024,** *34* (8), 2311365.

34. Xiang, F.-F.; Zhang, H.; Wu, Y.-L.; Chen, Y.-J.; Liu, Y.-Z.; Chen, S.-Y.; Guo, Y.-Z.; Yu, X.-Q.; Li, K. Machine-Learning-Assisted Rational Design of Si—Rhodamine as Cathepsin-Ph-Activated Probe for Accurate Fluorescence Navigation. *Adv. Mater.* **2024,** *36* (31), 2404828.

35. Zhang, L.-N.; Chen, S.-Y.; Shi, L.; Ran, X.-Y.; Zhang, H.; Moon, H.; Lee, Y.; Yu, X.-Q.; Kim, J. S.; Li, K. De Novo Construction of Pka-Tunable Xanthene Molecules for Ph Sensitive Fluorescence Navigation. *Adv. Funct. Mater.* **2025,** *35* (2), 2412595.

36. Zhang, L.-N.; Ran, X.-Y.; Zhang, H.; Zhao, Y.; Zhou, Q.; Chen, S.-Y.; Yang, C.; Yu, X.-Q.; Li, K. Molecular Engineering of Xanthene Dyes with 3d Multimodal-Imaging Ability to Guide Photothermal Therapy. *Adv. Healthcare Mater.* **2025,** *14* (1), 2402295.

Section C Quantum mechanics (QM) calculation

All quantum chemical calculations were performed with Gaussian 09 [1]. For each molecule, the ground state geometry was optimized at the B3LYP/6-31G(d) level [2] using the SMD implicit solvation model and the GD3BJ dispersion correction, with tight optimization criteria and an initial force constant evaluation. Frequency calculations were subsequently carried out at the same level of theory to confirm that the optimized structure corresponds to a true minimum on the potential energy surface. The maximum absorption wavelength was obtained from vertical excitation energies computed by time-dependent density functional theory (TD-DFT) at the B3LYP/6-31G(d) level with SMD and GD3BJ, requesting the lowest 10 excited states based on the optimized S0 geometry. The maximum emission wavelength was calculated by optimizing the first singlet excited state using TD-DFT with td opt at the same B3LYP/6-31G(d) level under SMD and GD3BJ, followed by evaluating the corresponding emission transition from the optimized excited state geometry.

Section D Pre-training Process

For a graph containing N nodes, 25% of the nodes were randomly sampled and replaced by zero vectors of the same dimensionality. As all original atom-level features were strictly nonzero-valued, the zero-vector served as an uninformative placeholder, effectively removing chemical information from

the selected nodes while preserving the original graph topology. Every molecule was generated two augmented views denoted as $t_1(G)$and $t_2(G)$.

Two graph neural network architectures were used as encoders during contrastive pretraining, a graph attention network (GAT) and a graph convolutional network (GCN) and each graph convolution layers followed by a ReLU activation. After the final graph convolution layer, node embeddings were aggregated into a fixed-length graph-level representation using global max pooling, and then was passed through a projection head composed of a fully connected (FC) layer followed by a multilayer perceptron (MLP). The parameters were optimized using the Adam optimizer with a fixed learning rate and 100 epochs. Nonlinear activation functions were applied after each graph convolution layer and a fully-connected layer.

Section E Synthesis of designed molecules

The synthetic routes of compounds P-C-ARh, I-C-ARh, and Q-C-ARh are shown in Figure S4.

Synthesis of compound 2–1:

1,4-Dichlorobutane (12.9 g, 60 mmol), m-bromoaniline (13.4 g, 78 mmol), and potassium carbonate (18.2 g, 132 mmol) were accurately weighed and dissolved in 150 mL of acetonitrile. The reaction mixture was heated under reflux for 12 h, and the reaction progress was monitored by TLC. After completion, potassium carbonate was removed by filtration, and the filtrate was concentrated under reduced pressure using a rotary evaporator to remove

the solvent. The resulting crude mixture was used directly in the subsequent step without further purification.

Synthesis of compound 2–2:

To a 250 mL round-bottom flask was added 20 mL of DMF, and the mixture was cooled at 0 °C for 10 min. Phosphorus oxychloride (8.4 mL, 90 mmol) was then added dropwise to the system via a constant-pressure dropping funnel, and the reaction was allowed to proceed at 0 °C for an additional 30 min. Subsequently, a DMF solution (50 mL) of compound 2–1 (13.5 g, 60 mmol) was added dropwise to the system via a constant-pressure dropping funnel. After the addition was complete, the reaction mixture was heated to 80 °C and stirred for 8 h, with the reaction progress monitored by TLC. After completion of the reaction, the mixture was poured into 500 mL of ice water and then extracted with dichloromethane. The combined organic phases were dried over anhydrous sodium sulfate, and the solvent was removed under reduced pressure using a rotary evaporator. The resulting mixture was purified by silica gel column chromatography (200–300 mesh), using petroleum ether/ethyl acetate as the eluent, to afford the target compound 2–2 as a colorless oil (15.6 g, yield 83%). ^{1}H NMR (400 MHz, $CDCl_3$) δ (ppm): 10.03 (s, 1H), 7.75 (d, J = 8.8 Hz, 1H), 6.63 (d, J = 2.4 Hz, 1H), 6.46 (dd, J = 9.2 Hz, 2.5 Hz, 1H), 3.33 (t, J = 6.7 Hz, 4H), 2.02 (t, J = 6.7 Hz, 4H). ^{13}C NMR (101 MHz, $CDCl_3$) δ (ppm): 190.2, 152.1, 131.1, 129.7, 121.4, 114.8, 110.8, 47.7, 25.3. ESI(+)-HRMS (m/z): $[M + H]^+$ calcd. for: 254.0181, found: 254.0176.

Synthesis of compound 2–3:

Compound 2–2 (6.3 g, 25 mmol) was accurately weighed and placed in a 100 mL round-bottom flask, followed by the addition of methanol (50 mL). The mixture was cooled at 0 °C for 10 min, after which sodium borohydride (1.2 g, 30 mmol) was slowly added portionwise. The reaction was maintained at 0 °C for 30 min, and the progress was monitored by TLC. After completion, 100 mL of saturated brine was added to the reaction mixture, followed by extraction with dichloromethane. The combined organic phases were dried over anhydrous sodium sulfate, and the solvent was removed under reduced pressure using a rotary evaporator. The resulting white solid was the pure target compound 2–3 (8.2 g, yield 99%). ^{1}H NMR (400 MHz, $CDCl_3$) δ (ppm): 7.19 (s, 1H), 6.71 (d, J = 2.5 Hz, 1H), 6.44 (dd, J = 8.4 Hz, 2.5 Hz, 1H), 4.61 (d, J = 6.2 Hz, 2H), 3.27–3.20 (m, 4H), 1.99 (t, J = 6.6 Hz, 4H). ^{13}C NMR (101 MHz, $CDCl_3$) δ (ppm): 148.5, 130.6, 125.8, 124.5, 115.1, 110.7, 65.2, 47.6, 25.4.

Synthesis of compound 2–4:

Compounds 2–3 (6.35 g, 25 mmol) and 2–2 (5.6 g, 25 mmol) were accurately weighed and placed in a 250 mL round-bottom flask, dissolved in dichloromethane (100 mL). Under stirring, a solution of boron trifluoride diethyl etherate (5 mL, 40 mmol) was added dropwise to the system via syringe. The reaction was allowed to proceed at room temperature for 30 min, with the progress monitored by TLC. After completion, 100 mL of dilute hydrochloric acid was added to the reaction mixture, and the layers were

separated. The organic phase was washed twice with saturated brine. The combined organic layers were dried over anhydrous sodium sulfate, and the solvent was removed under reduced pressure using a rotary evaporator. The resulting mixture was purified by silica gel column chromatography (200–300 mesh), first eluting with petroleum ether/ethyl acetate and then increasing the polarity to dichloromethane/methanol, to afford the target compound 2–4 as a white solid (7.5 g, yield 65%). ^{1}H NMR (400 MHz, $CDCl_3$) δ (ppm): 6.82 (d, J = 8.5 Hz, 2H), 6.77 (d, J = 2.5 Hz, 2H), 6.40 (dd, J = 8.5 Hz, 2.5 Hz, 2H), 3.98 (s, 2H), 3.22 (t, J = 6.6 Hz, 8H), 2.02–1.92 (m, 8H). ^{13}C NMR (101 MHz, $CDCl_3$) δ (ppm): 147.3, 130.8, 125.9, 125.6, 115.1, 110.9, 47.6, 39.9, 25.4. ESI(+)-HRMS (m/z): $[M + H]^+$ calcd. for: 463.0384, found: 463.0374.

Synthesis of compound 2–5:

Compound 2–4 (1.38 g, 3 mmol) was accurately weighed and placed in a 100 mL two-neck round-bottom flask. The system was evacuated and backfilled with nitrogen using a dual-manifold line to remove moisture and oxygen. Anhydrous tetrahydrofuran (40 mL), freshly distilled, was added to the system via syringe. The reaction mixture was cooled in a −78 °C low-temperature bath for 10 min. After complete cooling, a 2.5 M solution of *n*-butyllithium (2.52 mL, 6.3 mmol) was added dropwise to the system via syringe. After completion of the addition, the reaction was maintained at −78 °C for 1 h. Acetone (0.22 mL, 3 mmol) was then added to the system, and the reaction was continued at −78 °C for an additional 1 h. Subsequently, water (20 mL) was added, and the

reaction mixture was removed from the low-temperature bath and stirred at room temperature for 1 h. The reaction vessel was then opened, and the mixture was extracted with dichloromethane (50 mL). The organic phase was dried over anhydrous sodium sulfate, and the solvent was removed under reduced pressure using a rotary evaporator. The residue was dissolved again in dry dichloromethane (30 mL) and cooled at 0 °C for 10 min. Anhydrous aluminum chloride (3.99 g, 30 mmol) was added portionwise to the system, after which the reaction was stirred at room temperature overnight. Upon completion, water was added dropwise to quench the reaction. The mixture was then extracted with dichloromethane, and the organic phase was dried over anhydrous sodium sulfate and concentrated under reduced pressure. The resulting material was used directly in the subsequent oxidation step without further purification.

Synthesis of compound 2–6:

The crude product 2–5 obtained above was dissolved in acetone (100 mL), and the mixture was cooled in a −5 °C low-temperature bath for 10 min. After the system was completely cooled, potassium permanganate (1.42 g, 9 mmol) was added portionwise and slowly over approximately 4 h. The reaction mixture was then maintained at −5 °C for 2 h, followed by warming to room temperature and stirring for an additional 4 h. The reaction progress was monitored by TLC. After completion, the mixture was filtered through Celite to remove the manganese dioxide formed during the reaction. The filter cake

was thoroughly washed with a dichloromethane/methanol mixed solvent (approximately 1000 mL) until the filtrate became colorless. The combined filtrates were concentrated under reduced pressure using a rotary evaporator. The resulting mixture was purified by silica gel column chromatography (200–300 mesh) using dichloromethane/methanol as the eluent to afford the target compound 2–6 as a gray-green solid (308 mg, yield 29%). ^{1}H NMR (400 MHz, $CDCl_3$) δ (ppm): 8.25 (d, J = 9.4 Hz, 2H), 6.62–6.57 (m, 4H), 3.44–3.35 (t, J = 6.6 Hz, 8H), 2.06–2.00 (t, J = 6.5 Hz, 8H), 1.69 (s, 6H). ^{13}C NMR (101 MHz, $CDCl_3$) δ (ppm): 181.1, 152.4, 150.5, 129.2, 119.4, 110.8, 107.5, 47.5, 38.0, 33.8, 25.4. ESI(+)-HRMS (m/z): $[M + H]^+$ calcd. for: 361.2280, found: 361.2262.

Synthesis of compound P–C-ARh:

Compound 2–6 (120 mg, 0.3 mmol) was accurately weighed and dissolved in dichloromethane (20 mL). Pyridine (189 mg, 0.19 mL, 2.4 mmol) was added to the system by syringe, and the reaction mixture was cooled in a 0 °C low-temperature bath for 10 min. Trifluoromethanesulfonic anhydride (423 mg, 0.26 mL, 1.5 mmol) was then added by syringe. The solution was observed to change immediately from light yellow to deep blue. The reaction mixture was maintained at 0 °C for 1 h. After TLC monitoring indicated complete consumption of compound 2–6, propylamine (177 mg, 0.25 mL, 3 mmol) was added by syringe. Upon addition of propylamine, the solution color instantly changed from deep blue to bright wine-red. The reaction mixture was then allowed to warm to room temperature and stirred for 10 min, after which the

solvent was removed under reduced pressure using a rotary evaporator. The resulting reddish-brown mixture was purified by silica gel column chromatography (200–300 mesh) using dichloromethane/methanol as the eluent to afford the target compound P–C-ARh as a wine-red solid (84 mg, yield 70%). ^1H NMR (400 MHz, $CDCl_3$) δ (ppm): 7.77 (s, 2H), 6.72 (s, 2H), 6.61 (s, 2H), 3.91 (s, 2H), 3.45 (t, J = 6.9 Hz, 8H), 2.07 (t, J = 6.4 Hz, 8H), 1.99 (d, J = 5.7 Hz, 4H), 1.95–1.88 (m, 2H), 1.61 (s, 6H), 0.96 (t, J = 7.4 Hz, 3H). ^{13}C NMR (101 MHz, $CDCl_3$) δ (ppm): 165.0, 151.1, 124.6, 121.4, 118.2, 115.0, 111.5, 51.5, 47.8, 40.6, 32.2, 25.3, 22.9, 11.0. ESI(+)-HRMS (m/z): $[M]^+$ calcd. for: 402.2904; found: 402.2897.

Synthesis of compound 2–7:

6-Bromoindole (4.8 g, 24.5 mmol) and sodium cyanoborohydride (6.16 g, 98.1 mmol) were accurately weighed and placed in a 250 mL round-bottom flask. Glacial acetic acid (100 mL) was added to dissolve the reaction mixture, which was then stirred at room temperature for 6 h. The reaction progress was monitored by TLC. After completion, the reaction mixture was neutralized to pH 7 with sodium hydroxide and extracted with dichloromethane (100 mL). The organic phase was collected and dried over anhydrous sodium sulfate. The solvent was removed under reduced pressure using a rotary evaporator. The resulting mixture was purified by silica gel column chromatography (200–300 mesh) using petroleum ether/ethyl acetate as the eluent to afford the target compound 2–7 as a colorless oil (6.74 g, yield 85%). ^1H NMR (400 MHz, $CDCl_3$)

δ (ppm): 6.94 (d, J = 7.7 Hz, 1H), 6.79 (d, J = 7.8 Hz, 1H), 6.70 (s, 1H), 3.72 (s, 1H), 3.54 (t, J = 8.2 Hz, 2H), 2.95 (t, J = 8.2 Hz, 2H).

Synthesis of compound 2–8:

Compound 2–7 (1.96 g, 10 mmol) and potassium carbonate (2.76 g, 20 mmol) were accurately weighed and placed in a 250 mL round-bottom flask. Acetonitrile (100 mL) was added to dissolve the mixture, after which methyl iodide (0.93 mL, 15 mmol) was added to the system via syringe. The reaction mixture was heated under reflux. The reaction progress was monitored by TLC. After completion, the insoluble potassium carbonate was removed by filtration. The filtrate was collected, and the solvent was removed under reduced pressure using a rotary evaporator. The resulting reddish-brown mixture was purified by silica gel column chromatography (200–300 mesh) using petroleum ether/ethyl acetate as the eluent to afford the target compound 2–8 as a pale yellow oil (6.74 g, yield 85%). ^{1}H NMR (400 MHz, $CDCl_3$) δ (ppm): 6.88 (d, J = 7.7 Hz, 1H), 6.74 (d, J = 7.8 Hz, 1H), 6.53 (s, 1H), 3.32 (t, J = 8.2 Hz, 2H), 2.87 (t, J = 8.2 Hz, 2H), 2.72 (s, 1H). ^{13}C NMR (101 MHz, $CDCl_3$) δ (ppm): 154.7, 129.2, 125.2, 121.0, 120.0, 110.0, 56.0, 35.6, 28.2. ESI(+)-HRMS (m/z): $[M + H]^+$ calcd. for: 443.1888, found: 443.1907.

Synthesis of compound 2–9:

Compound 2–8 (16.6 g, 79 mmol) and 37% aqueous formaldehyde solution (3.85 g, 47.5 mmol) were accurately weighed and placed in a 250 mL round-bottom flask. Glacial acetic acid (100 mL) was added to dissolve the reaction

mixture, which was allowed to react at room temperature overnight. The reaction progress was monitored by TLC. After completion, the reaction mixture was neutralized to pH 7 with sodium hydroxide and extracted with dichloromethane (100 mL). The organic phase was collected and dried over anhydrous sodium sulfate. The solvent was removed under reduced pressure using a rotary evaporator. The resulting mixture was purified by silica gel column chromatography (200–300 mesh), first eluting with petroleum ether/ethyl acetate and then increasing the polarity to dichloromethane/methanol, to afford the target compound 2–9 as a colorless oil (7.4 g, yield 42%). ^{1}H NMR (400 MHz, $CDCl_3$) δ (ppm): 6.70 (s, 2H), 6.64 (s, 2H), 3.96 (s, 2H), 3.27 (t, J = 8.1 Hz, 1H), 2.81 (t, J = 7.9 Hz, 1H), 2.72 (s, 6H). ^{13}C NMR (101 MHz, $CDCl_3$) δ (ppm): 153.0, 130.2, 128.2, 126.0, 123.0, 110.8, 56.2, 40.5, 36.0, 28.3. ESI(+)-HRMS (m/z): $[M + H]^+$ calcd. for: 435.0071, found: 435.0059.

Synthesis of compound 2–10:

Compound 2–9 (1.25 g, 2.88 mmol) was accurately weighed and placed in a 100 mL two-neck round-bottom flask. The system was evacuated and backfilled with nitrogen using a dual-manifold line to remove moisture and oxygen. Anhydrous tetrahydrofuran (40 mL), freshly distilled, was added to the system via syringe, and the mixture was cooled in a −78 °C low-temperature bath for 10 min. After the system was completely cooled, a 2.5 M solution of *n*-butyllithium (2.42 mL, 6.0 mmol) was added dropwise via syringe. After completion of the addition, the reaction mixture was maintained at −78 °C for

1 h. Acetone (0.22 mL, 3 mmol) was then added to the system, and the reaction was continued at −78 °C for an additional 1 h. Subsequently, water (20 mL) was added, the reaction mixture was removed from the low-temperature bath, and stirring was continued at room temperature for 1 h. The reaction vessel was opened, and the mixture was extracted with dichloromethane (50 mL). The organic phase was dried over anhydrous sodium sulfate, and the solvent was removed under reduced pressure using a rotary evaporator. The residue was redissolved in dry dichloromethane (30 mL) and cooled at 0 °C for 10 min. Anhydrous aluminum chloride (3.84 g, 28.8 mmol) was added portionwise, and the reaction mixture was stirred at room temperature overnight. After completion, water was added dropwise to quench the reaction. The mixture was extracted with dichloromethane, and the organic phase was dried over anhydrous sodium sulfate and concentrated under reduced pressure. The resulting material was used directly in the subsequent oxidation step without further purification.

Synthesis of compound 2–11:

The crude product 2–10 obtained above was dissolved in acetone (100 mL), and the mixture was cooled in a −5 °C low-temperature bath for 10 min. After the system was completely cooled, potassium permanganate (1.42 g, 9 mmol) was added portionwise and slowly over approximately 4 h. The reaction mixture was then maintained at −5 °C for 2 h, followed by warming to room temperature and stirring for an additional 4 h. The reaction progress was

monitored by TLC. After completion, the mixture was filtered through Celite to remove the manganese dioxide formed during the reaction. The filter cake was thoroughly washed with a dichloromethane/methanol mixed solvent (approximately 1000 mL) until the filtrate became colorless. The filtrate was concentrated under reduced pressure using a rotary evaporator. The resulting mixture was purified by silica gel column chromatography (200–300 mesh) using dichloromethane/methanol as the eluent to afford the target compound 2–11 as a yellow-green solid (239 mg, yield 25%). ^{1}H NMR (400 MHz, $CDCl_3$) δ (ppm): 7.50 (s, 2H), 6.53 (s, 2H), 3.89 (t, J = 7.2 Hz, 2H), 3.63 (t, J = 8.4 Hz, 4H), 3.10 (t, J = 8.2 Hz, 4H), 1.90 (t, J = 7.4 Hz, 2H), 1.59 (s, 6H), 0.93 (t, J = 7.4 Hz, 4H). ^{13}C NMR (101 MHz, $CDCl_3$) δ (ppm): 181.1, 152.4, 150.5, 129.2, 119.4, 110.8, 107.5, 47.5, 38.0, 33.8, 25.4. ESI(+)-HRMS (m/z): $[M + H]^+$ calcd. for: 361.2280, found: 361.2262.

Synthesis of compound I–C-ARh:

Compound 2–11 (133 mg, 0.4 mmol) was accurately weighed and dissolved in dichloromethane (20 mL). Pyridine (253 mg, 0.26 mL, 3.2 mmol) was added to the system via syringe, and the reaction mixture was cooled in a 0 °C low-temperature bath for 10 min. Trifluoromethanesulfonic anhydride (564 mg, 0.34 mL, 2.0 mmol) was then added by syringe, upon which the solution was immediately observed to change from light yellow to deep blue. The reaction mixture was maintained at 0 °C for 1 h. After TLC monitoring indicated complete consumption of compound 2–6, propylamine (236 mg, 0.33 mL, 4

mmol) was added by syringe. Upon addition of propylamine, the solution color instantaneously changed from deep blue to bright wine-red. The reaction mixture was then allowed to warm to room temperature and stirred for 10 min. The solvent was removed under reduced pressure using a rotary evaporator. The resulting reddish-brown mixture was purified by silica gel column chromatography (200–300 mesh) using dichloromethane/methanol as the eluent to afford the target compound I–C-ARh as a wine-red solid (80 mg, yield 54%). ^{1}H NMR (400 MHz, $CDCl_3$) δ (ppm): 7.49 (s, 2H), 6.55 (s, 2H), 3.89 (td, J = 7.1, 3.0 Hz, 2H), 3.62 (t, J = 8.4 Hz, 4H), 3.09 (td, J = 8.5, 1.3 Hz, 4H), 1.89 (q, J = 7.3 Hz, 2H), 1.59 (s, 6H), 0.93 (t, J = 7.4 Hz, 3H). ^{13}C NMR (101 MHz, $CDCl_3$) δ (ppm): 164.9, 157.0, 121.4, 118.2, 101.1, 54.6, 51.7, 41.1, 33.5, 32.4, 26.9, 23.0, 11.0. ESI(+)-HRMS (m/z): $[M + H]^+$ calcd. for: 374.2591, found: 374.2592.

Synthesis of compound 2–12:

m-Bromoaniline (10 g, 105 mmol) and potassium carbonate (18.2 g, 132 mmol) were accurately weighed and placed in a 250 mL round-bottom flask. Acetone (100 mL) was then added to dissolve the reaction mixture, followed by the addition of three iodine crystals. The reaction mixture was heated to 100 °C and allowed to react for 12 h. The reaction progress was monitored by TLC. After completion, the solvent was removed under reduced pressure using a rotary evaporator. The resulting mixture was purified by silica gel column chromatography (200–300 mesh) using petroleum ether/ethyl acetate as the eluent to afford the target compound 2–12 as a brown oil (10.8 g, yield 41%). ^{1}H

NMR (400 MHz, $CDCl_3$) δ (ppm): 8.01 (s, 2H), 7.14 (d, J = 1.9 Hz, 1H), 7.02 (dd, J = 8.2, 1.9 Hz, 1H), 6.97 (d, J = 8.2 Hz, 1H), 5.43 (q, J = 1.5 Hz, 1H), 1.95 (d, J = 1.4 Hz, 3H), 1.34 (s, 6H). ^{13}C NMR (101 MHz, $CDCl_3$) δ (ppm): 137.8, 129.2, 128.4, 125.5, 125.1, 123.9, 121.7, 120.4, 54.0, 27.8, 18.3. ESI(+)-HRMS (m/z): $[M + H]^+$ calcd. for: 252.0388, found: 252.0378.

Synthesis of compound 2–13:

Compound 2–12 (5 g, 20 mmol) and potassium carbonate (5.5 g, 40 mmol) were accurately weighed and placed in a 100 mL round-bottom flask. Acetonitrile (50 mL) was added to dissolve the mixture, after which methyl iodide (1.5 mL, 24 mmol) was added to the system via syringe. The reaction mixture was heated under reflux for 12 h. The reaction progress was monitored by TLC. After completion, the reaction mixture was filtered to remove insoluble potassium carbonate. The filtrate was collected, and the solvent was removed under reduced pressure using a rotary evaporator. The resulting mixture was purified by silica gel column chromatography (200–300 mesh) using petroleum ether/ethyl acetate as the eluent to afford the target compound 2–13 as a brown oil (3.3 g, yield 62%). ^1H NMR (400 MHz, $CDCl_3$) δ (ppm): 8.01 (s, 2H), 7.14 (d, J = 1.9 Hz, 1H), 7.02 (dd, J = 8.2, 1.9 Hz, 1H), 6.97 (d, J = 8.2 Hz, 1H), 5.43 (q, J = 1.5 Hz, 1H), 1.95 (d, J = 1.4 Hz, 3H), 1.34 (s, 6H). ^{13}C NMR (101 MHz, $CDCl_3$) δ (ppm): 137.8, 129.2, 128.4, 125.5, 125.1, 123.9, 121.7, 120.4, 54.0, 27.8, 18.3.
ESI(+)-HRMS (m/z): $[M + H]^+$ calcd. for: 266.0544, found: 266.0533.

Synthesis of compound 2–14:

DMF (20 mL) was added to a 250 mL round-bottom flask and the mixture was cooled at 0 °C for 10 min. Phosphorus oxychloride (7.7 mL, 82.5 mmol) was then added dropwise to the system via a constant-pressure dropping funnel, and the reaction was maintained at 0 °C for an additional 30 min. Subsequently, a DMF solution (50 mL) of compound 2–13 (14.6 g, 55 mmol) was added dropwise via a constant-pressure dropping funnel. After completion of the addition, the reaction mixture was heated to 80 °C and stirred for 8 h, with the reaction progress monitored by TLC. After completion, the reaction mixture was poured into 500 mL of ice water and extracted with dichloromethane. The combined organic phases were dried over anhydrous sodium sulfate, and the solvent was removed under reduced pressure using a rotary evaporator. The resulting mixture was purified by silica gel column chromatography (200–300 mesh) using petroleum ether/ethyl acetate as the eluent to afford the target compound 2–14 as a colorless oil (12.4 g, yield 77%). ^{1}H NMR (400 MHz, $CDCl_3$) δ (ppm): 10.00 (s, 1H), 7.50 (s, 1H), 6.55 (s, 1H), 5.28 (s, 1H), 2.86 (s, 3H), 1.95 (d, J = 1.4 Hz, 3H), 1.34 (s, 6H). ^{13}C NMR (101 MHz, $CDCl_3$) δ (ppm): 190.2, 150.2, 129.9, 129.7, 126.6, 123.8, 121.5, 121.2, 113.0, 57.9, 31.4, 28.9, 18.5. ESI(+)-HRMS (m/z): $[M + H]^+$ calcd. for: 294.0494, found: 294.0488.

Synthesis of compound 2–15:

Compound 2–14 (11.7 g, 40 mmol) was accurately weighed and placed in a 250 mL round-bottom flask, followed by the addition of methanol (100 mL). The mixture was cooled at 0 °C for 10 min, after which sodium borohydride

(1.82 g, 48 mmol) was added slowly in portions. The reaction was maintained at 0 °C for 30 min, with the progress monitored by TLC. After completion, saturated brine (100 mL) was added to the reaction mixture, followed by extraction with dichloromethane. The combined organic phases were dried over anhydrous sodium sulfate, and the solvent was removed under reduced pressure using a rotary evaporator. The resulting white solid was the pure target compound 2–15 (8.2 g, yield 99%). ^{1}H NMR (400 MHz, $CDCl_3$) δ (ppm): 7.02 (s, 1H), 6.63 (s, 1H), 5.29 (s, 1H), 4.61 (d, J = 6.2 Hz, 2H), 2.76 (s, 3H), 1.95 (d, J = 1.4 Hz, 3H), 1.27 (s, 6H). ^{13}C NMR (101 MHz, $CDCl_3$) δ (ppm): 145.9, 130.5, 127.3, 126.5, 124.6, 123.4, 122.5, 114.1, 65.2, 56.5, 30.8, 27.4, 18.5. ESI(+)-HRMS (m/z): $[M + H]^+$ calcd. for: 296.0650, found: 296.0655.

Synthesis of compound 2–16:

Compounds 2–13 (8.98 g, 33.89 mmol) and 2–15 (10 g, 33.89 mmol) were accurately weighed and placed in a 250 mL round-bottom flask, dissolved in dichloromethane (100 mL). Under stirring, boron trifluoride diethyl etherate (4.3 mL, 67.78 mmol) was added dropwise via syringe. The reaction was allowed to proceed at room temperature for 30 min, with the progress monitored by TLC. After completion, dilute hydrochloric acid (100 mL) was added, the layers were separated, and the organic phase was washed twice with saturated brine. The combined organic phases were dried over anhydrous sodium sulfate, and the solvent was removed under reduced pressure using a rotary evaporator. The resulting mixture was purified by silica gel column

chromatography (200–300 mesh), first eluting with petroleum ether/ethyl acetate and then increasing the polarity to dichloromethane/methanol, to afford the target compound 2–16 as a white solid (13.2 g, yield 72%). ^{1}H NMR (400 MHz, $CDCl_3$) δ (ppm): 6.75 (s, 2H), 6.69 (s, 2H), 5.26 (d, J = 1.3 Hz, 2H), 3.96 (s, 2H), 2.75 (s, 6H), 1.82 (s, 6H), 1.26 (s, 12H). ^{13}C NMR (101 MHz, $CDCl_3$) δ (ppm): 144.6, 130.4, 127.8, 126.6, 125.1, 124.5, 122.8, 114.3, 56.3, 39.8, 30.7, 26.9, 18.3. ESI(+)-HRMS (m/z): $[M + H]^+$ calcd. for: 543.1010, found: 543.1007.

Synthesis of compound 2–17:

Compound 2–16 (1.63 g, 3 mmol) was accurately weighed and placed in a 100 mL two-neck round-bottom flask. The system was evacuated and backfilled with nitrogen using a dual-manifold line to remove moisture and oxygen. Anhydrous tetrahydrofuran (40 mL), freshly distilled, was added via syringe, and the mixture was cooled in a −78 °C low-temperature bath for 10 min. After complete cooling, a 2.5 M solution of *n*-butyllithium (2.48 mL, 6.2 mmol) was added dropwise via syringe. After completion of the addition, the reaction was maintained at −78 °C for 1 h. Acetone (0.23 mL, 3.2 mmol) was then added, and the reaction was continued at −78 °C for an additional 1 h. Subsequently, water (20 mL) was added, the reaction mixture was removed from the low-temperature bath, and stirring was continued at room temperature for 1 h. The reaction vessel was opened, and the mixture was extracted with dichloromethane (50 mL). The organic phase was dried over anhydrous sodium sulfate, and the solvent was removed under reduced pressure using a

rotary evaporator. The residue was redissolved in dry dichloromethane (30 mL) and cooled at 0 °C for 10 min. Anhydrous aluminum chloride (3.99 g, 30 mmol) was added portionwise, and the reaction mixture was stirred at room temperature overnight. After completion, water was added dropwise to quench the reaction. The mixture was extracted with dichloromethane, and the organic phase was dried over anhydrous sodium sulfate and concentrated under reduced pressure. The resulting material was used directly in the subsequent oxidation step without further purification.

Synthesis of compound 2–18:

The crude product 2–17 obtained was dissolved in acetone (100 mL), and the system was placed in a −5 °C low-temperature bath and cooled for 10 min. After the system was completely cooled, potassium permanganate (1.42 g, 9 mmol) was added slowly in portions over approximately four hours. The reaction was then maintained at −5 °C for 2 h, followed by warming to room temperature and continued reaction for 4 h. The reaction progress was monitored by TLC. After completion, the reaction mixture was filtered through Celite to remove the generated manganese dioxide. The filter cake was thoroughly washed with a dichloromethane/methanol mixed solvent (approximately 1000 mL) until the filtrate became colorless. The combined filtrate was concentrated under reduced pressure using a rotary evaporator to remove the solvent. The resulting mixture was purified by silica gel column chromatography (200–300 mesh), eluting with dichloromethane/methanol, to afford the target compound

2–18 as an earthy yellow solid (542 mg, yield 41%). ^1H NMR (400 MHz, $CDCl_3$) δ (ppm): 8.00 (s, 2H), 6.52 (s, 2H), 5.29 (s, 2H), 2.92 (s, 6H), 2.06 (d, J = 1.2 Hz, 6H), 1.68 (s, 6H), 1.36 (s, 12H). ^{13}C NMR (101 MHz, $CDCl_3$) δ (ppm): 180.9, 152.4, 148.3, 129.3, 127.6, 122.1, 121.1, 119.6, 106.0, 57.2, 38.0, 33.4, 31.1, 28.6, 18.7. ESI(+)-HRMS (m/z): $[M + H]^+$ calcd. for: 441.2906, found: 441.2906.

Synthesis of compound Q–C-ARh:

Compound 2–18 (440 mg, 1 mmol) was accurately weighed and dissolved in dichloromethane (20 mL). Pyridine (632 mg, 0.64 mL, 8 mmol) was added to the system via syringe, and the reaction mixture was placed in a 0 °C low-temperature bath and cooled for 10 min. Subsequently, trifluoromethanesulfonic anhydride (1.41 g, 0.84 mL, 8.0 mmol) was added via syringe. An immediate color change from light yellow to deep blue was observed. The reaction was maintained at 0 °C for 1 h. After TLC monitoring confirmed the complete consumption of compound 2–18, propylamine (591 mg, 0.84 mL, 10 mmol) was added via syringe. Upon addition of propylamine, the color of the system instantly changed from deep blue to bright wine red. The reaction mixture was then allowed to warm to room temperature and stirred for 10 min. The solvent was removed under reduced pressure using a rotary evaporator. The resulting reddish-brown mixture was purified by silica gel column chromatography (200–300 mesh), eluting with dichloromethane/methanol, to afford the target compound Q–C-ARh as a wine-red solid (288 mg, yield 60%). ^1H NMR (400 MHz, $CDCl_3$) δ (ppm): 7.39

(s, 2H), 6.64 (s, 2H), 5.34 (s, 2H), 3.94 (t, J = 13.5 Hz, 2H), 2.98 (s, 6H), 2.01 (s, 6H), 1.95 (q, 2H), 1.62 (s, 6H), 1.40 (s, 12H), 0.98 (t, J = 7.4 Hz, 3H). ^{13}C NMR (101 MHz, $CDCl_3$) δ (ppm): 64.2, 149.3, 130.2, 125.9, 124.6, 121.4, 118.2, 115.0, 111.8, 105.9, 58.2, 51.2, 40.6, 32.4, 31.4, 29.0, 23.3, 18.3, 11.0. ESI(+)-HRMS (m/z): $[M + H]^+$ calcd. for: 482.3530, found: 482.3532.

UV–visible absorption and fluorescence emission spectra of the dyes.

After successfully synthesizing a series of rhodamine derivatives with different heteroatom substitutions and amino-group modifications, we first measured their UV–visible absorption spectra in PBS buffer. The experimental resuls show that the maximum absorption of P-C-ARh, I-C-ARh, and Q-C-ARh are 465 nm, 469nm, 504 nm, respectively (Figure S6). After determining the UV–visible absorption characteristics of the dyes, we further investigated the fluorescence spectra of this series of carbon-substituted rhodamine derivatives. The experimental results show that the maximum emission wavelengths of these three carbon rhodamines in PBS buffer are 600 nm, 633 nm, and 652 nm, respectively (Figure S7).

References

[1] Frisch, M. J.; Trucks, G. W.; Schlegel, H. B.; Scuseria, G. E.; Robb, M. A.; Cheeseman, J. R.; Scalmani, G.; Barone, V.; Mennucci, B.; Petersson, G. A.; Nakatsuji, H.; Caricato, M.; Li, X.; Hratchian, H. P.; Izmaylov, A. F.; Bloino, J.; Zheng, G.; Sonnenberg, J. L.; Hada, M.; Ehara, M.; Toyota, K.; Fukuda, R.; Hasegawa, J.; Ishida, M.; Nakajima, T.; Honda, Y.; Kitao, O.; Nakai, H.; Vreven, T.; Montgomery, J. A., Jr.; Peralta, J. E.; Ogliaro, F.; Bearpark, M.; Heyd, J. J.; Brothers, E.; Kudin, K. N.; Staroverov, V. N.; Kobayashi, R.; Normand, J.; Raghavachari, K.; Rendell, A.; Burant, J. C.; Iyengar, S. S.; Tomasi, J.; Cossi, M.; Rega, N.; Millam, J. M.; Klene, M.; Knox, J. E.; Cross, J. B.; Bakken, V.; Adamo, C.; Jaramillo, J.; Gomperts, R.; Stratmann, R. E.; Yazyev, O.; Austin, A. J.; Cammi, R.; Pomelli, C.; Ochterski, J. W.; Martin, R. L.; Morokuma, K.; Zakrzewski, V. G.; Voth, G. A.; Salvador, P.; Dannenberg, J. J.; Dapprich, S.; Daniels, A. D.; Farkas, Ö .; Foresman, J. B.; Ortiz, J. V.; Cioslowski, J.; Fox, D. J. *Gaussian 09, revision D.01*; Gaussian Inc.: Wallingford, CT, 2013.

[2] Zhou, P. Why the Lowest Electronic Excitations of Rhodamines Are Overestimated by Time-Dependent Density Functional Theory. *Int. J. Quantum Chem.* **2018,** 118 (23), e25780.
